# Aromatic C–H amination in hexafluoroisopropanol†
†Electronic supplementary information (ESI) available: Detailed experimental procedures and spectroscopic characterization for all new compounds. CCDC 1545194. For ESI and crystallographic data in CIF or other electronic format see DOI: 10.1039/c8sc04966a


**DOI:** 10.1039/c8sc04966a

**Published:** 2019-01-11

**Authors:** Erica M. D'Amato, Jonas Börgel, Tobias Ritter

**Affiliations:** a Department of Chemistry and Chemical Biology , Harvard University , 12 Oxford Street , Cambridge , Massachusetts 02138 , USA; b Max-Planck-Institut für Kohlenforschung , Kaiser-Wilhelm-Platz 1 , D-45470 Mülheim an der Ruhr , Germany . Email: ritter@mpi-muelheim.mpg.de

## Abstract

We report a direct radical aromatic amination reaction that provides unprotected anilines with an improvement in the substrate scope compared to prior art. Hydrogen bonding by the solvent hexafluoroisopropanol to anions of cationic species is responsible for increased reactivity and can rationalize the enhancement in substrate scope. Our findings may have bearings on radical additions to arenes for direct C–H functionalization in general.

## Abstract 2

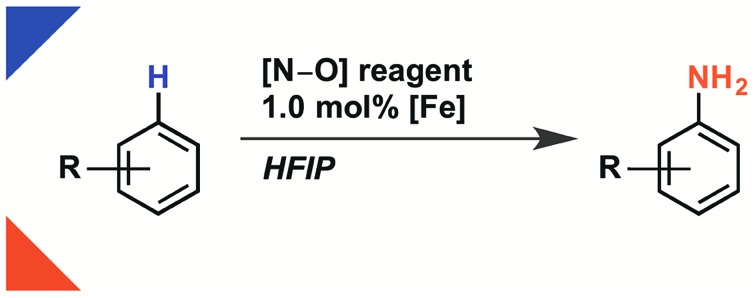
We report direct amination of electron-poor arenes and evaluate the crucial factors for the enhanced reactivity in hexafluoroisopropanol.

## Introduction

**Fig. 1:**
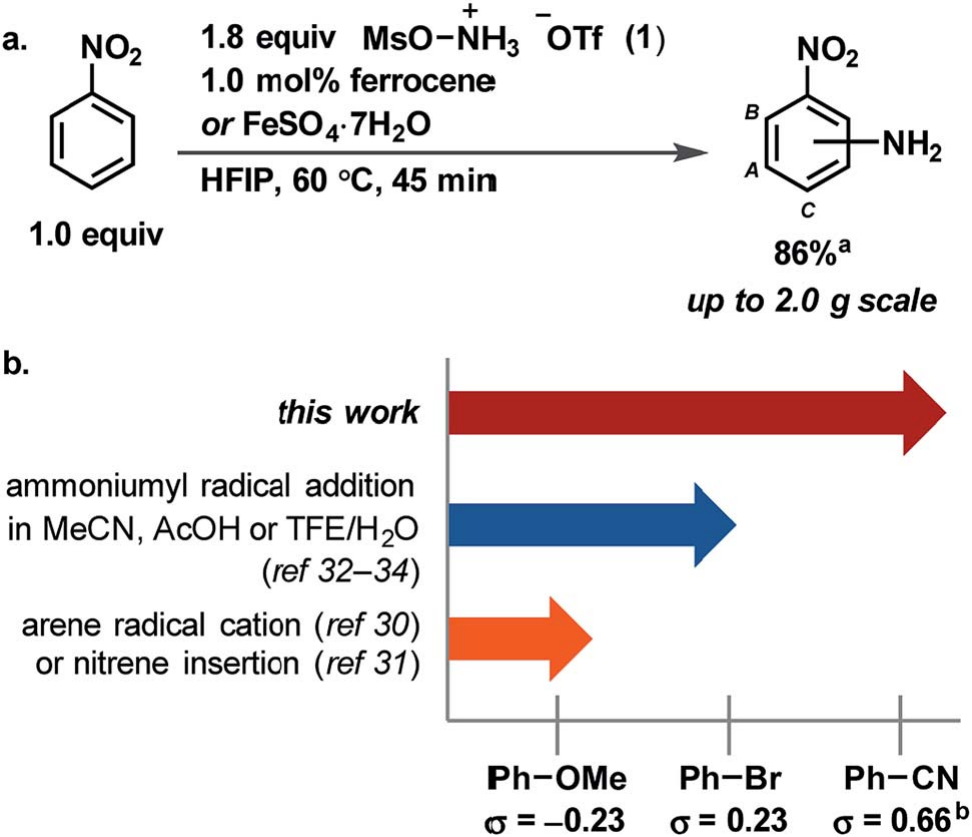
(a) [Fe]-catalyzed amination of nitrobenzene. (b)**.** Substrate scope of this work compared to prior art. ^a^Combined yield of isolated analytically pure individual isomers. Ratio A : B : C = 2.4 : 1.0 : 1.0. ^b^While *σ* values cannot be used to compare reactions proceeding through different mechanisms, they do provide a semi-quantitative measure of arene electron density.

Radical addition to arenes is an attractive strategy for aromatic C–H functionalization because it avoids the difficult C–H metalation step that is common to several C–H functionalization processes.^1^ Instead, radical addition can be followed by a facile C–H deprotonation as the final step. Radical addition to arenes has been used to install a variety of functional groups onto aromatic rings, including aryl, alkyl, hydroxyl and amino groups, and often delivers multiple isomeric products, which can be useful for small molecule diversification.^2^–^6^ However, the radical must be matched in polarity with the arene for a productive reaction.^7^–^10^ Therefore, the substrate scope of any particular radical addition is typically small, with electrophilic radicals reacting with nucleophilic arenes and *vice versa*. Herein, we demonstrate and rationalize the previously unappreciated, scope-expanding effect of the solvent hexafluoroisopropanol (HFIP) on a radical aromatic C–H amination that provides free anilines in a single step. Our reaction protocol enables efficient amination of electron-poor arenes, such as nitrobenzene (Fig. 1a). We discuss how ion pair disruption through specific hydrogen bonding interactions with HFIP can expand the substrate scope of a radical C–H functionalization and can obviate the need for the metal catalyst used in conventional reactions. Our hypothesis may provide a conceptual framework for the development of other, new radical addition reactions, and in the expansion of their scope.

Aryl amines are broadly useful in the pharmaceutical, agrochemical and material science fields and have been traditionally synthesized on scale using an electrophilic nitration/reduction sequence.^11^–^14^ While most modern aromatic C–H amination methods^15^–^27^ have failed to match the scope of electrophilic nitration, reaction development has succeeded in increasing functional group tolerance, reducing the two-step sequence to a one-step process, and improving the safety of the reaction. To date, only the addition of pyridinium radicals to arenes features a broad substrate scope of anilines in a 2-step sequence including electron-poor arenes.^28^,^29^ Advances in C–H amination to provide unprotected anilines have been reported by Nicewicz,^30^ under whose conditions ammonium carbamate traps an arene radical cation intermediate, and by Kürti and Falck,^31^ under whose conditions a rhodium nitrene intermediate is proposed to insert into a C–H bond. A third approach to direct aromatic C–H amination was pioneered by Minisci in the 1960s, in which hydroxylamine-O-sulfonic acid (HOSA) is used as an ammoniumyl radical precursor in the presence of iron(ii) salts.^2^,^32^ In 2016, Morandi revised the Minisci protocol with the use of the reagent [MsO–NH_3_]OTf (**1**).^33^ Moreover, in 2017, Jiao has demonstrated that ammoniumyl radicals, which were generated from different [RCO_2_–NH_3_]OTf reagents, add to a variety of electron-rich arenes in TFE/H_2_O.^34^ However, all reported modern amination methods to make anilines in a one-step procedure break down if an electron-poor arene is used as a substrate (Fig. 1b). For example, no reaction has been reported to afford more than 5% conversion with benzonitrile as a substrate, and most reactions do not afford synthetically useful yields for arenes less electron-rich than bromobenzene.

## Results and discussion

Herein, we show that the combination of the easy-to-handle hydroxylamine-derived reagent **1**, 1.0 mol% iron(ii) catalyst, and HFIP as solvent affords unprotected anilines from aromatic C–H bonds across an electronic range of arenes broader in scope than any reported modern aromatic C–H amination reaction. The reaction is characterized by a simple setup that does not require any special‡Cambridge Crystallographic Data Centre (CCDC) number of compound **1**: 1545194.precautions to exclude air or moisture, and by reaction times shorter than 2 h. In addition, multiple iron sources, including ferrocene and FeSO_4_·7H_2_O, are competent for the reaction. For example, on a 2.0 g scale, nitrobenzene is aminated in 86% yield within 45 min with 1.0 mol% iron loading (Fig. 1a). Each constitutional isomer was isolated as individual, analytically pure sample.

**Fig. 2:**
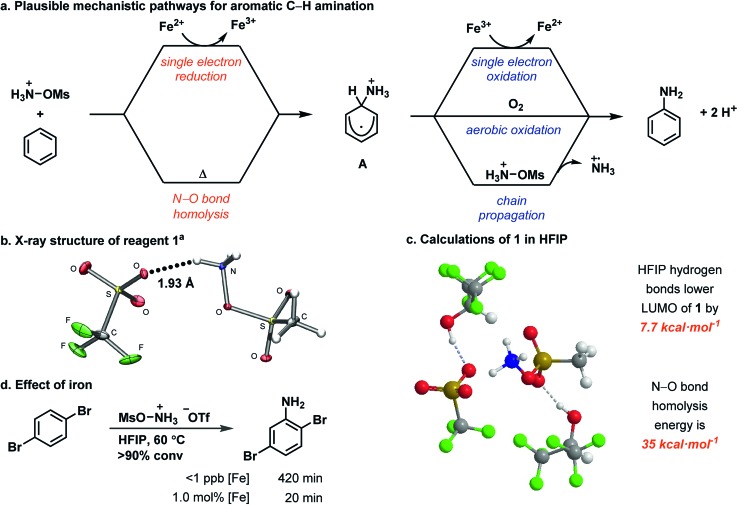
(a) Mechanistic hypothesis for aromatic C–H amination. (b) X-ray crystal structure of **1** crystallized from HFIP. (c) Calculated structure of **1** with explicit HFIP molecules. (d) Comparison of [Fe]-catalyzed and metal-free reactions. ^a^Structure shown with 50% probability ellipsoids.‡

The expansion of the scope is attributed to the unique properties of the solvent HFIP, including high polarity, low nucleophilicity, and strong hydrogen bond-donating ability.^35^,^36^ As a result, the use of HFIP has been shown to have a great effect on reactivity and/or selectivity in a number of reactions.^36^–^42^ We propose that HFIP increases the electrophilicity of several cationic species in our reaction through hydrogen-bonding with their anionic counterions, which in turn leads to effective amination of more electron-poor arenes. Such an increase in reactivity through hydrogen bonding has not been demonstrated previously for aromatic amination. Based on our experiments, we propose the mechanism shown in Fig. 2a: an ammoniumyl radical, generated either through N–O bond homolysis or iron-mediated single electron reduction, adds to an arene to generate a putative cationic cyclohexadienyl radical (**A**). Intermediate **A** then rearomatizes to the aniline product through one of three pathways: single electron oxidation by iron(iii),^43^–^45^ aerobic oxidation^46^–^50^ or chain propagation.^51^

We identified a hydrogen bond in reagent **1** between one N–H and the triflate counterion in the solid state (Fig. 2b). HFIP likely disrupts the internal hydrogen-bonding and ion pairing of **1**, because it functions as a hydrogen-bond donor to the triflate counterion but cannot function as a hydrogen-bond acceptor to [MsO–NH_3_]^+^.^52^,^53^ The result of the HFIP–triflate hydrogen bond is a less associated ion pair with a more localized cation on nitrogen. A second hydrogen bond can occur between an oxygen of the mesyl group and HFIP. Consequently, the cation of **1** is more electrophilic when it is dissolved in HFIP.§
§A mixture of MeCN and H_2_O, as was used in ref. 33, would not have the same effect on **1**, as the solvent can both donate and accept hydrogen bonds, and the zwitterionic nature of the reagent (HOSA) used by Minisci (ref. 32) mitigates any such disruption of ion pairing. DFT calculations with explicit HFIP solvent molecules support our hypothesis and show that the LUMO of reagent **1** is 7.7 kcal mol^–1^ lower in energy when both hydrogen-bonding interactions are present (Fig. 2c). The increased reactivity of reagent **1** in HFIP is confirmed by its reduction potential, which we measured by cyclic voltammetry in both HFIP (–0.77 V *vs.* Fc/Fc^+^) and MeCN (–1.28 V *vs.* Fc/Fc^+^) (see ESI†). The difference in the reduction potential of reagent **1** in the two solvents is ∼0.5 V and indicates that the reagent is a notably stronger oxidant in HFIP, which supports our hydrogen-bonding hypothesis. In addition, attempts to synthesize derivatives of **1** that contain counterions less capable of hydrogen bonding (*e.g.* PF_6_^–^) were unsuccessful, which suggests that the hydrogen bond between the triflate counterion and the reagent is an important stabilizing factor for **1** before it is activated by dissolution in HFIP (see ESI†).

HFIP may not only have an effect on the reactivity of **1** but also may increase the reactivity of cationic reaction intermediates, specifically the ammoniumyl radical and intermediate **A** (Fig. 2a), through hydrogen-bonding interactions with their triflate counterions. Decreased ion pairing and the lack of a hydrogen-bond accepting solvent would lower the LUMO of the ammoniumyl radical derived from the cation of **1** and would enable addition to more electron-poor arenes. Similarly, increased reactivity of the cationic cyclohexadienyl radical **A** would enable more efficient oxidation to the final product. The overall increased electrophilicity of **1**, the ammoniumyl radical, and **A** would synergize to give the much improved substrate scope presented herein.

HFIP also enables a metal-free reaction (<1 ppb Fe detected) to occur (Fig. 2d). Metal-free activation does not occur under any previously reported conditions for ammoniumyl radical addition to arenes but is viable due to the activating properties of HFIP, albeit with longer reaction times. Such a background reaction pathway is a common feature of radical chain reactions.^51^ Under metal-free conditions, we identified N–O bond homolysis as a likely initiation step to generate the ammoniumyl radical. The N–O bond homolysis energy was calculated using DFT (ωB97XD) to be 35 kcal mol^–1^ (Fig. 2c). N–O bond homolysis can therefore be considered feasible under the reaction conditions, regardless of the presence of an iron salt. Rearomatization of cyclohexadienyl radical **A** could then occur either by aerobic oxidation or chain propagation.

When FeSO_4_·7H_2_O is present in the reaction, formation of the ammoniumyl radical can occur through single electron reduction of reagent **1**. A third pathway for rearomatization then becomes available – single electron oxidation of **A** by iron(iii) generated in the reduction of **1**. Iron(iii) has been shown capable of oxidizing cyclohexadienyl radicals,^43^–^45^ but whether the iron(ii) salt in our reaction is turning over as a catalyst or acting as a radical chain initiator cannot be discerned from our data.

Based on our discovery of the beneficial effects of HFIP on radical C–H amination, we synthesized a number of anilines utilizing our new protocol with a primary focus on electron-deficient arenes (Table 1). While previous methods demonstrate efficient amination of arenes no more electron poor than bromobenzene to provide unprotected anilines, our method is suitable for the amination of arenes such as nitrobenzene (**2**), methyl phenyl sulfone (**3**), and benzonitrile (**4**). Reactivity is maintained with electron-rich arenes as well (see ESI†). Most halides are tolerated (**5**, **10**, **11**, **13**), as are tertiary amines (**11**) and benzylic C–H bonds (**7**, **13**). Amination can occur on five-membered heterocycles (**6**) and on benzofused five- and six-membered heterocycles (**7**, **9**). However, no amination has been observed on six-membered heterocycles. While esters (**6**, **9**), amides (**11**, **13**), nitriles (**10**, **4**), and sulfonamides (**8**) are suitable substrates, aldehydes, ketones, and alkenes typically undergo side reactions without appreciable ring amination. While for some densely functionalized substrates, no or low conversion of starting material was observed (see Table S6†), we demonstrated the utility of our method to late-stage functionalization of drug molecules such as moclobemide and rufinamide, which were aminated to give derivatives **11** and **13**, respectively.

**Table 1:** Aromatic C–H amination in hexafluoroisopropanol

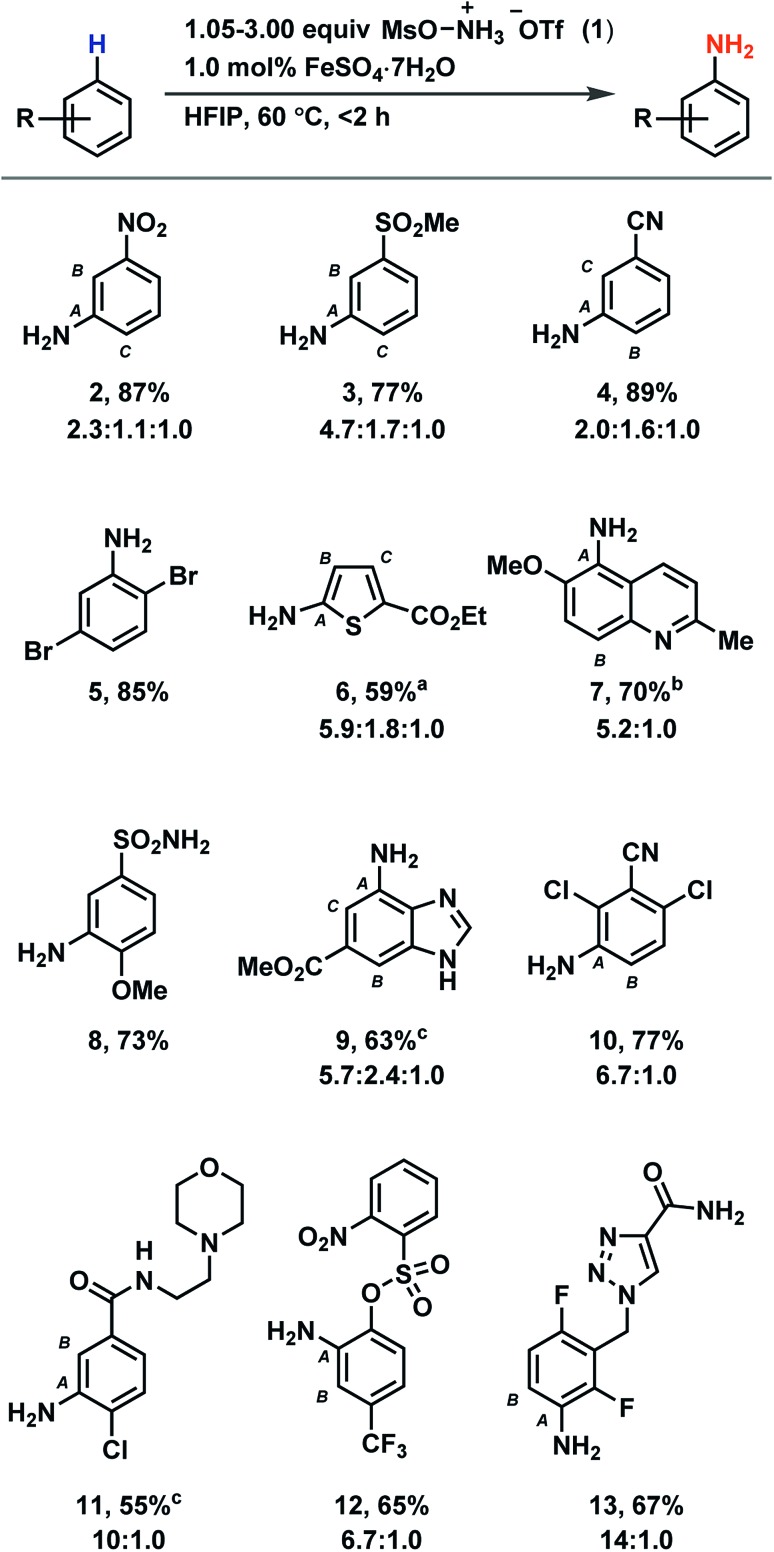

^*a*^Performed at 40 °C.

^*b*^Performed under an atmosphere of oxygen.

^*c*^TfOH (1.00 equiv.) added.

## Conclusions

We present a practical aromatic C–H amination reaction and provide a mechanistic framework for understanding the effect of the solvent HFIP on the reaction. Though aminiumyl radical additions have been known for over half a century, the mechanistic insight presented herein has resulted in a previously unrealized reaction utility. HFIP is proposed to comprise a unique solvent environment that increases the electrophilicity of multiple cationic species in the reaction to provide a drastically expanded substrate scope. We anticipate that our findings will inform further investigation and development of radical addition reactions for aromatic C–H functionalization.

## Conflicts of interest

There are no conflicts to declare.

## Supplementary Material

Supplementary information

Crystal structure data
