# Supplementary material for: Aromatic C–H amination in hexafluoroisopropanol
Source: Chem Sci. 2019 Jan 11;10(8):2424–8. doi: 10.1039/c8sc04966a (PMC6385662; doi:10.1039/c8sc04966a)

Electronic Supplementary Material (ESI) for Chemical Science.
This journal is © The Royal Society of Chemistry 2019

ELECTRONIC SUPPLEMENTARY INFORMATION

Aromatic C–H Amination in Hexafluoroisopropanol

Erica M. D'Amato,[a] Jonas Börgel[b] and Tobias Ritter*[ab]

[a]Department of Chemistry and Chemical Biology, Harvard University
12 Oxford Street, Cambridge, Massachusetts 02138, United States

[b]Max-Planck-Institut für Kohlenforschung
Kaiser-Wilhelm-Platz 1, D-45470 Mülheim an der Ruhr, Germany

*E-mail: ritter@mpi-muelheim.mpg.de

TABLE OF CONTENTS

TABLE OF CONTENTS 1

MATERIALS AND METHODS 6

EXPERIMENTAL DATA 8

General procedure for amination 8

Preparation of reagent **1** 8

N-Boc-*O*-Mesylhydroxylamine (**S1**) 8

[MsO–NH_3]OTf (**1**) 9

Preparation of substrates for amination 9

Moclebomide (**S2**) 9

4-(Trifluoromethyl)phenyl-2-nitrobenzenesulfonate (**S3**) 10

Amination of arenes 11

3-Nitroaniline (**2a**), 2-nitroaniline (**2b**), and 4-nitroaniline (**2c**) 11

3-(Methylsulfonyl)aniline (**3a**), 2-(methylsulfonyl)aniline (**3b**), and 4-(methylsulfonyl)aniline (**3c**) 12

3-Aminobenzonitrile (**4a**), 4-aminobenzonitrile (**4b**), and 2-aminobenzonitrile (**4c**) 13

2,5-Dibromoaniline (**5**) 14

Ethyl 5-aminothiophene-2-carboxylate (**6a**), ethyl 4-aminothiophene-2-carboxylate (**6b**), and ethyl 3-aminothiophene-2-carboxylate (**6c**) 15

5-Amino-6-methoxy-2-methylquinoline (**7a**) and 8-amino-6-methoxy-2-methylquinoline (**7b**) 16

3-Amino-4-methoxybenzenesulfonamide (**8**) 17

Methyl 4-amino-1*H*-benzimidazole-6-carboxylate (**9a**), methyl 7-amino-1*H*-benzimidazole-6-carboxylate (**9b**) and methyl 5-amino-1*H*-benzimidazole-6-carboxylate (**9c**) 18

3-Amino-2,6-dichlorobenzonitrile (**10a**) and 4-amino-2,6-dichlorobenzonitrile (**10b**) 19

3-Aminomoclebomide (**11a**) and 2-Aminomoclebomide (**11b**) 20

2-Amino-4-(trifluoromethyl)phenyl 2-nitrobenzenesulfonate (**12a**) and 3-amino-4-(trifluoromethyl)phenyl 2-nitrobenzenesulfonate (**12b**) 21

3-Aminorufinamide (**13a**) and 4-aminorufinamide (**13b**) 23

Gram-scale amination reaction 24

Comparison to other amination methods 24

4-Bromoaniline (**S4a**), 2-bromoaniline (**S4b**), and 3-bromoaniline (**S4c**) 25

4-Methoxyaniline (**S5a**), 2-methoxyaniline (**S5b**), and 3-methoxyaniline (**S5c**) 27

Effect of iron source, oxygen, and light on the amination reaction 29

Effect of iron and oxygen 29

Effect of iron source 30
Effect of light 31
Trace metal analysis 31
Consumption studies of reagent **1** 32
Synthesis of reagent **1** with other counterions 33
[MsO–NH_3]ONf (**S6**) 33
Electrochemical data 34
General methods 34
[MsO–NH_3]OTf (**1**) in HFIP 34
[MsO–NH_3]OTf (**1**) in MeCN 35
Comparison of electrochemical data 35
Failed substrates 36
DFT CALCULATIONS 37
DFT results for **1** in HFIP 37
DFT results for **1** in MeCN 38
DFT results for HFIP 39
DFT results for **1**·HFIP (OMs) 40
DFT results for **1**·HFIP (OTf) 42
DFT results for **1**·2HFIP 44
DFT results for $[NH_3]^{+\cdot}$(OTf) (**S7**) 46
DFT results for $MsO^{\cdot}$ (**S8**) 47
Calculation of homolysis energy 47
Comparison of LUMO energy differences and reduction potentials 48
X-RAY CRYSTALLOGRAPHIC ANALYSIS 49
[MsO–NH_3]OTf (**1**) (CCDC 1545194) 49
REFERENCES 51
SPECTROSCOPIC DATA 53
^{1}H NMR of *N*-Boc-*O*-mesylhydroxylamine (**S1**) 53
^{13}C NMR of *N*-Boc-*O*-mesylhydroxylamine (**S1**) 54
^{1}H NMR of [MsO–NH_3]OTf (**1**) 55
^{13}C NMR of [MsO–NH_3]OTf (**1**) 56
^{19}F NMR of [MsO–NH_3]OTf (**1**) 57

^{1}H NMR of moclebomide (**S2**)......58

^{13}C NMR of moclebomide (**S2**)......59

^{1}H NMR of 4-(trifluoromethyl)phenyl-2-nitrobenzenesulfonate (**S3**)......60

^{13}C NMR of 4-(trifluoromethyl)phenyl-2-nitrobenzenesulfonate (**S3**)......61

^{19}F NMR of 4-(trifluoromethyl)phenyl-2-nitrobenzenesulfonate (**S3**)......62

^{1}H NMR of 3-nitroaniline (**2a**)......63

^{13}C NMR of 3-nitroaniline (**2a**)......64

^{1}H NMR of 2-nitroaniline (**2b**)......65

^{13}C NMR of 2-nitroaniline (**2b**)......66

^{1}H NMR of 4-nitroaniline (**2c**)......67

^{13}C NMR of 4-nitroaniline (**2c**)......68

^{1}H NMR of 3-(methylsulfonyl)aniline (**3a**)......69

^{13}C NMR of 3-(methylsulfonyl)aniline (**3a**)......70

^{1}H NMR of 2-(methylsulfonyl)aniline (**3b**)......71

^{13}C NMR of 2-(methylsulfonyl)aniline (**3b**)......72

^{1}H NMR of 4-(methylsulfonyl)aniline (**3c**)......73

^{13}C NMR of 4-(methylsulfonyl)aniline (**3c**)......74

^{1}H NMR of 3-aminobenzonitrile (**4a**)......75

^{13}C NMR of 3-aminobenzonitrile (**4a**)......76

^{1}H NMR of 4-aminobenzonitrile (**4b**)......77

^{13}C NMR of 4-aminobenzonitrile (**4b**)......78

^{1}H NMR of 2-aminobenzonitrile (**4c**)......79

^{13}C NMR of 2-aminobenzonitrile (**4c**)......80

^{1}H NMR of 2,5-dibromoaniline (**5**)......81

^{13}C NMR of 2,5-dibromoaniline (**5**)......82

^{1}H NMR of ethyl 5-aminothiophene-2-carboxylate (**6a**)......83

^{13}C NMR of ethyl 5-aminothiophene-2-carboxylate (**6a**)......84

^{1}H NMR of ethyl 4-aminothiophene-2-carboxylate (**6b**)......85

^{13}C NMR of ethyl 4-aminothiophene-2-carboxylate (**6b**)......86

^{1}H NMR of ethyl 3-aminothiophene-2-carboxylate (**6c**) 87

^{13}C NMR of ethyl 3-aminothiophene-2-carboxylate (**6c**) 88

^{1}H NMR of 5-amino-6-methoxy-2-methylquinoline (**7a**) 89

^{13}C NMR of 5-amino-6-methoxy-2-methylquinoline (**7a**) 90

^{1}H NMR of 8-amino-6-methoxy-2-methylquinoline (**7b**) 91

^{13}C NMR of 8-amino-6-methoxy-2-methylquinoline **(7b**) 92

^{1}H NMR of 3-amino-4-methoxybenzenesulfonamide (**8**) 93

^{13}C NMR of 3-amino-4-methoxybenzenesulfonamide (**8**) 94

^{1}H NMR of methyl 4-amino-1*H*-benzimidazole-6-carboxylate (**9a**) and methyl 5-amino-1*H*-benzimidazole-6-carboxylate (**9c**) 95

^{13}C NMR of methyl 4-amino-1*H*-benzimidazole-6-carboxylate (**9a**) and methyl 5-amino-1*H*-benzimidazole-6-carboxylate (**9c**) 96

^{1}H NMR of methyl 7-amino-1*H*-benzimidazole-6-carboxylate (**9b**) 97

^{13}C NMR of methyl 7-amino-1*H*-benzimidazole-6-carboxylate (**9b**) 98

^{1}H NMR of 3-amino-2,6-dichlorobenzonitrile (**10a**) 99

^{13}C NMR of 3-amino-2,6-dichlorobenzonitrile (**10a**) 100

^{1}H NMR of 4-amino-2,6-dichlorobenzonitrile (**10b**) 101

^{13}C NMR of 4-amino-2,6-dichlorobenzonitrile (**10b**) 102

^{1}H NMR of 3-aminomoclebomide (**11a**) 103

^{13}C NMR of 3-aminomoclebomide (**11a**) 104

^{1}H NMR of 2-aminomoclebomide (**11b**) 105

^{13}C NMR of 2-aminomoclebomide (**11b**) 106

^{1}H NMR of 2-amino-4-(trifluoromethyl)phenyl 2-nitrobenzenesulfonate (**12a**) 107

^{13}C NMR of 2-amino-4-(trifluoromethyl)phenyl 2-nitrobenzenesulfonate (**12a**) 108

^{19}F NMR of 2-amino-4-(trifluoromethyl)phenyl 2-nitrobenzenesulfonate (**12a**) 109

^{1}H NMR of 3-amino-4-(trifluoromethyl)phenyl 2-nitrobenzenesulfonate (**12b**) 110

^{13}C NMR of 3-amino-4-(trifluoromethyl)phenyl 2-nitrobenzenesulfonate (**12b**) 111

^{19}F NMR of 3-amino-4-(trifluoromethyl)phenyl 2-nitrobenzenesulfonate (**12b**) 112

^{1}H NMR of 3-aminorufinamide (**13a**) and 4-aminorufinamide (**13b**) 113

^{13}C NMR of 3-aminorufinamide (**13a**) and 4-aminorufinamide (**13b**) 114
^{19}F NMR of 3-aminorufinamide (**13a**) and 4-aminorufinamide (**13b**) 115
^{1}H NMR of 4-bromoaniline (**S4a**) 116
^{13}C NMR of 4-bromoaniline (**S4a**) 117
^{1}H NMR of 2-bromoaniline (**S4b**) 118
^{13}C NMR of 2-bromoaniline (**S4b**) 119
^{1}H NMR of 3-bromoaniline (**S4c**) 120
^{13}C NMR of 3-bromoaniline (**S4c**) 121
^{1}H NMR of 4-methoxyaniline (**S5a**) 122
^{13}C NMR of 4-methoxyaniline (**S5a**) 123
^{1}H NMR of 2-methoxyaniline (**S5b**) 124
^{13}C NMR of 2-methoxyaniline (**S5b**) 125
^{1}H NMR of 3-methoxyaniline (**S5c**) 126
^{13}C NMR of 3-methoxyaniline (**S5c**) 127
^{1}H NMR of [MsO–NH_3]ONf (**S6**) 128
^{13}C NMR of [MsO–NH_3]ONf (**S6**) 129
^{19}F NMR of [MsO–NH_3]ONf (**S6**) 130

MATERIALS AND METHODS

All manipulations were carried out under ambient atmosphere unless otherwise noted.

Solvents

HFIP was purchased from Oakwood Chemicals and used as received except where noted. Where it is noted that HFIP was distilled and degassed, HFIP was distilled from 3Å molecular sieves and degassed by the freeze-pump-thaw method. Anhydrous diethyl ether, tetrahydrofuran, dichloromethane and acetonitrile were obtained by filtration through drying columns on an mBraun system.[1]

Chromatography

Thin layer chromatography (TLC) was performed using EMD TLC silica gel 60 F_{254} plates pre-coated with 250 μm thickness silica gel and visualized by fluorescence quenching under UV light and $KMnO_4$ stain. Preparative TLC was performed using Analtech Uniplates pre-coated with 1000 μm thickness silica gel GF with a volume of the mobile phase of ~100 mL. Flash chromatography was performed using silica gel (230-400 mesh) purchased from Silicycle Inc. Detailed flash column chromatography specifications are given for amination of the substrate ethyl 2-thiophenecarboxylate as a representative example.

Spectroscopy and Instruments

NMR spectra were recorded on either a Varian Unity/Inova 600 spectrometer operating at 600 MHz for ^1H acquisitions or a Varian Unity/Inova 500 spectrometer operating at 500 MHz, 470 MHz and 125 MHz for ^1H, ^{19}F and ^{13}C acquisitions, respectively or a Varian Mercury 400 spectrometer operating at 400 MHz and 375 MHz for ^1H and ^{19}F acquisitions, respectively. Chemical shifts are reported in ppm with the solvent resonance as the internal standard. For ^1H NMR: $CDCl_3$, δ 7.26; CD_3CN, δ 1.94; $(CD_3)_2CO$, δ 2.05; $(CD_3)_2SO$, δ 2.50; CD_2Cl_2, δ 5.32; CD_3OD, δ 3.31. For ^{13}C NMR: $CDCl_3$, δ 77.16; CD_3CN, δ 1.32; $(CD_3)_2CO$, δ 29.84; $(CD_3)_2SO$, δ 39.52; CD_2Cl_2, δ 53.84; CD_3OD, δ 49.00.[2] Chemical shifts for ^{19}F acquisitions were externally referenced to 3-nitro-1-fluorobenzene (δ −112.0). Data is reported as follows: s = singlet, d = doublet, t = triplet, q = quartet, m = multiplet, br = broad; coupling constants in Hz; integration. High resolution mass spectra were obtained using an Agilent ESI-TOF (6220) mass spectrometer. ICP-MS analysis was performed by Robertson Microlit Laboratories. Electrochemical measurements were made using a CH Instruments Model 600E Series Electrochemical Analyzer/Workstation.

Computational details

Density functional theory (DFT) calculations were performed using Gaussian09[3] at the computer cluster at the Max-Planck Institute für Kohlenforschung. Basis set I (BS I) includes 6-31G(d,p)[4] on H and 6-311G(d)[8] on C, N, O, F, S.

Geometry optimizations and frequency calculations were carried out at the ωB97XD[5]/BS I level using the atomic coordinates of the crystal structure of **1**. Explicit solvent molecules have been added using

GaussView5. The conductor-like polarizable continuum model (CPCM) has been used to simulate solvent effects (1,1,1,3,3,3-hexafluoro-2-propanol (HFIP), acetonitrile).[6,7] Ground state energies are given with respect to the thermal free energy correction at 298.15 K. Time-dependent DFT (TD-DFT) calculations have been carried out using the coordinates of the optimized ground state structures. Images of molecular structures and orbital plots were generated using GaussView5 and Chem3D.

Starting materials

All substrates were used as received from commercial suppliers, unless otherwise stated. $FeSO_4 \cdot 7H_2O$ was ground into a fine powder before use.

EXPERIMENTAL DATA

General procedure for amination

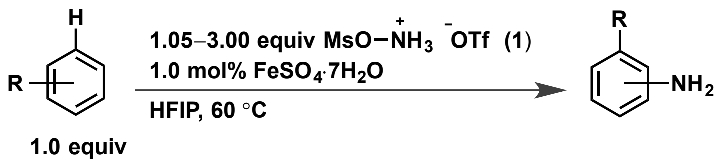

Reagent **1** (1.05–3.00 equiv), $FeSO_4 \cdot 7H_2O$ (0.8 mg, 3 μmol, 0.01 equiv), and the arene (if solid) (0.300 mmol, 1.00 equiv) were added to a 4-mL vial, followed by HFIP (1.5 mL, c = 0.2 M) and the arene (if liquid). The reaction mixture was stirred at 60 °C for 15–120 min, or until judged complete by the color and/or TLC. The reaction mixture was allowed to cool to room temperature and was concentrated. The residue was dissolved in 10 mL ethyl acetate and poured into a separatory funnel containing 10 mL sat. aq. Na_2CO_3. The layers were separated, and the aqueous layer was extracted with ethyl acetate (2 × 10 mL). The combined organic layers were dried over sodium sulfate, filtered and concentrated. The residue was purified by column chromatography and/or preparative TLC.

Preparation of reagent 1

N-Boc-*O*-Mesylhydroxylamine (S1)

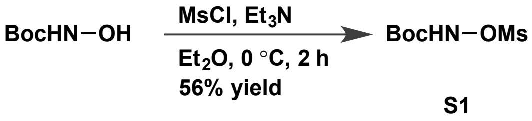

N-Boc-hydroxylamine (5.00 g, 37.6 mmol, 1.00 equiv) and triethylamine (3.81 g, 5.24 mL, 37.6 mmol, 1.00 equiv) were dissolved in anhydrous diethyl ether (190 mL, c = 0.2 M) in a flame-dried round bottom flask. The reaction mixture was cooled in a water-ice bath. Methanesulfonyl chloride (4.31 g, 2.91 mL, 37.6 mmol, 1.00 equiv) was then added slowly over 1 minute. The mixture was stirred at 0 ºC for 2 h, then allowed to warm to room temperature. The mixture was filtered over Celite, and the filtrate was concentrated. The residue was purified by column chromatography on silica gel eluting with a solvent mixture of ethyl acetate/hexane (15:85 (v/v)) to afford 4.43 g of the title compound as a colorless solid (56% yield). Spectroscopic data matched those previously reported.[8]

R_f = 0.92 (ethyl acetate/hexanes, 40:60 (v/v)).

NMR Spectroscopy:

^{1}H NMR (500 MHz, $CDCl_3$, 23 ºC, δ): 7.83 (br s, 1H), 3.18 (s, 3H), 1.52 (s, 9H).

^{13}C NMR (125 MHz, $CDCl_3$, 23 ºC, δ): 154.9, 84.8, 36.3, 28.1.

HRMS-FIA(m/z) calc'd for $C_6H_{17}N_2O_5S$ $[M+NH_4]^+$, 229.0858; found, 229.0860.

[MsO–NH$_3$]OTf (1)

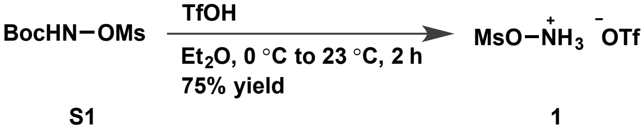

Reagent **1** was synthesized by the method of Morandi[9a] and Fagnou[9b]. Compound **S1** (1.50 g, 7.10 mmol, 1.00 equiv) was dissolved in anhydrous diethyl ether (36 mL, c = 0.2 M) in a flame-dried two-neck round bottom flask. The flask was evacuated and backfilled with nitrogen, then cooled in a water-ice bath. Triflic acid (1.07 g, 627 µL, 7.10 mmol, 1.00 equiv) was added using a plastic pipettor. The reaction mixture was stirred at 23 ºC for 2 h, during which time a colorless precipitate formed. Pentane (20 mL) was added to the flask. The colorless solid was collected on a Buchner funnel, rinsed with pentane (20 mL) and dried under high vacuum to give 1.39 g of the title compound as a colorless solid (75% yield). Spectroscopic data matched those previously reported.[9a]

NMR Spectroscopy:

^{1}H NMR (500 MHz, CD_3CN, 23 ºC, δ): 8.01–7.82 (br s, 3H), 3.36 (s, 3H).

^{13}C NMR (125 MHz, CD_3CN, 23 ºC, δ): 121.4 (q, J = 317 Hz), 39.9.

^{19}F NMR (375 MHz, CD_3CN, 23 ºC, δ): –79.8.

HRMS-FIA(m/z) calc'd for CH_6NO_3S $[M]^+$, 112.0068; found, 112.0069.

Preparation of substrates for amination

Moclebomide (S2)

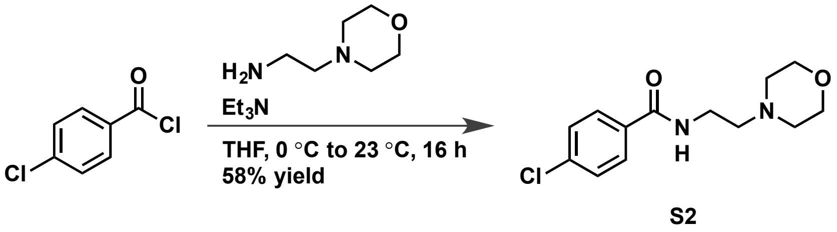

Moclebomide (**S2**) was synthesized by the method of Ahn.[10] 4-Chlorobenzoyl chloride (1.33 g, 977 µL, 7.62 mmol, 1.00 equiv) was dissolved in anhydrous tetrahydrofuran (35 mL, c = 0.2 M) in a flame-dried round bottom flask. The solution was cooled in a water-ice bath. Triethylamine (771 mg, 1.06 mL, 7.62 mmol, 1.00 equiv) and *N*-2-(aminoethyl)morpholine (992 mg, 1.00 mL, 7.62 mmol, 1.00 equiv) were then added. The reaction mixture was allowed to warm to room temperature and stir for 16 h at 23 ºC. The reaction mixture was poured into a separatory funnel containing EtOAc (100 mL) and water (200 mL). The layers were separated, and the aqueous layer was extracted with EtOAc (1 × 100 mL). The combined organic layers were dried over Na_2SO_4, filtered and concentrated. The residue was recrystallized from a mixture of EtOAc and hexanes at –5 ºC. The solid was collected on a Buchner funnel and washed with pentane (100 mL) to give 1.19 g of the title compound as a tan solid (58% yield).

R*f* = 0.13 (ethyl acetate/hexanes, 40:60 (v/v)).

NMR Spectroscopy:

^{1}H NMR (500 MHz, $CDCl_3$, 23 ºC, δ): 7.72 (d, *J* = 7.9 Hz, 2H), 7.42 (d, *J* = 8.5 Hz, 2H), 6.72 (br s, 1H), 3.74 (br s, 4H), 3.56 (br s, 2H), 2.62 (br s, 2H), 2.52 (br s, 4H).

^{13}C NMR (125 MHz, $CDCl_3$, 23 ºC, δ): 166.4, 137.7, 133.1, 128.9, 128.4, 67.1, 56.9, 53.4, 36.2.

HRMS-FIA(m/z) calc'd for $C_{13}H_{18}ClN_2O_2$ $[M+H]^+$, 269.1051; found, 269.1047.

4-(Trifluoromethyl)phenyl-2-nitrobenzenesulfonate (S3)

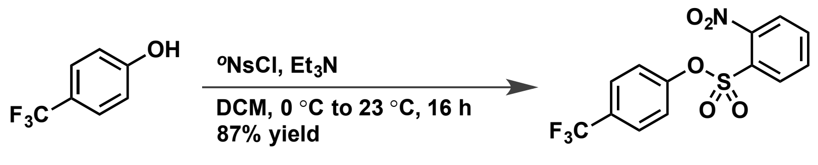

S3

The title compound was synthesized by the method of Williams.[11] 4-(Trifluoromethyl)phenol (400. mg, 2.47 mmol, 1.00 equiv) was dissolved in anhydrous dichloromethane (12 mL, c = 0.2 M) in a flame-dried round bottom two neck flask. Triethylamine (250 mg, 344 µL, 2.47 mmol, 1.00 equiv) was added and the mixture was cooled in a water-ice bath. 2-Nitrobenzenesulfonyl chloride (547 mg, 2.47 mmol, 1.00 equiv) was then added. The reaction mixture was stirred at 23 ºC for 16 h. The reaction mixture was then poured into a separatory funnel containing 1M HCl (aq) (25 mL). The layers were separated and the aqueous layer was extracted with dichloromethane (2 × 15 mL). The combined organic layers were dried over Na_2SO_4, filtered and concentrated. The residue was purified by column chromatography on a short plug of silica gel eluting with a solvent mixture of ethyl acetate/pentane (30:70 (v/v)). Purification afforded 747 mg of the title compound as a colorless solid (87% yield).

R*f* = 0.54 (ethyl acetate/hexanes, 40:60 (v/v)).

NMR Spectroscopy:

^{1}H NMR (500 MHz, $CDCl_3$, 23 ºC, δ): 8.00 (d, *J* = 7.6 Hz, 1H), 7.90–7.84 (m, 2H), 7.76–7.70 (m, 1H), 7.64 (d, *J* = 8.4 Hz, 2H), 7.36 (d, *J* = 8.5 Hz, 2H).

^{13}C NMR (125 MHz, $CDCl_3$, 23 ºC, δ): 151.4, 148.8, 135.9, 132.4, 132.2, 130.1 (q, *J* = 32.9 Hz), 128.2, 127.5 (q, *J* = 3.7 Hz), 125.2, 123.6 (q, *J* = 271 Hz), 122.9.

^{19}F NMR (470 MHz, $CDCl_3$, 23 ºC, δ): –62.5.

HRMS-FIA(m/z) calc'd for $C_6H_{17}N_2O_5S$ $[M+NH_4]^+$, 365.0414; found, 365.0410.

Amination of arenes

3-Nitroaniline (2a), 2-nitroaniline (2b), and 4-nitroaniline (2c)

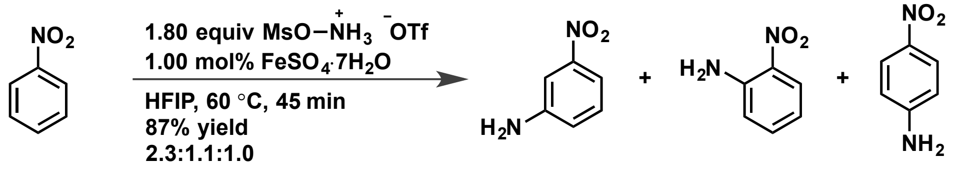

2a 2b 2c

Reagent **1** (141 mg, 0.540 mmol, 1.80 equiv) and $FeSO_4 \cdot 7H_2O$ (0.8 mg, 3 µmol, 0.01 equiv) were added to a 4-mL vial, followed by HFIP (1.5 mL, c = 0.2 M) and nitrobenzene (36.9 mg, 30.8 µL, 0.300 mmol, 1.00 equiv). The reaction mixture was stirred at 60 °C for 45 min. The orange reaction mixture was allowed to cool to room temperature and was concentrated. The residue was dissolved in 10 mL ethyl acetate and poured into a separatory funnel containing 10 mL sat. aq. Na_2CO_3. The layers were separated, and the aqueous layer was extracted with ethyl acetate (2 × 10 mL). The combined organic layers were dried over sodium sulfate, filtered and concentrated. A ^{1}H NMR spectrum of the residue indicated a ratio of **2a**:**2b**:**2c** = 2.3:1.1:1.0 by integrating the signal of **2a** at 6.94 ppm, the signal of **2b** at 6.76 ppm, and the signal of **2c** at 6.62 ppm. The residue was purified by column chromatography on silica gel eluting with a gradient solvent system, starting with a solvent mixture of diethyl ether/pentane (20:80 (v/v)) and finishing with diethyl ether/pentane (50:50 (v/v)). Purification afforded 2-nitroaniline (**2b**) in one fraction (8.1 mg) and 3-nitroaniline (**2a**) and 4-nitroaniline (**2c**) in a second fraction (27.8 mg) for a combined yield of 35.9 mg (87% yield). The second fraction was further purified by preparative TLC using a solvent system of diethyl ether/pentane (20:80 (v/v)) to give **2a** (14.9 mg) and **2c** (9.7 mg) as separate samples. Characterization data matched previously reported data for **2a**, **2b**, and **2c**.[12]

3-Nitroaniline (**2a**):

R*f* = 0.50 (ethyl acetate/hexanes, 40:60 (v/v)).

NMR Spectroscopy:

^{1}H NMR (500 MHz, $CDCl_3$, 23 ºC, δ): 7.57 (ddd, *J* = 8.2, 2.1, 0.9 Hz, 1H), 7.49 (dd, *J* = 2.2, 2.2 Hz, 1H), 7.27 (dd, *J* = 8.1, 8.1 Hz, 1H), 6.94 (ddd, *J* = 8.0, 2.3, 0.8 Hz, 1H), 4.00 (br s, 2H).

^{13}C NMR (125 MHz, $CDCl_3$, 23 ºC, δ): 149.4, 147.6, 130.0, 120.7, 113.3, 109.2.

HRMS-FIA(m/z) calc'd for $C_6H_7N_2O_2$ $[M+H]^+$, 139.0502; found, 139.0506.

2-Nitroaniline (**2b**):

R*f* = 0.35 (diethyl ether/pentanes, 30:70 (v/v)).

NMR Spectroscopy:

^{1}H NMR (500 MHz, $CDCl_3$, 23 ºC, δ): 8.12 (d, *J* = 8.6 Hz, 1H), 7.36 (dd, *J* = 7.7, 7.7 Hz, 1H), 6.80 (d, *J* = 8.4 Hz, 1H), 6.71 (dd, *J* = 7.8, 7.8 Hz, 1H), 6.05 (br s, 2H).

^{13}C NMR (125 MHz, $CDCl_3$, 23 ºC, δ): 144.8, 135.8, 132.3, 126.2, 118.9, 117.0.

HRMS-FIA(m/z) calc'd for $C_6H_7N_2O_2$ $[M+H]^+$, 139.0502; found, 139.0505.

4-Nitroaniline (**2c**):

R*f* = 0.36 (ethyl acetate/hexanes, 40:60 (v/v)).

NMR Spectroscopy:

^{1}H NMR (500 MHz, $CDCl_3$, 23 ºC, δ): 8.06 (d, *J* = 9.1 Hz, 2H), 6.62 (d, *J* = 9.1 Hz, 2H), 4.38 (br s, 2H).

^{13}C NMR (125 MHz, $CDCl_3$, 23 ºC, δ): 152.6, 139.3, 126.5, 113.5.

HRMS-FIA(m/z) calc'd for $C_6H_7N_2O_2$ $[M+H]^+$, 139.0502; found, 139.0500.

3-(Methylsulfonyl)aniline (3a), 2-(methylsulfonyl)aniline (3b), and 4-(methylsulfonyl)aniline (3c)

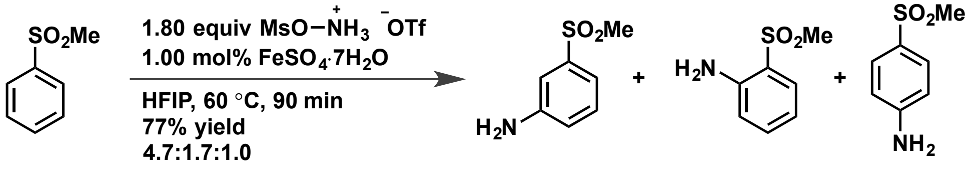

3a 3b 3c

Reagent **1** (141 mg, 0.540 mmol, 1.80 equiv), $FeSO_4 \cdot 7H_2O$ (0.8 mg, 3 µmol, 0.01 equiv), and phenyl methylsulfone (46.9 mg, 0.300 mmol, 1.00 equiv) were added to a 4-mL vial, followed by HFIP (1.5 mL, c = 0.2 M). The reaction mixture was stirred at 60 °C for 90 min. The orange reaction mixture was allowed to cool to room temperature and was concentrated. The residue was dissolved in 10 mL ethyl acetate and poured into a separatory funnel containing 10 mL sat. aq. Na_2CO_3. The layers were separated, and the aqueous layer was extracted with ethyl acetate (2 × 10 mL). The combined organic layers were dried over sodium sulfate, filtered and concentrated. A ^{1}H NMR spectrum of the residue indicated a ratio of **3a**:**3b**:**3c** = 4.7:1.7:1.0 by integrating the signal of **3a** at 6.87 ppm, the signal of **3b** at 6.76 ppm, and the signal of **3c** at 6.68 ppm. The residue was purified by column chromatography on silica gel eluting with a gradient solvent system, starting with a solvent mixture of diethyl ether/pentane (50:50 (v/v)) and finishing with diethyl ether. Purification afforded 2-(methylsulfonyl)aniline (**3b**) in one fraction (10.5 mg) and 3-(methylsulfonyl)aniline (**3a**) and 4-(methylsulfonyl)aniline (**3c**) in a second fraction (29.2 mg) for a combined yield of 39.7 mg (77% yield). The second fraction was further purified by preparative TLC using a solvent system of ethyl acetate/pentane (30:70 (v/v)) to give **3a** (14.6 mg) and **3c** (3.6 mg) as separate samples. Characterization data matched previously reported data for **3b**[13a] and **3c**[13b].

3-(Methylsulfonyl)aniline (**3a**)

R*f* = 0.17 (ethyl acetate/hexanes, 40:60 (v/v)).

NMR Spectroscopy:

^{1}H NMR (600 MHz, $CDCl_3$, 23 ºC, δ): 7.31 (dd, *J* = 7.9, 7.9 Hz, 1H), 7.26 (ddd, *J* = 7.8, 1.4, 1.4 Hz, 1H),

7.20 (dd, J = 2.0, 2.0 Hz, 1H), 6.89 (ddd, J = 8.0, 2.4, 1.0 Hz, 1H), 4.01 (br s, 2H), 3.02 (s, 3H).

^{13}C NMR (125 MHz, $CDCl_3$, 23 ºC, δ): 147.6, 141.5, 130.4, 119.8, 116.8, 112.9, 44.5.

HRMS-FIA(m/z) calc'd for $C_7H_{13}N_2O_2S$ $[M+NH_4]^+$, 172.0427; found, 172.0418.

2-(Methylsulfonyl)aniline (**3b**):

R*f* = 0.57 (diethyl ether/pentanes, 80:20 (v/v)).

NMR Spectroscopy:

^{1}H NMR (500 MHz, $CDCl_3$, 23 ºC, δ): 7.74 (d, J = 8.0 Hz, 1H), 7.37 (dd, J = 7.7, 7.7 Hz, 1H), 6.83 (dd, J = 7.6, 7.6 Hz, 1H), 6.76, (d, J = 8.2 Hz, 1H), 5.00 (br s, 2H), 3.06 (s, 3H).

^{13}C NMR (125 MHz, $CDCl_3$, 23 ºC, δ): 146.3, 135.3, 129.6, 122.2, 118.2, 117.7, 42.4.

HRMS-FIA(m/z) calc'd for $C_7H_{10}NO_2S$ $[M+H]^+$, 172.0427; found, 172.0422.

4-(Methylsulfonyl)aniline (**3c**):

R*f* = 0.13 (ethyl acetate/hexanes, 40:60 (v/v)).

NMR Spectroscopy:

^{1}H NMR (600 MHz, $CDCl_3$, 23 ºC, δ): 7.69 (d, J = 8.8 Hz, 2H), 6.71 (d, J = 8.8 Hz, 2H), 4.20 (br s, 2H), 3.00 (s, 3H).

^{13}C NMR (125 MHz, $CDCl_3$, 23 ºC, δ): 151.4, 129.6, 129.1, 114.2, 45.1.

HRMS-FIA(m/z) calc'd for $C_7H_{13}N_2O_2S$ $[M+NH_4]^+$, 172.0427; found, 172.0422.

3-Aminobenzonitrile (4a), 4-aminobenzonitrile (4b), and 2-aminobenzonitrile (4c)

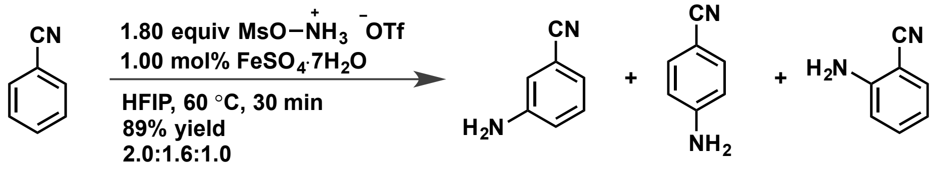

Reagent **1** (141 mg, 0.540 mmol, 1.80 equiv) and $FeSO_4{\cdot}7H_2O$ (0.8 mg, 3 μmol, 0.01 equiv) were added to a 4-mL vial, followed by HFIP (1.5 mL, c = 0.2 M) and benzonitrile (31.0 mg, 31.0 μL, 0.300 mmol, 1.00 equiv). The reaction mixture was stirred at 60 °C for 30 min. The red reaction mixture was allowed to cool to room temperature and was concentrated. The residue was dissolved in 10 mL ethyl acetate and poured into a separatory funnel containing 10 mL sat. aq. Na_2CO_3. The layers were separated, and the aqueous layer was extracted with ethyl acetate (2 × 10 mL). The combined organic layers were dried over sodium sulfate, filtered and concentrated. A ^{1}H NMR spectrum of the residue indicated a ratio of **4a**:**4b**:**4c** = 2.0:1.6:1.0 by integrating the signal of **4a** at 7.00 ppm, the signal of **4b** at 6.64 ppm, and the signal of **4c** at 6.76 ppm. The residue was purified by column chromatography on silica gel eluting with a gradient solvent system, starting with a solvent

mixture of diethyl ether/pentane (20:80 (v/v)) and finishing with diethyl ether/pentane (50:50 (v/v)). Purification afforded 2-aminobenzonitrile (**4c**) in one fraction (6.4 mg) and 3-aminobenzonitrile (**4a**) and 4-aminobenzonitrile (**4b**) in a second fraction (25.2 mg) for a combined yield of 31.6 mg (89% yield). The second fraction was further purified by preparative TLC using a solvent system of acetone/pentane (10:90 (v/v)) to give **4a** (10.0 mg) and **4b** (9.1 mg) as separate samples. Characterization data matched previously reported data for **4a**, **4b**, and **4c**.[14]

3-Aminobenzonitrile (**4a**):

R*f* = 0.50 (ethyl acetate/hexanes, 40:60 (v/v)).

NMR Spectroscopy:

^{1}H NMR (500 MHz, $CDCl_3$, 23 ºC, δ): 7.22 (dd, *J* = 7.9, 7.9 Hz, 1H), 7.01 (ddd, *J* = 7.6, 1.4, 1.0 Hz, 1H), 6.90 (dd, *J* = 1.7, 1.7, 1H), 6.86 (ddd, *J* = 8.2, 2.4, 1.0 Hz, 1H), 3.87 (br s, 2H).

^{13}C NMR (125 MHz, $CDCl_3$, 23 ºC, δ): 147.0, 130.2, 122.1, 119.3, 119.3, 117.6, 113.1.

HRMS-FIA(m/z) calc'd for $C_7H_7N_2$ $[M+H]^+$, 119.0604; found, 119.0608.

4-Aminobenzonitrile (**4b**):

R*f* = 0.38 (ethyl acetate/hexanes, 40:60 (v/v)).

NMR Spectroscopy:

^{1}H NMR (500 MHz, $CDCl_3$, 23 ºC, δ): 7.41 (d, *J* = 8.8 Hz, 2H), 6.64 (d, *J* = 8.8 Hz, 2H), 4.14 (br s, 2H).

^{13}C NMR (125 MHz, $CDCl_3$, 23 ºC, δ): 150.5, 134.0, 120.2, 114.6, 100.5.

HRMS-FIA(m/z) calc'd for $C_7H_7N_2$ $[M+H]^+$, 119.0604; found, 119.0601.

2-Aminobenzonitrile (**4c**):

R*f* = 0.38 (diethyl ether/pentanes, 40:60 (v/v)).

NMR Spectroscopy:

^{1}H NMR (600 MHz, $CDCl_3$, 23 ºC, δ): 7.39 (dd, *J* = 8.0, 1.5 Hz, 1H), 7.33 (dd, *J* = 7.9, 7.9 Hz, 1H), 6.76–6.72 (m, 2H), 4.39 (br s, 2H).

^{13}C NMR (125 MHz, $CDCl_3$, 23 ºC, δ): 149.7, 134.1, 132.5, 118.2, 117.7, 115.3, 96.3.

HRMS-FIA(m/z) calc'd for $C_7H_7N_2$ $[M+H]^+$, 119.0604; found, 119.0604.

2,5-Dibromoaniline (5)

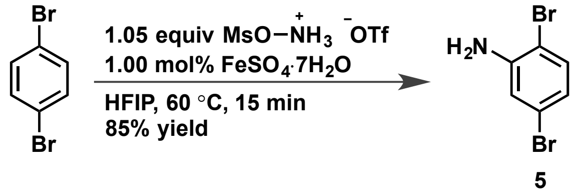

Reagent **1** (82.3 mg, 0.315 mmol, 1.05 equiv), $FeSO_4{\cdot}7H_2O$ (0.8 mg, 3 µmol, 0.01 equiv), and 1,4-dibromobenzene (70.8 mg, 0.300 mmol, 1.00 equiv) were added to a 4-mL vial, followed by HFIP (1.5 mL, c = 0.2 M). The reaction mixture was stirred at 60 °C for 15 min. The red reaction mixture was allowed to cool to room temperature and was concentrated. The residue was dissolved in 10 mL ethyl acetate and poured into a separatory funnel containing 10 mL sat. aq. Na_2CO_3. The layers were separated, and the aqueous layer was extracted with ethyl acetate (2 × 10 mL). The combined organic layers were dried over sodium sulfate, filtered and concentrated. The residue was purified by column chromatography on silica gel eluting with a solvent system of ethyl acetate/hexane (5:95 (v/v)). Purification afforded 64.2 mg of the title compound as a light orange solid (85% yield). Characterization data matched a commercial sample.

R*f* = 0.85 (ethyl acetate/hexanes, 40:60 (v/v)).

NMR Spectroscopy:

^{1}H NMR (600 MHz, $CDCl_3$, 23 ºC, δ): 7.25 (d, *J* = 8.5 Hz, 1H), 6.91 (d, *J* = 2.0 Hz, 1H), 6.74 (dd, *J* = 8.5, 2.2 Hz, 1H), 4.23 (br s, 2H).

^{13}C NMR (125 MHz, $CDCl_3$, 23 ºC, δ): 145.4, 133.7, 122.3, 121.8, 118.2, 107.9.

HRMS-FIA(m/z) calc'd for $C_6H_5Br_2N$ $[M+H]^+$, 251.8841; found, 251.8839.

Ethyl 5-aminothiophene-2-carboxylate (6a), ethyl 4-aminothiophene-2-carboxylate (6b), and ethyl 3-aminothiophene-2-carboxylate (6c)

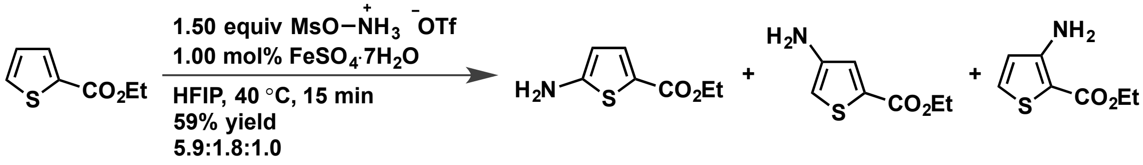

6a 6b 6c

Reagent **1** (118 mg, 0.450 mmol, 1.50 equiv) and $FeSO_4{\cdot}7H_2O$ (0.8 mg, 3 µmol, 0.01 equiv) were added to a 4-mL vial, followed by HFIP (1.5 mL, c = 0.2 M) and ethyl 2-thiophenecarboxylate (46.9 mg, 40.3 µL, 0.300 mmol, 1.00 equiv). The reaction mixture was stirred at 40 °C for 15 min. The red reaction mixture was allowed to cool to room temperature and was concentrated. The residue was dissolved in 10 mL ethyl acetate and poured into a separatory funnel containing 10 mL sat. aq. Na_2CO_3. The layers were separated, and the aqueous layer was extracted with ethyl acetate (2 × 10 mL). The combined organic layers were dried over sodium sulfate, filtered and concentrated. A ^{1}H NMR spectrum of the residue indicated a ratio of **6a**:**6b**:**6c** = 5.9:1.8:1.0 by integrating the signal of **6a** at 6.09 ppm, the signal of **6b** at 6.39 ppm, and the signal of **6c** at 6.55 ppm. The residue was purified by column chromatography on silica gel eluting with a gradient solvent system, starting with a solvent mixture of diethyl ether/pentane (5:95 (v/v)) and finishing with diethyl ether/pentane (50:50 (v/v)).* Purification afforded ethyl 3-aminothiophene-2-carboxylate (**6c**) in one fraction (2.9 mg), ethyl 5-aminothiophene-2-carboxylate (**6a**) in a second fraction (18.0 mg), and ethyl 4-aminothiophene-2-carboxylate (**6b**) in a third fraction (6.6 mg) for a combined yield of 30.5 mg (59% yield).

*Column specifications: diameter = 3 cm, packing height = 7 cm; total amount of silica used: 18 g; total

amount of eluent mixture to collect all products: 610 mL.

Characterization data matched previously reported data for **6a**.[15]

Ethyl 5-aminothiophene-2-carboxylate (**6a**):

R*f* = 0.40 (diethyl ether/pentanes, 50:50 (v/v)).

NMR Spectroscopy:

> **^{1}H NMR** (500 MHz, CD_2Cl_2, 23 ºC, δ): 7.41 (d, *J* = 4.0 Hz, 1H), 6.09 (d, *J* = 4.0 Hz, 1H), 4.45 (br s, 2H), 4.24 (q, *J* = 7.1 Hz, 2H), 1.31 (t, *J* = 7.2 Hz, 3H).

> **^{13}C NMR** (125 MHz, CD_2Cl_2, 23 ºC, δ): 162.8, 159.5, 134.9, 118.2, 107.9, 60.8, 14.6.

HRMS-FIA(m/z) calc'd for $C_1H_{10}NO_2S$ $[M+H]^+$, 172.0427; found, 172.0422.

Ethyl 4-aminothiophene-2-carboxylate (**6b**):

R*f* = 0.24 (diethyl ether/pentanes, 50:50 (v/v)).

NMR Spectroscopy:

> **^{1}H NMR** (500 MHz, CD_2Cl_2, 23 ºC, δ): 7.28 (d, *J* = 1.9 Hz, 1H), 6.39 (d, *J* = 1.8 Hz, 1H), 4.28 (q, *J* = 7.1 Hz, 3H), 3.70 (br s, 2H), 1.33 (t, *J* = 7.1 Hz, 2H).

> **^{13}C NMR** (125 MHz, CD_2Cl_2, 23 ºC, δ): 162.4, 146.3, 133.3, 126.1, 107.4, 61.4, 14.5.

HRMS-FIA(m/z) calc'd for $C_1H_{10}NO_2S$ $[M+H]^+$, 172.0427; found, 172.0422.

Ethyl 3-aminothiophene-2-carboxylate (**6c**):

R*f* = 0.56 (diethyl ether/pentanes, 50:50 (v/v)).

NMR Spectroscopy:

> **^{1}H NMR** (500 MHz, CD_2Cl_2, 23 ºC, δ): 7.28 (d, *J* = 5.4 Hz, 1H), 6.55 (d, *J* = 5.4 Hz, 1H), 5.45 (br s, 2H), 4.26 (q, *J* = 7.1 Hz, 2H), 1.32 (t, *J* = 7.2 Hz, 3H).

> **^{13}C NMR** (125 MHz, CD_2Cl_2, 23 ºC, δ): 164.9, 154.3, 131.6, 120.3, 101.7, 60.4, 14.7.

HRMS-FIA(m/z) calc'd for $C_1H_{10}NO_2S$ $[M+H]^+$, 172.0427; found, 172.0421.

5-Amino-6-methoxy-2-methylquinoline (7a) and 8-amino-6-methoxy-2-methylquinoline (7b)

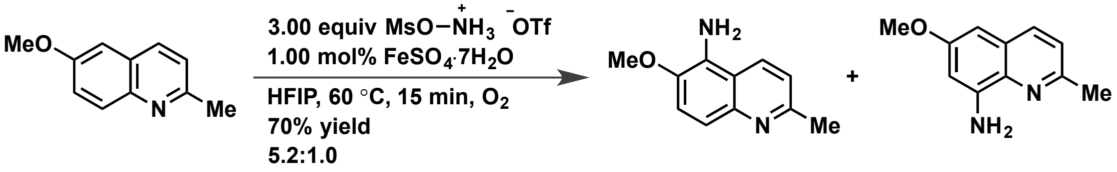

7a **7b**

Reagent **1** (235 mg, 0.900 mmol, 3.00 equiv), $FeSO_4{\cdot}7H_2O$ (0.8 mg, 3 µmol, 0.01 equiv), and 6-methoxy-2-methylquinoline (52.0 mg, 0.300 mmol, 1.00 equiv) were added to a 4-mL vial, followed by HFIP (1.5 mL, c =

0.2 M). The reaction mixture was stirred at 60 °C for 15 min under an atmosphere of oxygen. The dark green reaction mixture was allowed to cool to room temperature and was concentrated. The residue was dissolved in 10 mL ethyl acetate and poured into a separatory funnel containing 10 mL sat. aq. Na_2CO_3. The layers were separated, and the aqueous layer was extracted with ethyl acetate (2 × 10 mL). The combined organic layers were dried over sodium sulfate, filtered and concentrated. A ^{1}H NMR spectrum of the residue indicated a ratio of **7a**:**7b** = 5.2:1.0 by integrating the signal of **7a** at 8.01 ppm and the signal of **7b** at 7.84 ppm. The residue was purified by column chromatography on silica gel eluting with a gradient solvent system, starting with a solvent mixture of diethyl ether/pentane (10:90 (v/v)) and finishing with diethyl ether. Purification afforded 5-amino-6-methoxy-2-methylquinoline (**7a**) in one fraction (13.5 mg) and 8-amino-6-methoxy-2-methylquinoline (**7b**) in a second fraction (26.2 mg) for a combined yield of 39.7 mg (70% yield).

5-Amino-6-methoxy-2-methylquinoline (**7a**):

R*f* = 0.14 (ethyl acetate/hexanes, 50:50 (v/v)).

NMR Spectroscopy:

^{1}H NMR (500 MHz, $CDCl_3$, 23 ºC, δ): 8.01 (d, *J* = 9.0 Hz, 1H), 7.50 (d, *J* = 9.1 Hz, 1H), 7.38 (d, *J* = 9.1 Hz, 1H), 7.17 (d, *J* = 8.7 Hz, 1H), 4.23 (br s, 2H), 3.95 (s, 3H), 2.69 (s, 3H).

^{13}C NMR (125 MHz, $(CD_3)_2CO$, 23 ºC, δ): 156.7, 145.0, 142.2, 132.1, 130.7, 120.5, 118.0, 117.3, 117.3, 57.0, 25.0.

HRMS-FIA(m/z) calc'd for $C_{11}H_{13}N_2O$ $[M+H]^+$, 189.1022; found, 189.1014.

8-Amino-6-methoxy-2-methylquinoline (**7b**):

R*f* = 0.61 (ethyl acetate/hexanes, 50:50 (v/v)).

NMR Spectroscopy:

^{1}H NMR (600 MHz, $CDCl_3$, 23 ºC, δ): 7.84 (d, *J* = 8.3 Hz, 1H), 7.20 (d, *J* = 8.3 Hz, 1H), 6.55 (d, *J* = 2.6 Hz, 1H), 6.45 (d, *J* = 2.6 Hz, 1H), 5.00 (br s, 2H), 3.86 (s, 3H), 2.66 (s, 3H).

^{13}C NMR (125 MHz, $CDCl_3$, 23 ºC, δ): 158.2, 153.7, 144.6, 135.1, 134.9, 127.8, 122.7, 101.6, 94.8, 55.4, 25.0.

HRMS-FIA(m/z) calc'd for $C_{11}H_{13}N_2O$ $[M+H]^+$, 189.1022; found, 189.1022.

3-Amino-4-methoxybenzenesulfonamide (8)

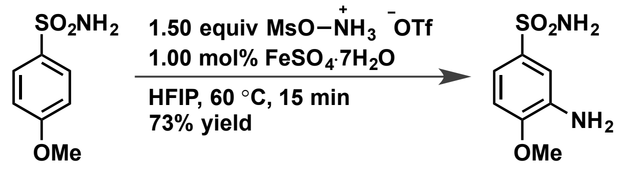

8

Reagent **1** (118 mg, 0.450 mmol, 1.50 equiv), $FeSO_4·7H_2O$ (0.8 mg, 3 μmol, 0.01 equiv), and 4-

methoxybenzenesulfonamide (56.2 mg, 0.300 mmol, 1.00 equiv) were added to a 4-mL vial, followed by HFIP (1.5 mL, c = 0.2 M). The reaction mixture was stirred at 60 °C for 15 min. The dark green reaction mixture was allowed to cool to room temperature and was concentrated. The residue was dissolved in 10 mL ethyl acetate and poured into a separatory funnel containing 10 mL sat. aq. Na_2CO_3. The layers were separated, and the aqueous layer was extracted with ethyl acetate (2 × 10 mL). The combined organic layers were dried over sodium sulfate, filtered and concentrated. The residue was purified by column chromatography on silica gel eluting with a gradient solvent system, starting with a solvent mixture of diethyl ether/pentane (70:30 (v/v)) and finishing with diethyl ether. Purification afforded 44.4 mg of the title compound (73% yield).

R*f* = 0.15 (ethyl acetate/hexanes, 50:50 (v/v)).

NMR Spectroscopy:

^{1}H NMR (500 MHz, CD_3OD, 23 ºC, δ): 7.24–7.20 (m, 2H), 6.91 (d, *J* = 8.1 Hz, 1H), 3.90 (s, 3H).

^{13}C NMR (125 MHz, CD_3OD, 23 ºC, δ): 151.5, 138.7, 136.7, 117.3, 112.8, 110.6, 56.3.

HRMS-FIA(m/z) calc'd for $C_7H_{11}N_2O_3S$ $[M+H]^+$, 203.0485; found, 203.0477.

Methyl 4-amino-1*H*-benzimidazole-6-carboxylate (9a), methyl 7-amino-1*H*-benzimidazole-6-carboxylate (9b) and methyl 5-amino-1*H*-benzimidazole-6-carboxylate (9c)

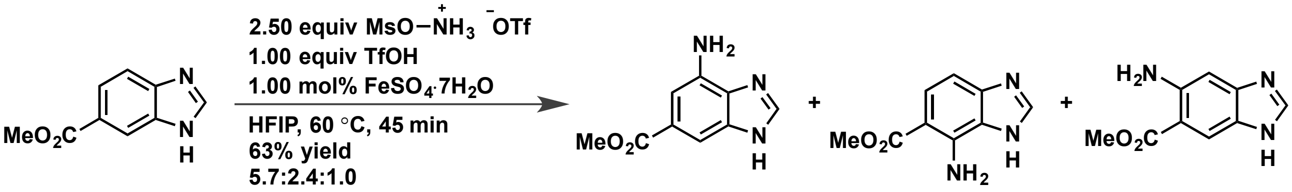

9a **9b** **9c**

$FeSO_4 \cdot 7H_2O$ (0.8 mg, 3 µmol, 0.01 equiv) and methyl 1*H*-benzimidazole-6-carboxylate (52.9 mg, 0.300 mmol, 1.00 equiv) were added to a 4-mL vial, followed by HFIP (1.5 mL, c = 0.2 M) and TfOH (26.5 µL, 0.300 mmol, 1.00 equiv). Reagent **1** (196 mg, 0.750 mmol, 2.50 equiv) was then added to the vial. The reaction mixture was stirred at 60 °C for 45 min. The brown reaction mixture was allowed to cool to room temperature and was concentrated. The residue was dissolved in 10 mL ethyl acetate and poured into a separatory funnel containing 10 mL sat. aq. Na_2CO_3. The layers were separated, and the aqueous layer was extracted with ethyl acetate (2 × 10 mL). The combined organic layers were dried over sodium sulfate, filtered and concentrated. A ^{1}H NMR spectrum of the residue indicated a ratio of **9a**:**9b**:**9c** = 5.7:2.4:1.0 by integrating the signal of **9a** at 7.22 ppm, the signal of **9b** at 6.76 ppm, and the signal of **9c** at 6.85 ppm. The residue was purified by column chromatography on basified silica gel (NH_4OH) eluting with a gradient solvent system, starting with a solvent mixture of methanol/dichloromethane (2:98 (v/v)) and finishing with methanol/dichloromethane (5:95 (v/v)). Purification afforded methyl 7-amino-1*H*-benzimidazole-6-carboxylate (**9b**) and a small amount of unreacted starting material in one fraction and methyl 4-amino-1*H*-benzimidazole-6-carboxylate (**9a**) and methyl 5-amino-1*H*-benzimidazole-6-carboxylate (**9c**) in a second fraction (31.7 mg). The first fraction was further purified by preparative TLC using a solvent system of methanol/dichloromethane

(2:98 (v/v)) to give **9b** (4.3 mg). The second fraction was characterized as a mixture. A combined yield of 36.0 mg (63% yield) was obtained. Characterization data matched previously reported data for **9c**.[16]

Methyl 4-amino-1*H*-benzimidazole-6-carboxylate (**9a**) and methyl 5-amino-1*H*-benzimidazole-6-carboxylate (**9c**):

R*f* = 0.19 (methanol/dichloromethane, 5:95 (v/v)).

NMR Spectroscopy:

^{1}**H NMR** (500 MHz, CD_3OD, 23 ºC, δ): 8.16 (s, 1H), 8.15* (s, 1H), 8.03* (s, 1H), 7.61 (s, 1H), 7.22 (s, 1H), 6.85* (s, 1H), 3.87 (s, 3H), 3.87* (s, 3H).

^{13}**C NMR** (125 MHz, CD_3OD/CD_2Cl_2, 23 ºC, δ): 170.1*, 169.7, 148.8*, 144.1*, 143.0*, 143.0, 138.6, 136.4, 135.2*, 134.0, 126.8, 121.0*, 109.9*, 108.1, 106.2, 99.2*, 52.6, 52.1*.

*Denotes signals of minor isomer (**9c**).

HRMS-FIA(m/z) calc'd for $C_9H_{10}N_3O_2$ $[M+H]^+$, 192.0768; found, 192.0762.

Methyl 7-amino-1*H*-benzimidazole-6-carboxylate (**9b**):

R*f* = 0.54 (methanol/dichloromethane, 5:95 (v/v)).

NMR Spectroscopy:

^{1}**H NMR** (500 MHz, CD_3OD, 23 ºC, δ): 8.05 (s, 1H), 7.73 (d, *J* = 8.8 Hz, 1H), 6.76 (d, *J* = 8.3 Hz, 1H), 3.86 (s, 3H).

^{13}**C NMR** (125 MHz, CD_3OD, 23 ºC, δ): 170.6, 145.1, 141.0, 138.5, 131.2, 127.3, 103.7, 101.6, 51.7.

HRMS-FIA(m/z) calc'd for $C_9H_{10}N_3O_2$ $[M+H]^+$, 192.0768; found, 192.0760.

3-Amino-2,6-dichlorobenzonitrile (10a) and 4-amino-2,6-dichlorobenzonitrile (10b)

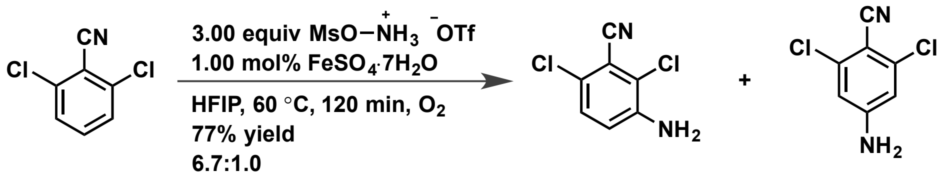

10a **10b**

Reagent **1** (235 mg, 0.900 mmol, 3.00 equiv), $FeSO_4{\cdot}7H_2O$ (0.8 mg, 3 µmol, 0.01 equiv), and 2,6-dichlorobenzonitrile (51.6 mg, 0.300 mmol, 1.00 equiv) were added to a 4-mL vial, followed by HFIP (1.5 mL, c = 0.2 M). The reaction mixture was stirred at 60 °C for 120 min under an atmosphere of oxygen. The orange reaction mixture was allowed to cool to room temperature and was concentrated. The residue was dissolved in 10 mL ethyl acetate and poured into a separatory funnel containing 10 mL sat. aq. Na_2CO_3. The layers were separated, and the aqueous layer was extracted with ethyl acetate (2 × 10 mL). The combined organic layers were dried over sodium sulfate, filtered and concentrated. A ^{1}H NMR spectrum of the residue indicated a ratio of **10a**:**10b** = 6.7:1.0 by integrating the signal of **10a** at 6.86 ppm and the signal of **10b** at

6.68 ppm. The residue was purified by column chromatography on silica gel eluting with a solvent system of ethyl acetate/pentane (20:80 (v/v)). Purification afforded 3-amino-2,6-dichlorobenzonitrile (**10a**) and 4-amino-2,6-dichlorobenzonitrile (**10b**) in one fraction for a combined yield of 43.4 mg (77% yield). The fraction was further purified by preparative TLC using a solvent system of dichloromethane/pentane (50:50 (v/v)) to give **10a** (32.4 mg) and **10b** (3.9 mg).

3-Amino-2,6-dichlorobenzonitrile (**10a**):

R*f* = 0.50 (ethyl acetate/hexanes, 40:60 (v/v)).

NMR Spectroscopy:

^{1}H NMR (500 MHz, $CDCl_3/CD_3OD$, 23 ºC, δ): 6.86 (d, *J* = 8.9 Hz, 1H), 6.67 (d, *J* = 8.9 Hz, 1H).

^{13}C NMR (125 MHz, $CDCl_3/CD_3OD$, 23 ºC, δ): 143.7, 128.1, 124.1, 119.9, 119.6, 113.6, 112.8.

HRMS-FIA(m/z) calc'd for $C_7H_5Cl_2N_2$ $[M+H]^+$, 186.9824; found, 186.9818.

4-Amino-2,6-dichlorobenzonitrile (**10b**):

R*f* = 0.46 (ethyl acetate/hexanes, 40:60 (v/v)).

NMR Spectroscopy:

^{1}H NMR (500 MHz, CD_3OD, 23 ºC, δ): 6.68 (s, 2H).

^{13}C NMR (125 MHz, CD_3OD, 23 ºC, δ): 156.6, 139.7, 116.0, 113.5, 99.2.

HRMS-FIA(m/z) calc'd for $C_7H_5Cl_2N_2$ $[M+H]^+$, 186.9824; found, 186.9828.

3-Aminomoclebomide (11a) and 2-Aminomoclebomide (11b)

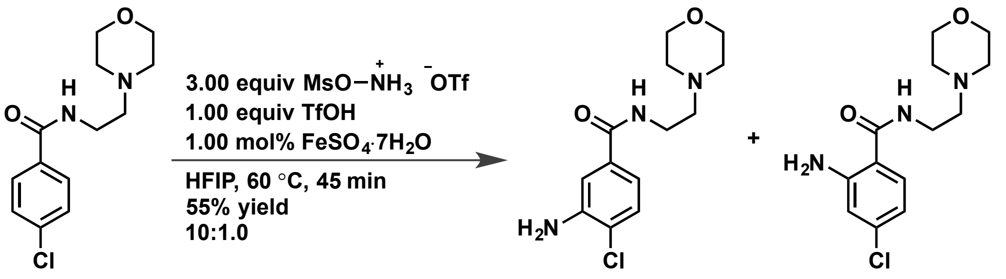

$FeSO_4{\cdot}7H_2O$ (0.8 mg, 3 µmol, 0.01 equiv) and moclebomide (**S2**) (80.6 mg, 0.300 mmol, 1.00 equiv) were added to a 4-mL vial, followed by HFIP (1.5 mL, c = 0.2 M) and TfOH (26.5 µL, 0.300 mmol, 1.00 equiv). Reagent **1** (235 mg, 0.900 mmol, 3.00 equiv) was then added to the vial. The reaction mixture was stirred at 60 °C for 45 min. The red brown reaction mixture was allowed to cool to room temperature and was concentrated. The residue was dissolved in 10 mL ethyl acetate and poured into a separatory funnel containing 10 mL sat. aq. Na_2CO_3. The layers were separated, and the aqueous layer was extracted with ethyl acetate (2 × 10 mL). The combined organic layers were dried over sodium sulfate, filtered and concentrated. A ^{1}H NMR spectrum of the residue indicated a ratio of **11a**:**11b** = 10:1.0 by integrating the

signal of **11a** at 7.25 ppm and the signal of **11b** at 6.63 ppm. The residue was purified by column chromatography on basified silica gel (NH_4OH) eluting with a gradient solvent system, starting with a solvent mixture of methanol/dichloromethane (1:99 (v/v)) and finishing with methanol/dichloromethane (5:95 (v/v)). Purification afforded 2-aminomoclebomide (**11b**) in one fraction and 3-aminomoclebomide (**11a**) in a second fraction (42.6 mg). The first fraction was further purified by preparative TLC using a solvent system of methanol/dichloromethane (2:98 (v/v)) to give **11b** (3.8 mg). A combined yield of 46.4 mg (55% yield) was obtained.

3-Aminomoclebomide (**11a**):

R*f* = 0.18 (methanol/dichloromethane, 5:95 (v/v)).

NMR Spectroscopy:

^{1}H NMR (500 MHz, $CDCl_3$, 23 ºC, δ): 7.28 (d, *J* = 8.2 Hz, 1H), 7.25 (d, *J* = 2.0 Hz, 1H), 6.97 (dd, *J* = 8.2, 2.1 Hz, 1H), 6.69 (br s, 1H), 4.21 (br s, 2H), 3.72 (t, *J* = 4.6 Hz, 4H), 3.51 (dt, *J* = 5.7, 5.7 Hz, 2H), 2.58 (t, *J* = 6.1 Hz, 2H), 2.49 (br s, 4H).

^{13}C NMR (125 MHz, $CDCl_3$, 23 ºC, δ): 166.9, 143.4, 134.4, 129.5, 122.2, 116.5, 114.8, 67.1, 57.0, 53.5, 36.2.

HRMS-FIA(m/z) calc'd for $C_{13}H_{19}ClN_3O_2$ $[M+H]^+$, 284.1160; found, 284.1163.

2-Aminomoclebomide (**11b**):

R*f* = 0.25 (methanol/dichloromethane, 5:95 (v/v)).

NMR Spectroscopy:

^{1}H NMR (500 MHz, $CDCl_3$, 23 ºC, δ): 7.25 (d, *J* = 7.7 Hz, 1H), 6.67 (d, *J* = 1.9 Hz, 1H), 6.63 (dd, *J* = 8.4, 2.0 Hz, 1H), 5.67 (br s, 1H), 3.74 (br s, 4H), 3.51 (dt, *J* = 5.7, 5.7 Hz, 2H), 2.61 (t, *J* = 5.7 Hz, 2H), 2.52 (br s, 4H).

^{13}C NMR (125 MHz, $CDCl_3$, 23 ºC, δ): 168.7, 150.0, 138.2, 128.6, 116.9, 116.7, 114.5, 67.0, 57.0, 53.5, 35.8.

HRMS-FIA(m/z) calc'd for $C_{13}H_{19}ClN_3O_2$ $[M+H]^+$, 284.1160; found, 284.1158.

2-Amino-4-(trifluoromethyl)phenyl 2-nitrobenzenesulfonate (12a) and 3-amino-4-(trifluoromethyl)phenyl 2-nitrobenzenesulfonate (12b)

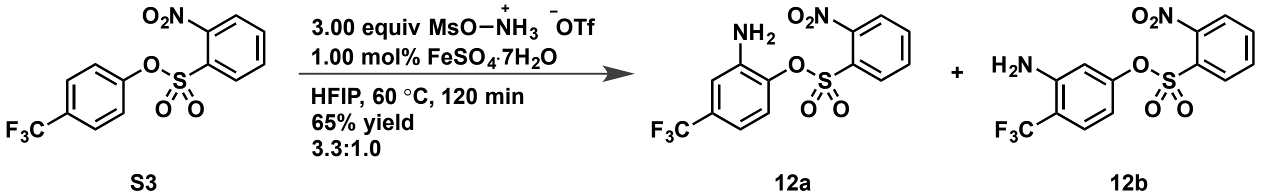

S3 12a 12b

Reagent **1** (235 mg, 0.900 mmol, 3.00 equiv), $FeSO_4·7H_2O$ (0.8 mg, 3 µmol, 0.01 equiv), and 4-(trifluoromethyl)phenyl 2-nitrobenzenesulfonate (**S3**) (104 mg, 0.300 mmol, 1.00 equiv) were added to a 4-mL

vial, followed by HFIP (1.5 mL, c = 0.2 M). The reaction mixture was stirred at 60 °C for 120 min. The orange reaction mixture was allowed to cool to room temperature and was concentrated. The residue was dissolved in 10 mL ethyl acetate and poured into a separatory funnel containing 10 mL sat. aq. Na_2CO_3. The layers were separated, and the aqueous layer was extracted with ethyl acetate (2 × 10 mL). The combined organic layers were dried over sodium sulfate, filtered and concentrated. A ^{1}H NMR spectrum of the residue indicated a ratio of **12a**:**12b** = 3.3:1.0 by integrating the signal of **12a** at 6.91 ppm and the signal of **12b** at 6.58 ppm. The residue was purified by column chromatography on silica gel eluting with a gradient solvent system, starting with a solvent mixture of diethyl ether/pentane (20:80 (v/v)) and finishing with diethyl ether. Purification afforded 2-amino-4-(trifluoromethyl)phenyl 2-nitrobenzenesulfonate (**12a**) and 3-amino-4-(trifluoromethyl)phenyl 2-nitrobenzenesulfonate (**12b**) in one fraction for a combined yield of 71.0 mg (65% yield). The fraction was further purified by preparative TLC using a solvent system of acetone/pentane (10:90 (v/v)) to give **12a** (47.3 mg) and **12b** (11.1 mg) as separate samples.

2-Amino-4-(trifluoromethyl)phenyl 2-nitrobenzenesulfonate (**12a**):

R*f* = 0.14 (ethyl acetate/hexanes, 20:80 (v/v)).

NMR Spectroscopy:

^{1}H NMR (500 MHz, $CDCl_3$, 23 ºC, δ): 7.93 (d, *J* = 8.0 Hz, 1H), 7.89–7.83 (m, 2H), 7.71 (ddd, *J* = 7.9, 6.4, 2.5 Hz, 1H), 7.34 (d, *J* = 8.5 Hz, 1H), 6.93 (dd, *J* = 8.6, 1.8 Hz, 1H), 6.91 (d, *J* = 1.9 Hz, 1H), 4.27 (br s, 2H).

^{13}C NMR (125 MHz, $CDCl_3$, 23 ºC, δ): 148.7, 140.2, 137.9, 136.0, 132.5, 132.3, 130.7 (q, *J* = 32.6 Hz), 128.5, 125.2, 124.1, 123.7 (q, *J* = 270.9 Hz), 114.9 (q, *J* = 3.8 Hz), 113.7 (q, *J* = 3.8 Hz).

^{19}F NMR (470 MHz, $CDCl_3$, 23 ºC, δ): –63.2.

HRMS-FIA(m/z) calc'd for $C_{13}H_{10}F_3N_2O_5S$ $[M+H]^+$, 363.0257; found, 363.0246.

3-Amino-4-(trifluoromethyl)phenyl 2-nitrobenzenesulfonate (**12b**):

R*f* = 0.10 (ethyl acetate/hexanes, 20:80 (v/v)).

NMR Spectroscopy:

^{1}H NMR (500 MHz, $CDCl_3$, 23 ºC, δ): 8.02 (d, *J* = 8.0 Hz, 1H), 7.88–7.82 (m, 2H), 7.72 (ddd, *J* = 8.0, 6.0, 2.7 Hz, 1H), 7.37 (d, *J* = 8.7 Hz, 1H), 6.65 (d, *J* = 1.9 Hz, 1H), 6.58 (ddd, *J* = 8.7, 1.5, 0.8 Hz, 1H), 4.32 (br s, 2H).

^{13}C NMR (125 MHz, $CDCl_3$, 23 ºC, δ): 152.3, 148.8, 146.4, 135.7, 132.3, 132.3, 128.6 (q, *J* = 5.2), 128.5, 125.1, 124.5 (q, *J* = 272 Hz), 113.0 (q, *J* = 30.7 Hz), 110.8, 110.3.

^{19}F NMR (470 MHz, $CDCl_3$, 23 ºC, δ): –63.2.

HRMS-FIA(m/z) calc'd for $C_{13}H_{10}F_3N_2O_5S$ $[M+H]^+$, 363.0257; found, 363.0528.

3-Aminorufinamide (13a) and 4-aminorufinamide (13b)

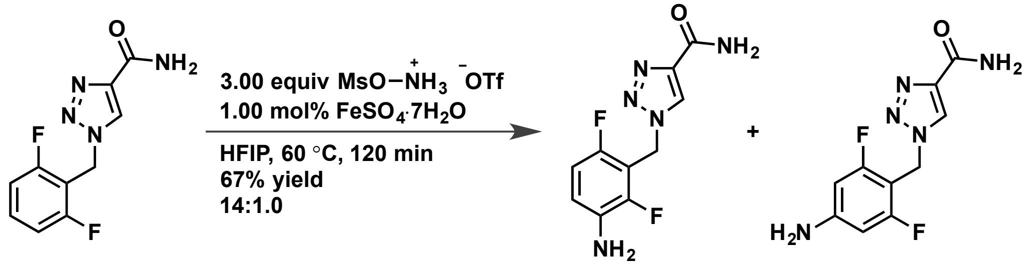

13a 13b

Reagent **1** (235 mg, 0.900 mmol, 3.00 equiv), $FeSO_4{\cdot}7H_2O$ (0.8 mg, 3 µmol, 0.01 equiv), and rufinamide (71.5 mg, 0.300 mmol, 1.00 equiv) were added to a 4-mL vial, followed by HFIP (1.5 mL, c = 0.2 M). The reaction mixture was stirred at 60 °C for 120 min. The red reaction mixture was allowed to cool to room temperature and was concentrated. The residue was dissolved in 10 mL ethyl acetate and poured into a separatory funnel containing 10 mL sat. aq. Na_2CO_3. The layers were separated, and the aqueous layer was extracted with ethyl acetate (2 × 10 mL). The combined organic layers were dried over sodium sulfate, filtered and concentrated. A ^{1}H NMR spectrum of the residue indicated a ratio of **13a**:**13b** = 14:1.0 by integrating the signal of **13a** at 8.47 ppm and the signal of **13b** at 8.37 ppm. The residue was purified by column chromatography on silica gel basified with NH_4OH eluting with a gradient solvent system, starting with a solvent mixture of methanol/dichloromethane (1:99 (v/v)) and finishing with methanol/dichloromethane (2:98 (v/v)). Purification afforded 3-aminorufinamide and 4-aminorufinamide in one fraction for a combined yield of 51.0 mg (67% yield). The two isomers were characterized as a mixture.

R*f* = 0.10 (methanol/dichloromethane, 2:98 (v/v)).

NMR Spectroscopy:

^{1}H NMR (500 MHz, $(CD_3)_2SO$, 23 ºC, δ): 8.47 (s, 1H), 8.37* (s, 1H), 7.85 (s, 1H), 7.46 (s, 1H), 6.90–6.84 (m, 1H), 6.83–6.76 (m, 1H), 6.23* (d, *J* = 10.4, 2H), 5.65 (s, 2H), 5.47* (s, 2H).

^{13}C NMR (125 MHz, $(CD_3)_2SO$, 23 ºC, δ): 161.8* (dd, *J* = 242.4, 11.2 Hz), 161.3, 161.2*, 151.3 (dd, *J* = 235.5, 5.7 Hz), 148.2 (dd, *J* = 242.0, 6.9 Hz), 151.9* (d, *J* = 14.5 Hz), 142.8, 142.7*, 133.1 (d, *J* = 12.8 Hz), 116.3 (dd, *J* = 6.4, 6.4 Hz), 111.0 (dd, *J* = 21.7, 3.5 Hz), 110.5 (dd, *J* = 19.8, 16.4 Hz), 96.5* (m), 96.1–95.8* (m), 41.6 (dd, *J* = 3.7, 3.7 Hz), 41.2* (t, 3.7 Hz).

^{19}F NMR (470 MHz, $CDCl_3$, 23 ºC, δ): –120.6*, –134.5, –141.1.

*Denotes signals of minor isomer (**13b**).

HRMS-FIA(m/z) calc'd for $C_{10}H_{10}F_2N_5O$ $[M+H]^+$, 254.0848; found, 254.0842.

Gram-scale amination reaction

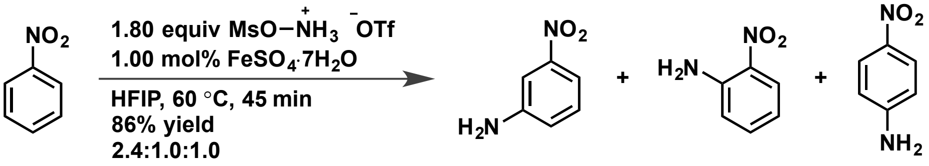

2a 2b 2c

Reagent **1** (7.64 g, 29.2 mmol, 1.80 equiv) and $FeSO_4·7H_2O$ (450. mg, 1.62 mmol, 0.0100 equiv) were added to a round bottom flask equipped with a reflux condenser, followed by HFIP (80 mL, c = 0.2 M) and nitrobenzene (1.99 g, 1.67 mL, 16.2 mmol, 1.00 equiv). The reaction mixture was stirred at 60 °C for 45 min. The orange reaction mixture was allowed to cool to room temperature and was concentrated. The residue was dissolved in 200 mL ethyl acetate and poured into a separatory funnel containing 200 mL sat. aq. Na_2CO_3. The layers were separated, and the aqueous layer was extracted with ethyl acetate (2 × 200 mL). The combined organic layers were dried over sodium sulfate, filtered and concentrated. A ^{1}H NMR spectrum of the residue indicated a ratio of **2a**:**2b**:**2c** = 2.4:1.0:1.0 by integrating the signal of **2a** at 6.94 ppm, the signal of **2b** at 6.76 ppm, and the signal of **2c** at 6.62 ppm. The residue was purified by column chromatography on silica gel eluting with a gradient solvent system, starting with a solvent mixture of diethyl ether/pentane (10:90 (v/v)) and finishing with diethyl ether/pentane (60:40 (v/v)). Purification afforded 2-nitroaniline (**2b**) in one fraction (472 mg), 3-nitroaniline (**2a**) in a second fraction (479 mg) and 4-nitroaniline (**2c**) in a third fraction (139 mg). A fourth fraction was collected that contained both **2a** and **2c**. The fourth fraction was further purified by column chromatography on silica gel eluting with a gradient solvent system, starting with a solvent mixture of diethyl ether/pentane (10:90 (v/v)) and finishing with diethyl ether/pentane (60:40 (v/v)), to afford one fraction containing **2a** (507 mg) and a second fraction containing **2c** (338 mg). A combined yield of 1.94 g (86% yield) was obtained.

Comparison to other amination methods

In comparison to other reported modern amination methods that use an ammoniumyl radical precursor, our method is applicable to a much broader electronic scope of aromatic substrates. The most relevant conditions are compared in Table S1.

Table S1. Comparison of modern amination methods.

conditions	*Ph–OMe*	*Ph–Br*	*Ph–CN*
**this work** [MsO–NH_3]OTf (1.5–1.8 equiv), $FeSO_4·7H_2O$ (0.01 equiv), HFIP, 60 ºC	57%	66%	89%
**ref 9a**	65%	77%	n.r.

[MsO–NH_3]OTf (1.5–4.0 equiv), $FeSO_4·7H_2O$ (0.05 equiv), MeCN/H_2O (degassed), 23 ºC			
**ref 17** HOSA (1.0 equiv), $FeSO_4·7H_2O$ (0.03 equiv), AcOH/H_2O, 40 ºC	60%	30%	5%

Our method also works in the absence of an iron salt, albeit with longer reaction times. When other reported methods are used in the absence of an iron salt, essentially no reaction is observed. Bromobenzene was used to compare reactivity in the absence of iron (Table S2).

Table S2. Comparison of modern amination methods in the absence of iron.

conditions	*NMR yield of* ***S4***
**this work** [MsO–NH_3]OTf (1.5 equiv), HFIP, 60 ºC	83%
**ref 9a** [MsO–NH_3]OTf (4.0 equiv), MeCN/H_2O (degassed), 23 ºC	0%
**ref 17** HOSA (1.0 equiv), AcOH/H_2O, 40 ºC	3%

4-Bromoaniline (S4a), 2-bromoaniline (S4b), and 3-bromoaniline (S4c)

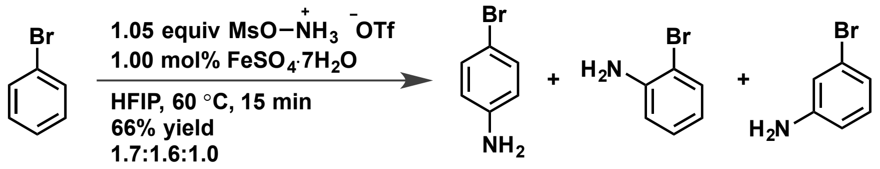

S4a **S4b** **S4c**

Standard procedure (Table S1). Reagent **1** (82.3 mg, 0.315 mmol, 1.05 equiv) and $FeSO_4·7H_2O$ (0.8 mg, 3 μmol, 0.01 equiv) were added to a 4-mL vial, followed by HFIP (1.5 mL, c = 0.2 M) and bromobenzene (47.1 mg, 31.5 μL, 0.300 mmol, 1.00 equiv). The reaction mixture was stirred at 60 °C for 30 min. The purple

reaction mixture was allowed to cool to room temperature and was concentrated. The residue was dissolved in 10 mL ethyl acetate and poured into a separatory funnel containing 10 mL sat. aq. Na_2CO_3. The layers were separated, and the aqueous layer was extracted with ethyl acetate (2 × 10 mL). The combined organic layers were dried over sodium sulfate, filtered and concentrated. A ^1H NMR spectrum of the residue indicated a ratio of **S4a**:**S4b**:**S4c** = 1.7:1.5:1.0 by integrating the signal of **S4a** at 7.40 ppm, the signal of **S4b** at 7.22 ppm, and the signal of **S4c** at 7.00 ppm. The residue was purified by column chromatography on silica gel eluting with a gradient solvent system, starting with a solvent mixture of diethyl ether/pentane (5:95 (v/v)) and finishing with a solvent mixture of diethyl ether/pentane (30:70 (v/v)). Purification afforded 2-bromoaniline (**S4b**), 3-bromoaniline (**S4c**) and 4-bromoaniline (**S4a**) in one fraction for a combined yield of 34.3 mg (66% yield). Further purification by column chromatography eluting with a gradient solvent system, starting with a solvent mixture of diethyl ether/pentane (5:95 (v/v)) and finishing with a solvent mixture of diethyl ether/pentane (30:70 (v/v)) gave **S4b** (10.0 mg), **S4c** (3.7 mg), and **S4a** (12.8 mg) in separate fractions. Characterization data matched previously reported data for **S4a**, **S4b**, and **S4c**.[9a]

This work (Table S2). Reagent **1** (86.2 mg, 0.330 mmol, 1.10 equiv) was added to a flame-dried Schlenk tube, followed by HFIP (1.5 mL, c = 0.2 M) and bromobenzene (47.1 mg, 31.5 µL, 0.300 mmol, 1.00 equiv). The reaction mixture was stirred at 60 °C for 16 h. The brown reaction mixture was allowed to cool to room temperature and was concentrated. The residue was dissolved in 10 mL ethyl acetate and poured into a separatory funnel containing 10 mL sat. aq. Na_2CO_3. An ethyl acetate solution containing 1,3,5-trimethoxybenzene (0.1 mmol) was added as an internal standard, and the layers were separated. The aqueous layer was extracted with ethyl acetate (2 × 10 mL). The combined organic layers were dried over sodium sulfate, filtered and concentrated. A ^1H NMR spectrum of the residue was taken, and yield was determined based on integration against the internal standard to be 83%.

Ref 9a (Table S2). Reagent **1** (313 mg, 1.20 mmol, 4.00 equiv) was added to a flame-dried Schlenk tube, followed by degassed MeCN/H_2O (2:1, 900 µL, c = 0.33 M) and bromobenzene (47.1 mg, 31.5 µL, 0.300 mmol, 1.00 equiv). The reaction mixture was stirred at 23 °C for 16 h. The colorless reaction mixture was diluted with 1.0 M NaOH (aq) (10 ml) and was poured into a separatory funnel. An ethyl acetate solution containing 1,3,5-trimethoxybenzene (0.1 mmol) was added as an internal standard, and the aqueous layer was extracted with dichloromethane (3 × 10 mL). The combined organic layers were dried over sodium sulfate, filtered and concentrated. A ^1H NMR spectrum of the residue was taken, and yield was determined based on integration against the internal standard to be 0%.

Ref 17 (Table S2). Hydroxylamine-*O*-sulfonic acid (33.9 mg, 0.300 mmol, 1.00 equiv) was added to a flame-dried Schlenk tube, followed by AcOH/H_2O (2:1, 500 µL, c = 0.60 M) and bromobenzene (47.1 mg, 31.5 µL, 0.300 mmol, 1.00 equiv). The reaction mixture was stirred at 40 °C for 16 h. The colorless reaction mixture was basified with 1.0 M NaOH (aq) (~10 ml) and was poured into a separatory funnel. An ethyl acetate solution containing 1,3,5-trimethoxybenzene (0.1 mmol) was added as an internal standard, and the aqueous layer was extracted with diethyl ether (3 × 10 mL). The combined organic layers were dried over sodium sulfate, filtered and concentrated. A ^1H NMR spectrum of the residue was taken, and yield was determined

based on integration against the internal standard to be 3%.

4-Bromoaniline (**S4a**):

R*f* = 0.28 (ethyl acetate/hexanes, 20:80 (v/v)).

NMR Spectroscopy:

^{1}H NMR (500 MHz, CD_2Cl_2, 23 °C, δ): 7.22 (d, *J* = 8.8 Hz, 2H), 6.57 (d, *J* = 8.8 Hz, 2H), 3.74 (br s, 2H).

^{13}C NMR (125 MHz, CD_2Cl_2, 23 °C, δ): 146.4, 132.3, 116.9, 109.9.

HRMS-FIA(m/z) calc'd for C_6H_7NBr $[M+H]^+$, 171.9756; found, 171.9751.

2-Bromoaniline (**S4b**):

R*f* = 0.61 (ethyl acetate/hexanes, 20:80 (v/v)).

NMR Spectroscopy:

^{1}H NMR (500 MHz, $CDCl_3$, 23 °C, δ): 7.40 (dd, *J* = 8.0, 1.3 Hz, 1H), 7.10 (dd, *J* = 7.7, 7.7 Hz, 1H), 6.77 (dd, *J* = 8.0, 1.5 Hz, 1H), 6.62 (dd, *J* = 7.0, 7.0 Hz, 1H), 4.09 (br s, 2H).

^{13}C NMR (125 MHz, $CDCl_3$, 23 °C, δ): 144.2, 132.7, 128.5, 119.5, 115.9, 109.5.

HRMS-FIA(m/z) calc'd for C_6H_7NBr $[M+H]^+$, 171.9756; found, 171.9758.

3-Bromoaniline (**S4c**):

R*f* = 0.38 (ethyl acetate/hexanes, 20:80 (v/v)).

NMR Spectroscopy:

^{1}H NMR (500 MHz, CD_2Cl_2, 23 °C, δ): 7.00 (dd, *J* = 8.0, 8.0 Hz, 1H), 6.85–6.80 (m, 2H), 6.62–6.57 (m, 1H), 3.78 (br s, 2H).

^{13}C NMR (125 MHz, CD_2Cl_2, 23 °C, δ): 148.7, 131.0, 123.2, 121.3, 117.8, 113.9.

HRMS-FIA(m/z) calc'd for C_6H_7NBr $[M+H]^+$, 171.9756; found, 171.9754.

4-Methoxyaniline (S5a), 2-methoxyaniline (S5b), and 3-methoxyaniline (S5c)

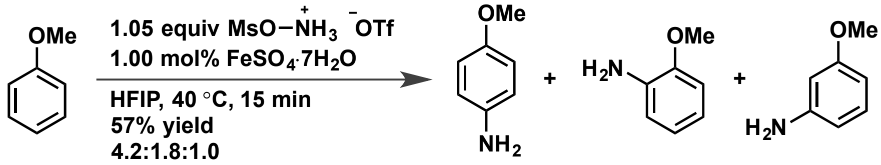

S5a **S5b** **S5c**

Reagent **1** (137 mg, 0.525 mmol, 1.05 equiv) and $FeSO_4·7H_2O$ (1.4 mg, 5.0 μmol, 0.010 equiv) were added to a 4-mL vial, followed by HFIP (1.5 mL, c = 0.2 M) and anisole (54.1 mg, 54.3 μL, 0.500 mmol, 1.00 equiv). The reaction mixture was stirred at 40 °C for 15 min. The blue reaction mixture was allowed to cool to room temperature and was concentrated. The residue was dissolved in 15 mL ethyl acetate and poured into a

separatory funnel containing 15 mL sat. aq. Na_2CO_3. The layers were separated, and the aqueous layer was extracted with ethyl acetate (2 × 15 mL). The combined organic layers were dried over sodium sulfate, filtered and concentrated. A ^{1}H NMR spectrum of the residue indicated a ratio of **S5a**:**S5b**:**S5c** = 4.2:1.8:1.0 by integrating the signal of **S5a** at 6.72 ppm, the signal of **S5b** at 6.79 ppm and the signal of **S5c** at 7.06 ppm. The residue was purified by column chromatography on silica gel eluting with a gradient solvent system, starting with a solvent mixture of diethyl ether/pentane (10:90 (v/v)) and finishing with a solvent mixture of diethyl ether/pentane (40:60 (v/v)). Purification afforded 2-methoxyaniline (**S5b**), 3-methoxyaniline (**S5c**) and 4-methoxyaniline (**S5a**) in one fraction for a combined yield of 35.3 mg (57% yield). Further purification by column chromatography eluting with a gradient solvent system, starting with a solvent mixture of diethyl ether/pentane (10:90 (v/v)) and finishing with a solvent mixture of diethyl ether/pentane (40:60 (v/v)) gave **S5b** (5.9 mg), **S5c** (3.0 mg), and **S5a** (14.9 mg) in separate fractions. Characterization data matched previously reported data for **S5a**, **S5b**, and **S5c**.[9a]

4-Methoxyaniline (**S5a**):

R*f* = 0.14 (ethyl acetate/hexanes, 20:80 (v/v)).

NMR Spectroscopy:

^{1}H NMR (500 MHz, CD_2Cl_2, 23 ºC, δ): 6.72 (d, *J* = 8.9 Hz, 2H), 6.62 (d, *J* = 9.0 Hz, 2H), 3.71 (s, 3H), 3.45 (br s, 2H).

^{13}C NMR (125 MHz, CD_2Cl_2, 23 ºC, δ): 153.0, 140.8, 116.4, 115.1, 56.0.

HRMS-FIA(m/z) calc'd for $C_7H_{10}NO$ $[M+H]^+$, 124.0757; found, 124.0758.

2-Methoxyaniline (**S5b**):

R*f* = 0.63 (ethyl acetate/pentanes, 30:70 (v/v)).

NMR Spectroscopy:

^{1}H NMR (500 MHz, $CDCl_3$, 23 ºC, δ): 6.79 (m, 2H), 6.73 (m, 2H), 3.85 (s, 3H), 3.78 (br s, 2H).

^{13}C NMR (125 MHz, $CDCl_3$, 23 ºC, δ): 147.5, 136.3, 121.2, 118.6, 115.2, 110.6, 55.6.

HRMS-FIA(m/z) calc'd for $C_7H_{10}NO$ $[M+H]^+$, 124.0757; found, 124.0755.

3-Methoxyaniline (**S5c**):

R*f* = 0.43 (ethyl acetate/pentanes, 30:70 (v/v)).

NMR Spectroscopy:

^{1}H NMR (500 MHz, $CDCl_3$, 23 ºC, δ): 7.06 (dd, *J* = 8.1, 8.1 Hz, 1H), 6.33 (ddd, *J* = 8.2, 2.3, 0.8 Hz, 1H), 6.30 (ddd, *J* = 7.9, 2.1, 0.8 Hz, 1H), 6.25 (dd, *J* = 2.3, 2.3 Hz, 1H), 3.76 (s, 3H), 3.66 (br s, 2H).

^{13}C NMR (125 MHz, $CDCl_3$, 23 ºC, δ): 160.9, 147.9, 130.3, 108.1, 104.1, 101.2, 55.2.

HRMS-FIA(m/z) calc'd for $C_7H_{10}NO$ $[M+H]^+$, 124.0757; found, 124.0755.

Effect of iron source, oxygen, and light on the amination reaction

Effect of iron and oxygen

The presence of both iron and oxygen has an effect on the reaction time with the shortest reaction times being observed when both are present. 1,4-Dibromobenzene was used to show the effect of iron and oxygen. See below (pg. S31) for a description of reaction setup and trace metal analysis for metal-free experiments.

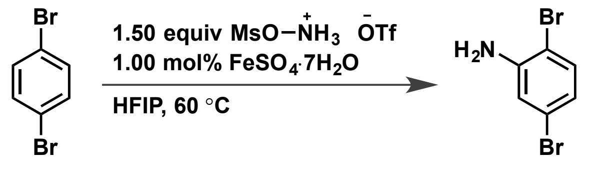

Table S3. Effect of iron and oxygen on reaction time.

conditions	*NMR yield of 2,5-dibromoaniline (**5**)*	*time*
no [Fe], under N_2	88%	7 h
no [Fe], under air	89%	5 h
under N_2	89%	20 min
under air	90%	13 min

Under nitrogen. Reagent **1** (118 mg, 0.450 mmol, 1.50 equiv), $FeSO_4 \cdot 7H_2O$ (0.8 mg, 3 μmol, 0.01 equiv), and 1,4-dibromobenzene (70.8 mg, 0.300 mmol, 1.00 equiv) were added to a flame-dried Schlenk tube. The vessel was evacuated and backfilled with nitrogen three times. Distilled, degassed HFIP (1.5 mL, c = 0.2 M) was then added. The reaction mixture was stirred at 60 °C until judged complete. The red reaction mixture was allowed to cool to room temperature and was concentrated. The residue was dissolved in 10 mL ethyl acetate and poured into a separatory funnel containing 10 mL sat. aq. Na_2CO_3. An ethyl acetate solution containing 1,3,5-trimethoxybenzene (0.1 mmol) was added as an internal standard, and the layers were separated. The aqueous layer was extracted with ethyl acetate (2 × 10 mL). The combined organic layers were dried over sodium sulfate, filtered and concentrated. A ^{1}H NMR spectrum of the residue was taken, and yield was determined based on integration against the internal standard.

Under air. Reagent **1** (118 mg, 0.450 mmol, 1.50 equiv), $FeSO_4 \cdot 7H_2O$ (0.8 mg, 3 μmol, 0.01 equiv), and 1,4-dibromobenzene (70.8 mg, 0.300 mmol, 1.00 equiv) were added to a flame-dried Schlenk tube. Distilled HFIP (1.5 mL, c = 0.2 M) that had been vigorously stirred under air for >20 min was then added. The reaction mixture was stirred at 60 °C until judged complete. The red reaction mixture was allowed to cool to room temperature and was concentrated. The residue was dissolved in 10 mL ethyl acetate and poured into a separatory funnel containing 10 mL sat. aq. Na_2CO_3. An ethyl acetate solution containing 1,3,5-trimethoxybenzene (0.1 mmol) was added as an internal standard, and the layers were separated. The aqueous layer was extracted with ethyl acetate (2 × 10 mL). The combined organic layers were dried over

sodium sulfate, filtered and concentrated. A ^{1}H NMR spectrum of the residue was taken, and yield was determined based on integration against the internal standard.

Effect of iron source

Multiple iron(II) and iron(III) sources were observed to promote the amination reaction effectively, and the reaction also works in the absence of iron for multiple substrates. Nitrobenzene was used to show the effect of the iron source.

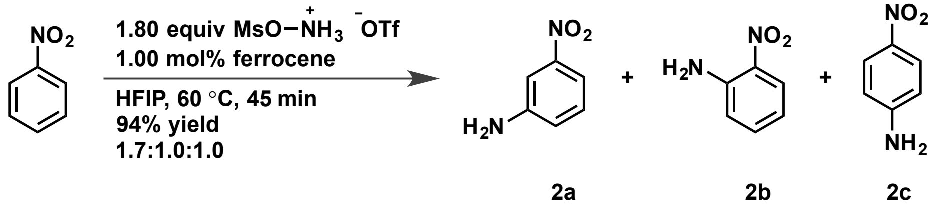

With ferrocene. Reagent **1** (141 mg, 0.540 mmol, 1.80 equiv) and ferrocene (0.6 mg, 3.0 μmol, 0.010 equiv) were added to a 4-mL vial, followed by HFIP (1.5 mL, c = 0.2 M) and nitrobenzene (36.9 mg, 30.8 μL, 0.300 mmol, 1.00 equiv). The reaction mixture was stirred at 60 °C for 45 min. The orange reaction mixture was allowed to cool to room temperature and was concentrated. The residue was dissolved in 10 mL ethyl acetate and poured into a separatory funnel containing 10 mL sat. aq. Na_2CO_3. The layers were separated, and the aqueous layer was extracted with ethyl acetate (2 × 10 mL). The combined organic layers were dried over sodium sulfate, filtered and concentrated. A ^{1}H NMR spectrum of the residue indicated a ratio of **2a**:**2b**:**2c** = 1.7:1.0:1.0 by integrating the signal of **2a** at 6.94 ppm, the signal of **2b** at 6.76 ppm, and the signal of **2c** at 6.62 ppm. The residue was purified by column chromatography on silica gel eluting with a gradient solvent system, starting with a solvent mixture of diethyl ether/pentane (20:80 (v/v)) and finishing with diethyl ether/pentane (40:60 (v/v)). Purification afforded 2-nitroaniline (**2b**), 3-nitroaniline (**2a**) and 4-nitroaniline (**2c**) in separate fractions for a combined yield of 39.0 mg (94% yield).

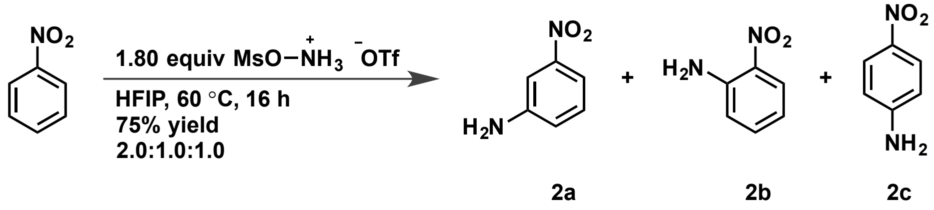

No iron source. Reagent **1** (141 mg, 0.540 mmol, 1.80 equiv) was added to an oven-dried Schlenk tube, followed by HFIP (1.5 mL, c = 0.2 M) and nitrobenzene (36.9 mg, 30.8 μL, 0.300 mmol, 1.00 equiv). The reaction mixture was stirred at 60 °C for 16 h. The orange reaction mixture was allowed to cool to room temperature and was concentrated. The residue was dissolved in 10 mL ethyl acetate and poured into a separatory funnel containing 10 mL sat. aq. Na_2CO_3. The layers were separated, and the aqueous layer was extracted with ethyl acetate (2 × 10 mL). The combined organic layers were dried over sodium sulfate, filtered and concentrated. A ^{1}H NMR spectrum of the residue indicated a ratio of **2a**:**2b**:**2c** = 2.0:1.0:1.0 by integrating the signal of **2a** at 6.94 ppm, the signal of **2b** at 6.76 ppm, and the signal of **2c** at 6.62 ppm. The residue was

purified by column chromatography on silica gel eluting with a gradient solvent system, starting with a solvent mixture of diethyl ether/pentane (20:80 (v/v)) and finishing with diethyl ether/pentane (40:60 (v/v)). Purification afforded 2-nitroaniline (**2b**), 3-nitroaniline (**2a**) and 4-nitroaniline (**2c**) in separate fractions for a combined yield of 31.1 mg (75% yield).

Effect of light

Ambient light was found to have no effect on the results of the amination reaction. A reaction run in the dark (wrapped in foil) gave similar results to the standard reaction conditions.

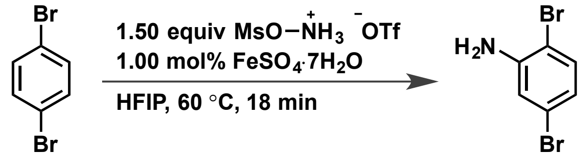

5

**in dark**	**93%**
**standard conditions**	**86%**

Reagent **1** (118 mg, 0.450 mmol, 1.50 equiv), $FeSO_4{\cdot}7H_2O$ (0.8 mg, 3 µmol, 0.01 equiv), and 1,4-dibromobenzene (70.8 mg, 0.300 mmol, 1.00 equiv) were added to a 4-mL vial, followed by HFIP (1.5 mL, c = 0.2 M). The reaction mixture was stirred at 60 °C for 18 min. The red reaction mixture was allowed to cool to room temperature and was concentrated. The residue was dissolved in 10 mL ethyl acetate and poured into a separatory funnel containing 10 mL sat. aq. Na_2CO_3. An ethyl acetate solution containing 1,3,5-trimethoxybenzene (0.1 mmol) was added as an internal standard, and the layers were separated. The aqueous layer was extracted with ethyl acetate (2 × 10 mL). The combined organic layers were dried over sodium sulfate, filtered and concentrated. A ^1H NMR spectrum of the residue was taken, and yield was determined based on integration against the internal standard.

Trace metal analysis

Reactions performed in the absence of $FeSO_4{\cdot}7H_2O$ were conducted with the following precautions: All glassware and stirbars were washed with aqua regia solution, rinsed with deionized water and dried in an oven. Solids were handled with glass pipettes. Solvents were distilled before use.

ICP-MS analysis was performed by Robertson Microlit Laboratories, 1705 U.S. Highway 46, Suite 1D, Ledgewood, NJ 07852. Samples were prepared and sent out for analysis in the following way:

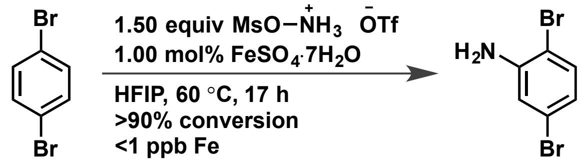

5

Reagent **1** (82.3 mg, 0.315 mmol, 1.05 equiv), $FeSO_4{\cdot}7H_2O$ (0.8 mg, 3 µmol, 0.01 equiv), and 1,4-

dibromobenzene (70.8 mg, 0.300 mmol, 1.00 equiv) were added to a flame-dried Schlenk tube. The vessel was evacuated and backfilled with nitrogen three times. Distilled, degassed HFIP (1.5 mL, c = 0.2 M) was then added. The reaction mixture was stirred at 60 °C for 17 hr. The red reaction mixture was allowed to cool to room temperature. A small aliquot was taken to determine conversion, which was always >90% as judged by ^{1}H NMR. The bulk of the reaction mixture was transferred to a glass vial, sealed and sent out for ICP-MS analysis. Duplicate samples were analyzed in this manner. One contained <1 ppb Fe and 60 ppb Cu, while the other contained <1 ppb Fe and <1 ppb Cu.

Consumption studies of reagent 1

Reagent **1** is consumed to generate methanesulfonic acid (MsOH) even when an arene substrate is not added to the reaction. MsOH is formed faster in the presence of $FeSO_4{\cdot}7H_2O$ and/or residual moisture.

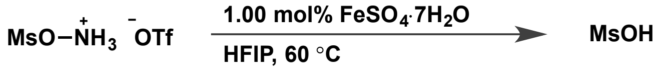

1

Table S4. Consumption of reagent 1 in the absence of arene.

change from reaction conditions	*NMR yield of MsOH*
no [Fe], under N_2, 16h	5%
no [Fe], under air, 16h	33%
under N_2, 4h	33%
under air, 4h	52%
under N_2, 16h	100%
under air, 16h	100%

Under nitrogen. Reagent **1** (78.4 mg, 0.300 mmol, 1.00 equiv) and $FeSO_4{\cdot}7H_2O$ (0.8 mg, 3 μmol, 0.01 equiv) were added to a flame-dried Schlenk tube. The vessel was evacuated and backfilled with nitrogen three times. Distilled, degassed HFIP (1.5 mL, c = 0.2 M) was then added. The reaction mixture was stirred at 60 °C for the designated time. The reaction mixture was allowed to cool to room temperature and was concentrated. A solution containing nitromethane (0.1 mmol) in CD_3CN was added as an internal standard. A ^{1}H NMR spectrum of the residue was taken, and yield was determined based on integration against the internal standard.

Under air. Reagent **1** (78.4 mg, 0.300 mmol, 1.00 equiv) and $FeSO_4{\cdot}7H_2O$ (0.8 mg, 3 μmol, 0.01 equiv) were added to a flame-dried Schlenk tube. Distilled HFIP (1.5 mL, c = 0.2 M) that had been vigorously stirred under air for >20 min was then added. The reaction mixture was stirred at 60 °C for the designated time. The reaction mixture was allowed to cool to room temperature and was concentrated. A solution containing

nitromethane (0.1 mmol) in CD_3CN was added as an internal standard. A ^1H NMR spectrum of the residue was taken, and yield was determined based on integration against the internal standard.

Synthesis of reagent 1 with other counterions

Attempts to synthesize $[MsO–NH_3]^+$ with counterions less capable of hydrogen bonding were unsuccessful:

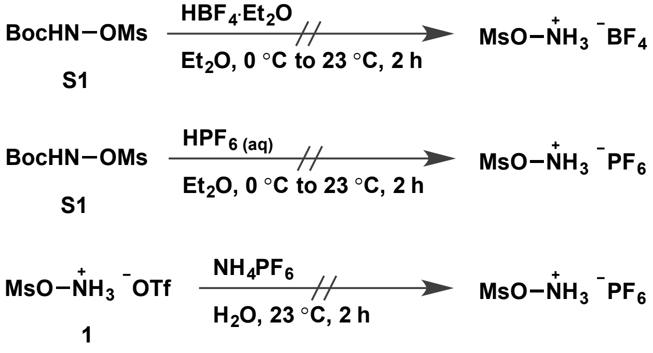

Synthesis of the reagent with a nonaflate counterion was successful, as might be expected due to its similar hydrogen bond donating ability as compared to triflate:

$[MsO–NH_3]ONf$ (S6)

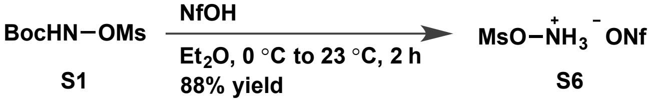

Compound **S1** (1.00 g, 4.73 mmol, 1.00 equiv) was dissolved in anhydrous diethyl ether (24 mL) in a flame-dried round bottom flask. The flask was evacuated and backfilled with nitrogen, then cooled in a water-ice bath. Nonafluorobutanesulfonic acid (1.42 g, 784 µL, 4.73 mmol, 1.00 equiv) was added. The reaction mixture was allowed to stir at room temperature for 2 h, during which time a colorless precipitate formed. Heptane (15 mL) was added to the flask. The colorless solid was collected on a Buchner funnel, rinsed with heptane (10 mL) and dried under high vacuum to give 1.72 g of the title compound as a colorless solid (88% yield). ^{13}C NMR peaks for the nonaflate counterion were not observed due to the complex C–F splitting. The presence of a nonaflate counterion was confirmed by ^{19}F NMR spectroscopy.

NMR Spectroscopy:

^1H NMR (500 MHz, $(CD_3)_2CO$, 23 ºC, δ): 3.15 (s, 3H).

^{13}C NMR (125 MHz, $(CD_3)_2CO$, 23 ºC, δ): 36.6.

^{19}F NMR (470 MHz, CD_3CN, 23 ºC, δ): –82.1 (tt, J = 10.1, 2.8 Hz, 3F), –116.0 (m, 2F), –122.6 (m, 2F), –127.0 (m, 2F).

HRMS-FIA(m/z) calc'd for CH_6NO_3S $[M]^+$, 112.0068; found, 112.0049.

Electrochemical data

General methods

Cyclic voltammetry (CV) was performed in a nitrogen-filled glovebox using a solution of approximately 2 mg/mL of reagent [MsO–NH_3]OTf (**1**) in 0.1 M Bu_4NOTf in either HFIP or MeCN. HFIP and MeCN were distilled and degassed. Bu_4NOTf was recrystallized from DCM/Et_2O at –10 ºC and dried under high vacuum at 65 ºC for 24 h. Cyclic voltammetry was measured using a three-electrode setup with a glassy carbon working electrode, a platinum wire counter electrode and a Ag^0 quasi-reference electrode. Ferrocene was used as an external standard, and potentials are reported vs. Fc/Fc^+. For each solvent, the CV of reagent **1** was measured at five different scan rates (25, 50, 100, 200 and 400 mV/s). The irreversible reduction events are assigned a reduction potential that corresponds to the potential at half the maximum current ($E_{p/2}$).[18]

[MsO–NH_3]OTf (1) in HFIP

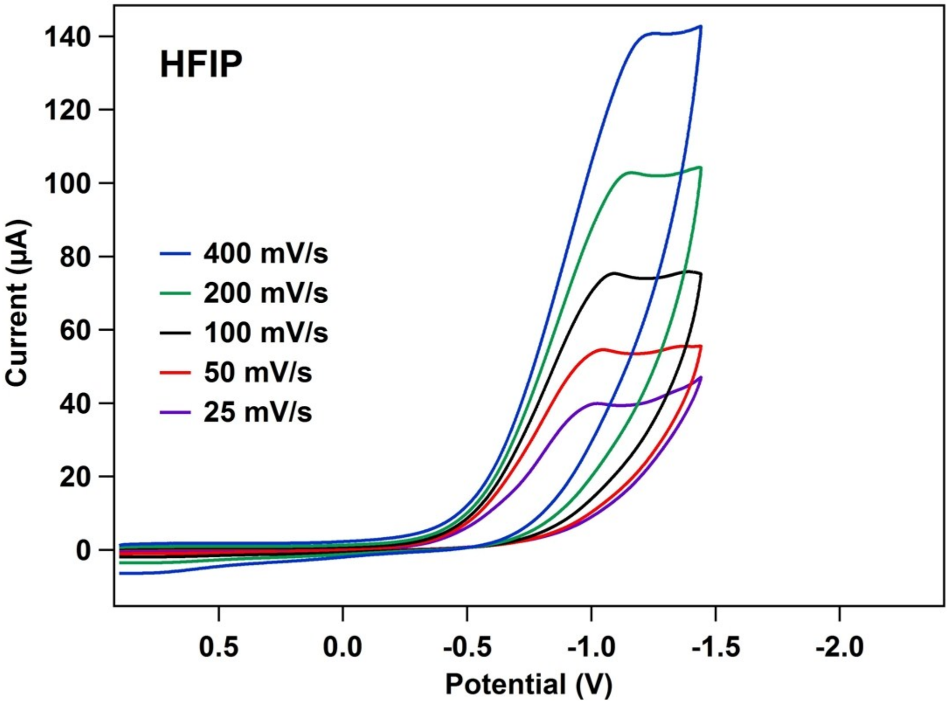

Figure S1. CV of [MsO–NH_3]OTf (**1**) in HFIP referenced to Fc/Fc^+.

[MsO–NH$_3$]OTf (1) in MeCN

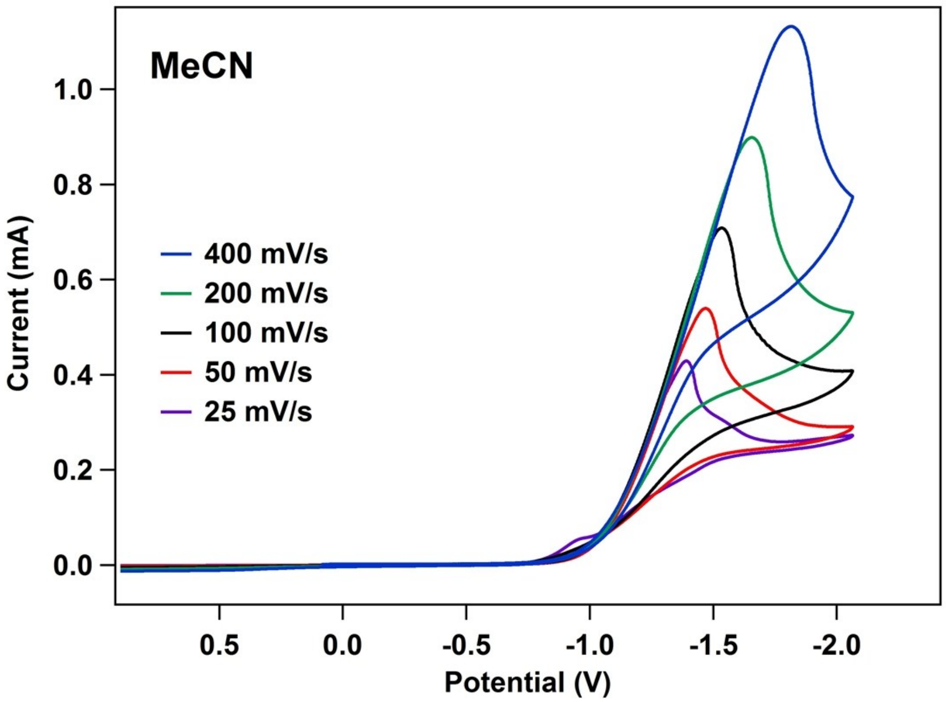

Figure S2. CV of [MsO–NH$_3$]OTf (**1**) in MeCN referenced to Fc/Fc$^+$.

Comparison of electrochemical data

Table S5. Dependence of the reduction potential of [MsO–NH$_3$]OTf (**1**) on scan rate in HFIP and MeCN.

Scan rate (mV/s)	*$E_{p/2}$ (HFIP)*	*$E_{p/2}$ (MeCN)*
25	–0.74 V	–1.12 V
50	–0.74 V	–1.25 V
100	–0.77 V	–1.28 V
200	–0.81 V	–1.35 V
400	–0.86 V	–1.43 V

In both HFIP and MeCN, the reduction potential of [MsO–NH$_3$]OTf (**1**) is scan rate dependent, as is summarized in Table S5. However, there is a clear difference in reduction potential between the two solvents despite the scan rate, with the reduction potential being ~0.5 V less negative in HFIP than MeCN. Therefore, [MsO–NH$_3$]OTf (**1**) is a stronger oxidant in HFIP than in MeCN.

Failed substrates

All reactions have been carried out according to the general procedure for substrate amination. In case of low conversion, the reaction has not been further investigated with respect to products.

Table S6: Substrates that have failed in the described amination reaction.

Substrate	Reaction Outcome
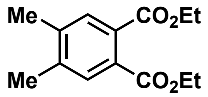	0% conversion
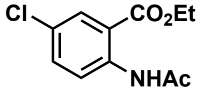	~5% conversion
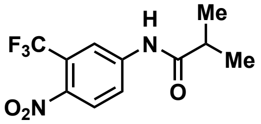	only hydrolysis of amide to the aniline is observed
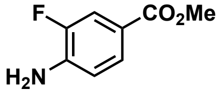	~20% conversion
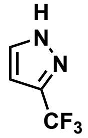	0% conversion
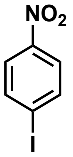	0% conversion

DFT CALCULATIONS

DFT results for 1 in HFIP

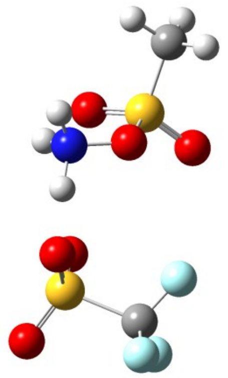

Figure S3. Optimized structure of 1.

Table S7. Cartesian coordinates (Å) of optimized structure of 1 with ωB97XD/BS I.

Atom	X	Y	Z
S	1.796119	-0.804916	-0.286781
F	3.233472	0.913433	1.094697
O	1.334813	-1.387044	1.004446
O	0.697022	-0.643827	-1.234329
F	2.655681	1.609426	-0.867493
F	1.201110	1.548177	0.729083
O	3.028937	-1.381853	-0.790420
C	2.250219	0.926183	0.198942
S	-2.693440	0.317373	-0.287657
O	-2.713364	-0.775045	-1.229209
O	-2.222457	1.624092	-0.631403
O	-1.621029	-0.178356	0.938170
N	-1.257345	-1.527771	0.895352
C	-4.210494	0.407179	0.617176

H	-1.764932	-2.035396	1.622092
H	-0.186057	-1.544840	1.044402
H	-1.470844	-1.919339	-0.033632
H	-4.963931	0.769301	-0.081610
H	-4.075160	1.110424	1.435881
H	-4.462625	-0.588301	0.975089

DFT results for 1 in MeCN

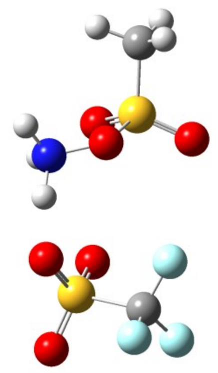

Figure S4. Optimized structure of 1.

Table S8. Cartesian coordinates (Å) of optimized structure of 1 with ωB97XD/BS I.

Atom	X	Y	Z
S	1.800599	-0.804773	-0.288553
F	3.232280	0.904859	1.109498
O	1.329137	-1.385153	0.998805
O	0.711034	-0.637109	-1.245728
F	2.686222	1.601880	-0.861530
F	1.209946	1.554982	0.715742
O	3.034840	-1.387158	-0.783772
C	2.261007	0.923572	0.200738

S	-2.704718	0.316999	-0.288489
O	-2.722171	-0.777341	-1.227877
O	-2.237715	1.624202	-0.636971
O	-1.623723	-0.169575	0.932908
N	-1.268377	-1.521772	0.904108
C	-4.219560	0.403721	0.619703
H	-1.769899	-2.016751	1.643961
H	-0.197729	-1.542517	1.042738
H	-1.495674	-1.925443	-0.016270
H	-4.976867	0.755550	-0.079961
H	-4.086409	1.114135	1.432451
H	-4.464816	-0.590362	0.986057

The hydrogen bond length between the triflate anion and the $[MsO–NH_3]^+$ cation in the optimized structures in continuum HFIP and continuum acetonitrile is found to be 1.53 Å, which is 0.40 Å shorter than in the crystal structure. Significantly shorter ion-pair distances in calculated structures have been observed previously and were attributed to steric effects.[19]

DFT results for HFIP

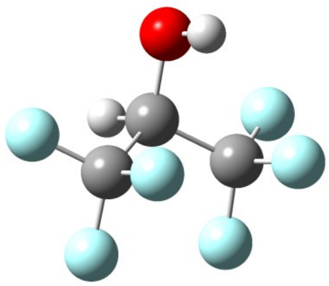

Figure S5. Optimized structure of HFIP.

Table S9. Cartesian coordinates (Å) of optimized structure of HFIP with ωB97XD/BS I.

Atom	X	Y	Z
H	-0.000233	1.962522	0.779924
C	0.000008	0.533793	-0.530643
H	0.000092	0.471179	-1.619331
C	-1.284463	-0.150012	-0.042764
C	1.284507	-0.149938	-0.042746
O	-0.000104	1.879411	-0.179037
F	-2.345488	0.456622	-0.580588
F	-1.406670	-0.064486	1.290796
F	-1.336208	-1.440603	-0.378578
F	1.336619	-1.440255	-0.379610
F	2.345565	0.457321	-0.579751
F	1.406255	-0.065493	1.290911

DFT results for 1·HFIP (OMs)

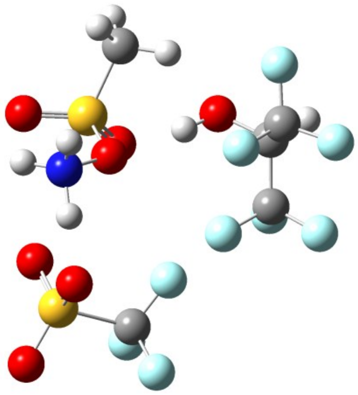

Figure S6. Optimized structure of 1·HFIP (OMs).

Table S10. Cartesian coordinates (Å) of optimized structure of 1·HFIP (OMs) with ωB97XD/BS I.

Atom	X	Y	Z
S	-3.473665	-0.641749	-0.038481
F	-2.574829	-2.856120	1.065888

O	-3.541288	0.019003	1.295876
O	-2.926154	0.251969	-1.055774
F	-1.843894	-2.498932	-0.936160
F	-1.060129	-1.356094	0.722125
O	-4.661430	-1.397788	-0.388175
C	-2.153618	-1.919969	0.220176
S	-0.276784	2.473025	-0.641640
O	-1.333915	3.166014	-1.332130
O	-0.986649	1.842275	0.761408
N	-2.292756	2.277462	1.011275
C	0.900786	3.591360	0.058247
H	-2.273532	2.963178	1.768576
H	-2.853405	1.383948	1.258724
H	-2.691716	2.696947	0.158643
H	1.490352	3.978178	-0.772333
H	1.527575	3.030140	0.747831
H	0.363456	4.391875	0.561755
O	0.386066	1.338785	-1.222309
H	2.134488	0.684036	-1.354443
C	3.457469	-0.369143	-0.389210
H	4.538516	-0.508081	-0.441072
C	2.823083	-1.746074	-0.632998
C	3.162000	0.202023	1.005038
O	3.089296	0.521854	-1.385669
F	3.289880	-2.254832	-1.777404
F	1.492084	-1.660210	-0.746800
F	3.097532	-2.616680	0.342911
F	3.641742	-0.566592	1.982893

F	3.722684	1.410987	1.120870
F	1.843264	0.359701	1.217009

1·HFIP (OMs) is found to be 5.9 kcal/mol higher in energy than **1** and a free HFIP molecule.

DFT results for 1·HFIP (OTf)

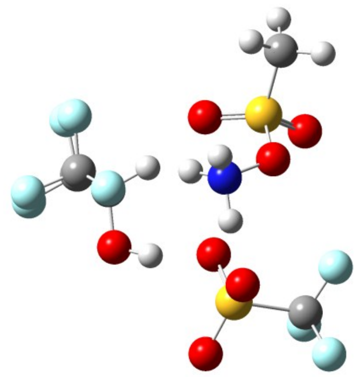

Figure S7. Optimized structure of 1·HFIP (OTf).

Table S11. Cartesian coordinates (Å) of optimized structure of 1·HFIP (OTf) with ωB97XD/BS I.

Atom	X	Y	Z
S	2.086636	-1.472640	0.603406
F	4.555115	-1.821527	-0.218333
O	2.580400	-0.658431	1.737944
O	0.918230	-0.842606	-0.037118
F	3.071290	-1.915007	-1.786489
F	3.629124	-0.007237	-0.938678
O	1.985760	-2.894410	0.859339
C	3.423813	-1.290519	-0.668777
S	0.626680	2.711856	-0.566268
O	1.724792	2.175883	0.618847
N	1.151856	1.550538	1.730173
C	0.582973	4.448615	-0.236207

H	1.150055	2.199820	2.519533
H	1.760740	0.689616	1.903854
H	0.191275	1.235679	1.516733
H	-0.056960	4.886772	-1.001518
H	1.596659	4.834943	-0.313492
H	0.160892	4.606189	0.753422
O	1.305962	2.435150	-1.794070
O	-0.627927	2.090055	-0.216739
C	-2.293283	-0.651059	-0.221455
H	-1.732267	0.154097	-0.706153
H	-0.635401	-1.616283	-0.125543
O	-1.584436	-1.838152	-0.125014
C	-3.523163	-0.918606	-1.089343
C	-2.644023	-0.139804	1.181565
F	-3.135920	-1.235620	-2.329318
F	-4.262545	-1.931611	-0.627063
F	-4.316238	0.157937	-1.175353
F	-3.403903	-0.989740	1.872302
F	-3.260502	1.043952	1.160304
F	-1.499886	0.020663	1.887910

1·HFIP (OTf) is found to be 0.8 kcal/mol higher in energy than **1** and a free HFIP molecule.

DFT results for 1·2HFIP

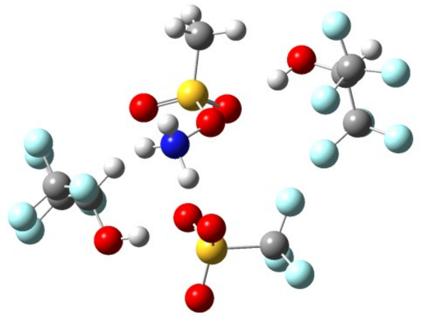

Figure S8. Optimized structure of 1·2HFIP.

Table S12. Cartesian coordinates (Å) of optimized structure of 1·2HFIP with ωB97XD/BS I.

Atom	X	Y	Z
S	-0.789858	2.765024	-0.382283
F	1.350688	4.225629	0.037819
O	-0.587276	2.820805	-1.849649
O	-1.154309	1.405516	0.053830
F	0.919508	2.804574	1.607809
F	1.754361	2.125630	-0.265841
O	-1.576337	3.846210	0.173573
C	0.922983	2.994238	0.293868
S	0.102062	-1.586078	-1.233650
O	0.354402	-0.181709	-2.146124
N	-0.794228	0.364108	-2.729221
C	0.843838	-2.804323	-2.276980
H	-0.784355	0.161335	-3.730895
H	-0.741221	1.414859	-2.517723
H	-1.644264	-0.027467	-2.292000

H	0.794787	-3.745324	-1.729728
H	1.877642	-2.516411	-2.455727
H	0.273082	-2.861408	-3.201632
O	0.887191	-1.371342	-0.051360
H	2.509902	-2.007067	0.605562
C	4.404047	-1.587500	0.772822
H	5.309608	-2.141903	1.024597
C	4.252244	-0.481106	1.827313
C	4.615699	-1.041186	-0.647408
O	3.332933	-2.465519	0.830827
F	4.304487	-1.017002	3.050420
F	3.073605	0.145254	1.715358
F	5.216458	0.440855	1.742608
F	5.780160	-0.398135	-0.764798
F	4.617828	-2.053340	-1.521813
F	3.642567	-0.196697	-1.020417
O	-1.329689	-1.741122	-1.166780
C	-3.658520	-0.537915	0.720610
H	-2.701307	-1.041782	0.556025
H	-2.663683	1.100543	0.861950
O	-3.526258	0.766717	1.169224
C	-4.389542	-1.325952	1.808287
C	-4.384917	-0.552353	-0.630426
F	-3.641799	-1.358899	2.916458
F	-5.565217	-0.777456	2.131911
F	-4.619441	-2.592765	1.436937
F	-5.604591	-0.017121	-0.570563
F	-4.495458	-1.780281	-1.143229

F	-3.678278	0.186739	-1.516858

1·2HFIP is found to be 6.6 kcal/mol higher in energy than **1** and two free HFIP molecules, which suggests HFIP destabilizes reagent **1**. The sum of the individual effects of one HFIP hydrogen bonding to $[MsO–NH_3]^+$ (5.9 kcal/mol) and one HFIP hydrogen bonding to the triflate counterion (0.8 kcal/mol) is 6.7 kcal/mol, which is consistent with the results of the calculation with two HFIP hydrogen bonding interactions.

DFT results for $[NH_3]^{+\cdot}$(OTf) (S7)

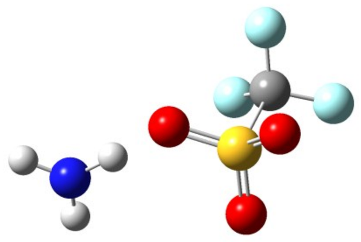

Figure S9. Optimized structure of $[NH_3]^{+\cdot}$(OTf).

Table S13. Cartesian coordinates (Å) of optimized structure of $[NH_3]^{+\cdot}$(OTf) with ωB97XD/BS I.

Atom	X	Y	Z
S	-0.088001	0.824335	0.019420
F	1.473110	-0.886948	-1.239373
O	-1.198631	0.352257	-0.856073
O	-0.475925	0.959109	1.415595
F	2.120072	-0.394575	0.761894
F	0.434535	-1.714799	0.465358
O	0.679733	1.907240	-0.568632
C	1.057172	-0.634100	-0.001692
N	-3.324336	-0.817477	-0.011555
H	-4.037069	-1.124406	-0.669113
H	-2.414690	-0.323134	-0.342151

H	-3.503772	-0.996815	0.973541

DFT results for MsO$^{\cdot}$ (S8)

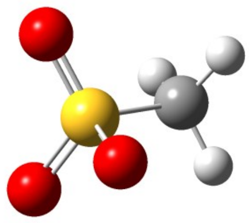

Figure S10. Optimized structure of MsO$^{\cdot}$.

Table S14. Cartesian coordinates (Å) of optimized structure of MsO$^{\cdot}$ with ωB97XD/BS I.

Atom	X	Y	Z
S	-0.100444	0.072405	0.000071
O	-0.373948	1.488671	0.000135
O	-0.700174	-0.694735	-1.136201
O	-0.700326	-0.695102	1.135911
C	1.644592	-0.200872	0.000029
H	2.055581	0.264806	-0.894046
H	1.823804	-1.273744	0.000077
H	2.055743	0.265026	0.893903

Calculation of homolysis energy

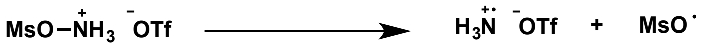

1 **S7** **S8**

The homolysis energy $E_{homolysis}$ of **1** has been calculated using the following equation:

$$E_{homolysis} = E(S7) + E(S8) - E(1) = 35.6\ kcal * mol^{-1} \quad (1)$$

The homolysis energy of $35.6\ kcal * mol^{-1}$ implies that homolysis is a feasible mechanistic step to generate ammoniumyl and mesyloxyl radicals.

Comparison of LUMO energy differences and reduction potentials

The LUMO energies of **1**, **1·HFIP(OMs)**, **1·HFIP(OTf)** and **1·2HFIP** have been calculated by addition of the corresponding HOMO energies to the transition energy ΔE^1 to the first excited state determined by TD-DFT calculations.[20]

$$\Delta E^1 = E(LUMO) – E(HOMO) \leftrightarrow E(LUMO) = \Delta E^1 + E(HOMO) \qquad (2)$$

The LUMO of **1·2HFIP** is 7.7 kcal·mol^{-1} lower in energy than the LUMO of **1** in HFIP and 8.3 kcal·mol^{-1} lower than the LUMO of **1** in acetonitrile. The LUMO of **1·HFIP(OMs)** is 4.8 kcal·mol^{-1} lower in energy than the LUMO of **1** and the LUMO of **1·HFIP(OTf)** is 3.2 kcal·mol^{-1} lower than the LUMO of **1**. Their sum (8.0 kcal·mol^{-1}) is consistent with the value calculated from **1·2HFIP**.

The energy difference of 8.3 kcal mol^{-1} would correspond to a difference in the reduction potential of ΔE = 0.4 V between **1** in MeCN and **1·2HFIP** coordination. This value is consistent with our experimentally measured CV data, which show ~0.5 V difference between the reduction potential of **1** in MeCN and HFIP (see Table S5).

$$\Delta E = \frac{34.7\,J}{96.5\,C} = 0.4\,V$$

The LUMO of **1·2 HFIP** shows large contributions of the σ*(N–O) orbital.

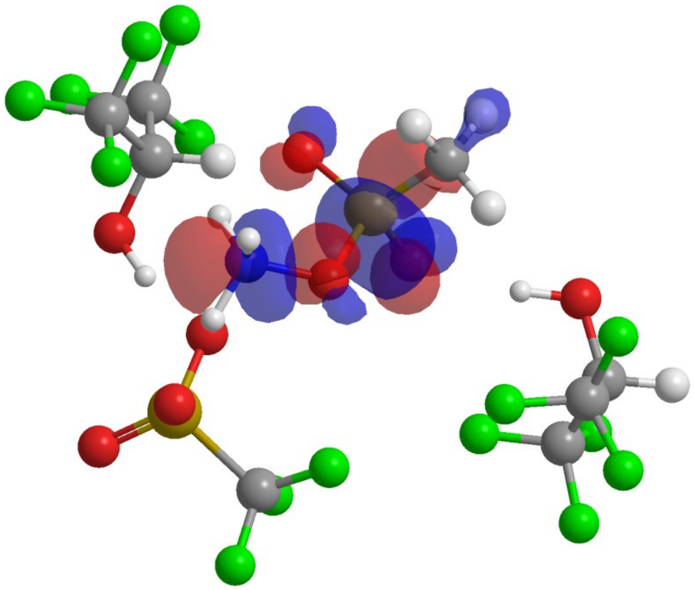

Figure S11. LUMO of 1·2HFIP plotted with an isosurface value of 0.05.

X-RAY CRYSTALLOGRAPHIC ANALYSIS

[MsO–NH$_3$]OTf (1) (CCDC 1545194)

Reagent **1** was crystallized from HFIP (~20 mg in 2 mL) at –10 ºC. A crystal was mounted on a nylon loop using Paratone-N oil, and transferred to a Bruker APEX II CCD diffractometer (MoKα radiation, λ = 0.71073 Å) equipped with an Oxford Cryosystems nitrogen flow apparatus. The sample was held at 100 K during the experiment. The collection method involved 0.5º scans in ω at 28º in 2θ. Data integration down to 0.82 Å resolution was carried out using SAINT V8.34 C (Bruker diffractometer, 2014) with reflection spot size optimization. Absorption corrections were made with the programs SADABS (Bruker diffractometer, 2014). The structure was solved by the direct methods procedure and refined by least-squares methods against F2 using SHELXT-2014 and SHELXL-2014 (Sheldrick, 2015). Non-hydrogen atoms were refined anisotropically, and hydrogen atoms were allowed to ride on the respective atoms. Crystal data as well as details of data collection and refinement are summarized in Table S14 below.

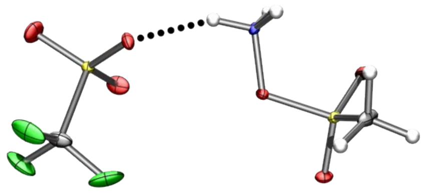

Figure S12. X-ray crystal structure of [MsO–NH$_3$]OTf (**1**). Thermal ellipsoids are drawn at 50% probability level. Red = oxygen, blue = nitrogen, yellow = sulfur, green = fluorine.

Table S15. Experimental details for [MsO–NH$_3$]OTf (**1**).

Empirical formula	$C_2H_6F_3NO_6S_2$	
Formula weight	261.20	
Temperature	100(2) K	
Wavelength	0.71073 Å	
Crystal system	orthorhombic	
Space group	$Pna2_1$	
Unit cell dimensions	a = 8.9227(15) Å	α = 90°
	b = 18.531(3) Å	β = 90°
	c = 5.5268(9) Å	γ = 90°
Volume	913.8(3) Å^3	
Z	4	
Density (calculated)	1.899 Mg/m^3	

Absorption coefficient	0.639 mm^{-1}
F(000)	528.0
Crystal size	0.82 × 0.13 × 0.08 mm^3
θ range for data collection	2.198 to 25.013°
Index ranges	$-10 \le h \le 10$, $-22 \le k \le 22$, $-6 \le l \le 6$
Reflections collected	12755
Independent reflections	1623 [R_{int} = 0.0221]
Reflections with I>2σ(I)	1597
Max. and min. transmission	0.7772 and 0.8620
Data / restraints / parameters	1623 / 1 / 129
Goodness-of-fit on F^2	1.080
Final R indices [I>2σ(I)]	R_1 = 0.0228, wR^2 = 0.0557
R indices (all data)	R_1 = 0.0232, wR^2 = 0.0560
Largest diff. peak and hole	0.356 and -0.289 e·$Å^{-3}$

REFERENCES

1 A. B Pangborn,. M. A. Giardello, R. H Grubbs, R. K. Rosen and F. J. Timmers, *Organometallics,* 1996, **15**, 1518–1520.

2 G. R. Fulmer, A. J. Miller, N. H. Sherden, H. E. Gottlieb, A. Nudelman, B. M. Stoltz, J. E. Bercaw and K. I. Goldberg *Organometallics,* 2010, **29**, 2176–2179.

3 M. J. Frisch, G. W. Trucks, H. B. Schlegel, G. E. Scuseria, M. A. Robb, J. R. Cheeseman, G. Scalmani, V. Barone, B. Mennucci, G. A. Petersson, H. Nakatsuji, M. Caricato, X. Li, H. P. Hratchian, A. F. Izmaylov, J. Bloino, G. Zheng, J. L. Sonnenberg, M. Hada, M. Ehara, K. Toyota, R. Fukuda, J. Hasegawa, M. Ishida, T. Nakajima, Y. Honda, O. Kitao, H. Nakai, T. Vreven, J. A. Montgomery Jr., J. E. Peralta, F. Ogliaro, M. Bearpark, J. J. Heyd, E. Brothers, K.N. Kudin, V. N. Staroverov, J. Normand, K. Raghavachari, A. Rendell, J.C. Burant, S. S. Iyengar, J. M. Cossi, N. Rega, J. M. Millam, M. Klene, J. E. Knox, J. B. Cross, V. Bakken, C. Adam, J. Jaramillo, R. Gomperts, R. E. Stratmann, O. Yazyev, A. J. Austin, R. Cammi, C. Pomelli, J. W. Ochterski, R. L. Martin, K. Morokuma, V. G. Zakrzewski, G. A. Voth, P. Salvador, J. J. Dannenberg, S. Dapprich, A. D.Daniels, O. Farkas, J. B. Foresman, J. V. Ortiz, J. Cioslowski and D. J. Fox, Gaussian 09(Revision D.01), Gaussian Inc., Wallingford, CT, 2013.

4. P. C. Harihara and J. A. Pople, *Theor. Chim. Acta,* 1973, **28**, 213–222.

5. J. D. Chai and M. Head-Gordon, *Phys. Chem. Chem. Phys.,* 2008, **10**, 6615–6620.

6. D.-P. Hong, M. Hoshino, R. Kuboi and Y. Goto, *J. Am. Chem. Soc.,* 1999, **121**, 8427–8433; b. V. Barone and M. Cossi, *J. Phys. Chem. A,* 1998, **102**, 1995–2001.

7. J. Tomasi, B. Mennucci and R. Cammi, *Chem. Rev.,* 2005, **105**, 2999–3094.

8. J. A. Stafford, S. S. Gonzales, D. G. Barrett, E. M. Suh and P. L. Feldman, *J. Org. Chem.,* 1998, **63**, 10040–10044.

9. a. L. Legnani, G. Prina Cerai and B. Morandi, *ACS Catal.,* 2016, **6**, 8162–8165; b. N. Guimond, S. I. Gorelsky and K. Fagnou, *J. Am. Chem. Soc.,* 2011, **133**, 6449–6457.

10. D. Kim, S. Sambasivan, H. Nam, K. H: Kim, J. Y. Kim, T. Joo, K.-H. Lee, K.-T. Kim and K. H. Ahn, *Chem. Commun.,* 2012, **48**, 6833–6835.

11. S. Thea, G. Guanti, A. R. Hopkins and A. Williams, *J. Org. Chem.,* 1985, **50**, 3336–3341.

12. X. Gao, H. Fu, R. Qiao, Y. Jiang and Y. Zhao, *J. Org. Chem.,* 2008, **73**, 6864–6866.

13. a. K. K. Anderson, S. Chumpradit and D. J. McIntyre, *J. Org. Chem.,* 1988, **53**, 4667–4675; b. A. Courtin, *Helv. Chim. Acta,* 1983, **66**, 1046–1052.

14. R. J. Rahaim Jr. and R. E. Maleczka Jr., *Org. Lett.,* 2005, **7**, 5087–5090.

15. A. Robin, J.-C. Meslin and D. Deniaud, *Synthesis,* 2004, 1633–1640.

16. E. A. Meyer, N. Donati, M. Guillot, W. B. Schweizer, F. Diederich, B. Stengl, R. Brenk, K. Reuter and G. Klebe, *Helv. Chim. Acta,* 2006, **89**, 573–597.

17. A. Citterio, A. Gentile, F. Minisci, V. Navarrini, M. Serravalle and S.Ventura, *J. Org. Chem.,* 1984, **49**, 4479–4482.

18. H. G. Roth, N. A. Romero and D. A. Nicewicz, *Synlett,* 2016, **27**, 714–723.

19. S. Zhang, Z. Chen, Y. Lu, Z. Xu, W. Wu, W. Zhu, C. Peng and H. Liu, *RSC Adv.,* 2015, **5**, 74284–74294.

20. G. Zhang and C. B. Musgrave, *J. Phys. Chem. A,* 2007, **111**, 1554–1561.

SPECTROSCOPIC DATA

^{1}H NMR of *N*-Boc-*O*-mesylhydroxylamine (S1)

BocHN−OMs

S1

$CDCl_3$, 23 ºC

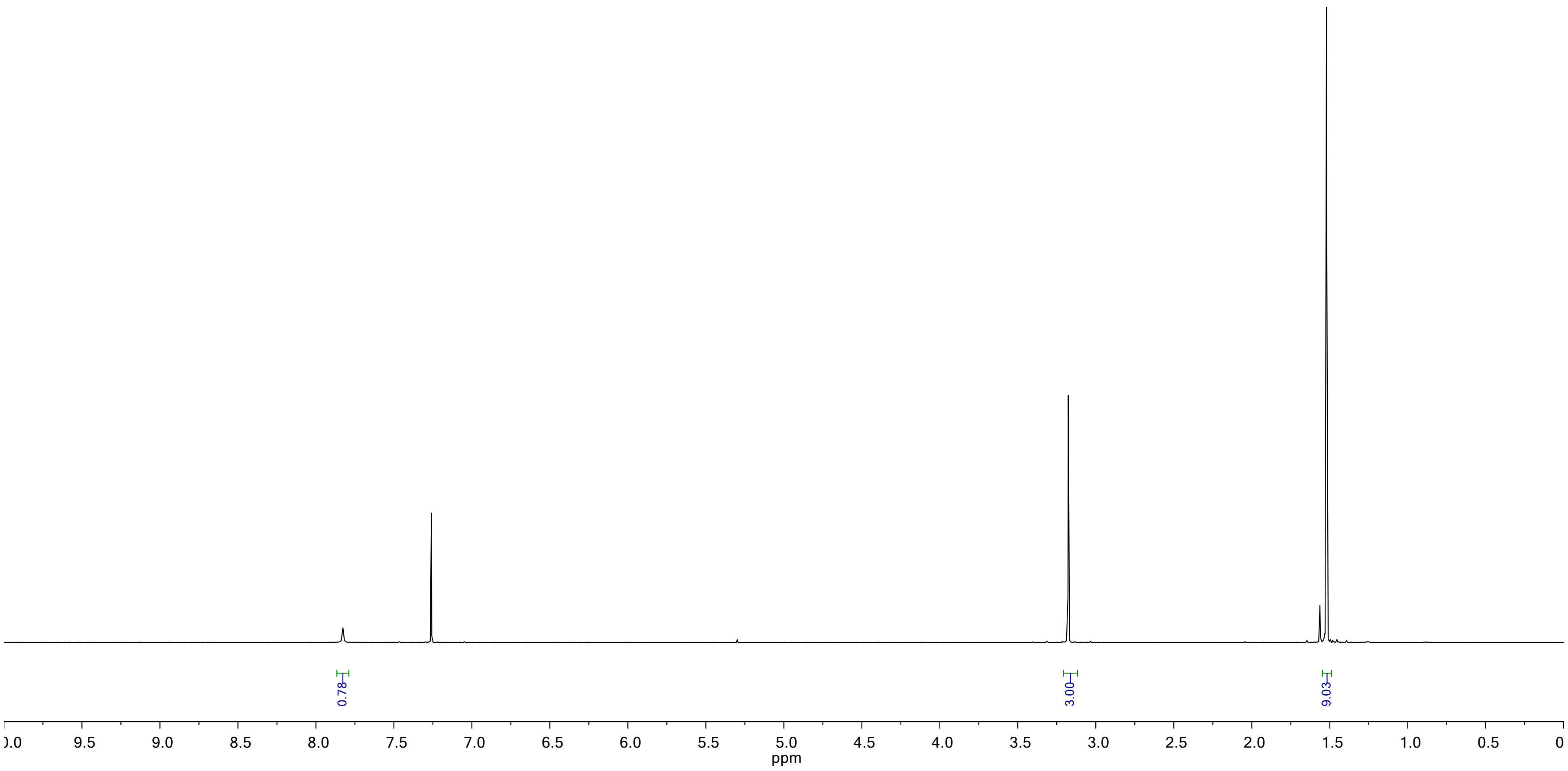

^{13}C NMR of *N*-Boc-*O*-mesylhydroxylamine (S1)

CDCl$_3$, 23 ºC

BocHN−OMs

S1

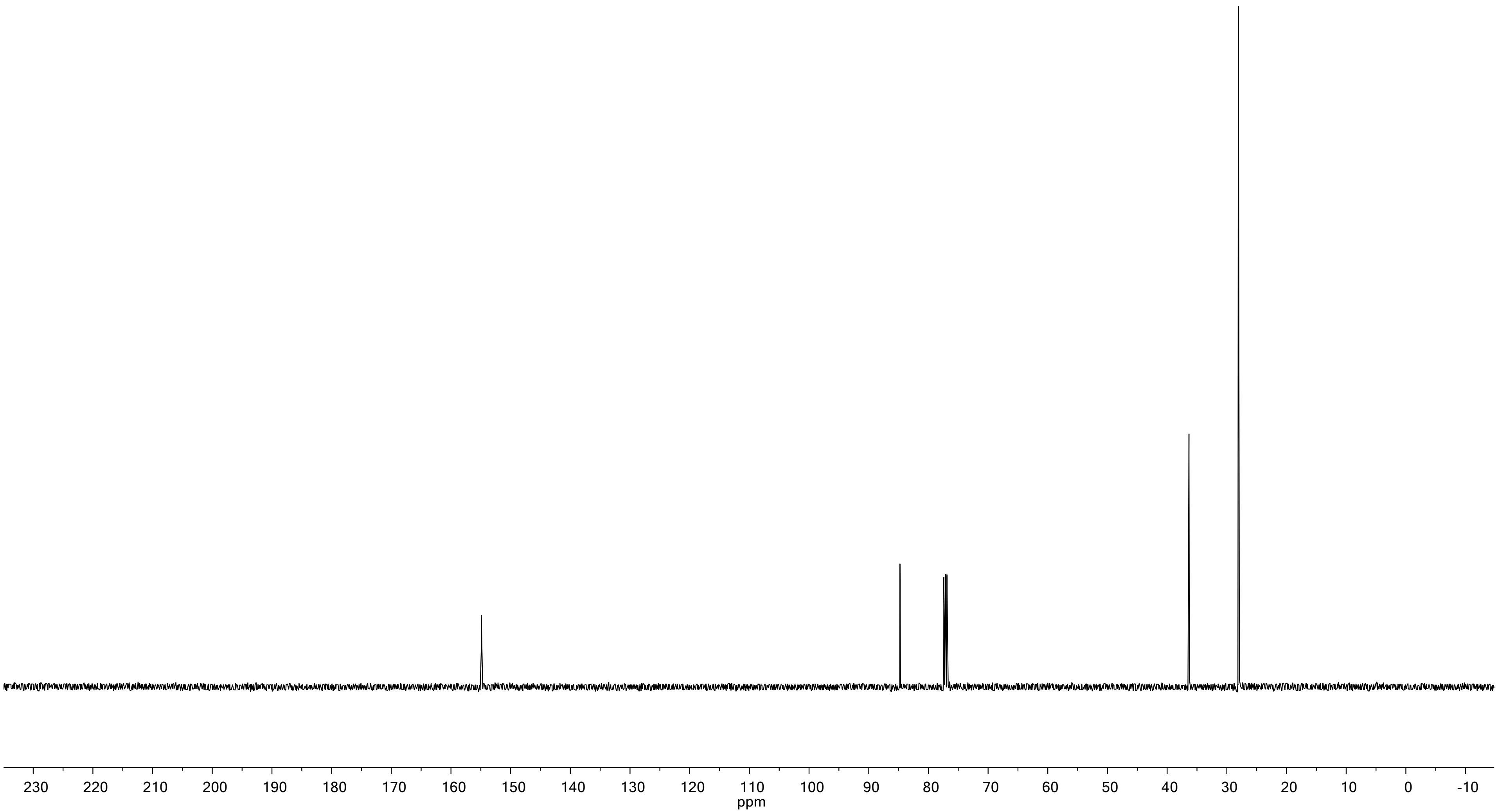

^{1}H NMR of [MsO–NH_3]OTf (1)

CD_3CN, 23 ºC

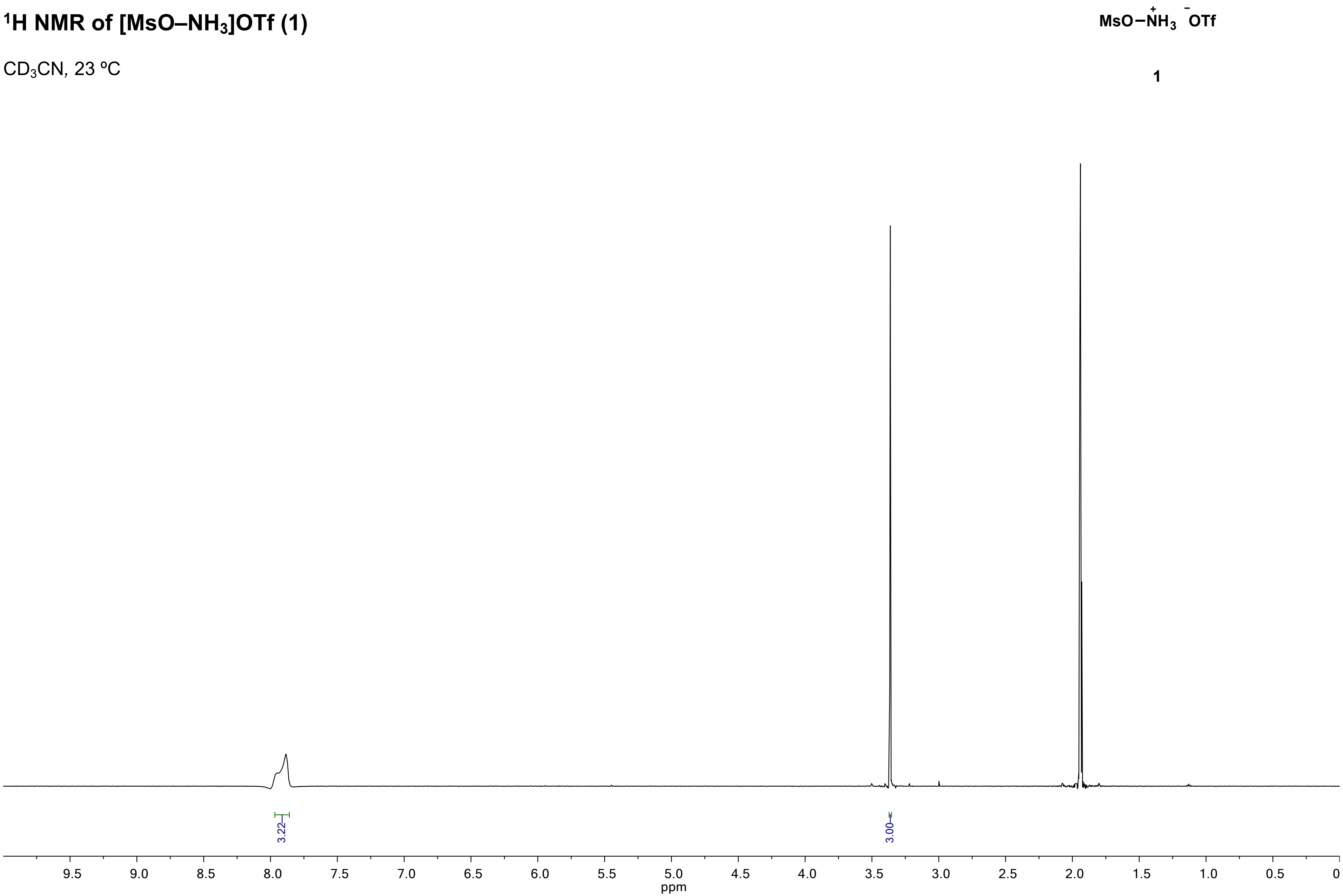

^{13}C NMR of [MsO–NH_3]OTf (1)

CD_3CN, 23 ºC

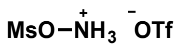

1

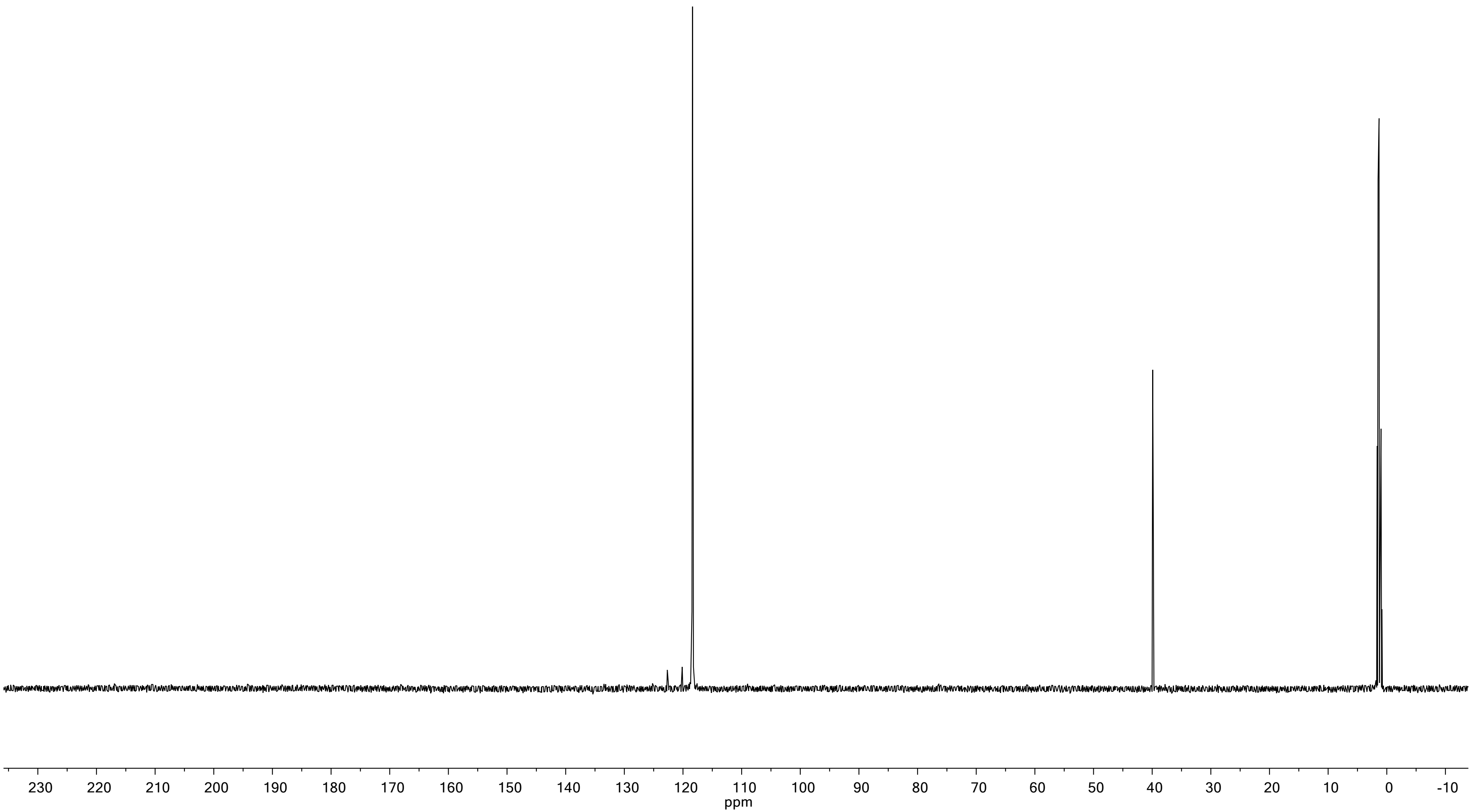

^{19}F NMR of [MsO–NH$_3$]OTf (1)

CD_3CN, 23 ºC

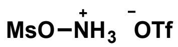

1

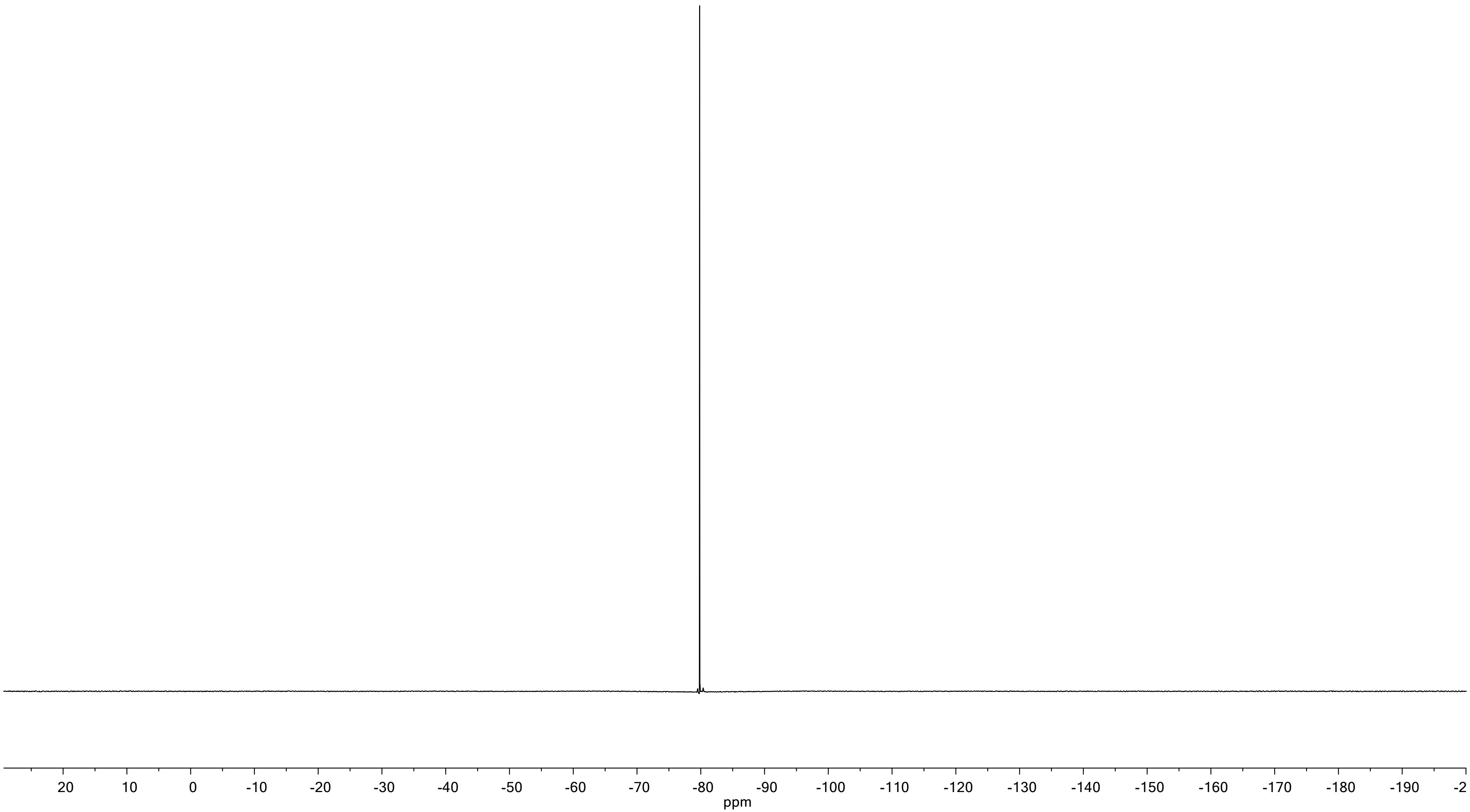

^{1}H NMR of moclebomide (S2)

$CDCl_3$, 23 ºC

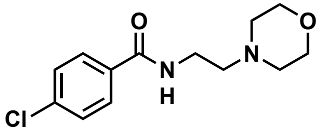

S2

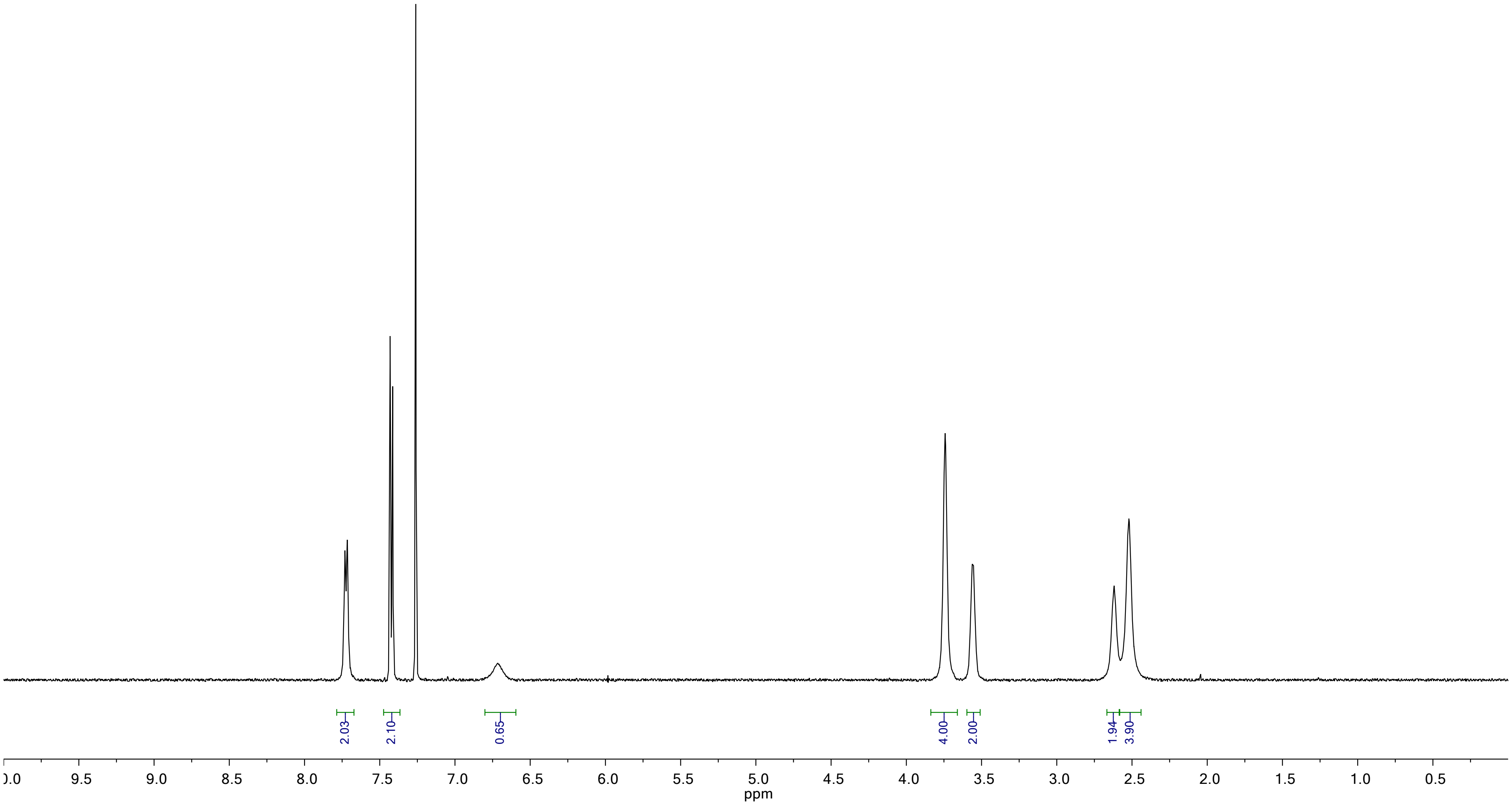

^{13}C NMR of moclebomide (S2)

$CDCl_3$, 23 ºC

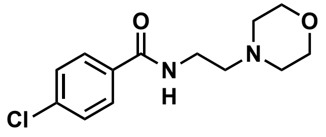

S2

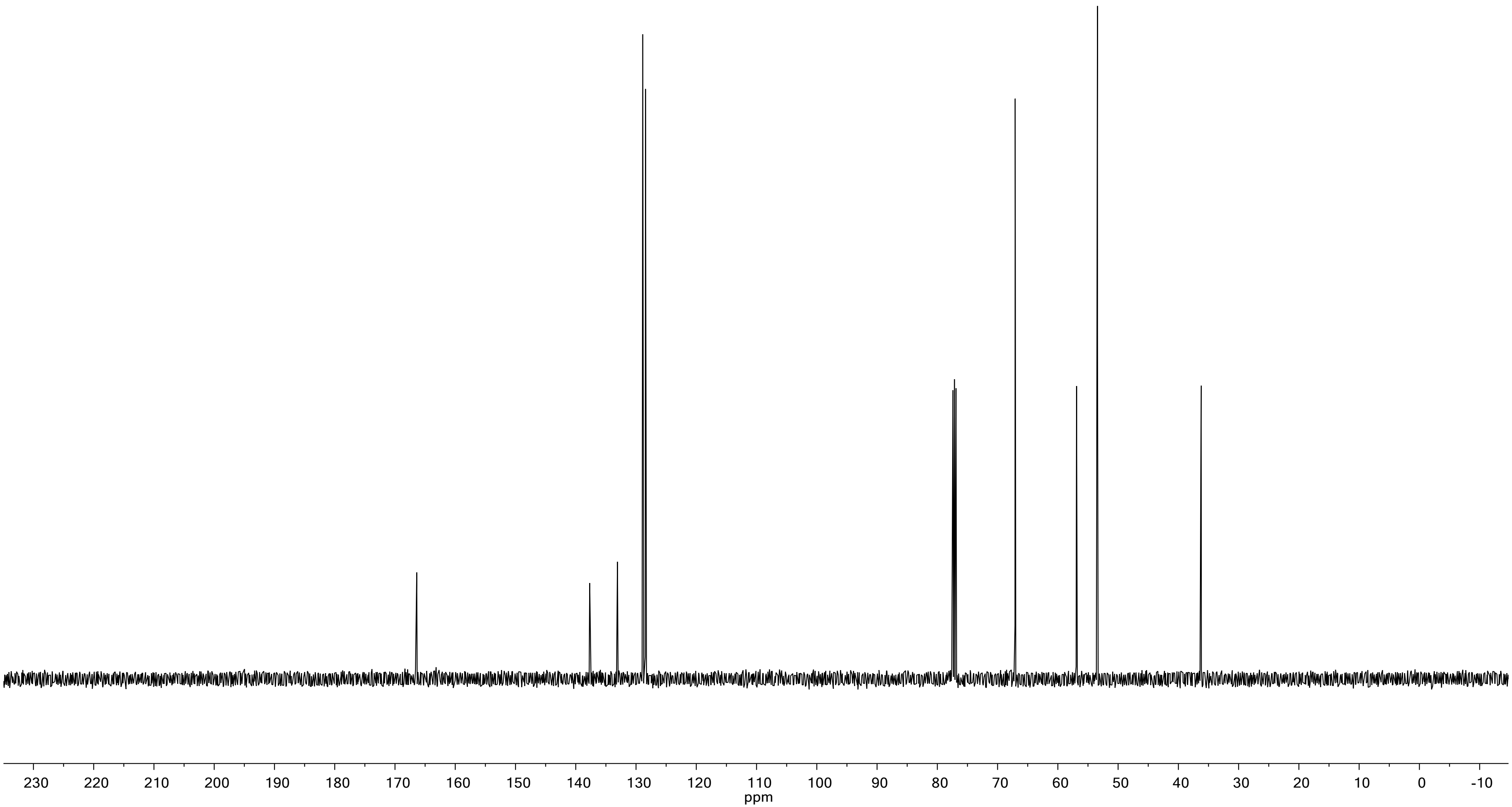

^{1}H NMR of 4-(trifluoromethyl)phenyl-2-nitrobenzenesulfonate (S3)

$CDCl_3$, 23 ºC

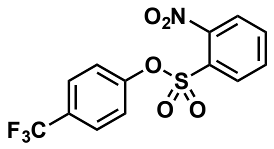

S3

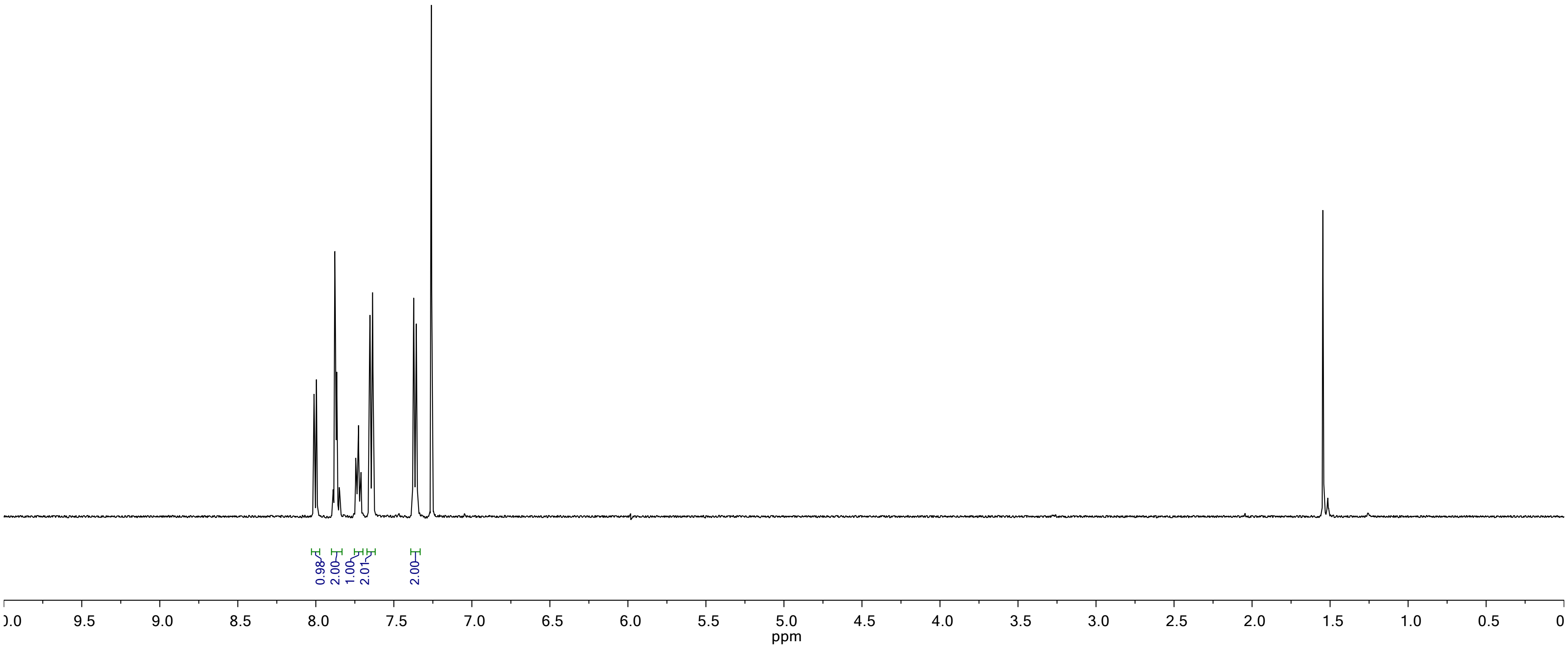

^{13}C NMR of 4-(trifluoromethyl)phenyl-2-nitrobenzenesulfonate (S3)

$CDCl_3$, 23 ºC

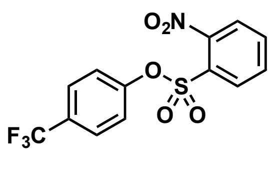

S3

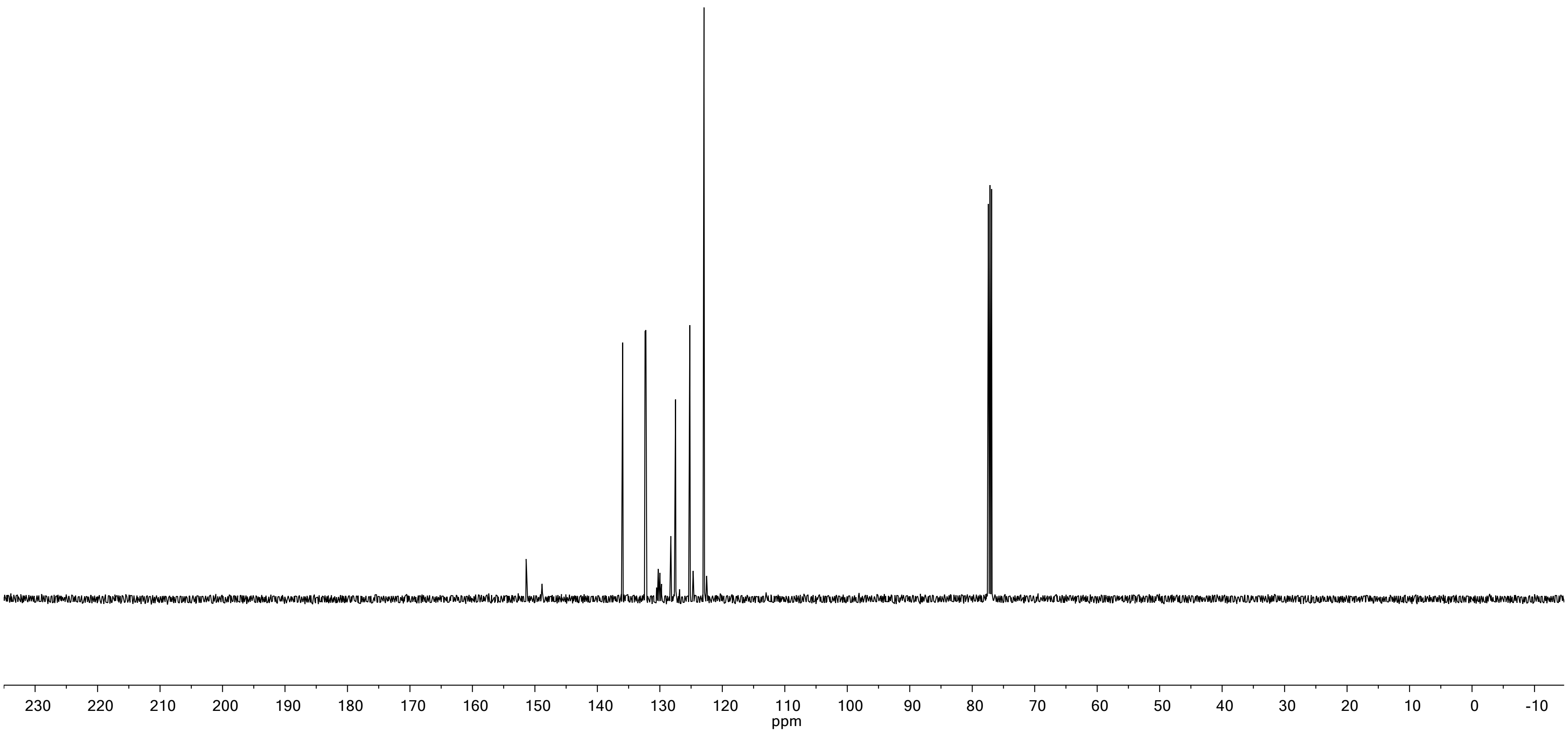

^{19}F NMR of 4-(trifluoromethyl)phenyl-2-nitrobenzenesulfonate (S3)

$CDCl_3$, 23 ºC

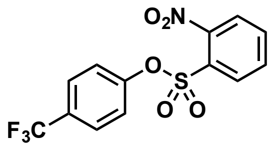

S3

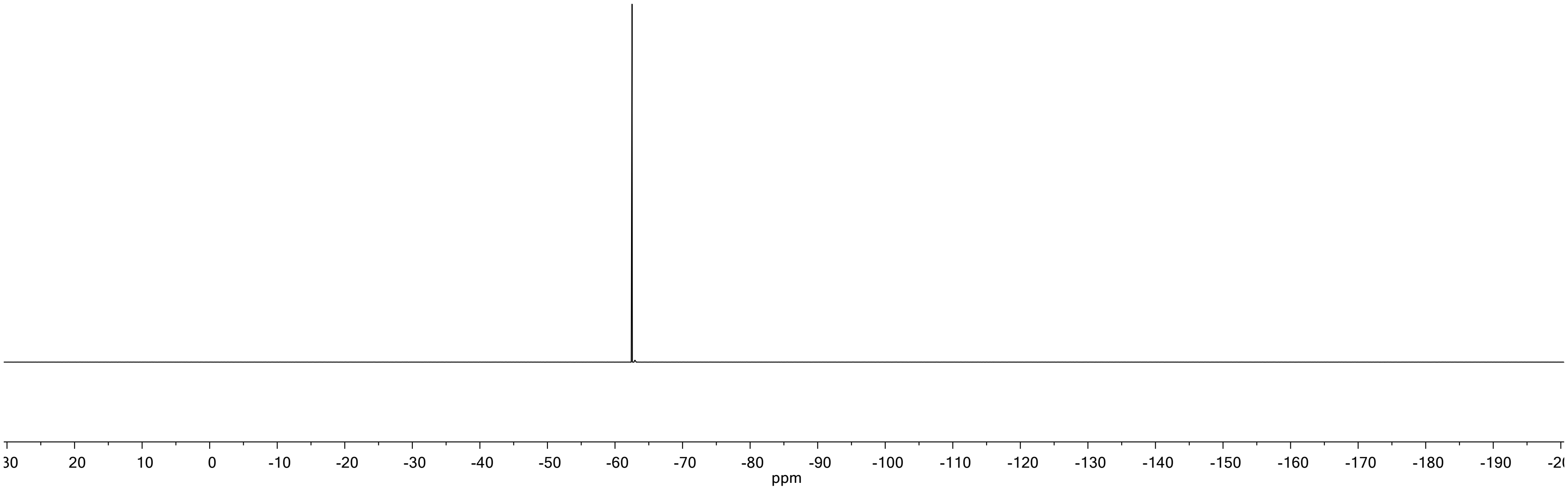

^{1}H NMR of 3-nitroaniline (2a)

$CDCl_3$, 23 ºC

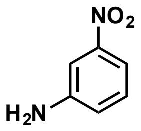

2a

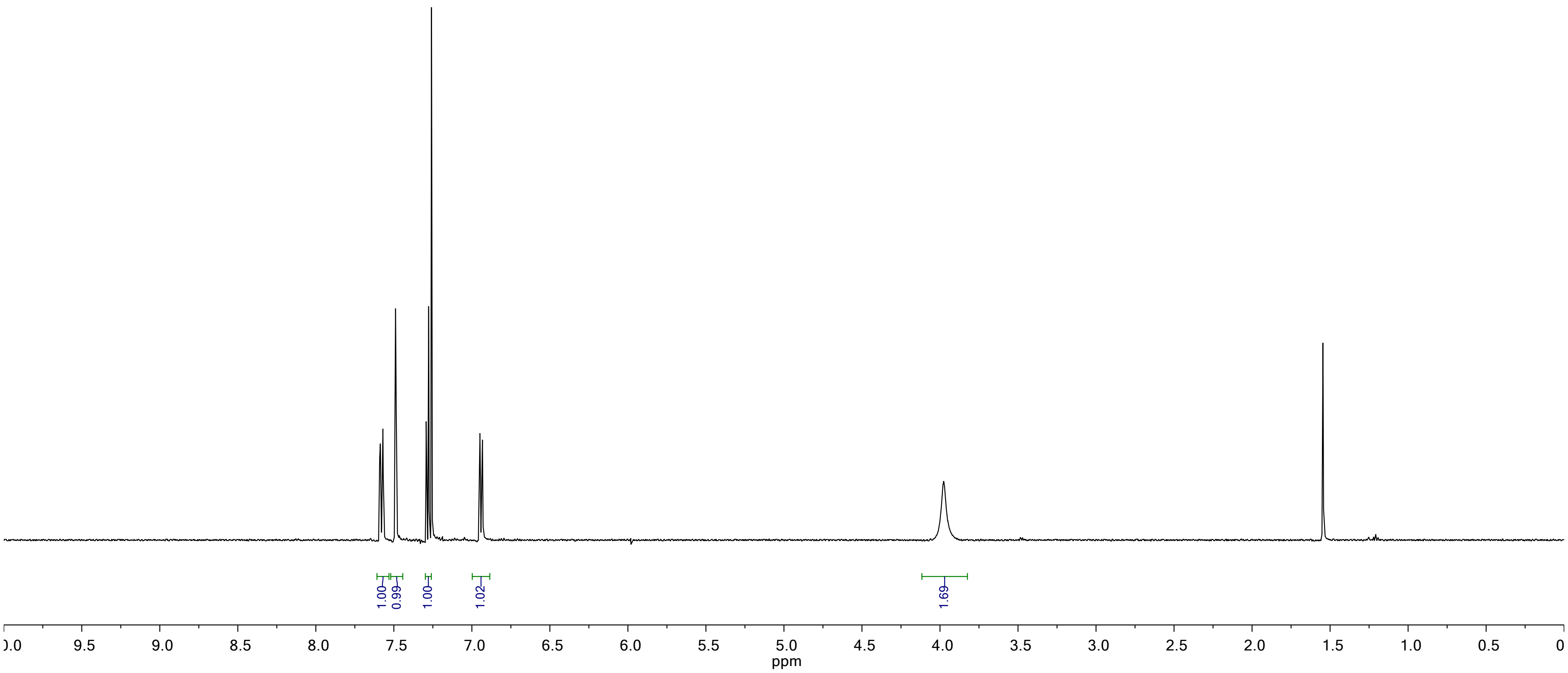

^{13}C NMR of 3-nitroaniline (2a)

$CDCl_3$, 23 ºC

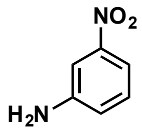

2a

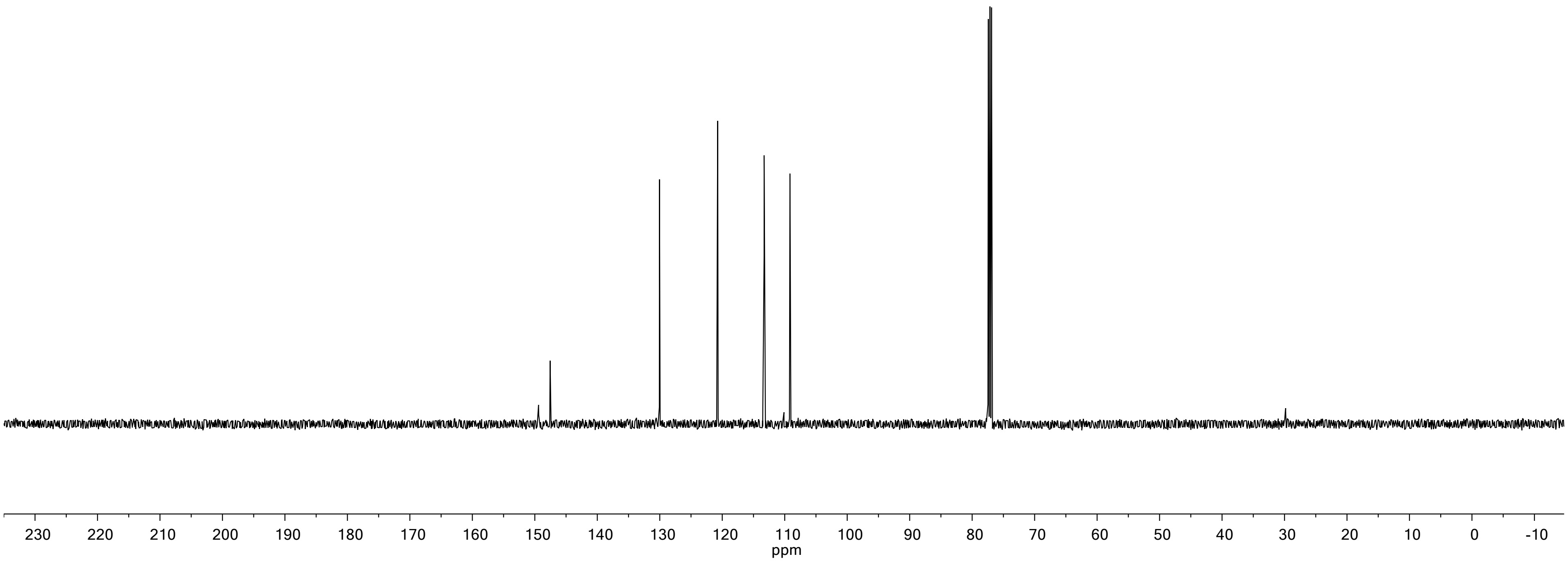

^{1}H NMR of 2-nitroaniline (2b)

$CDCl_3$, 23 ºC

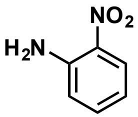

2b

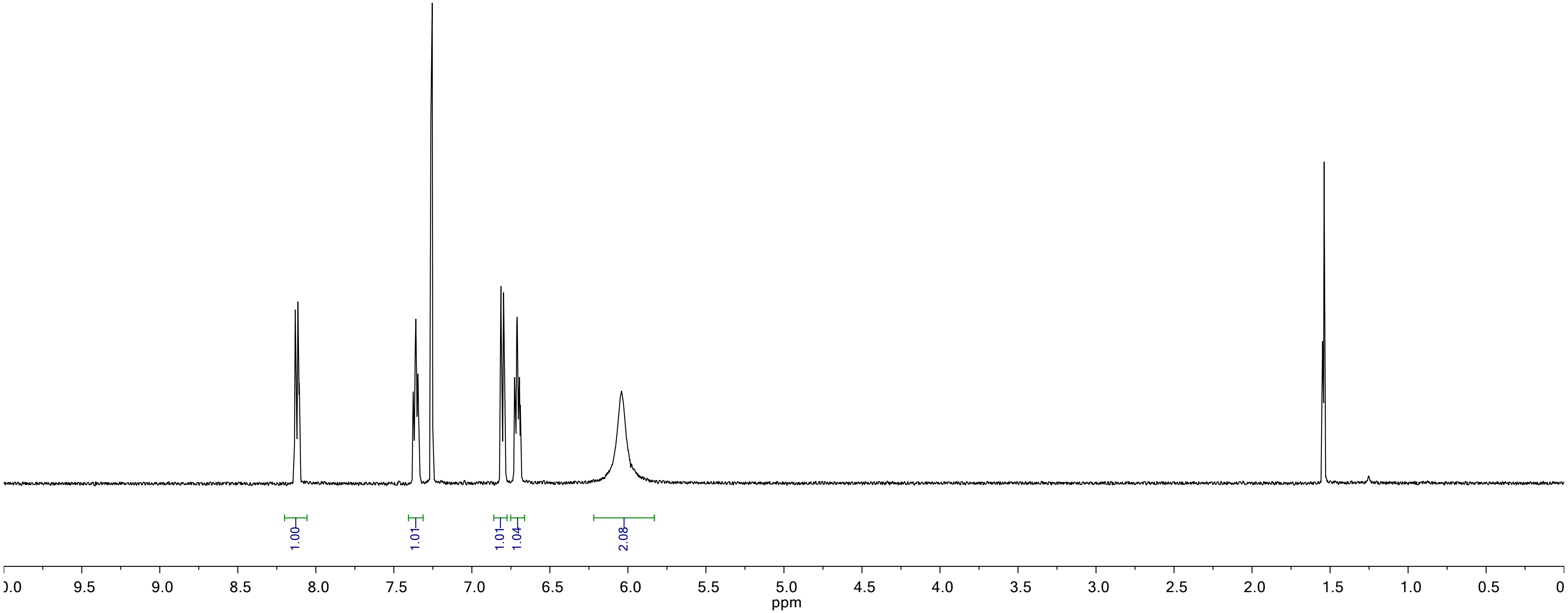

^{13}C NMR of 2-nitroaniline (2b)

$CDCl_3$, 23 ºC

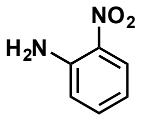

2b

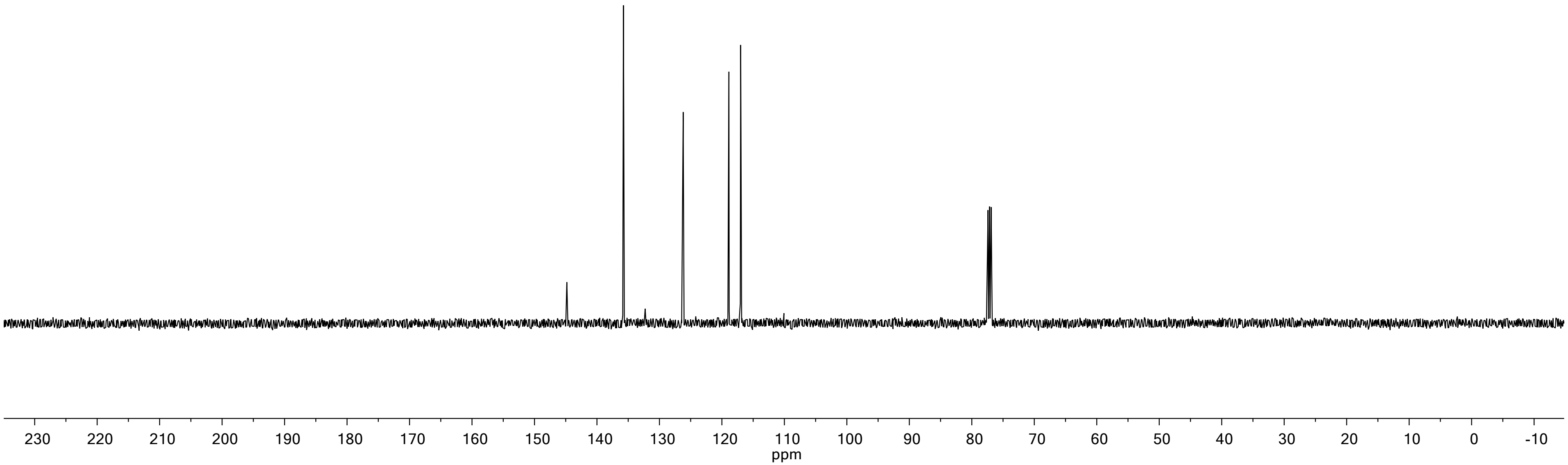

^{1}H NMR of 4-nitroaniline (2c)

$CDCl_3$, 23 ºC

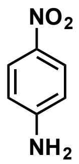

2c

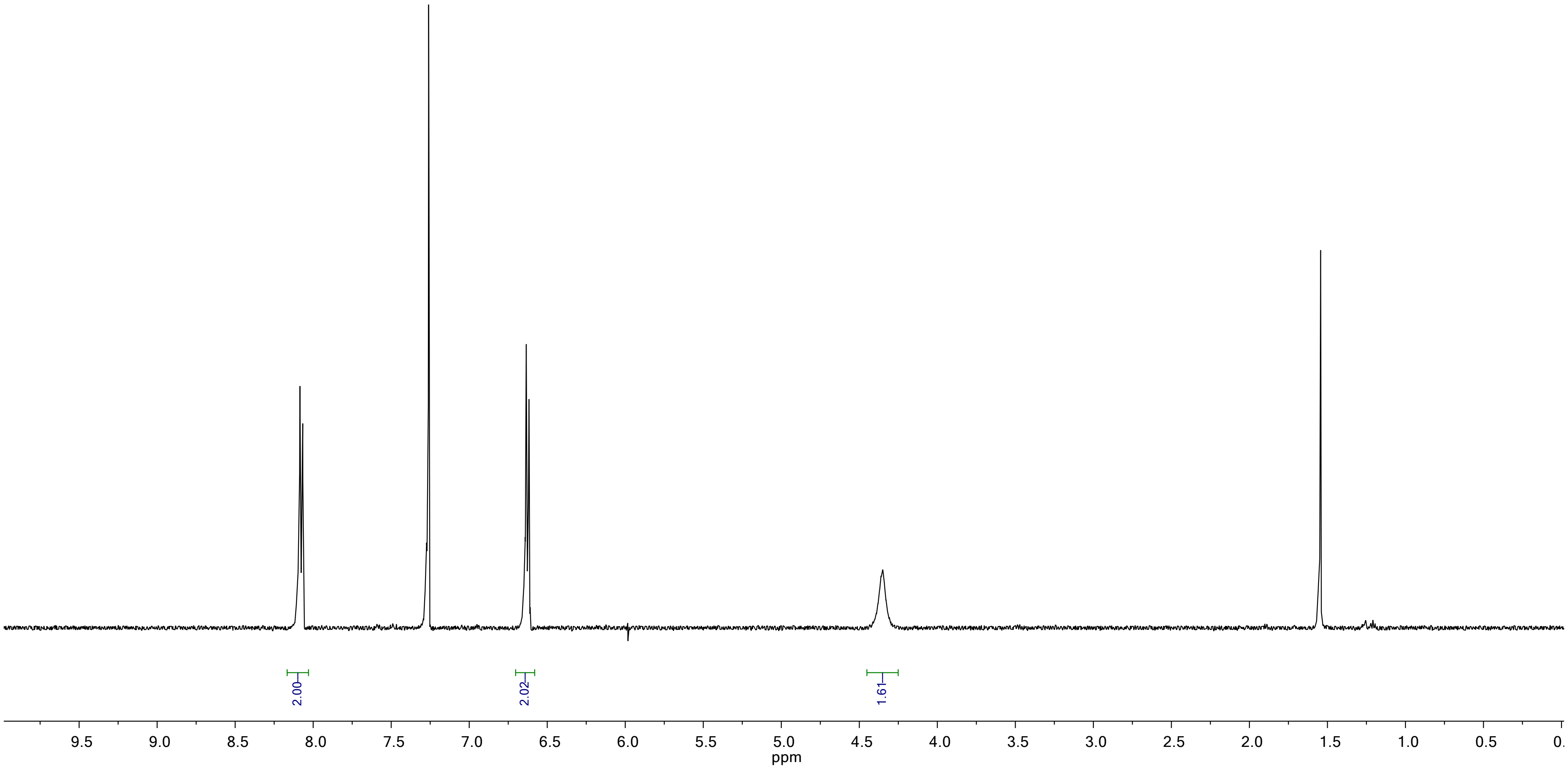

^{13}C NMR of 4-nitroaniline (2c)

$CDCl_3$, 23 ºC

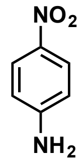

2c

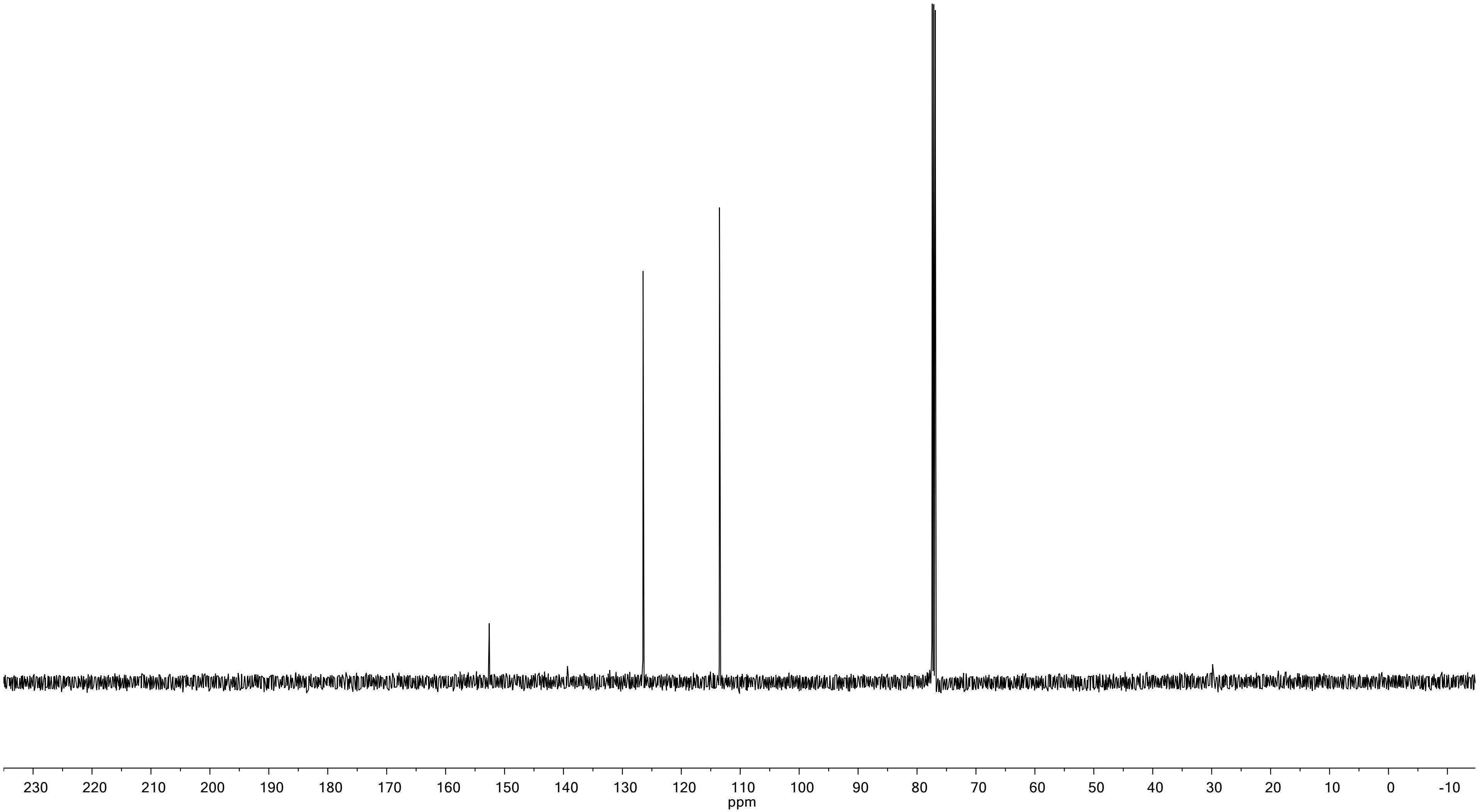

^{1}H NMR of 3-(methylsulfonyl)aniline (3a)

$CDCl_3$, 23 ºC

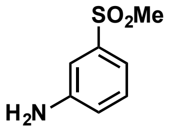

3a

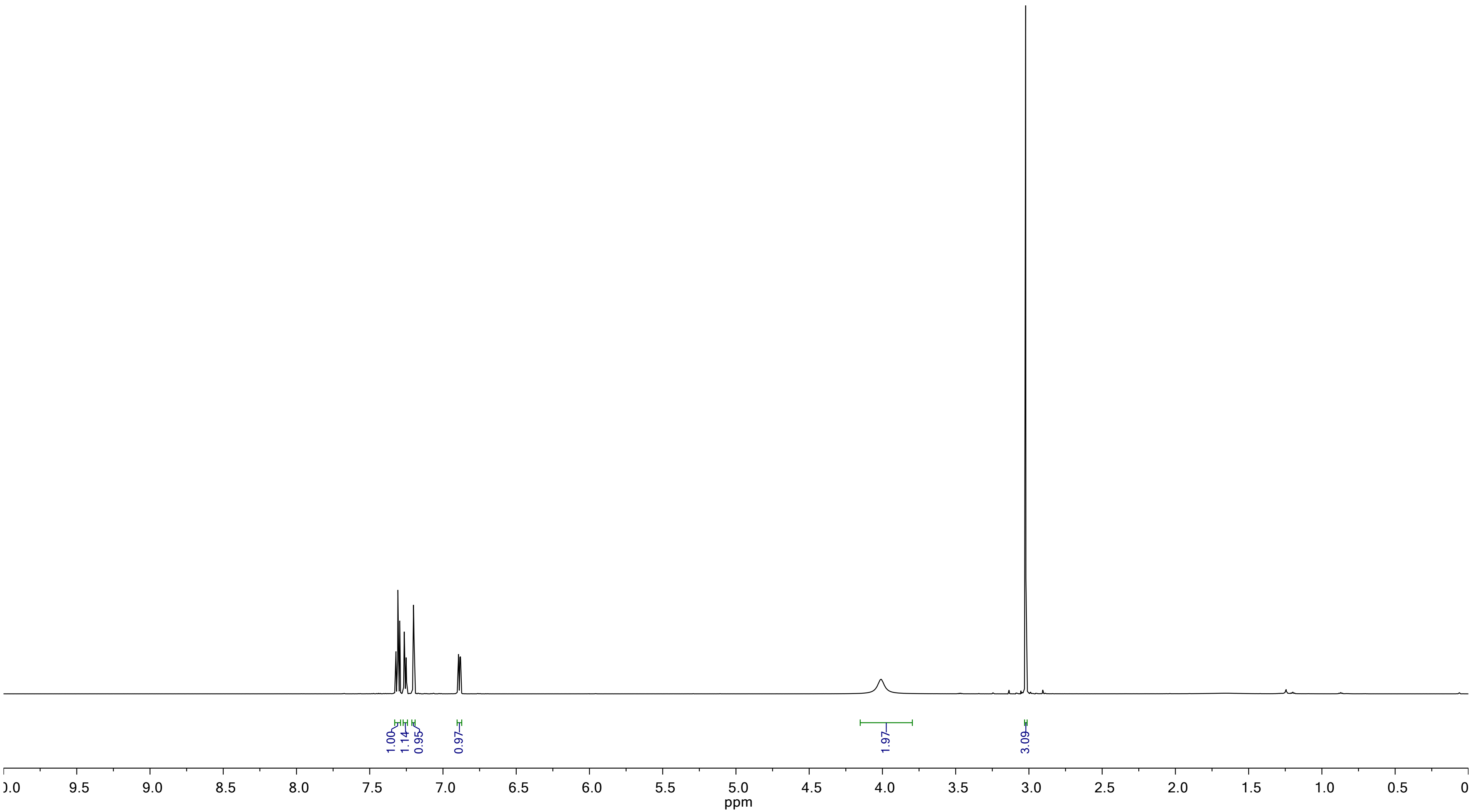

^{13}C NMR of 3-(methylsulfonyl)aniline (3a)

$CDCl_3$, 23 ºC

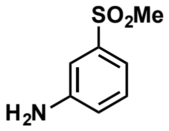

3a

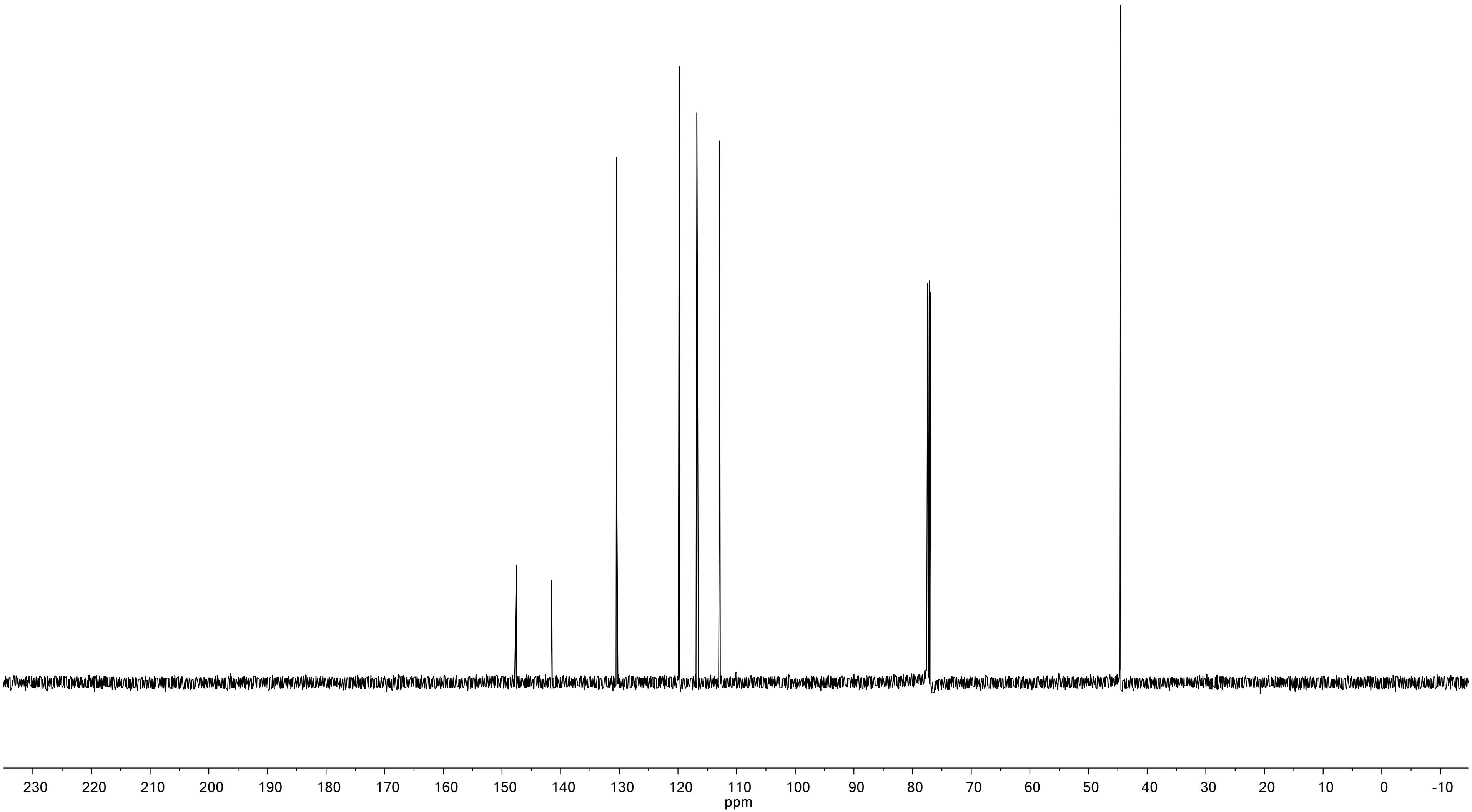

^{1}H NMR of 2-(methylsulfonyl)aniline (3b)

$CDCl_3$, 23 ºC

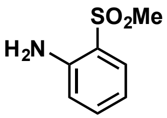

3b

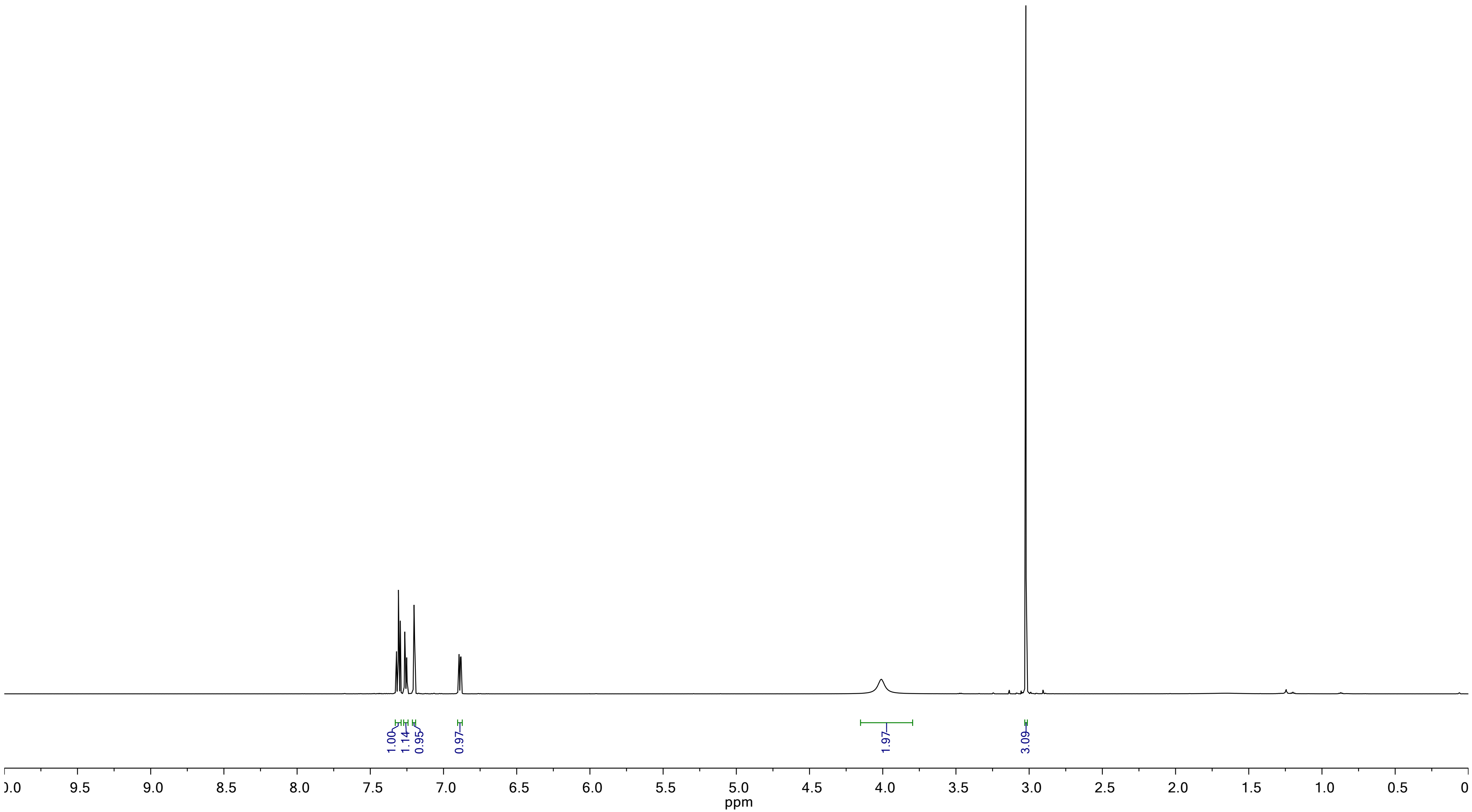

^{13}C NMR of 2-(methylsulfonyl)aniline (3b)

$CDCl_3$, 23 ºC

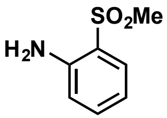

3b

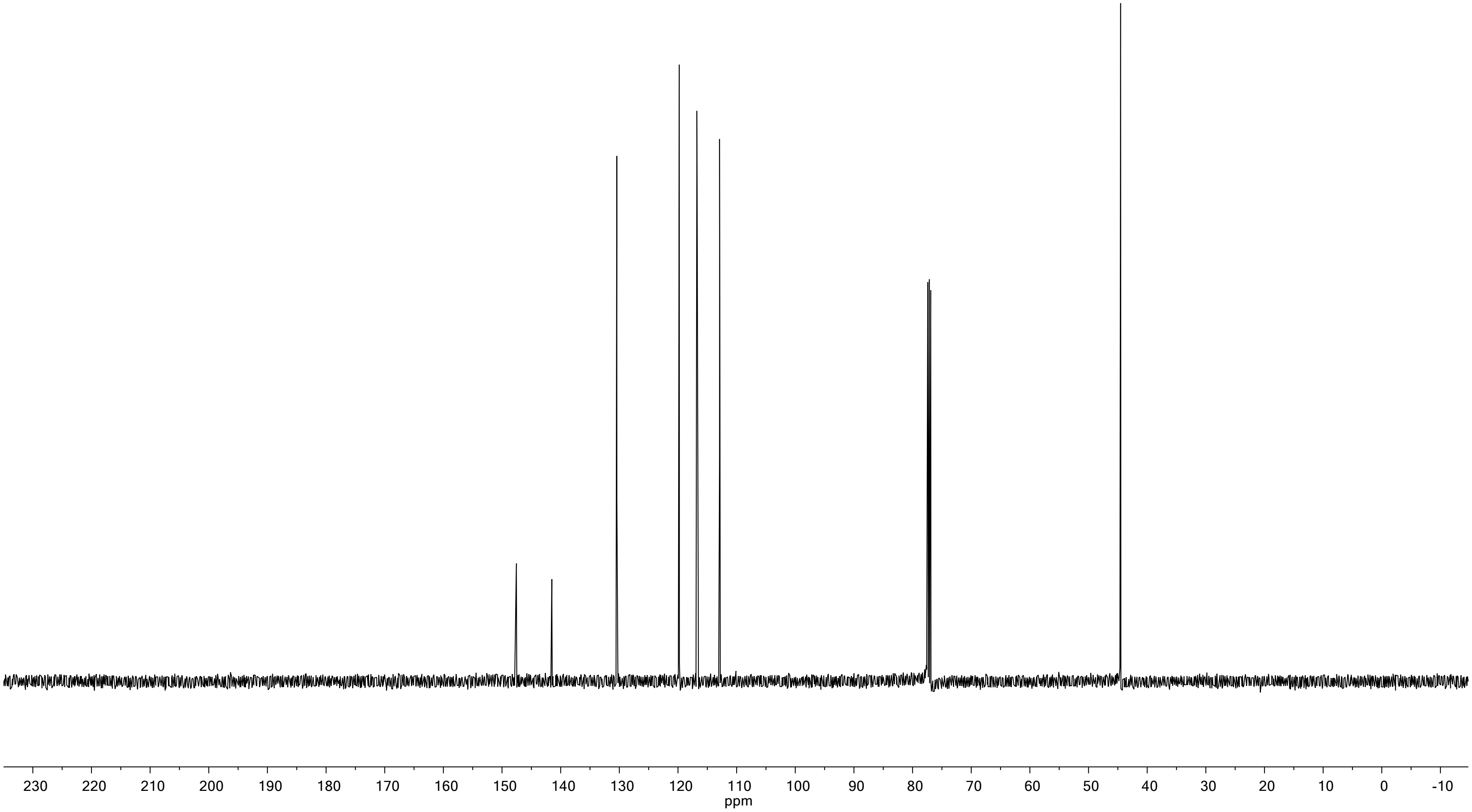

^{1}H NMR of 4-(methylsulfonyl)aniline (3c)

$CDCl_3$, 23 ºC

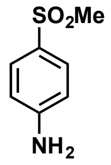

3c

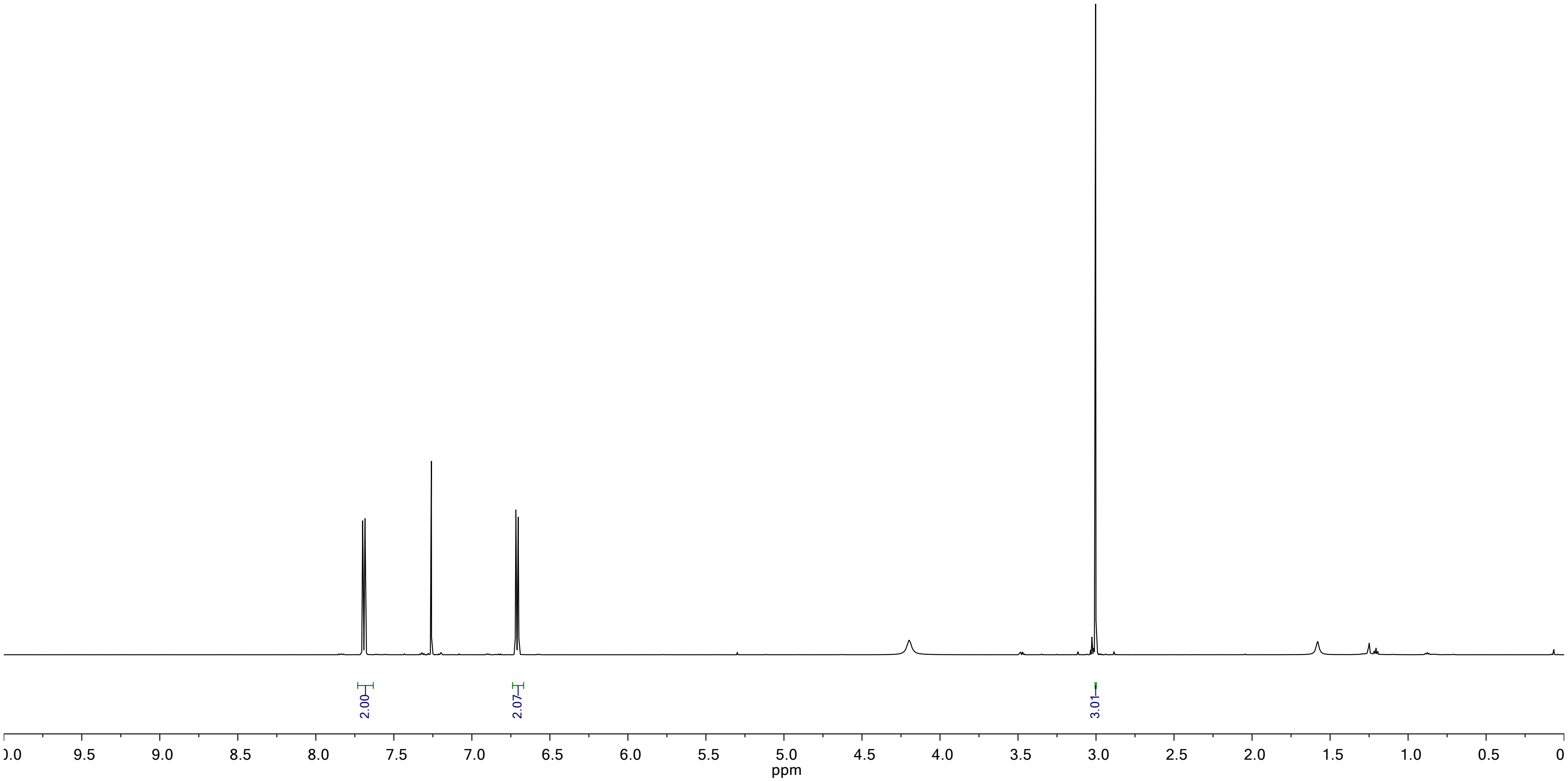

^{13}C NMR of 4-(methylsulfonyl)aniline (3c)

$CDCl_3$, 23 ºC

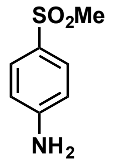

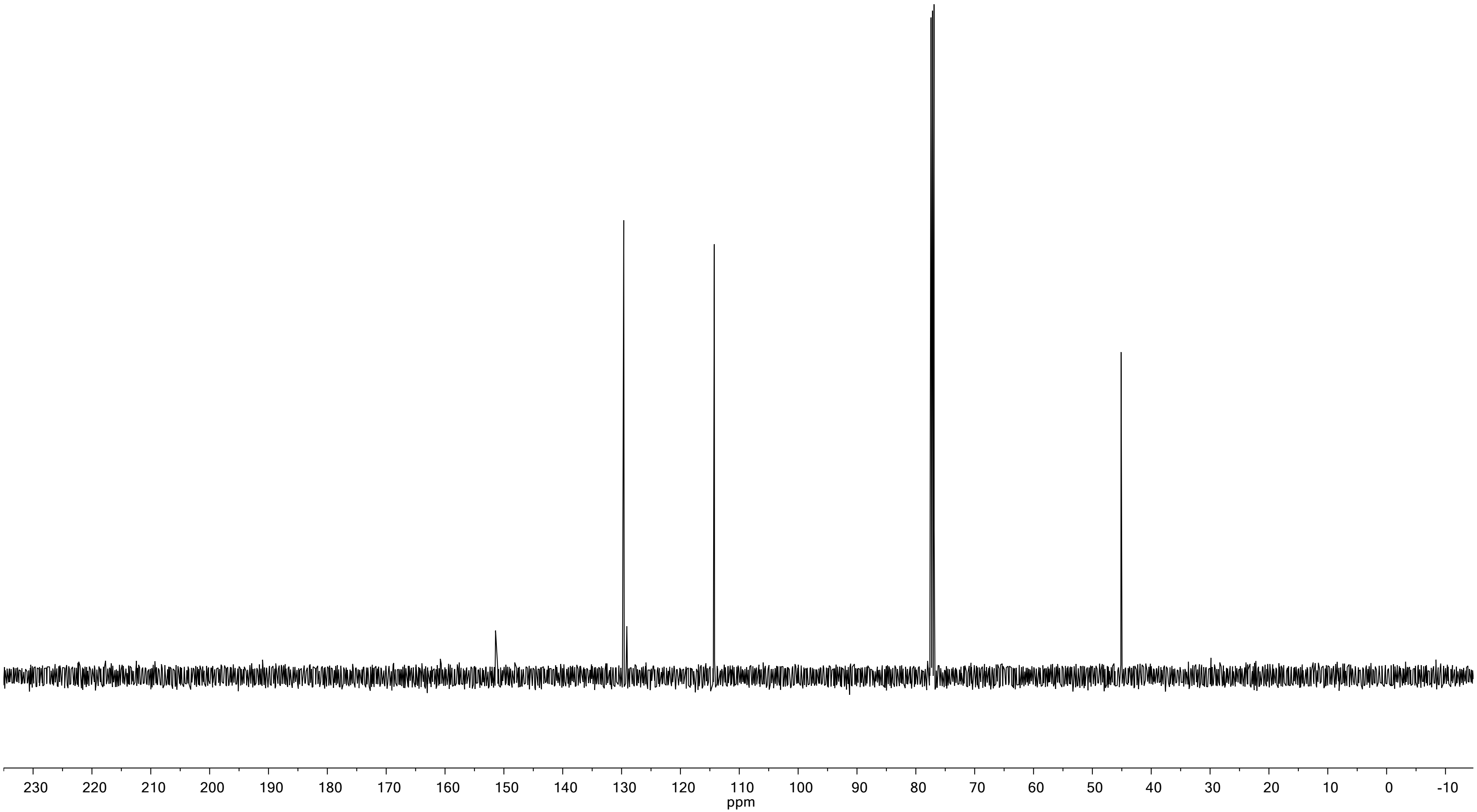

^{1}H NMR of 3-aminobenzonitrile (4a)

$CDCl_3$, 23 ºC

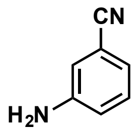

4a

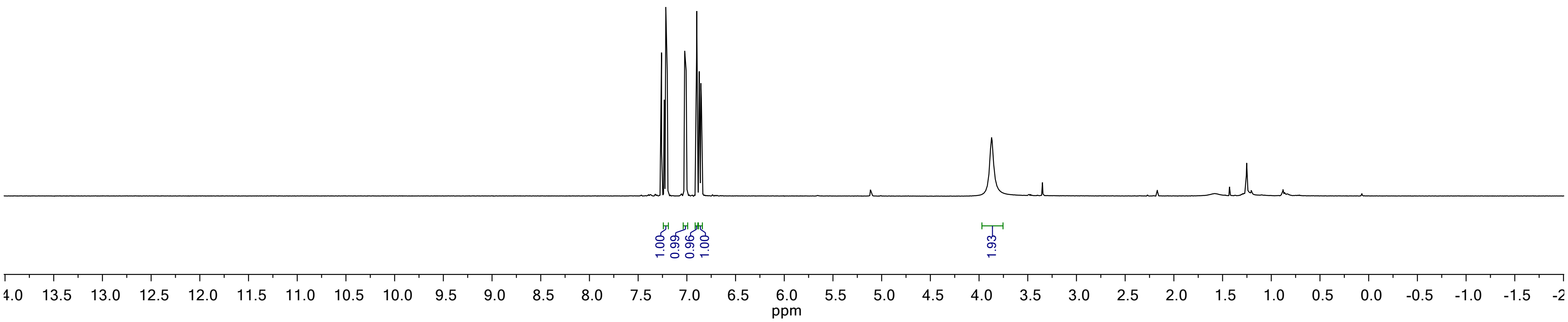

^{13}C NMR of 3-aminobenzonitrile (4a)

$CDCl_3$, 23 ºC

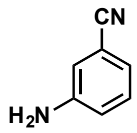

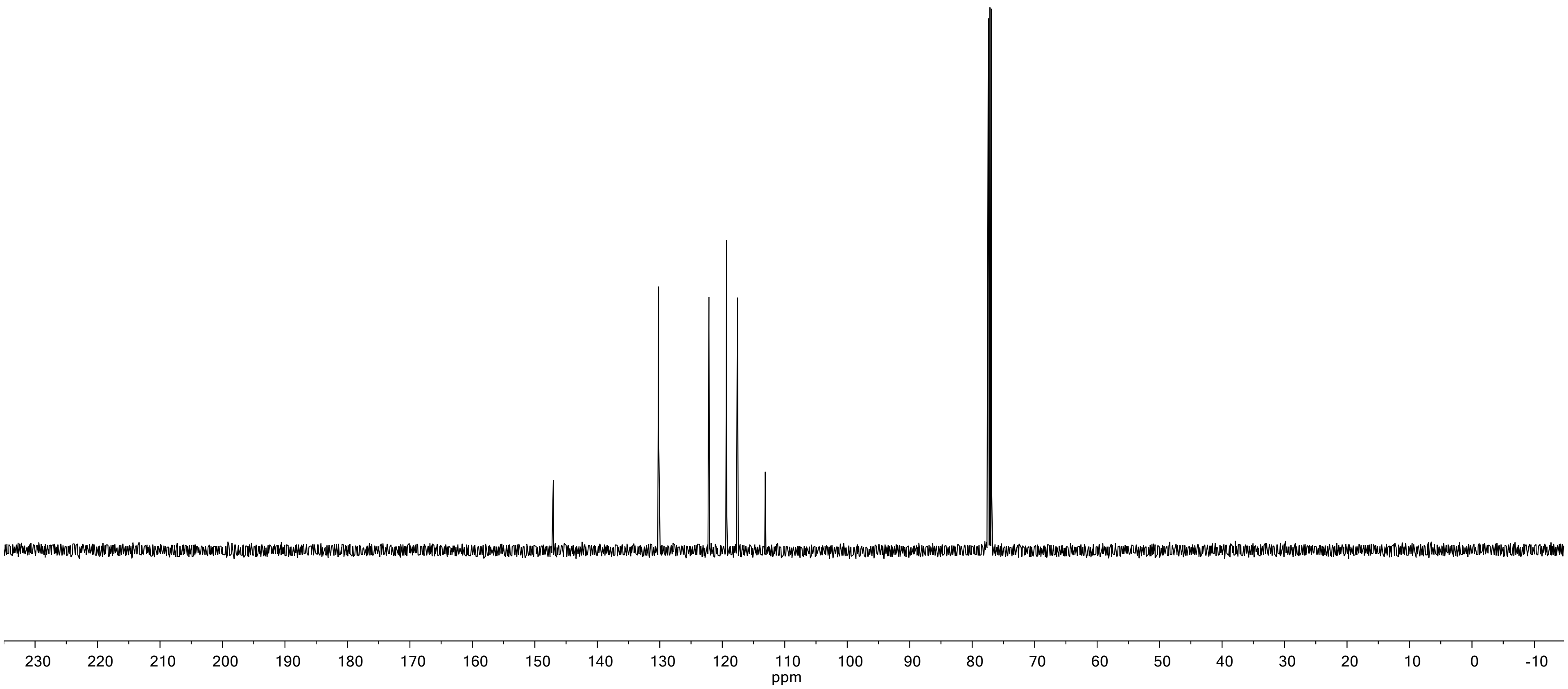

^{1}H NMR of 4-aminobenzonitrile (4b)

$CDCl_3$, 23 ºC

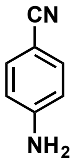

4b

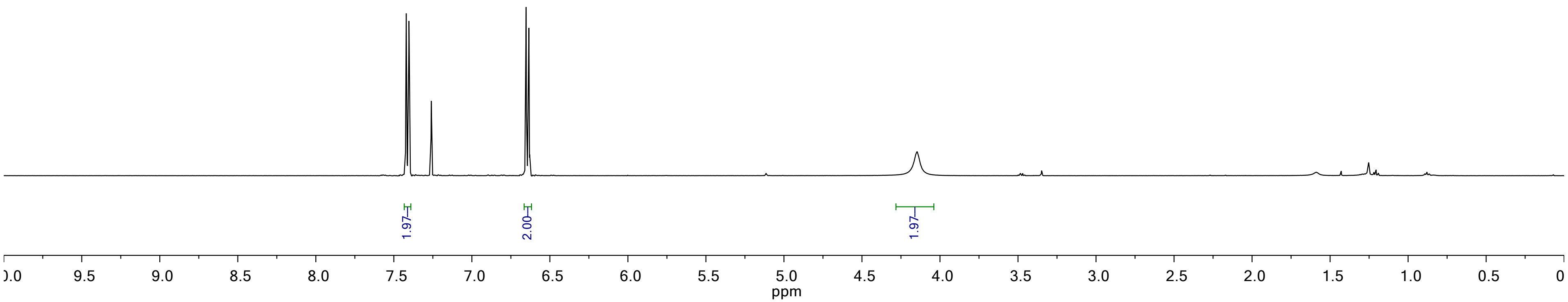

^{13}C NMR of 4-aminobenzonitrile (4b)

$CDCl_3$, 23 ºC

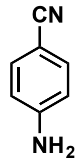

4b

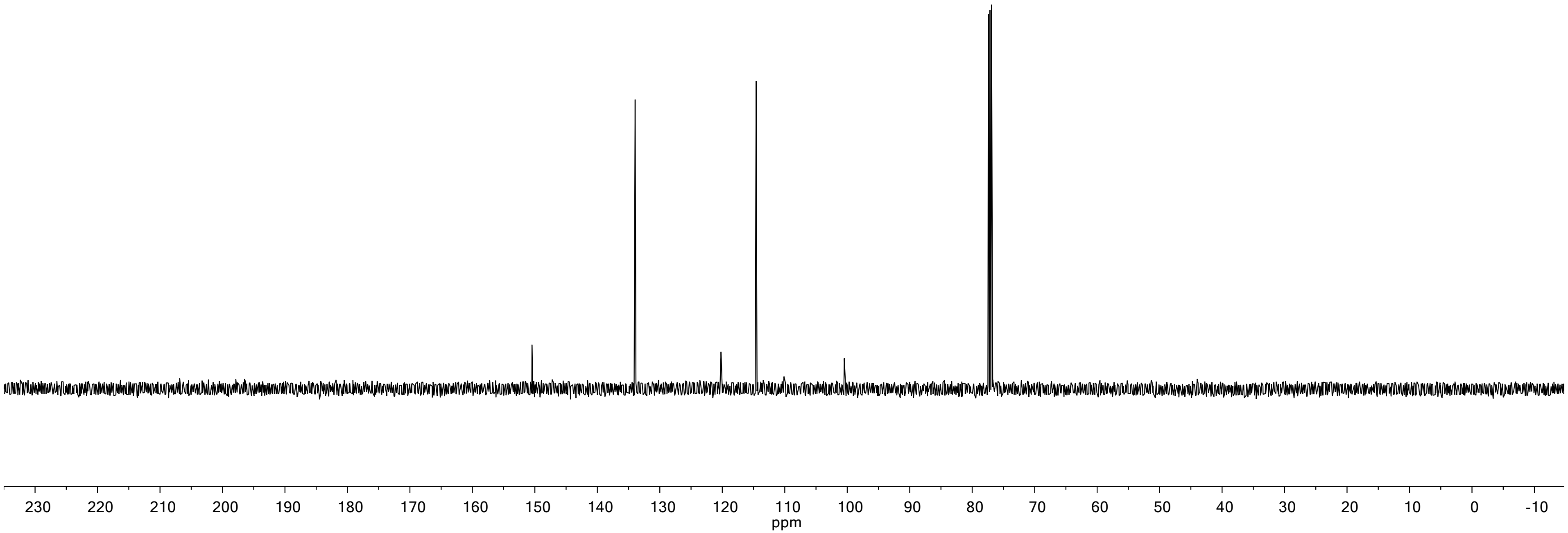

^{1}H NMR of 2-aminobenzonitrile (4c)

$CDCl_3$, 23 ºC

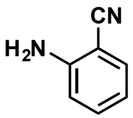

4c

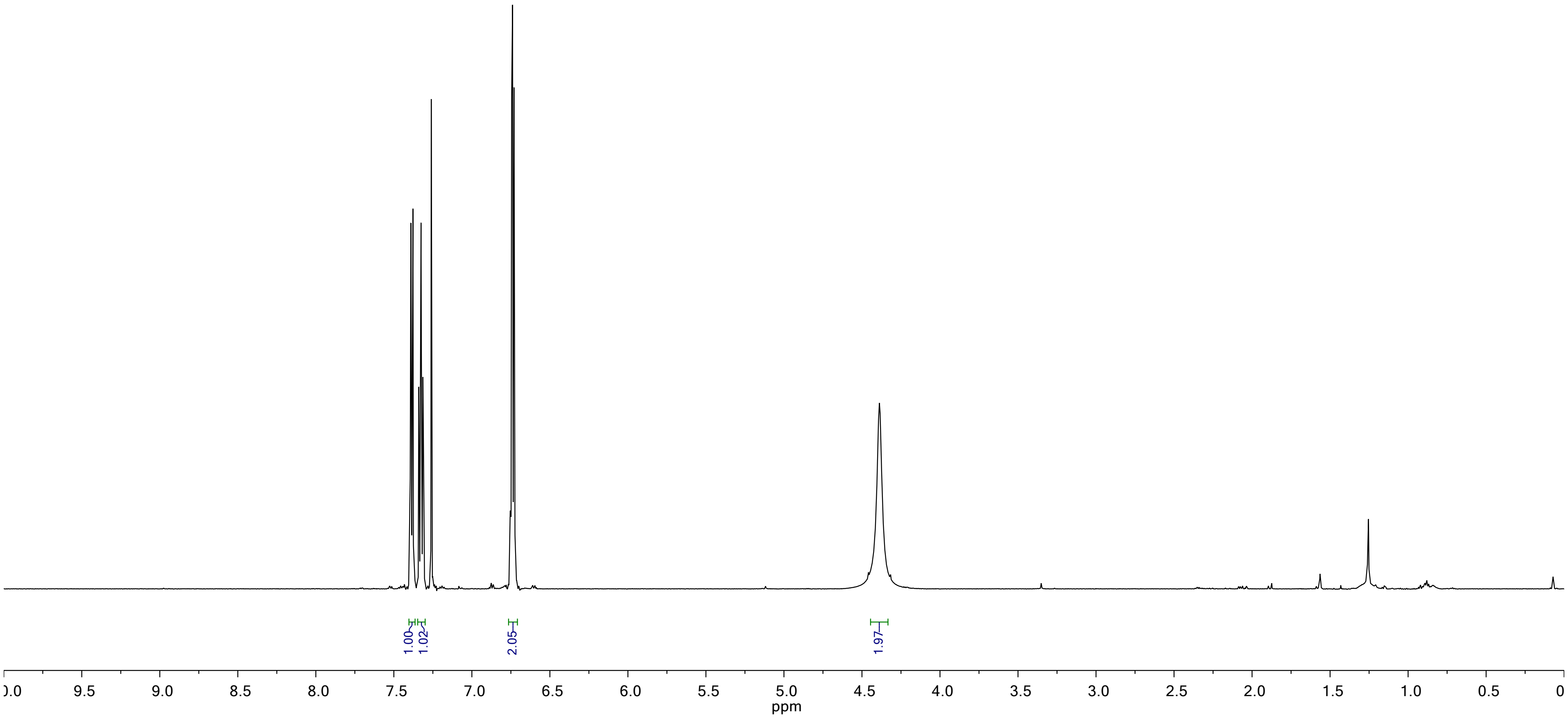

^{13}C NMR of 2-aminobenzonitrile (4c)

$CDCl_3$, 23 ºC

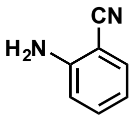

4c

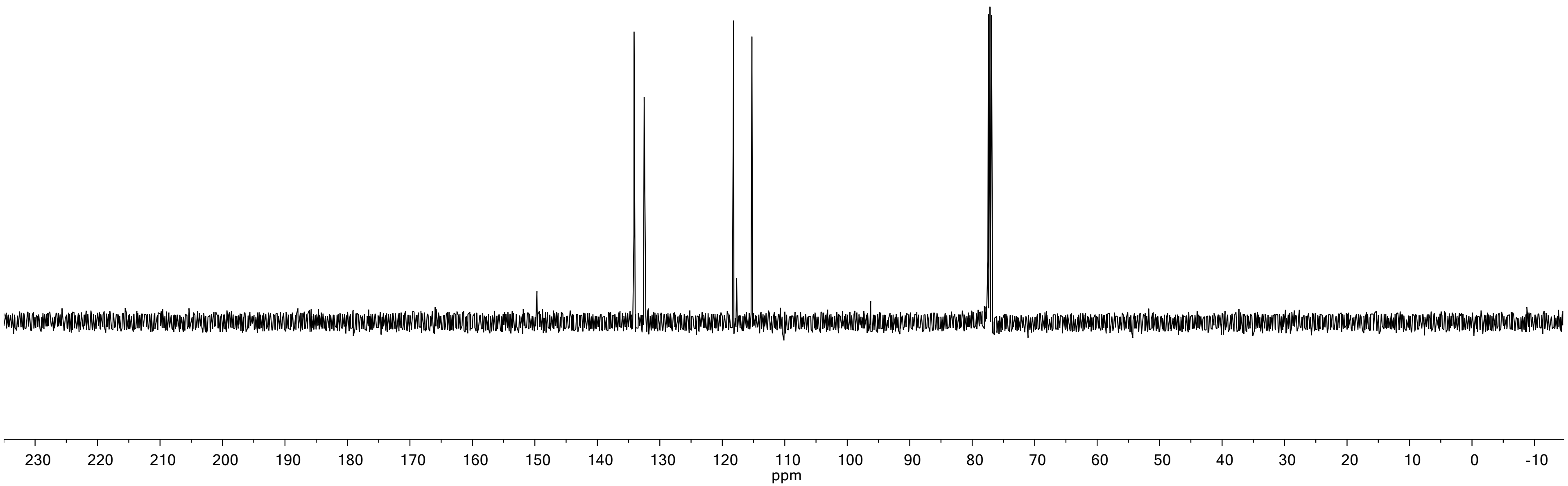

^{1}H NMR of 2,5-dibromoaniline (5)

$CDCl_3$, 23 ºC

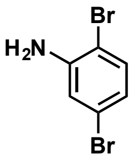

5

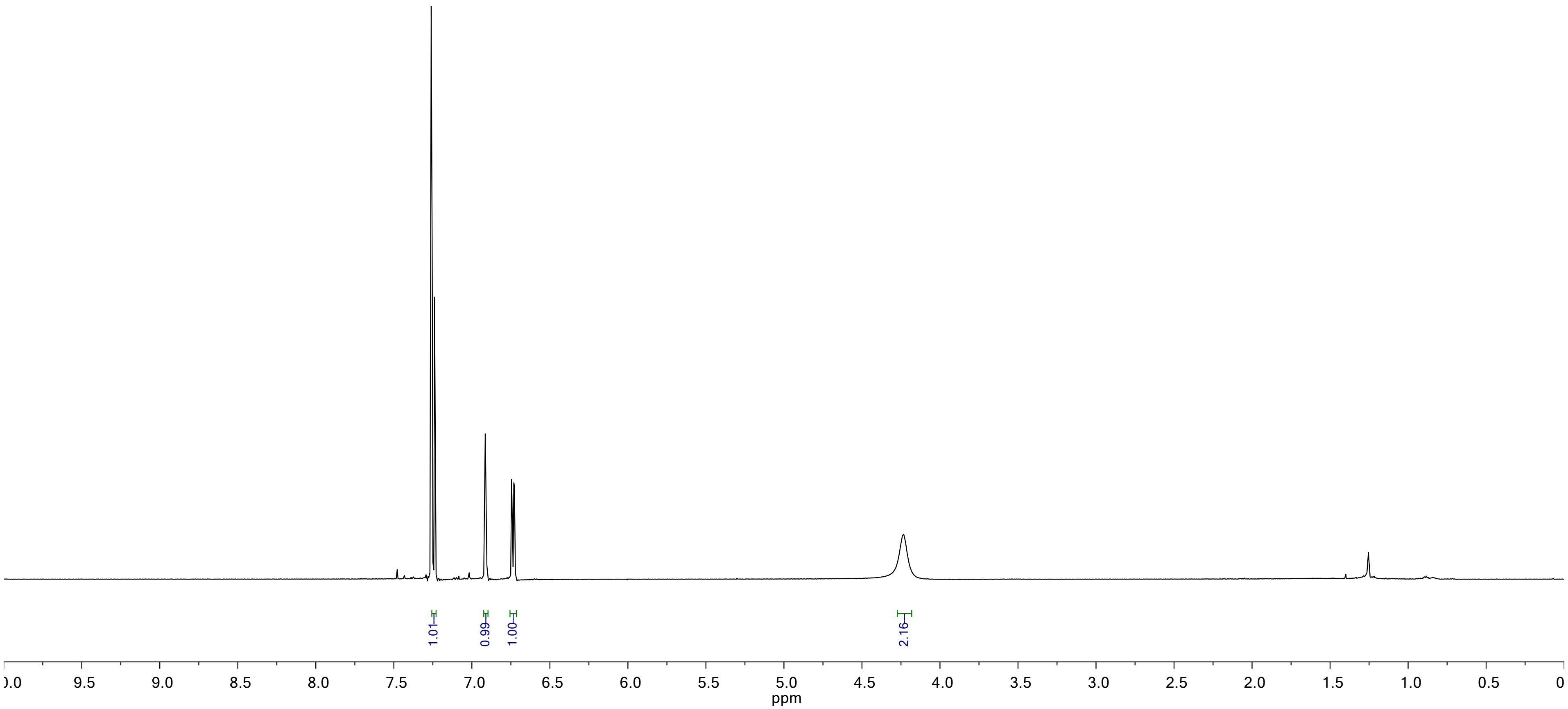

^{13}C NMR of 2,5-dibromoaniline (5)

$CDCl_3$, 23 ºC

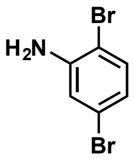

5

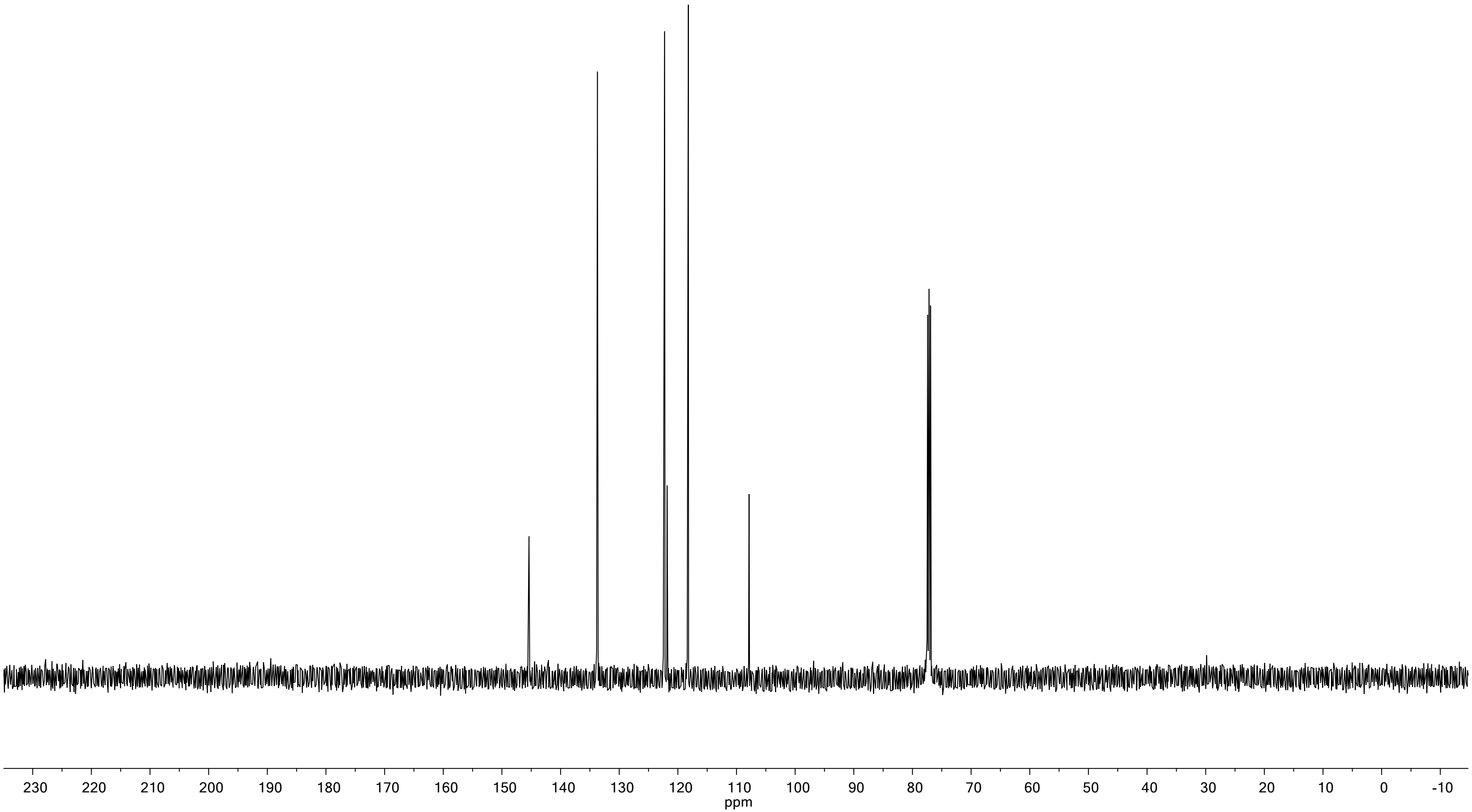

^{1}H NMR of ethyl 5-aminothiophene-2-carboxylate (6a)

CD_2Cl_2, 23 ºC

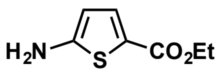

6a

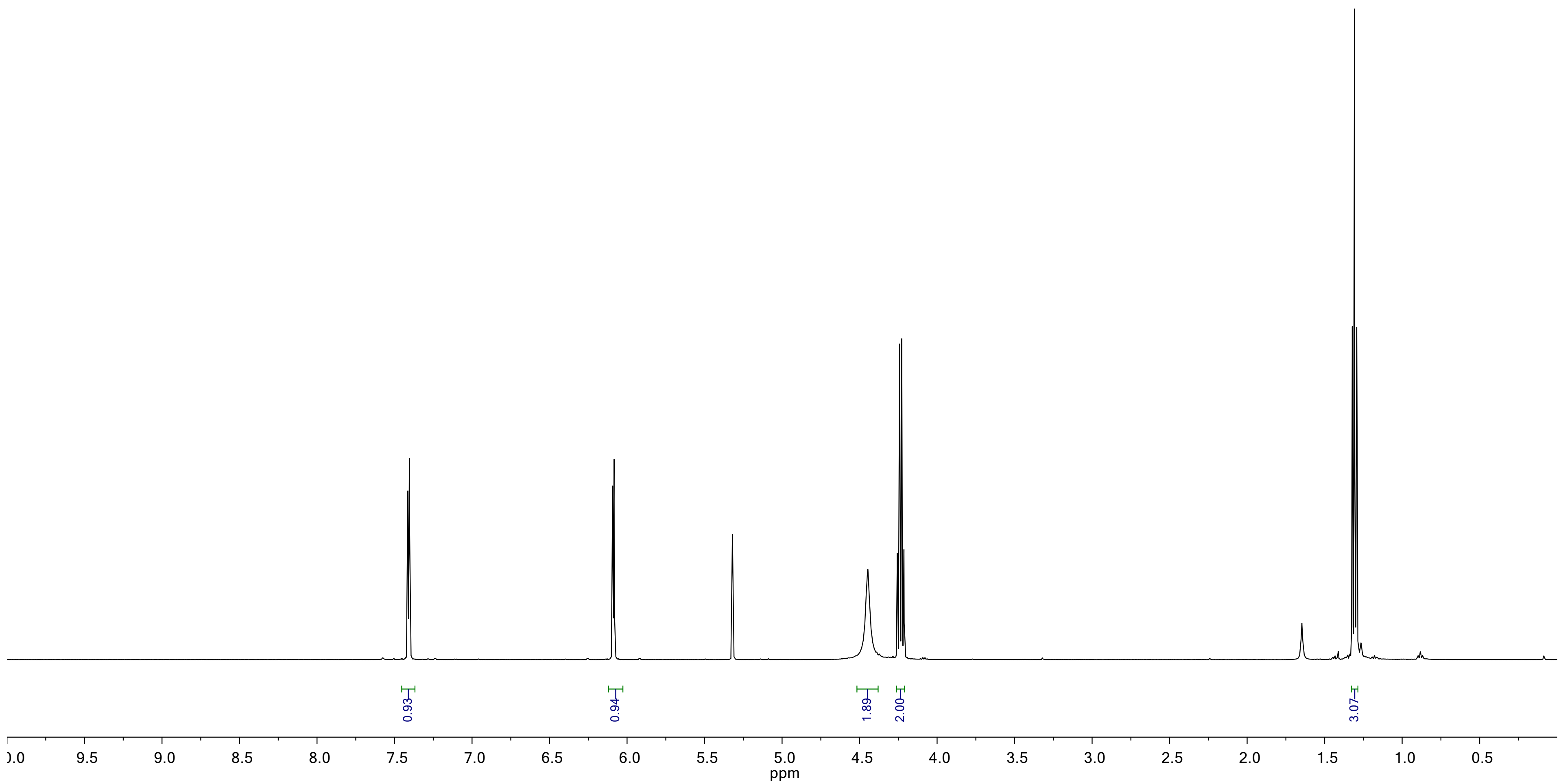

^{13}C NMR of ethyl 5-aminothiophene-2-carboxylate (6a)

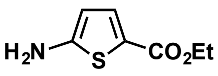

6a

CD_2Cl_2, 23 ºC

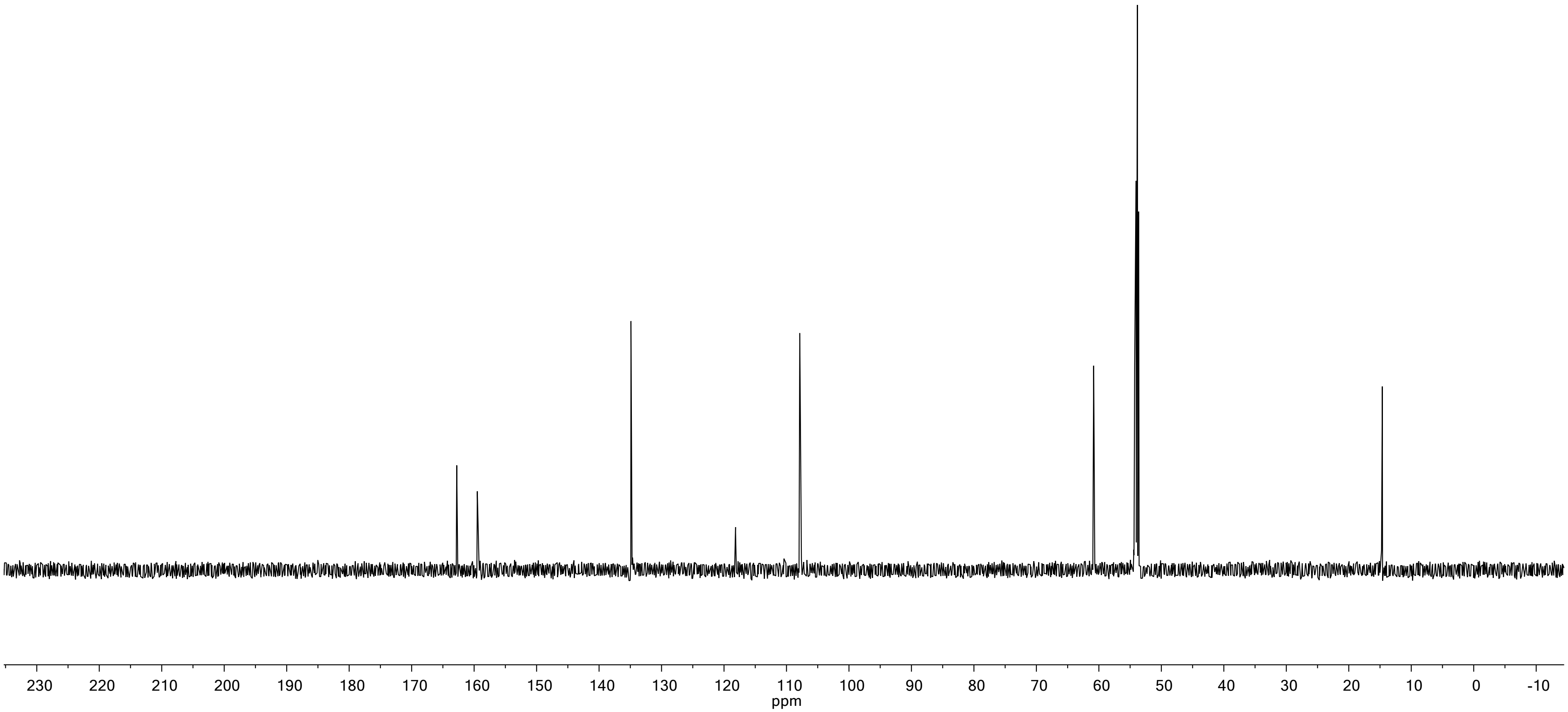

^{1}H NMR of ethyl 4-aminothiophene-2-carboxylate (6b)

CD_2Cl_2, 23 ºC

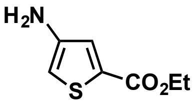

6b

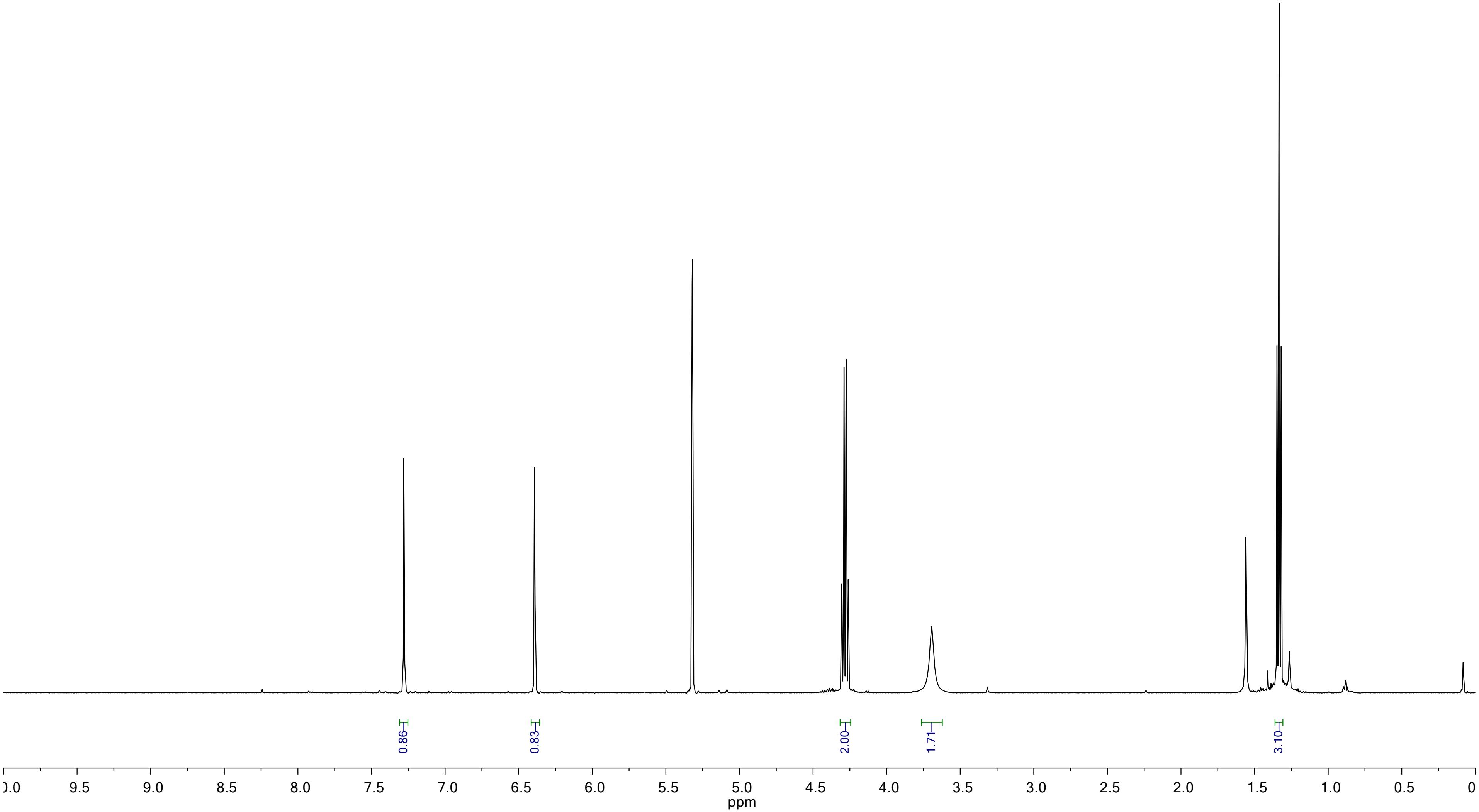

^{13}C NMR of ethyl 4-aminothiophene-2-carboxylate (6b)

CD_2Cl_2, 23 ºC

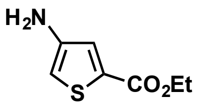

6b

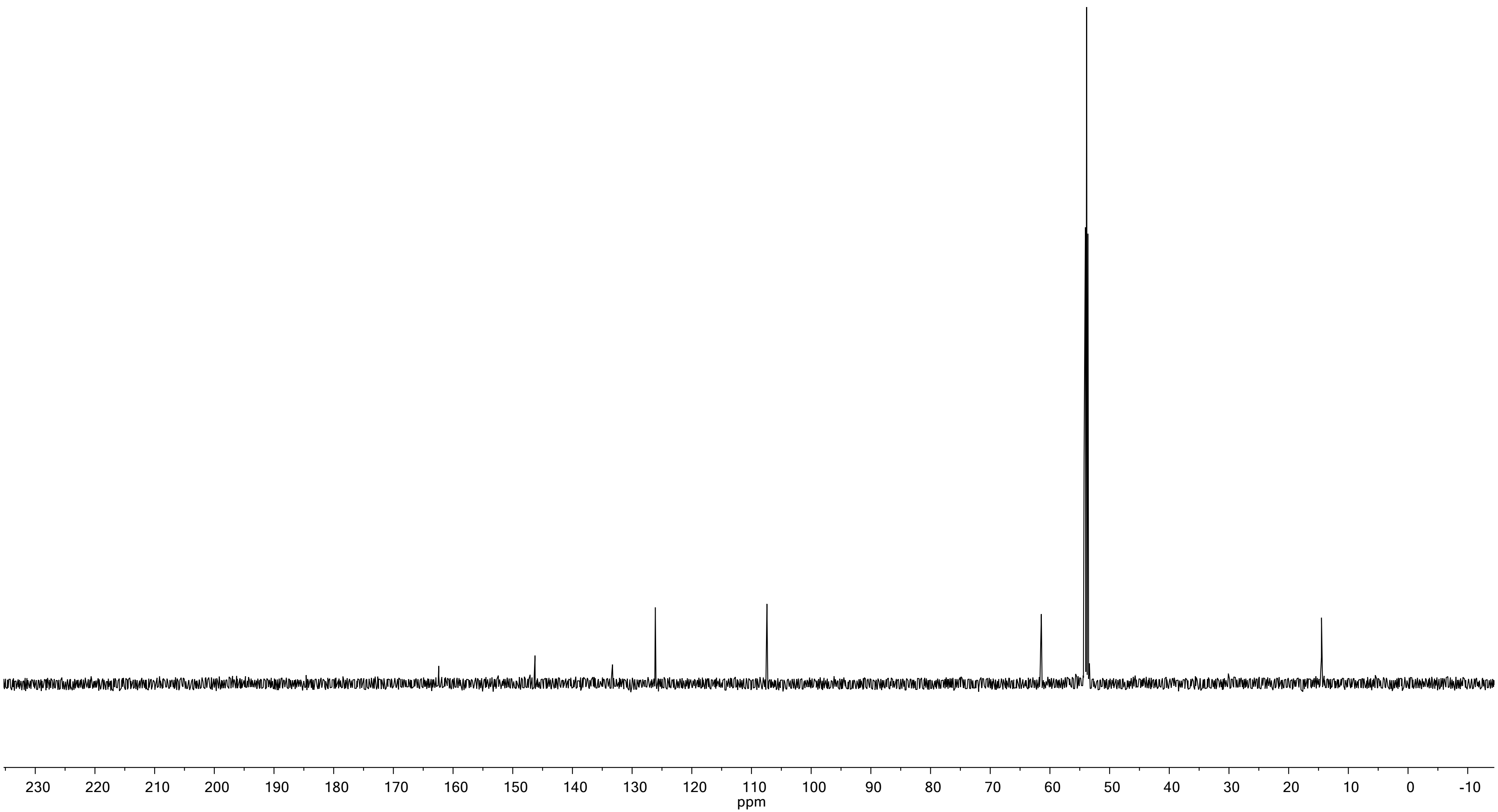

^{1}H NMR of ethyl 3-aminothiophene-2-carboxylate (6c)

CD_2Cl_2, 23 ºC

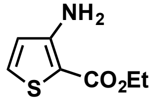

6c

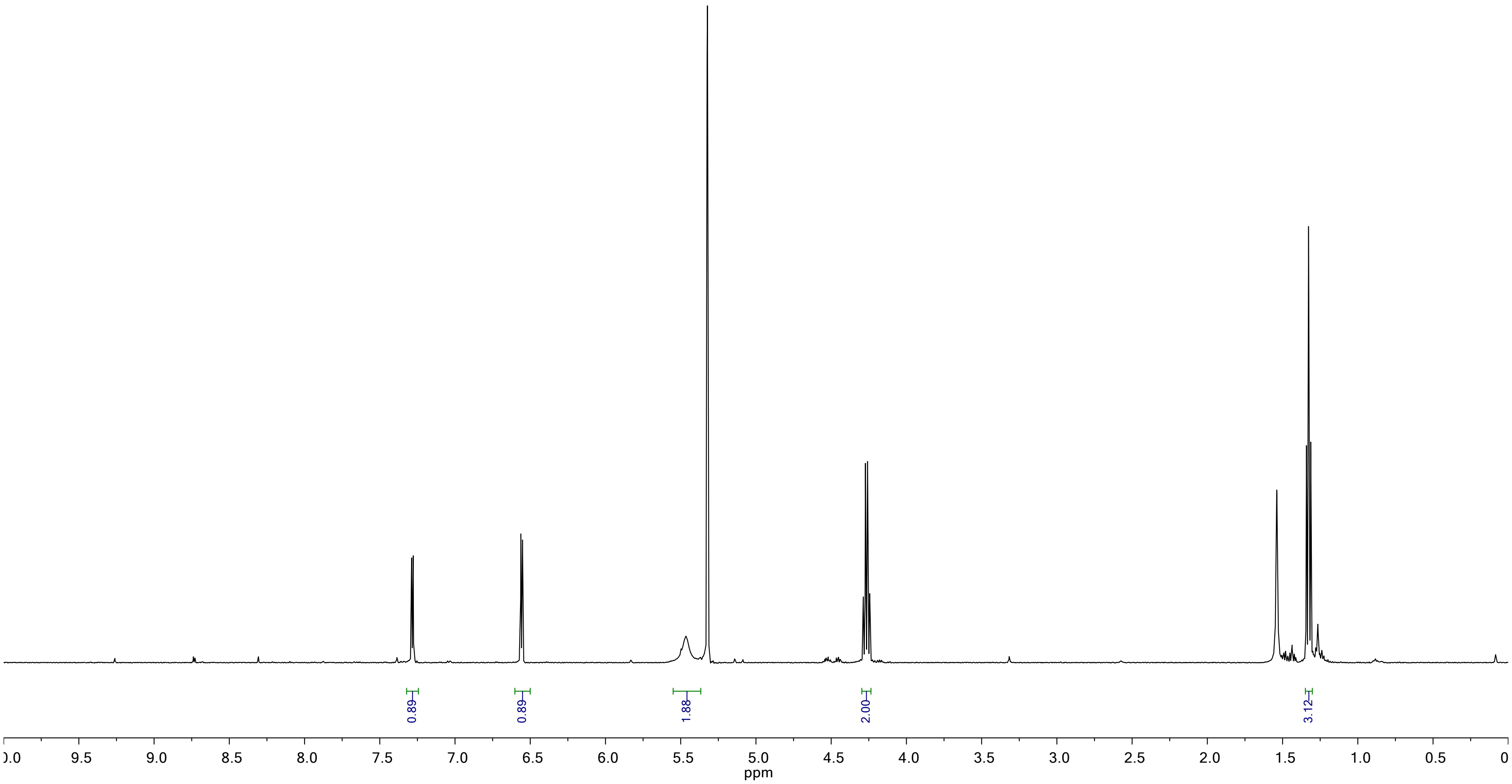

^{13}C NMR of ethyl 3-aminothiophene-2-carboxylate (6c)

CD_2Cl_2, 23 ºC

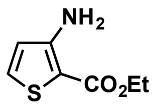

6c

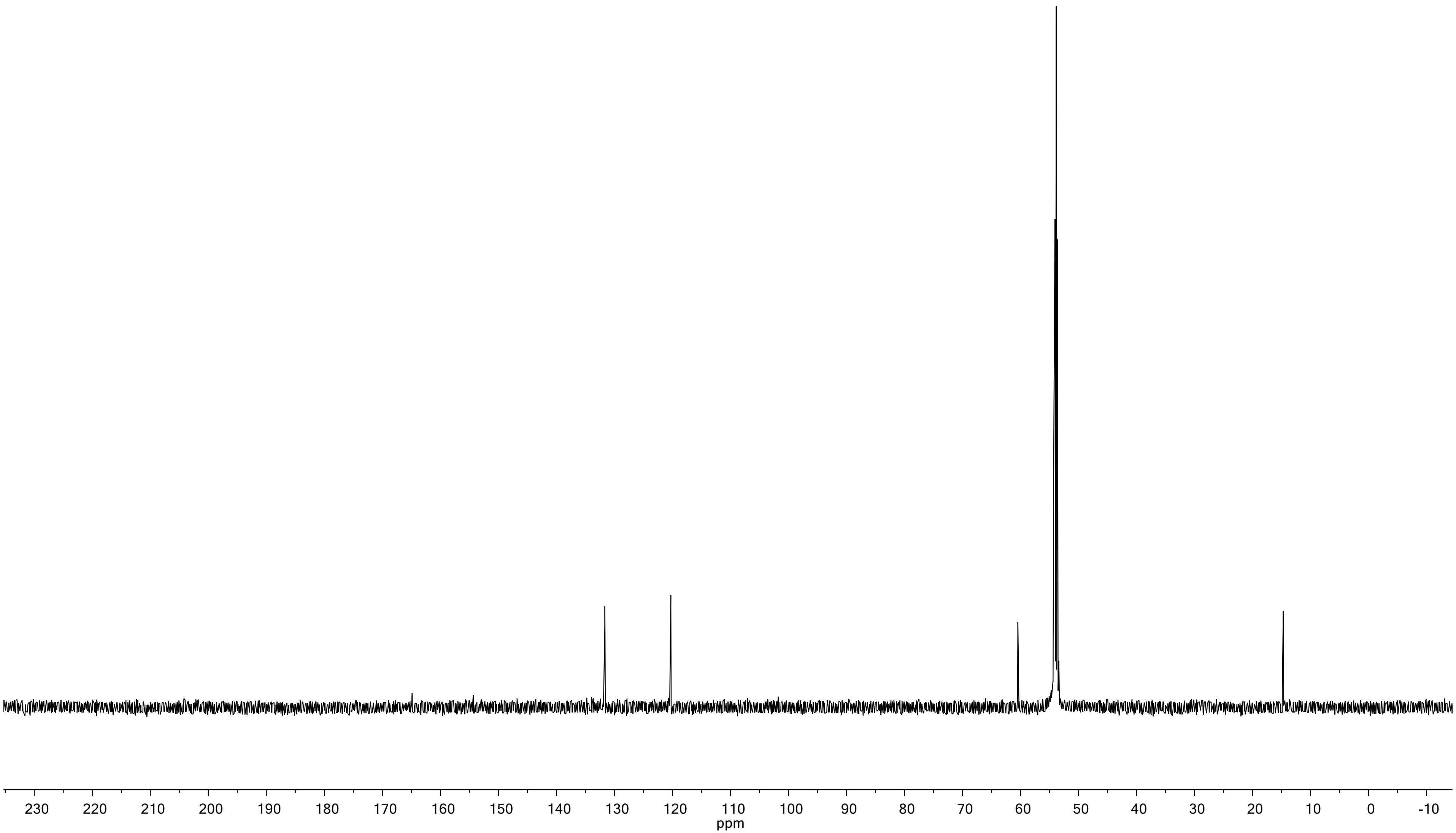

^{1}H NMR of 5-amino-6-methoxy-2-methylquinoline (7a)

$CDCl_3$, 23 ºC

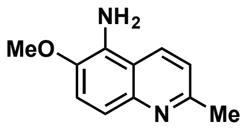

7a

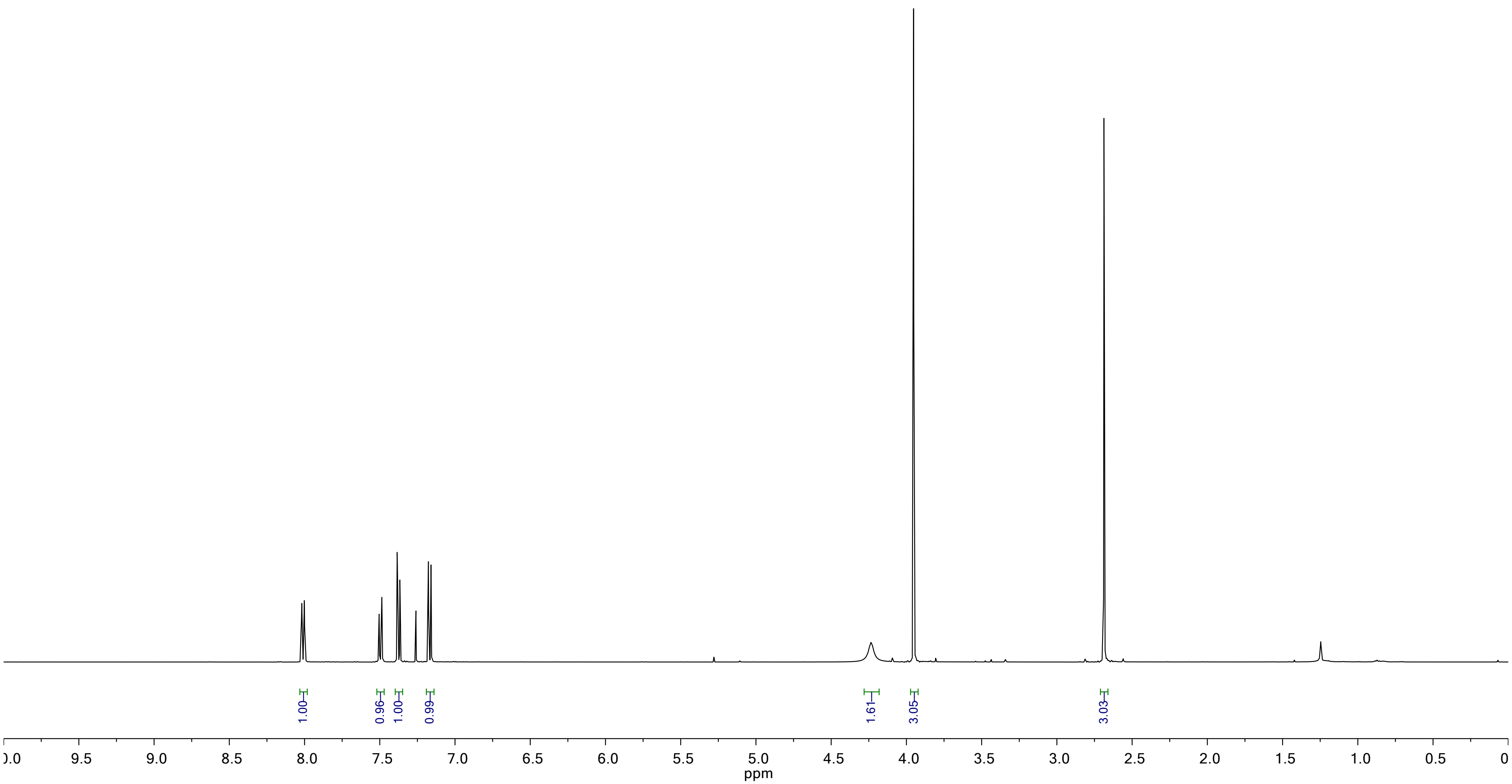

^{13}C NMR of 5-amino-6-methoxy-2-methylquinoline (7a)

$(CD_3)_2CO$, 23 ºC

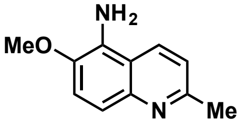

7a

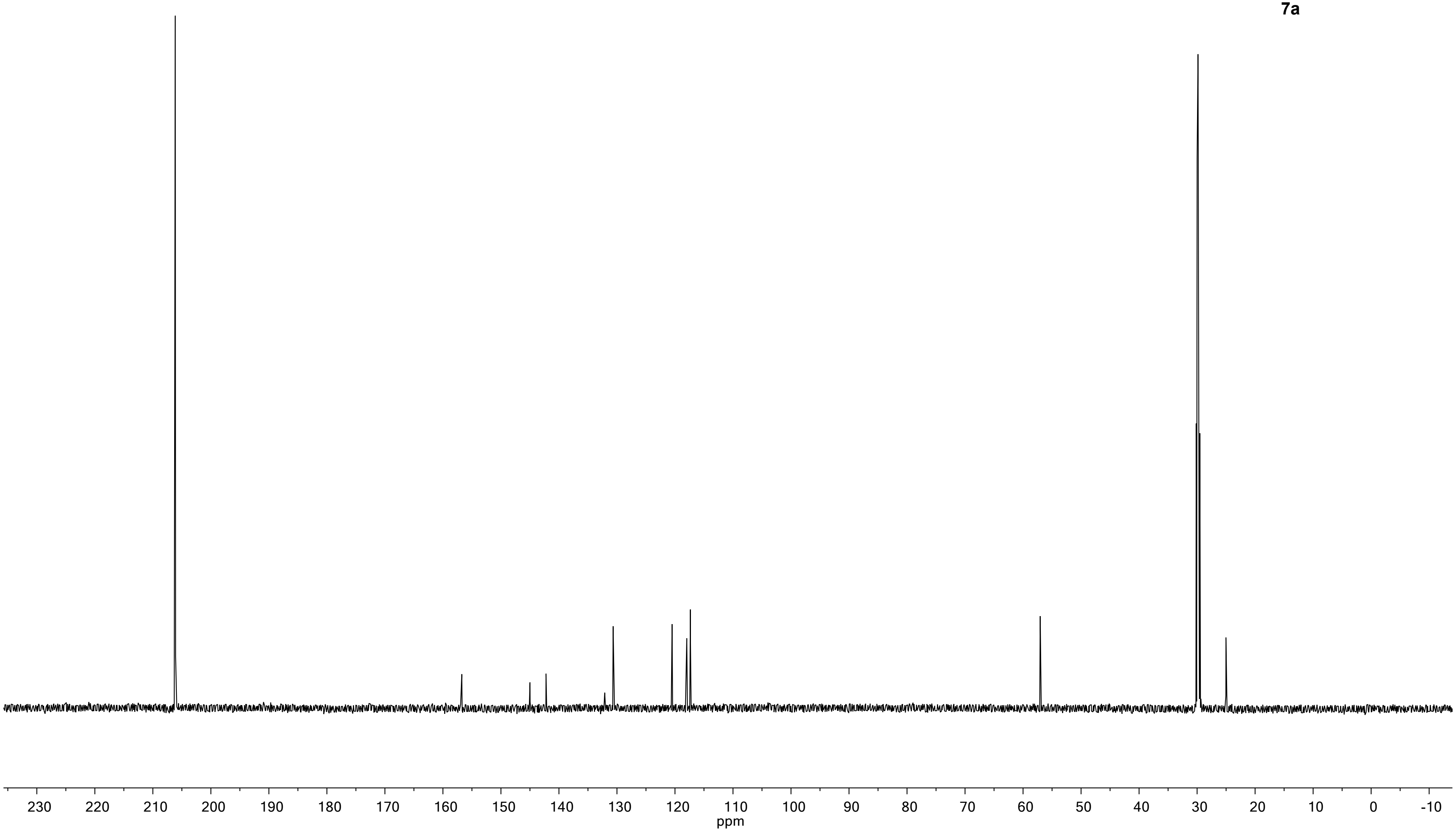

^{1}H NMR of 8-amino-6-methoxy-2-methylquinoline (7b)

$CDCl_3$, 23 ºC

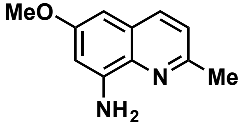

7b

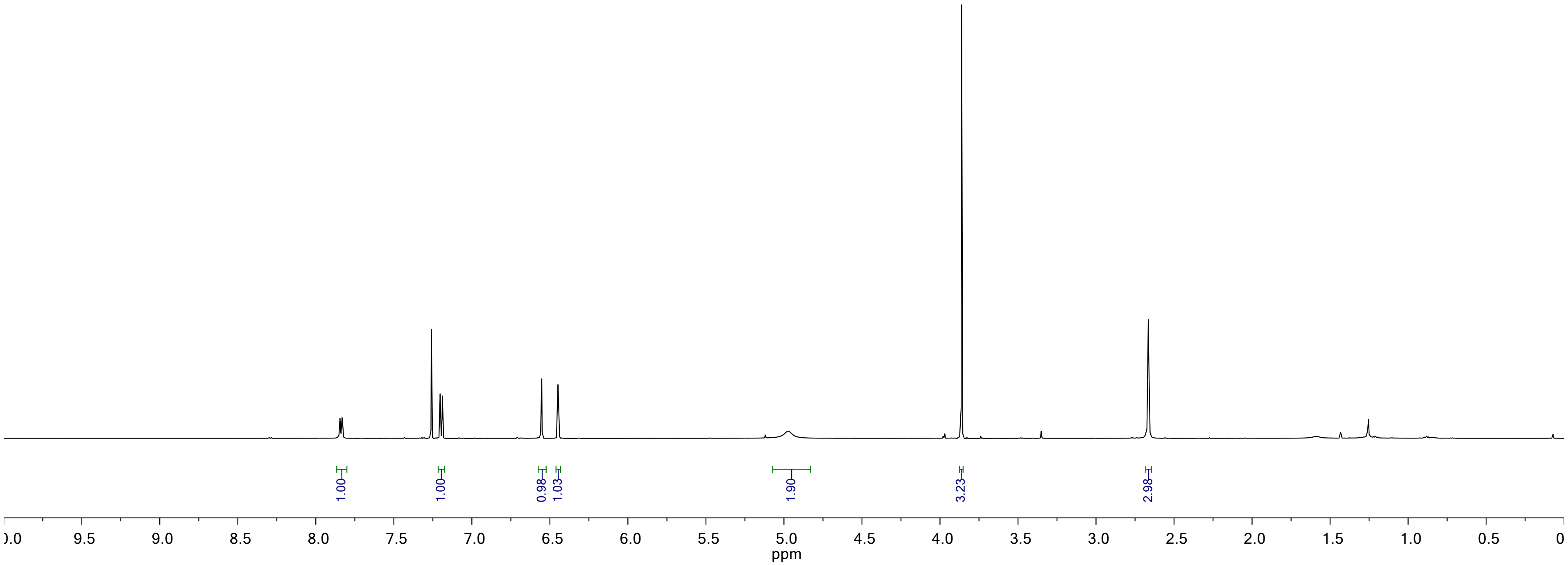

^{13}C NMR of 8-amino-6-methoxy-2-methylquinoline (7b)

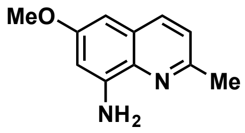

7b

$CDCl_3$, 23 ºC

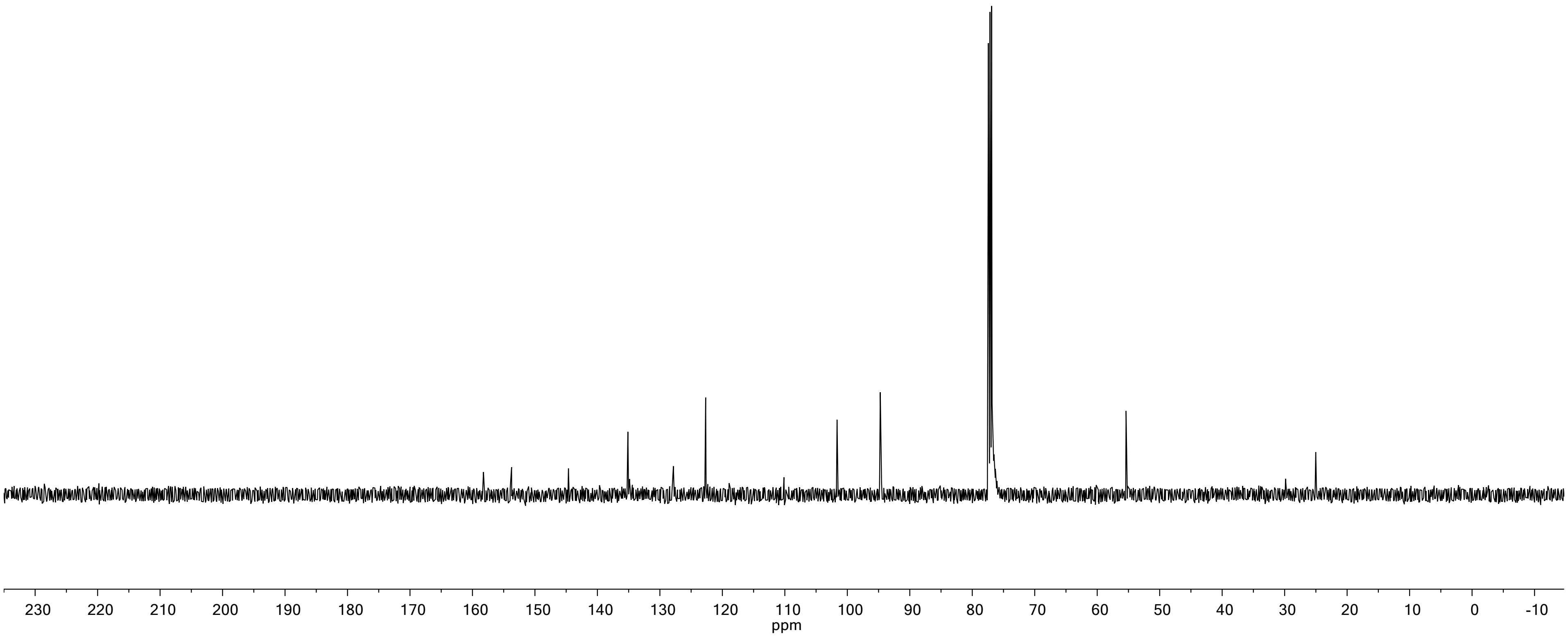

^{1}H NMR of 3-amino-4-methoxybenzenesulfonamide (8)

CD_3OD, 23 ºC

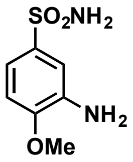

8

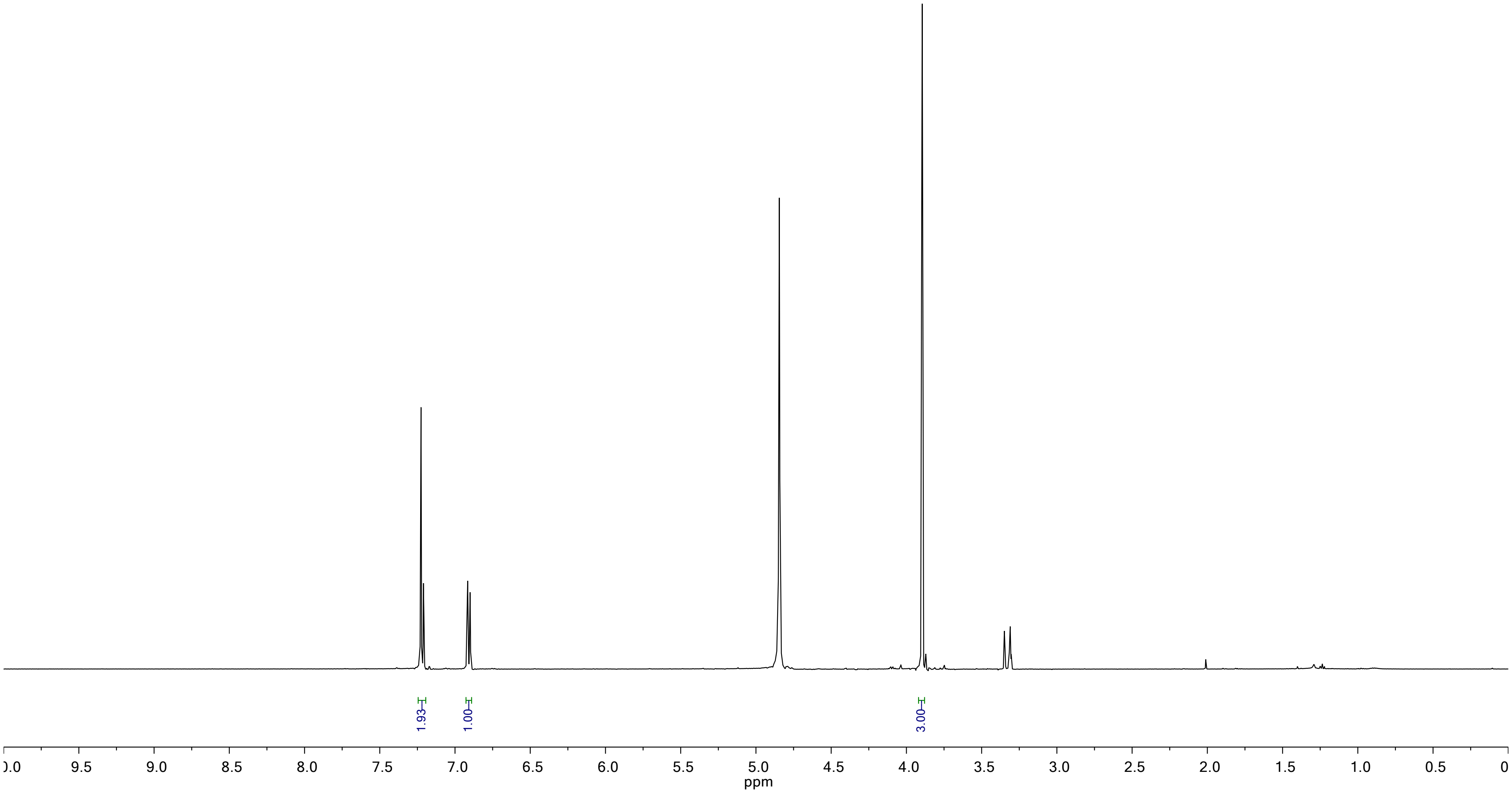

^{13}C NMR of 3-amino-4-methoxybenzenesulfonamide (8)

CD_3OD, 23 ºC

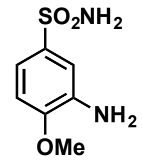

8

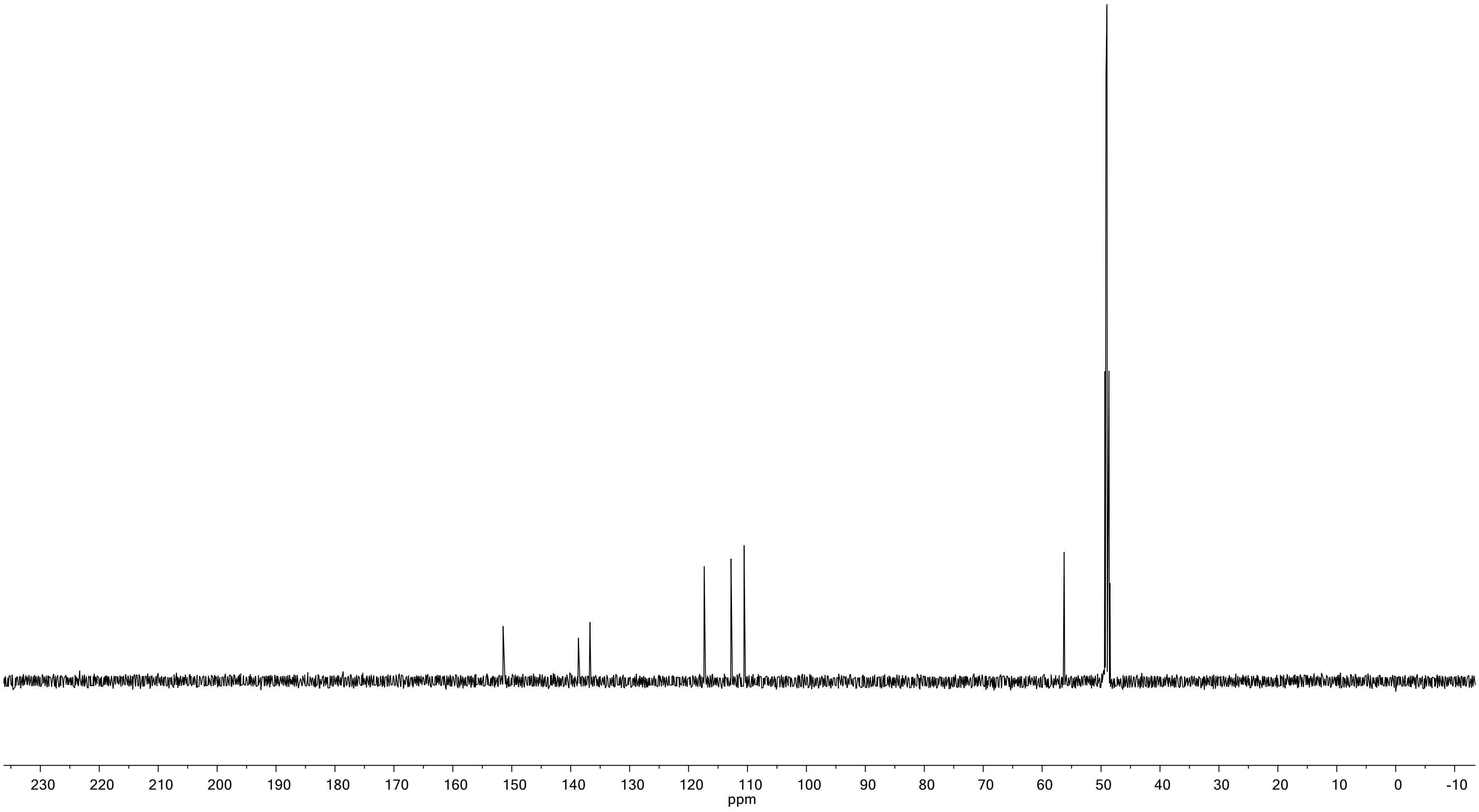

^{1}H NMR of methyl 4-amino-1*H*-benzimidazole-6-carboxylate (9a) and methyl 5-amino-1*H*-benzimidazole-6-carboxylate (9c)

CD_3OD, 23 ºC

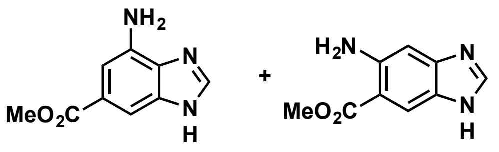

9a   9c

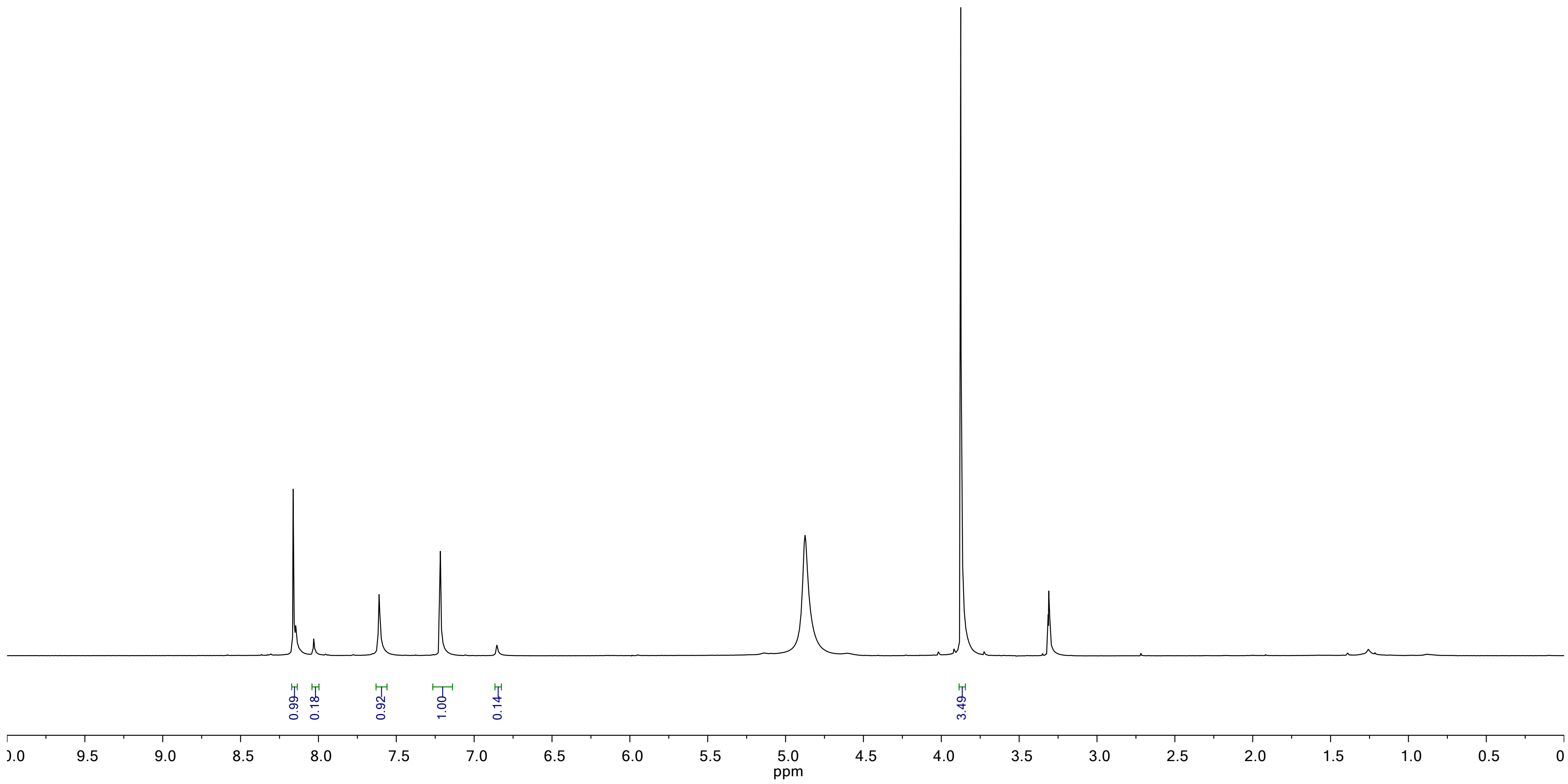

^{13}C NMR of methyl 4-amino-1*H*-benzimidazole-6-carboxylate (9a) and methyl 5-amino-1*H*-benzimidazole-6-carboxylate (9c)

CD_3OD/CD_2Cl_2, 23 ºC

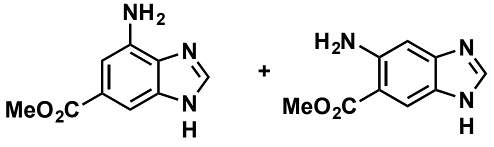

9a 9c

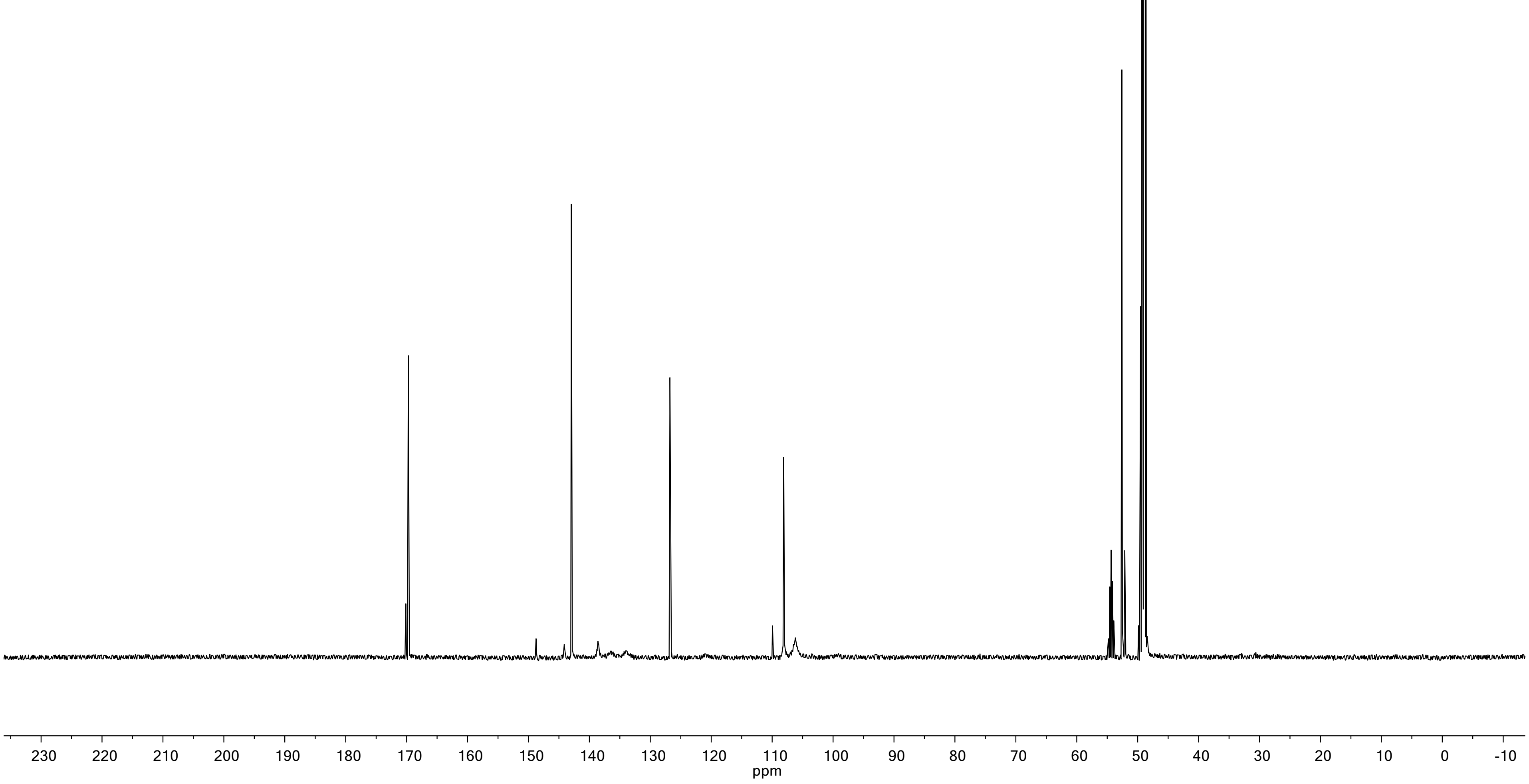

^{1}H NMR of methyl 7-amino-1*H*-benzimidazole-6-carboxylate (9b)

CD_3OD, 23 ºC

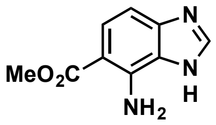

9b

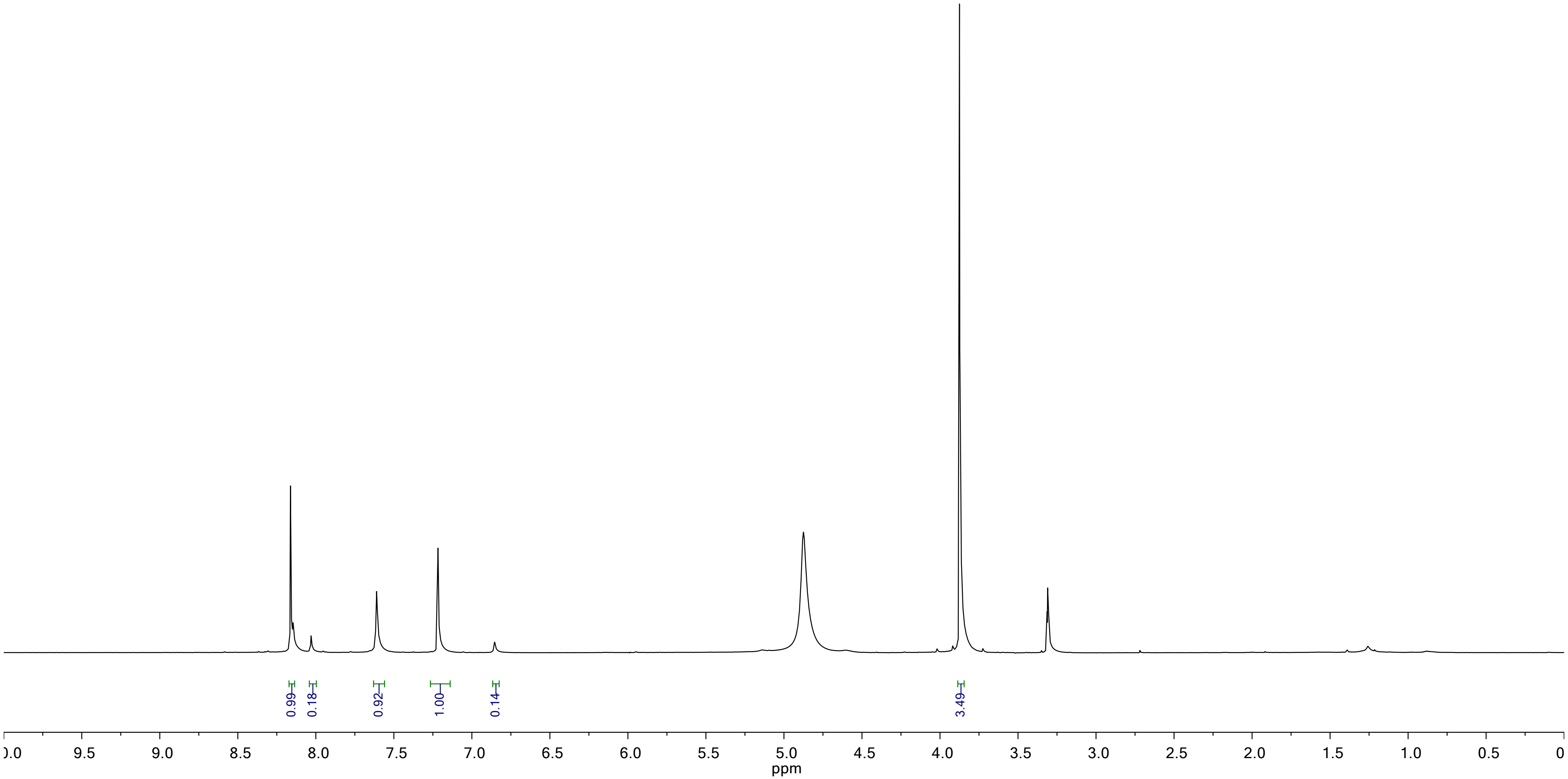

^{13}C NMR of methyl 7-amino-1*H*-benzimidazole-6-carboxylate (9b)

CD_3OD, 23 ºC

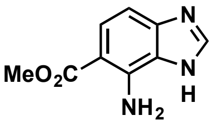

9b

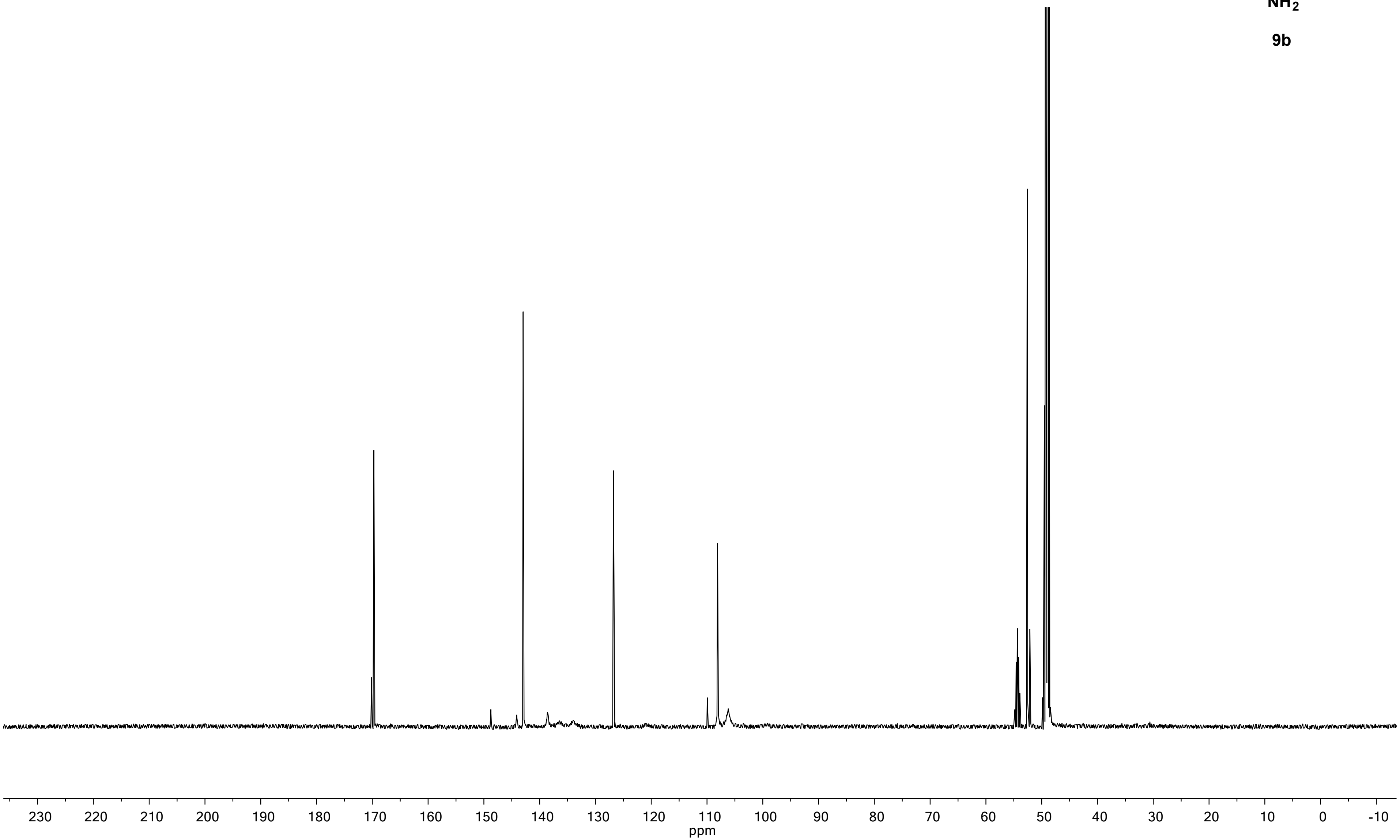

^{1}H NMR of 3-amino-2,6-dichlorobenzonitrile (10a)

$CDCl_3/CD_3OD$, 23 ºC

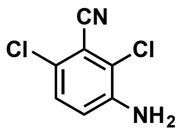

10a

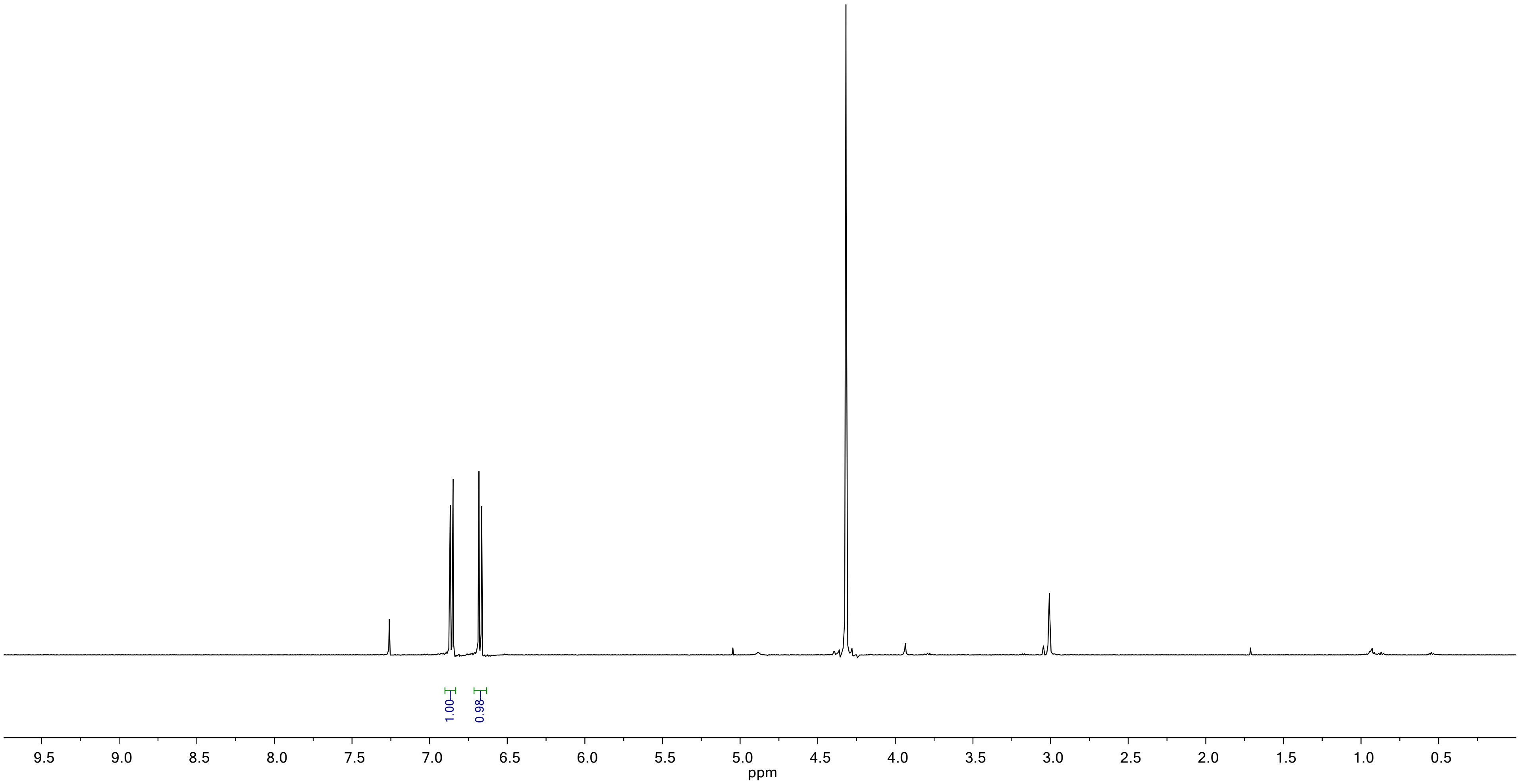

^{13}C NMR of 3-amino-2,6-dichlorobenzonitrile (10a)

$CDCl_3/CD_3OD$, 23 ºC

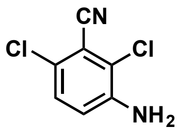

10a

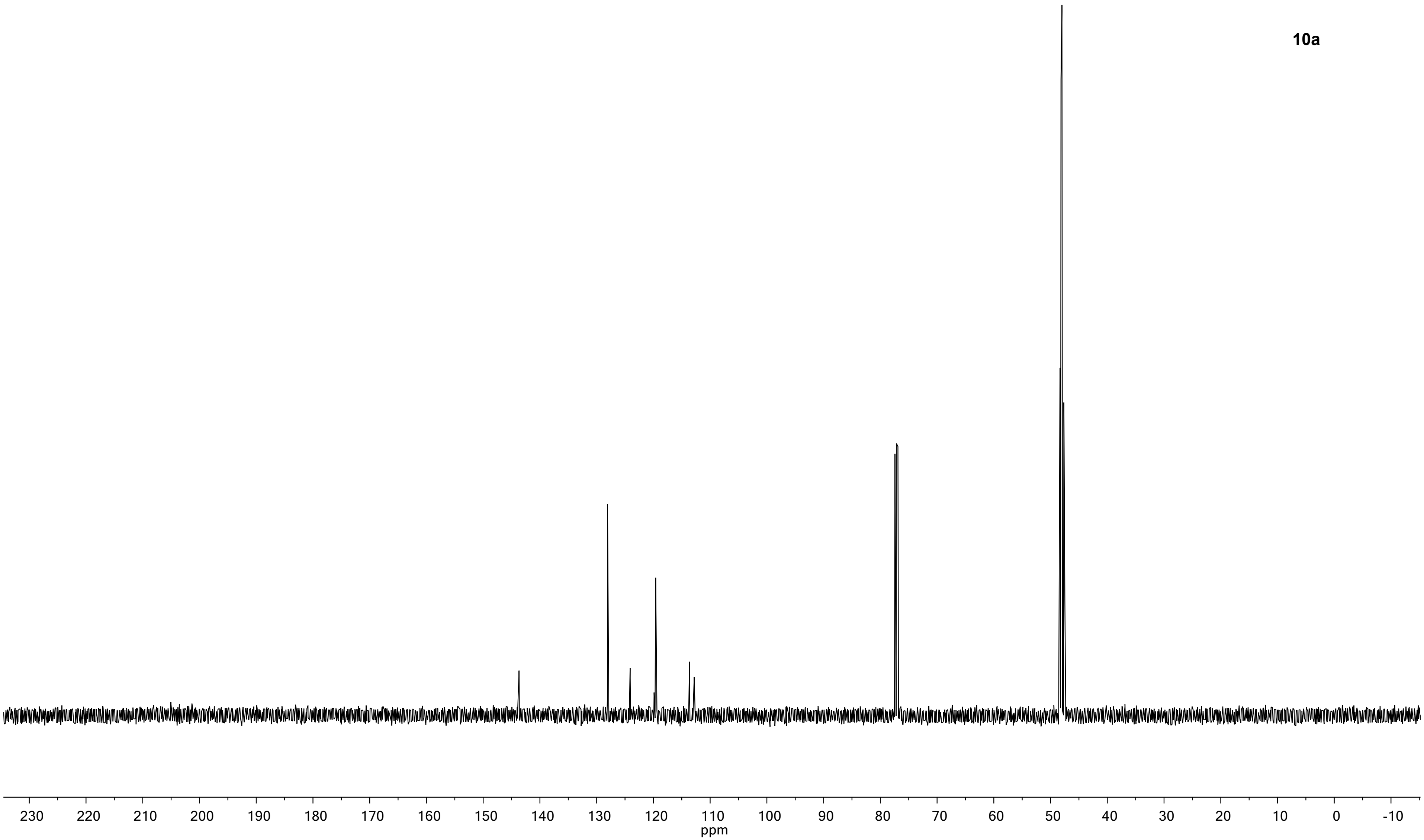

^{1}H NMR of 4-amino-2,6-dichlorobenzonitrile (10b)

CD_3OD, 23 ºC

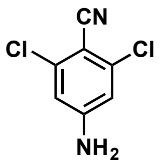

10b

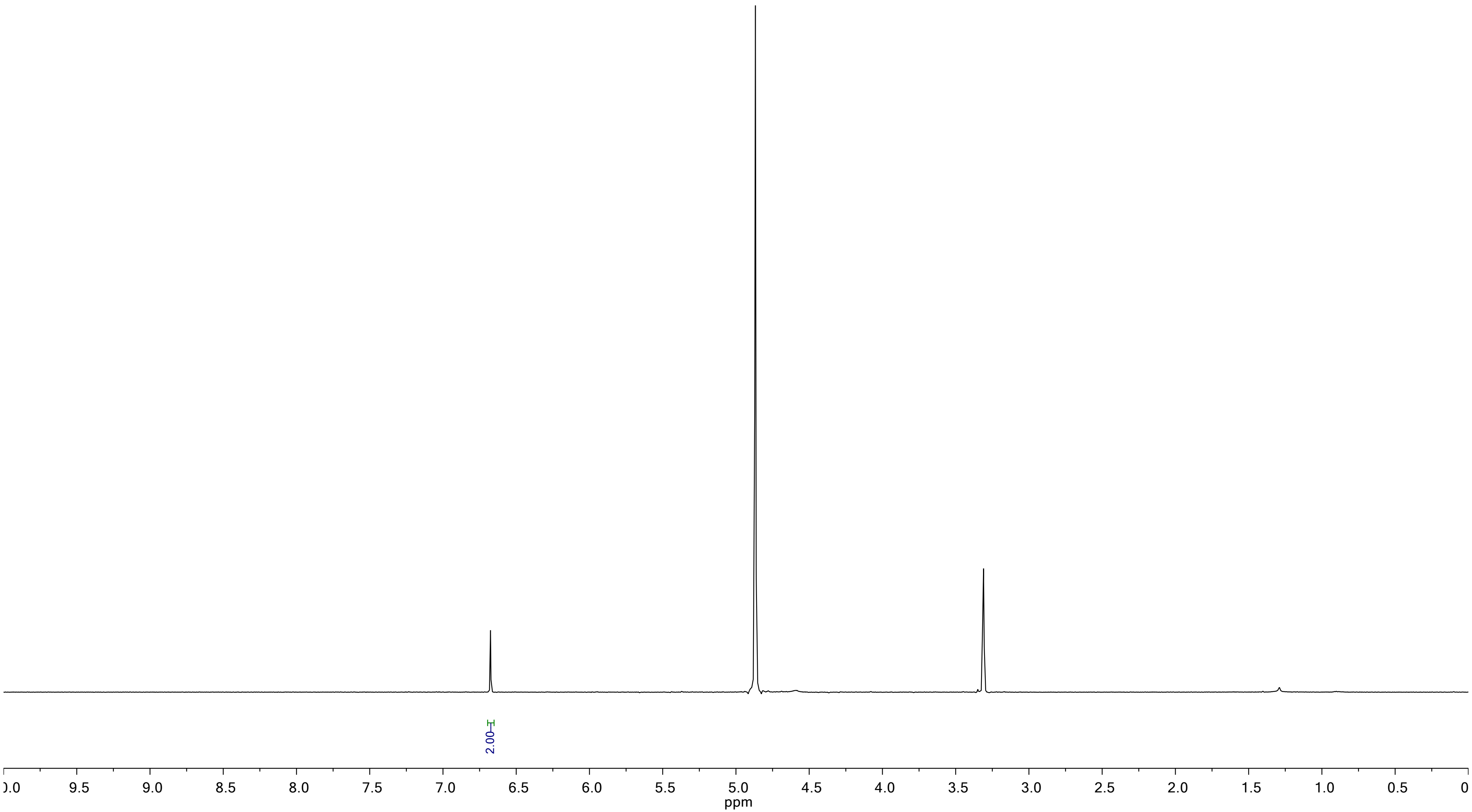

^{13}C NMR of 4-amino-2,6-dichlorobenzonitrile (10b)

CD_3OD, 23 ºC

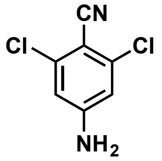

10b

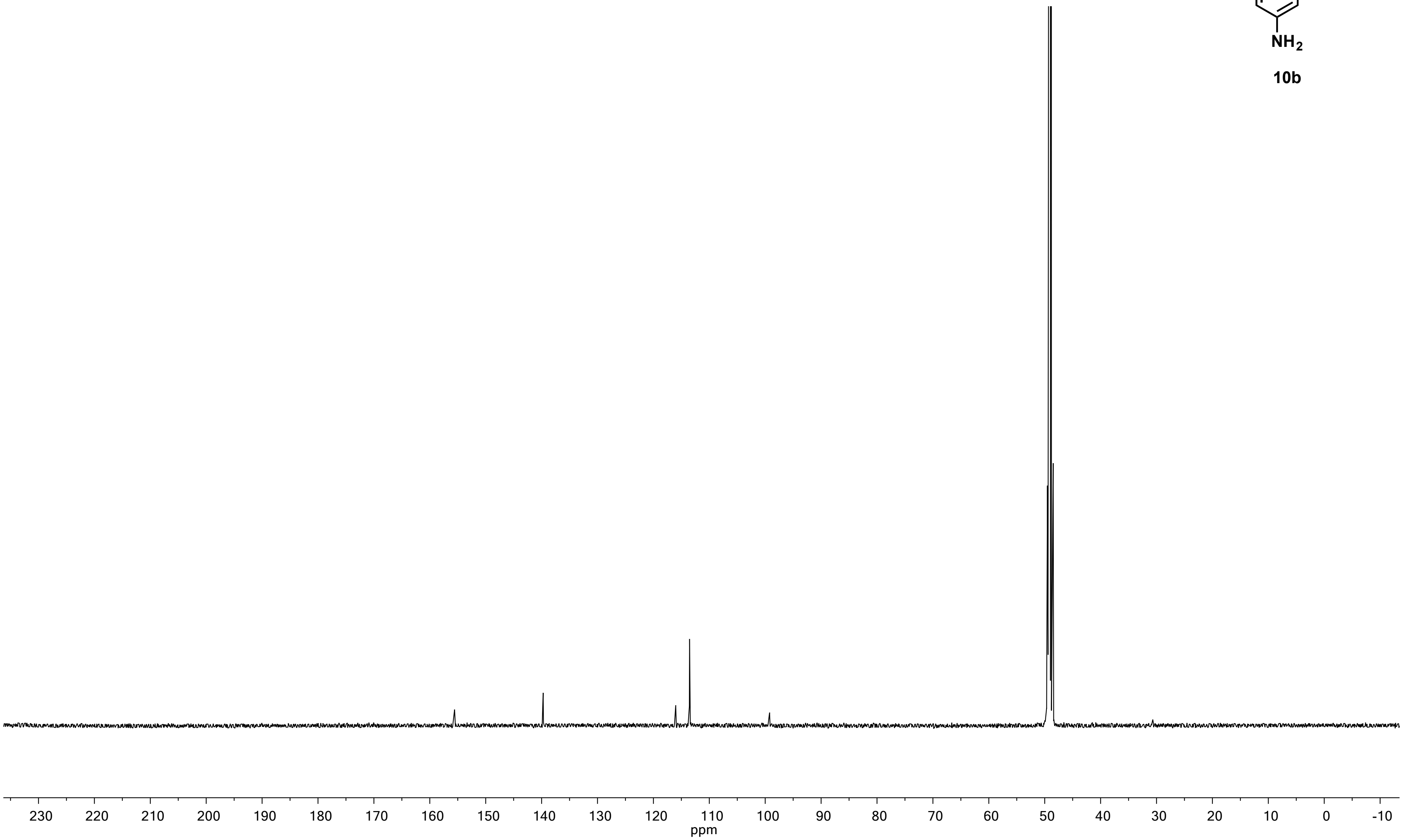

^{1}H NMR of 3-aminomoclebomide (11a)

$CDCl_3$, 23 ºC

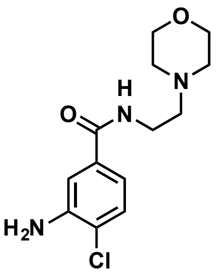

11a

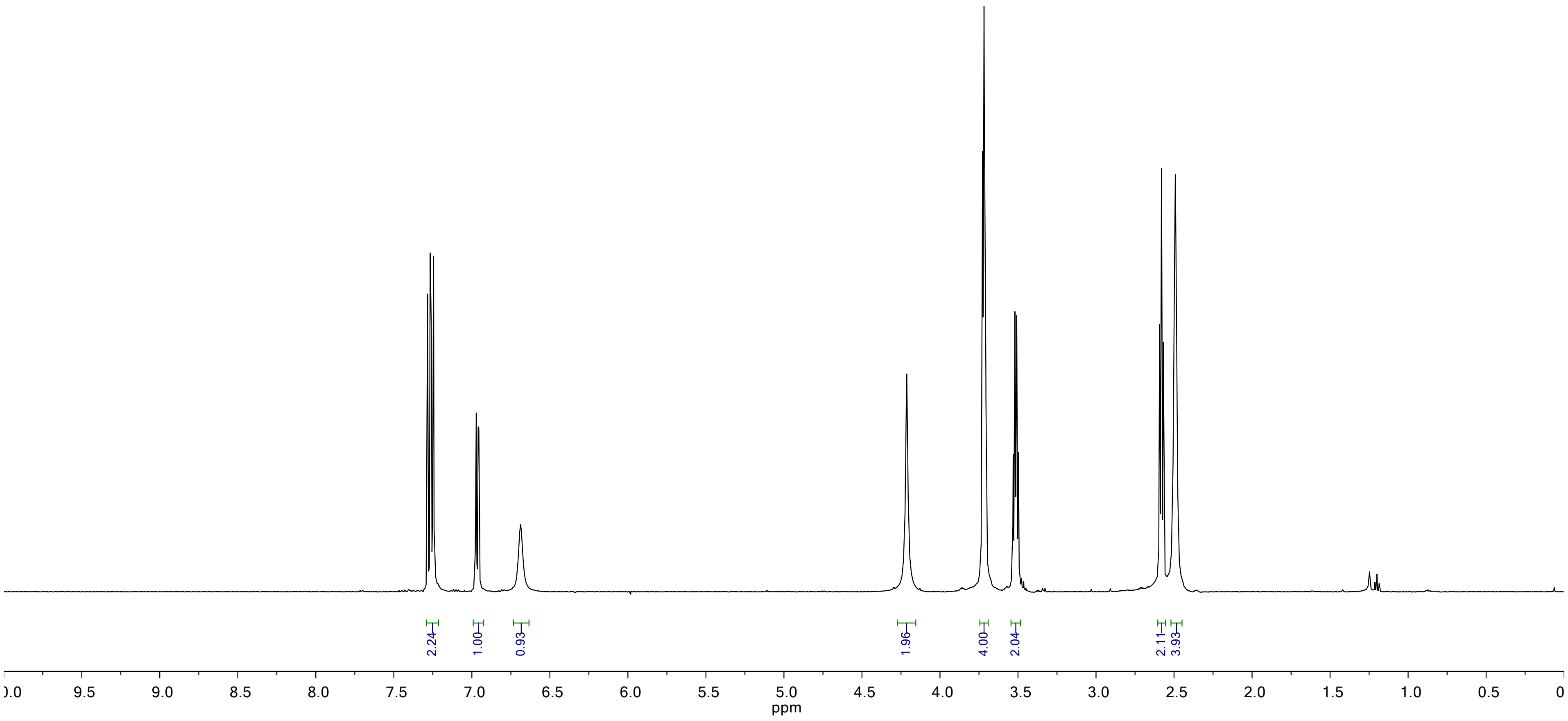

^{13}C NMR of 3-aminomoclebomide (11a)

$CDCl_3$, 23 ºC

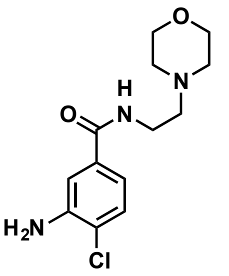

11a

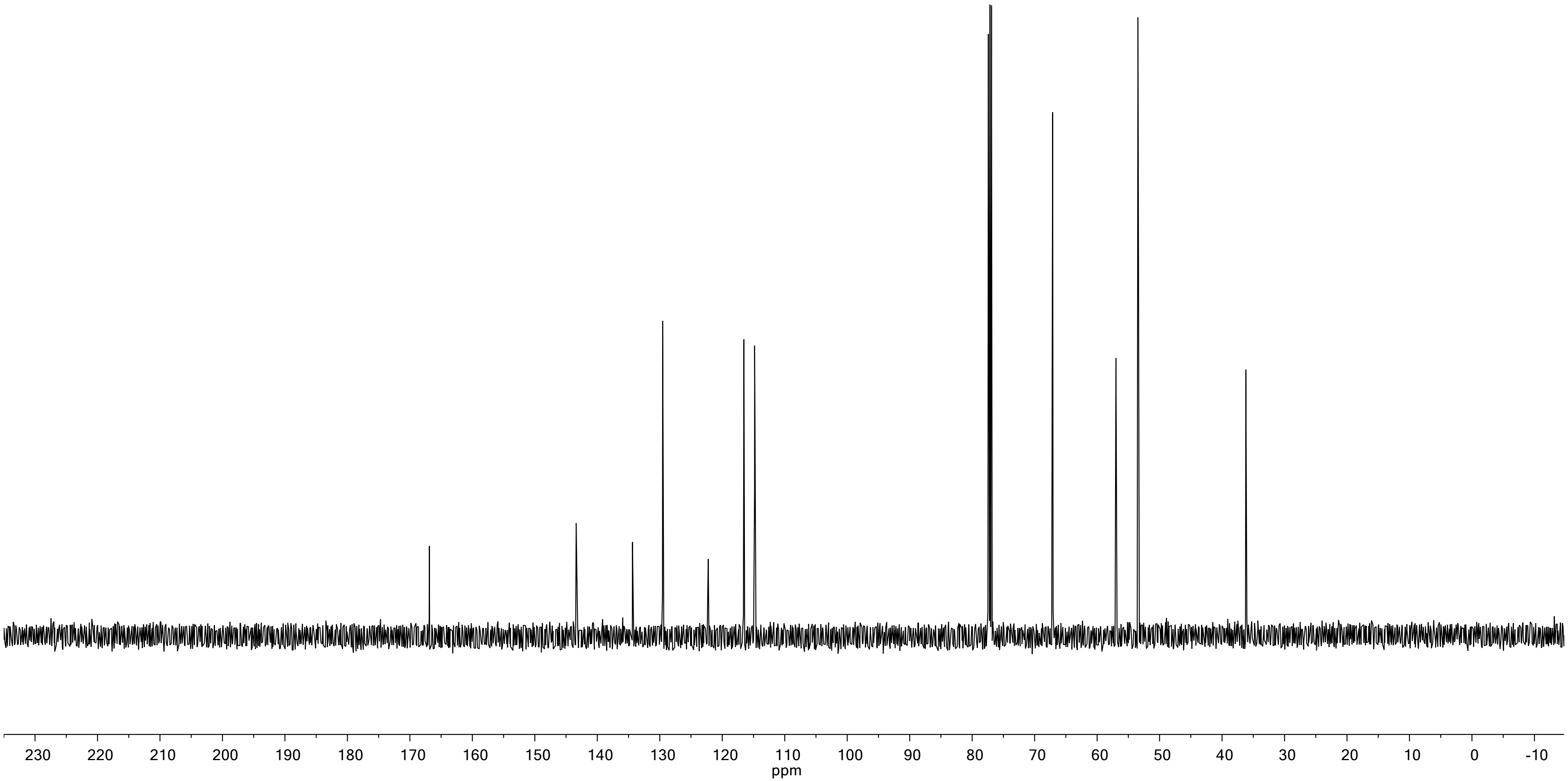

^{1}H NMR of 2-aminomoclebomide (11b)

$CDCl_3$, 23 ºC

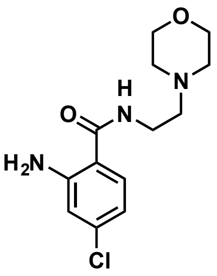

11b

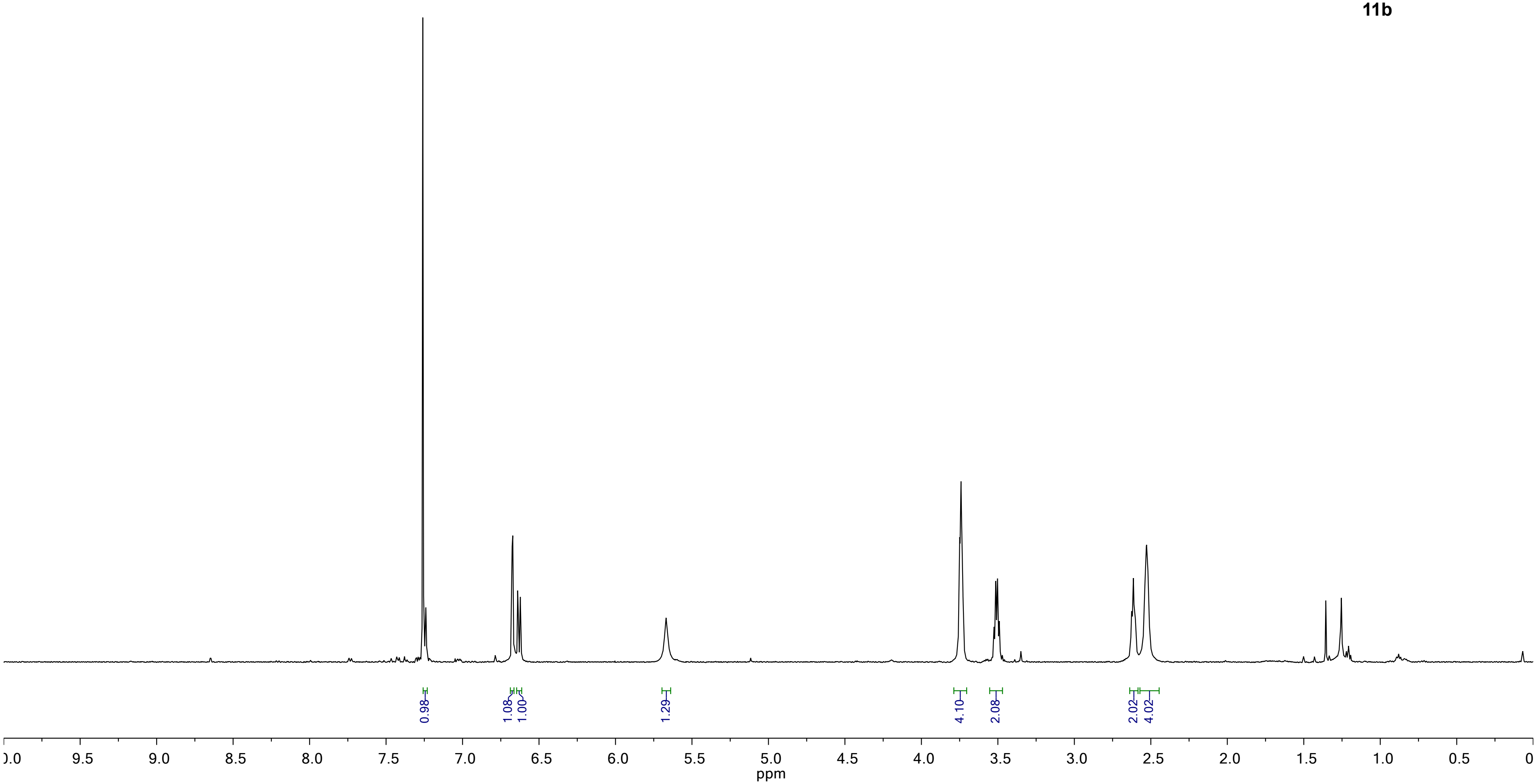

^{13}C NMR of 2-aminomoclebomide (11b)

$CDCl_3$, 23 ºC

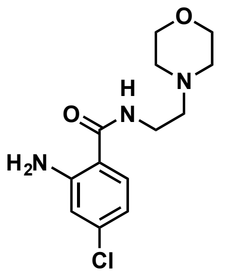

11b

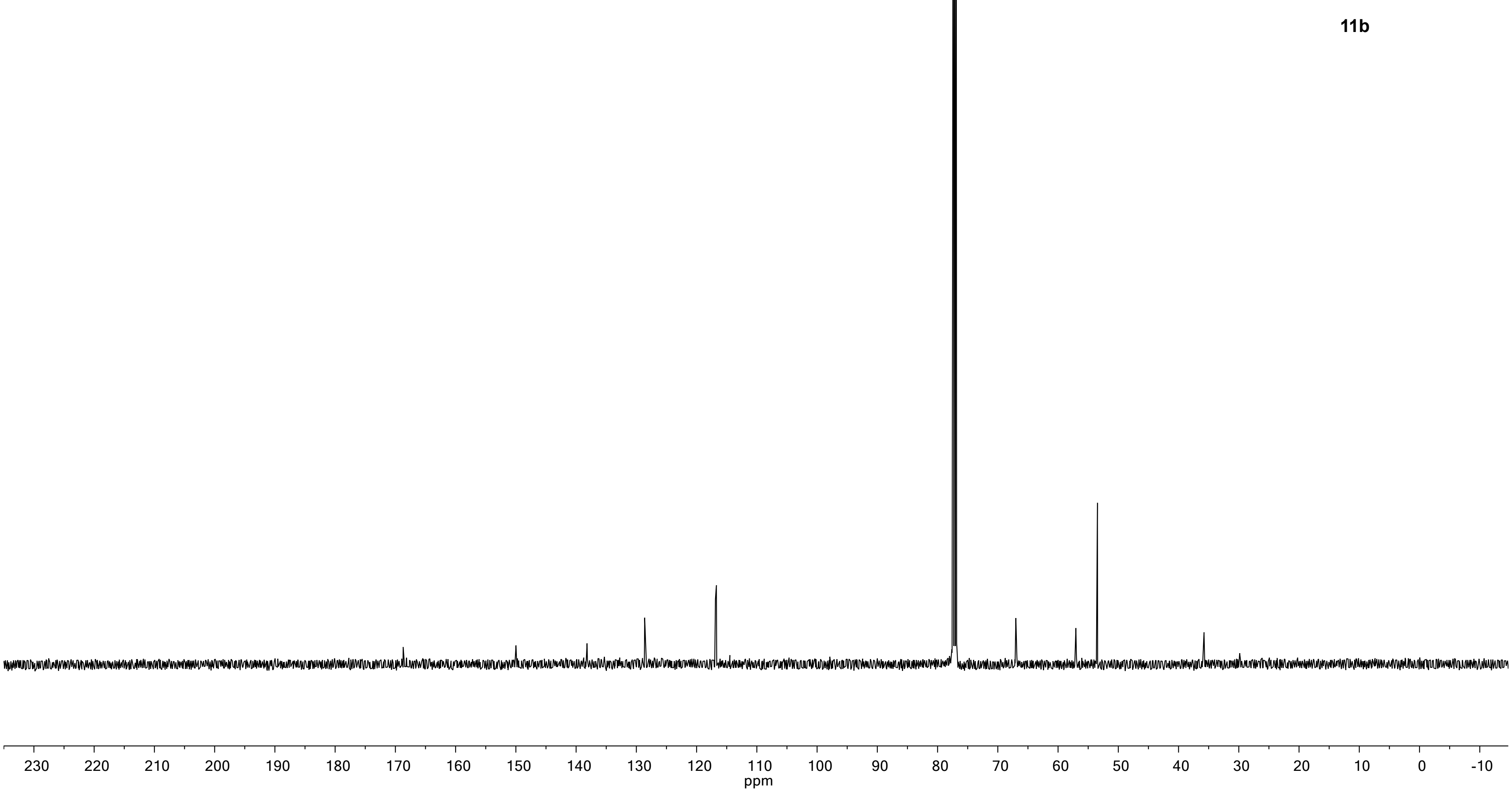

^{1}H NMR of 2-amino-4-(trifluoromethyl)phenyl 2-nitrobenzenesulfonate (12a)

$CDCl_3$, 23 ºC

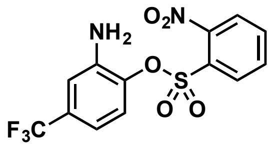

12a

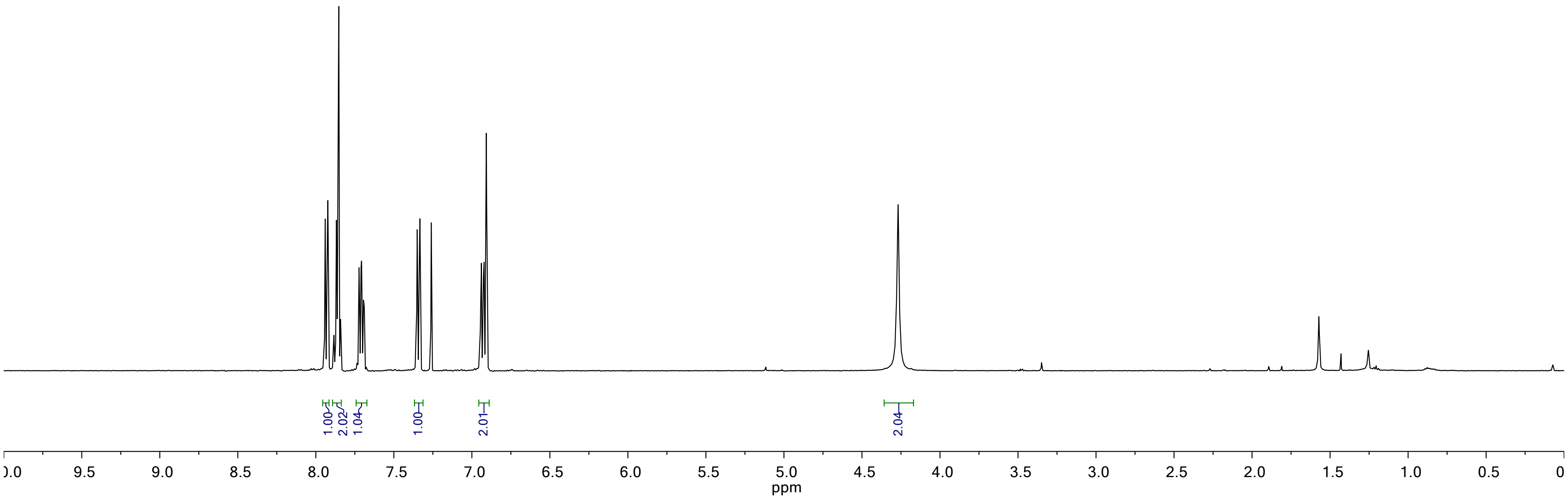

^{13}C NMR of 2-amino-4-(trifluoromethyl)phenyl 2-nitrobenzenesulfonate (12a)

$CDCl_3$, 23 ºC

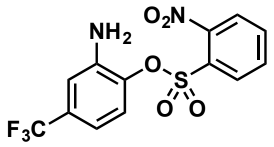

12a

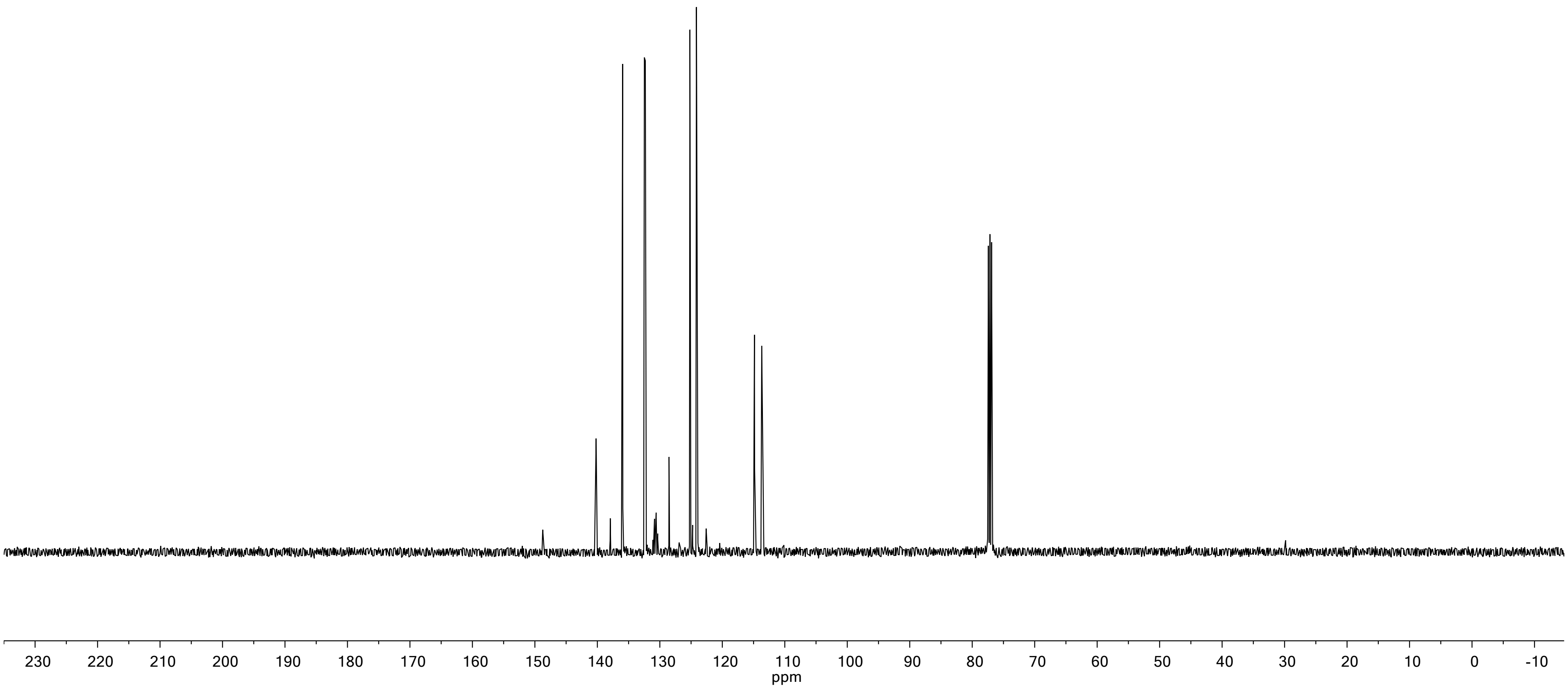

^{19}F NMR of 2-amino-4-(trifluoromethyl)phenyl 2-nitrobenzenesulfonate (12a)

$CDCl_3$, 23 ºC

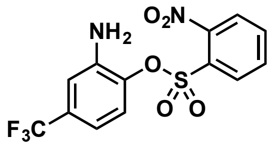

12a

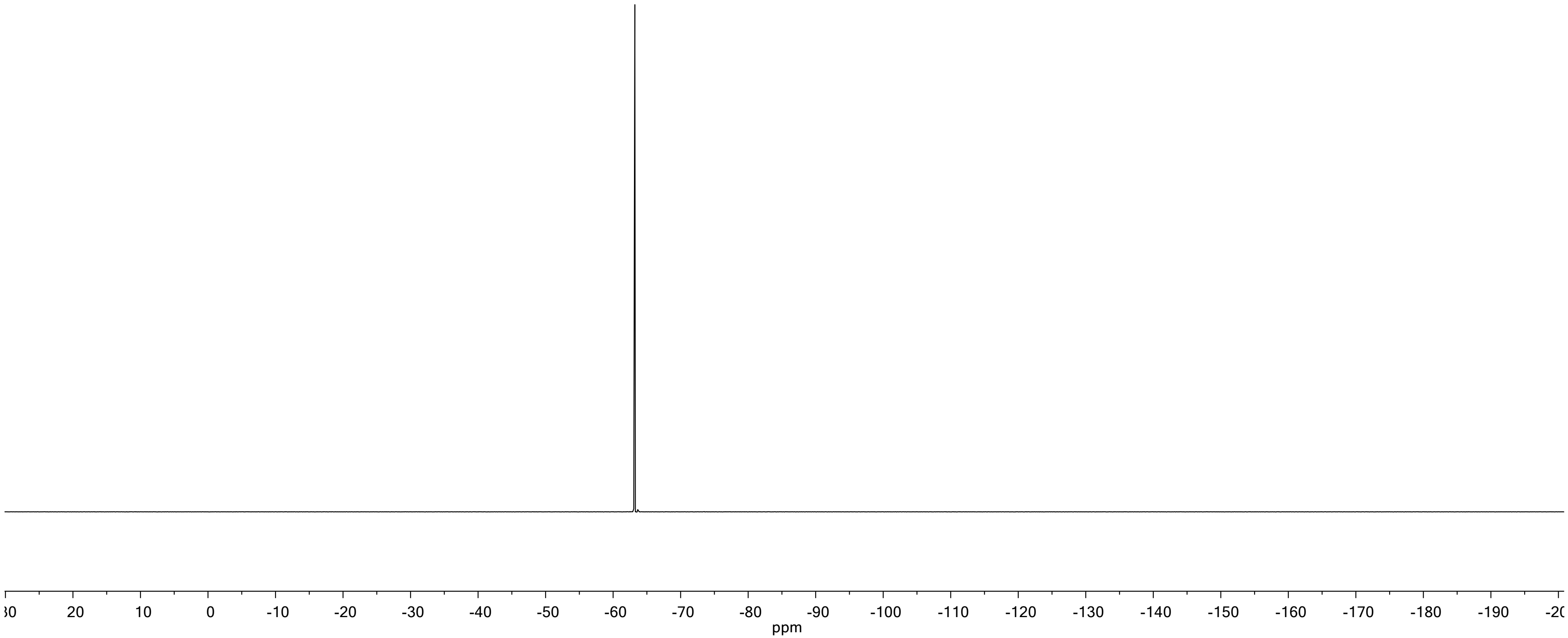

^{1}H NMR of 3-amino-4-(trifluoromethyl)phenyl 2-nitrobenzenesulfonate (12b)

$CDCl_3$, 23 ºC

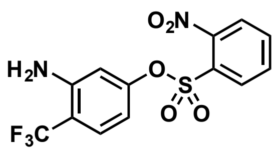

12b

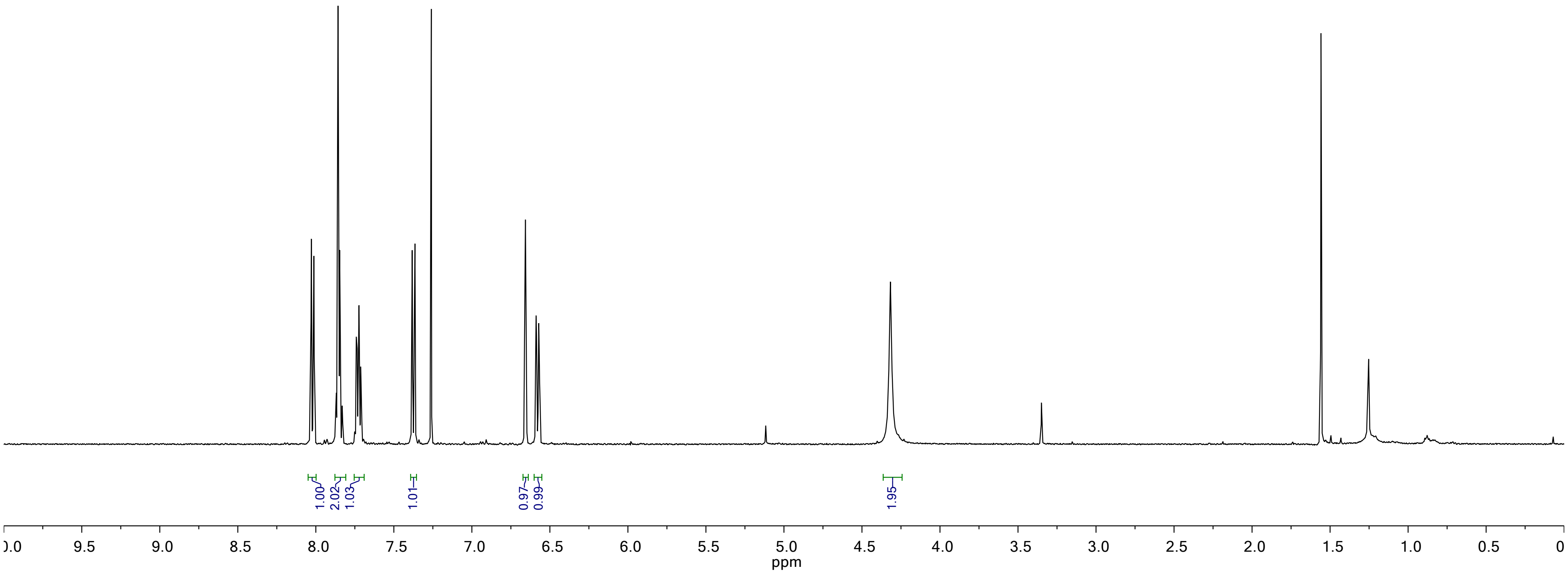

^{13}C NMR of 3-amino-4-(trifluoromethyl)phenyl 2-nitrobenzenesulfonate (12b)

$CDCl_3$, 23 ºC

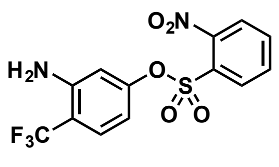

12b

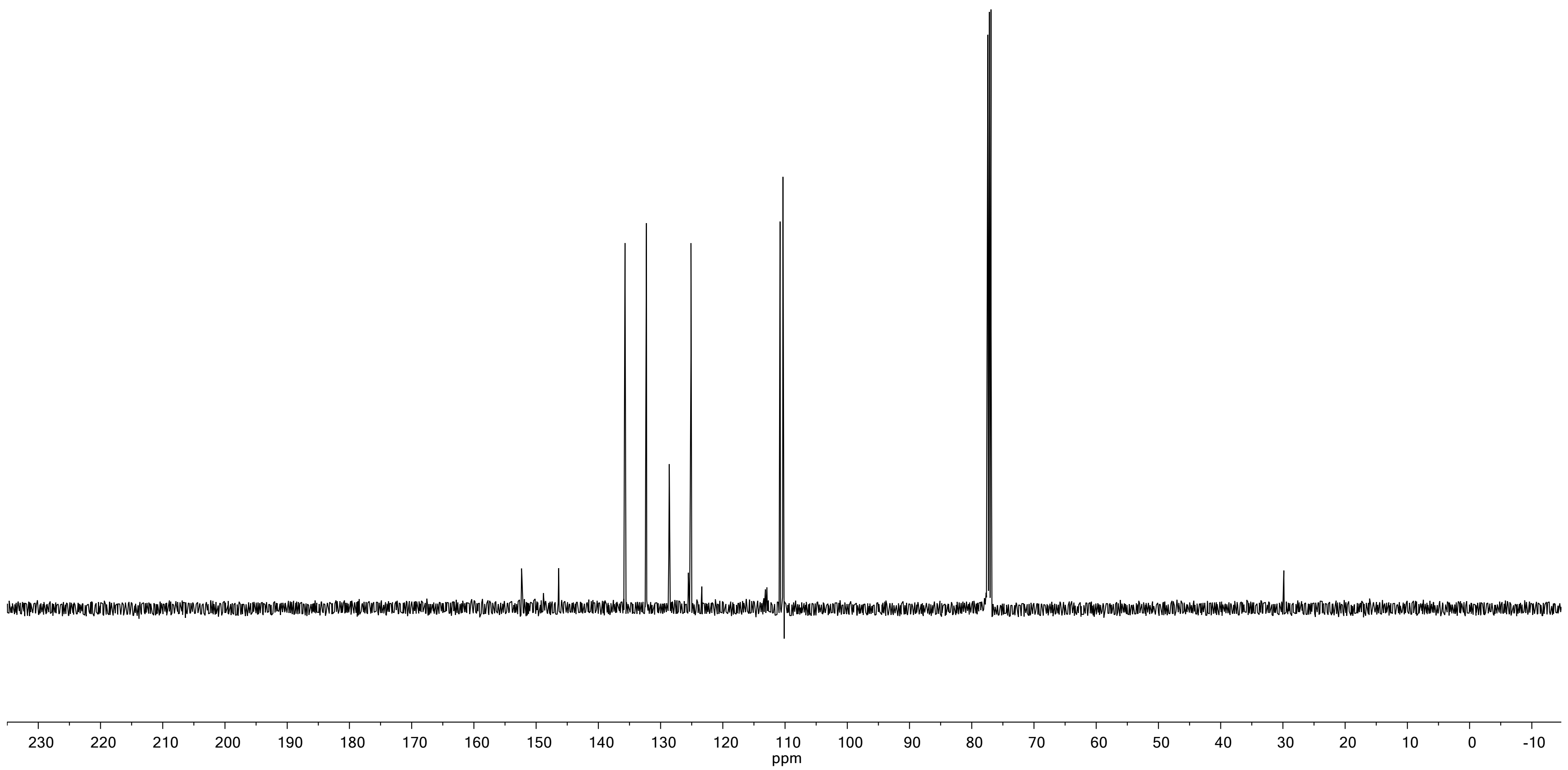

^{19}F NMR of 3-amino-4-(trifluoromethyl)phenyl 2-nitrobenzenesulfonate (12b)

$CDCl_3$, 23 ºC

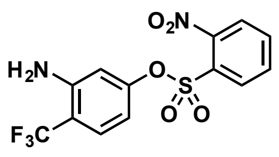

12b

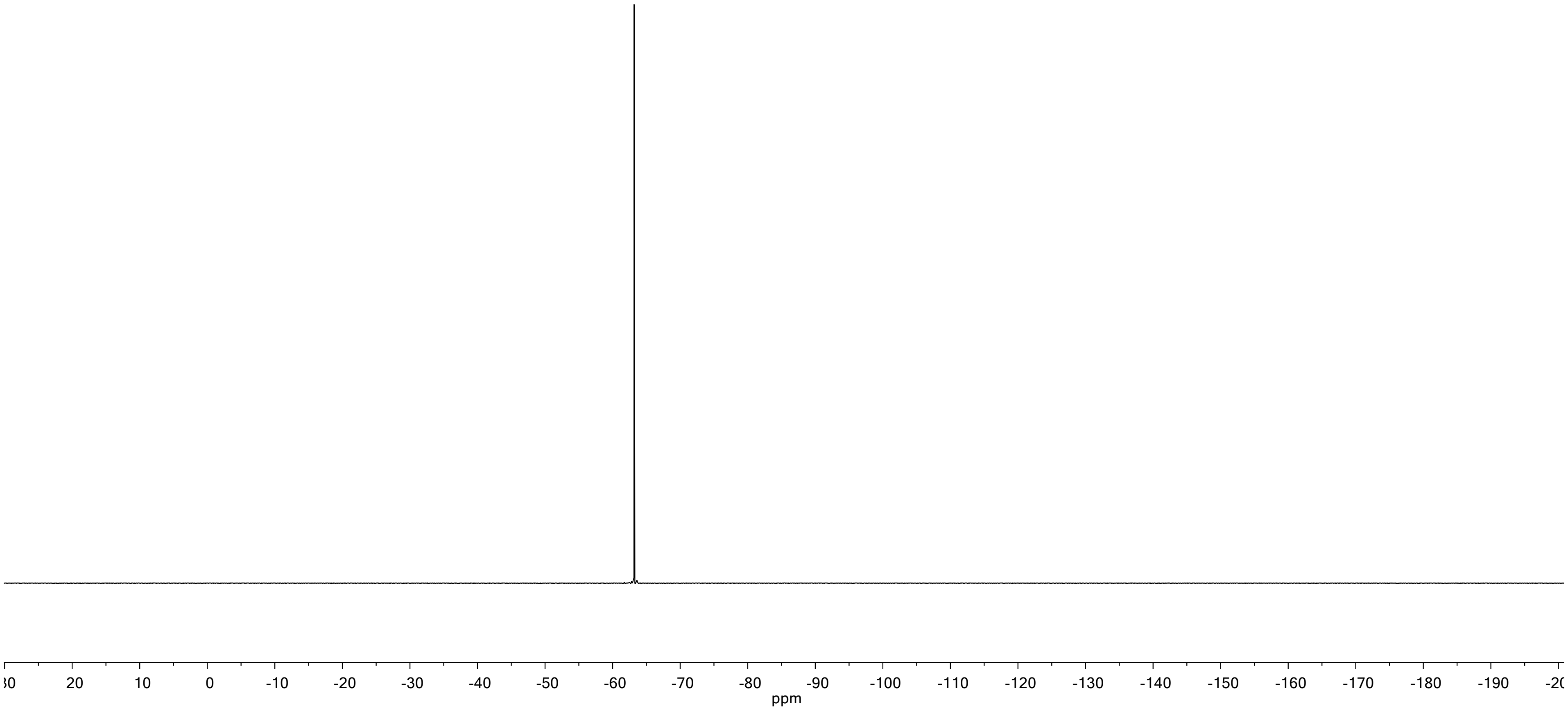

^{1}H NMR of 3-aminorufinamide (13a) and 4-aminorufinamide (13b)

$(CD_3)_2SO$, 23 ºC

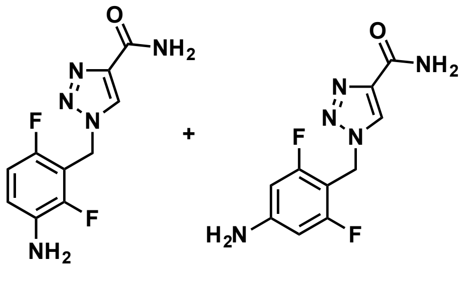

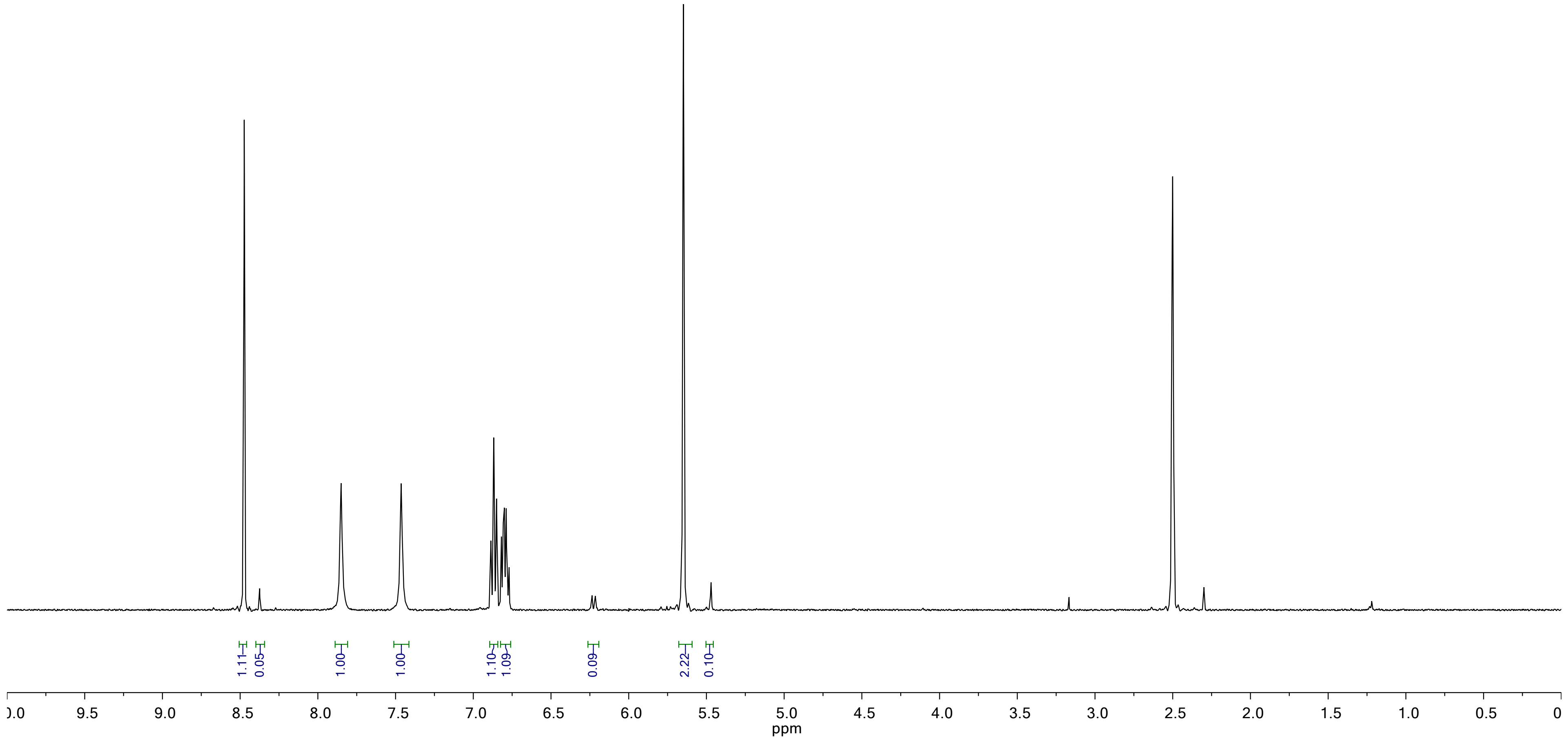

^{13}C NMR of 3-aminorufinamide (13a) and 4-aminorufinamide (13b)

$(CD_3)_2SO$, 23 ºC

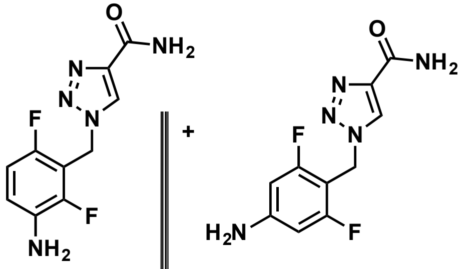

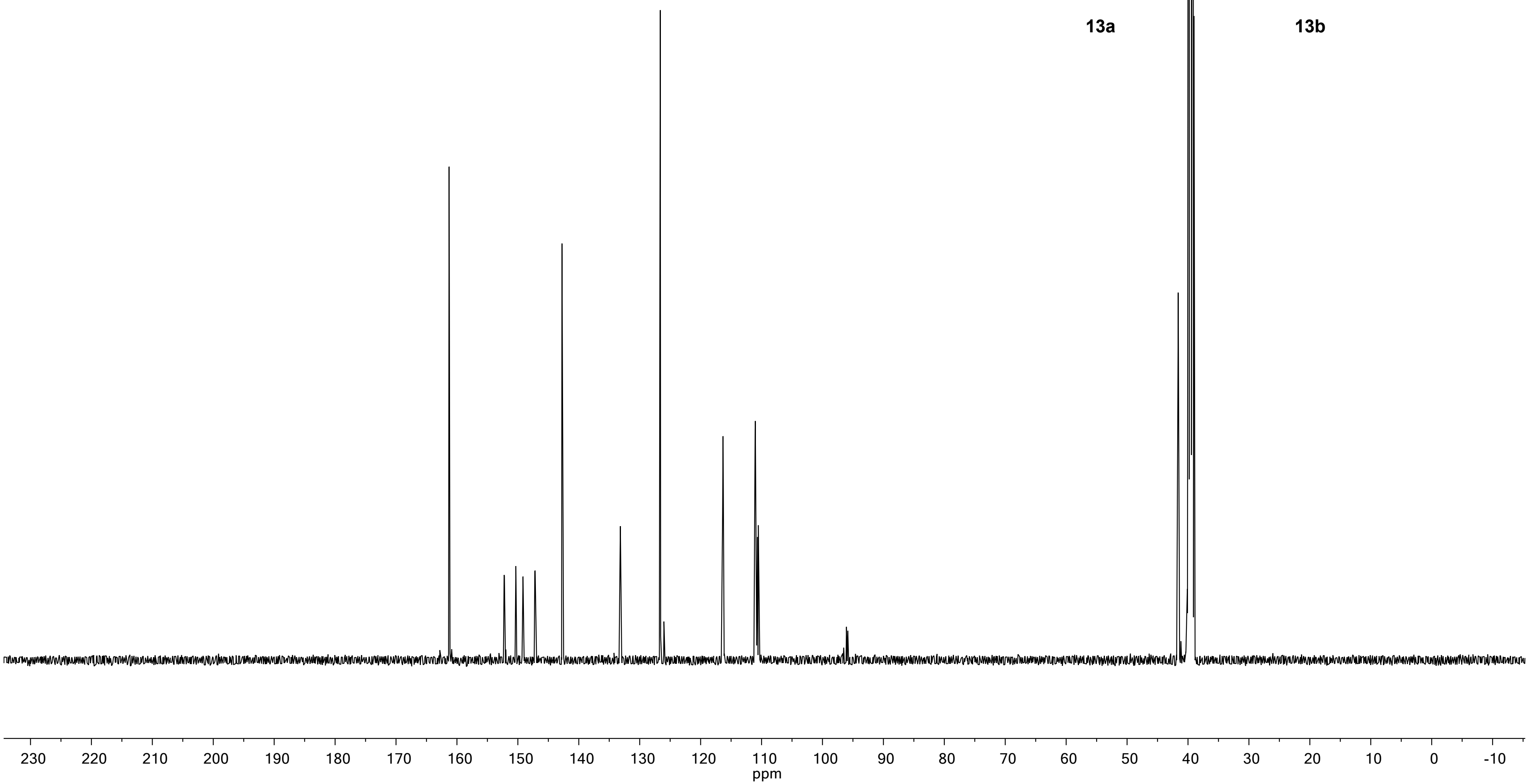

^{19}F NMR of 3-aminorufinamide (13a) and 4-aminorufinamide (13b)

CDCl$_3$, 23 ºC

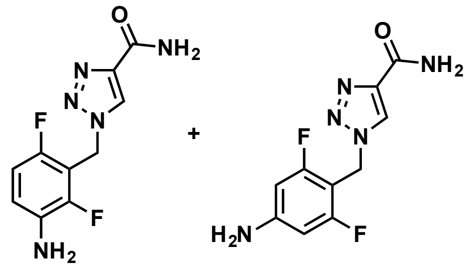

13a 13b

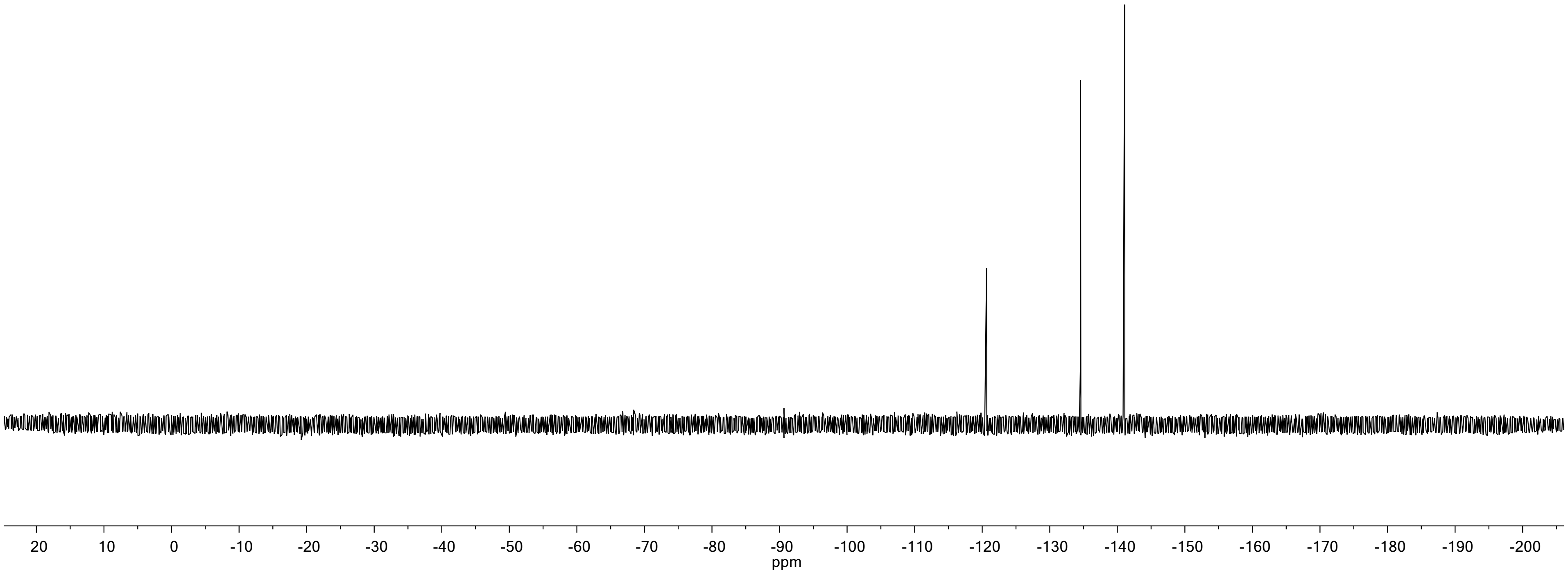

^{1}H NMR of 4-bromoaniline (S4a)

CD_2Cl_2, 23 ºC

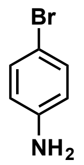

S4a

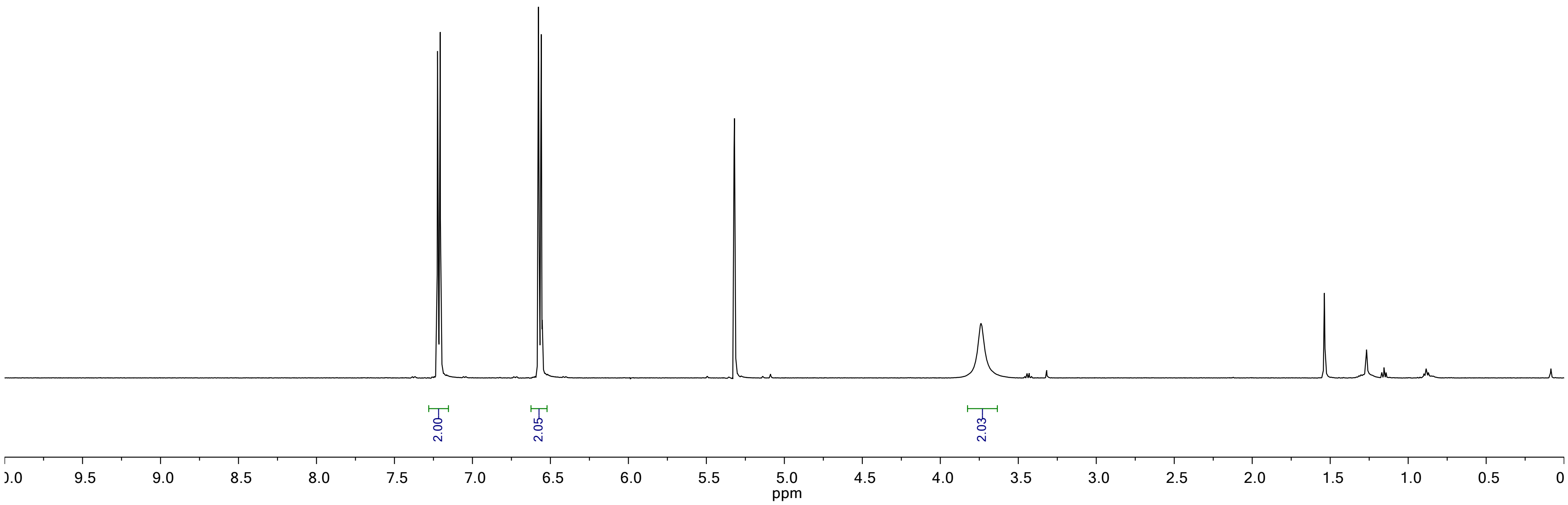

^{13}C NMR of 4-bromoaniline (S4a)

CD_2Cl_2, 23 ºC

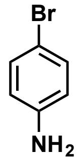

S4a

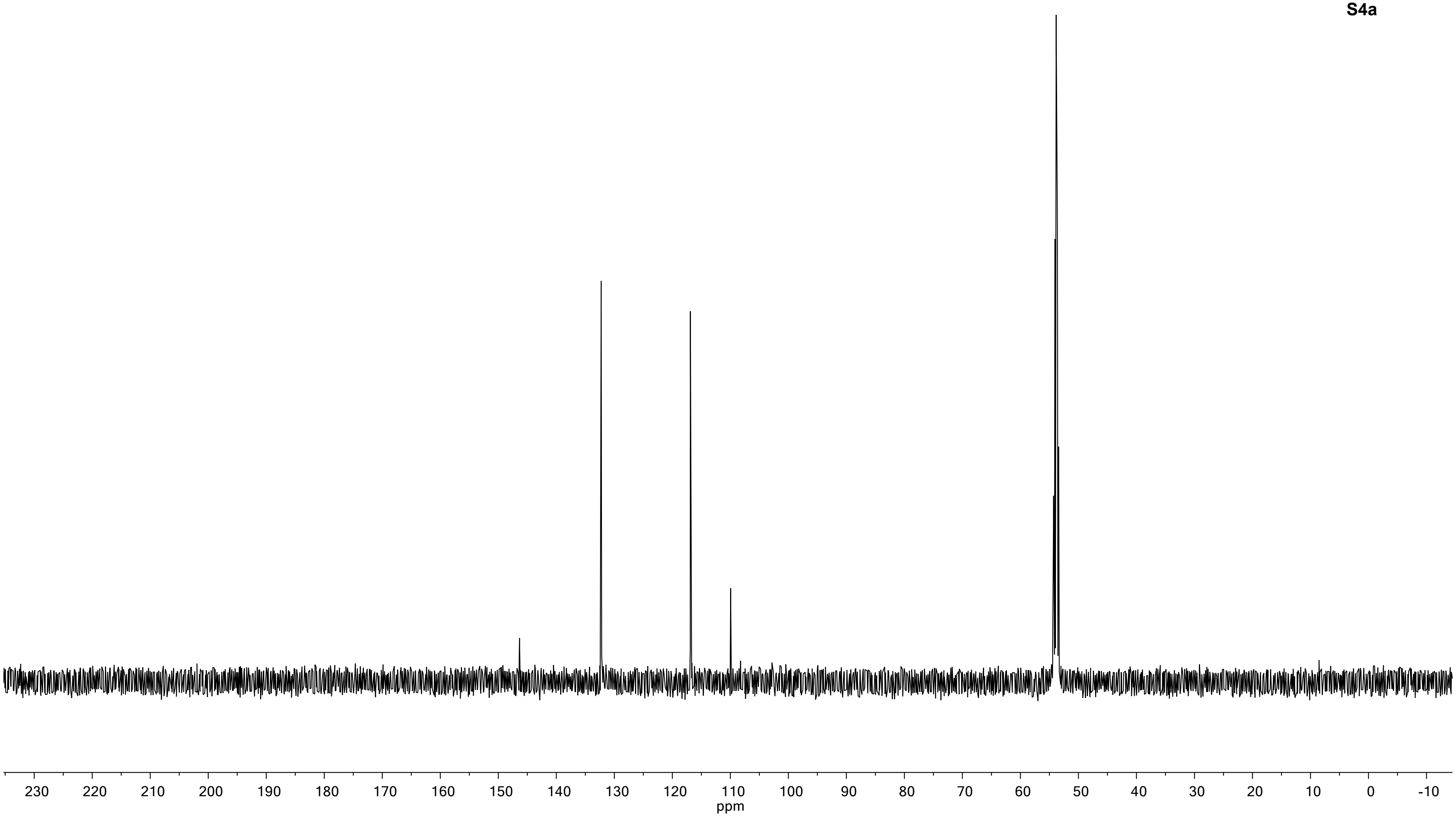

^{1}H NMR of 2-bromoaniline (S4b)

CD_2Cl_2, 23 ºC

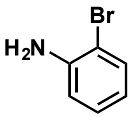

S4b

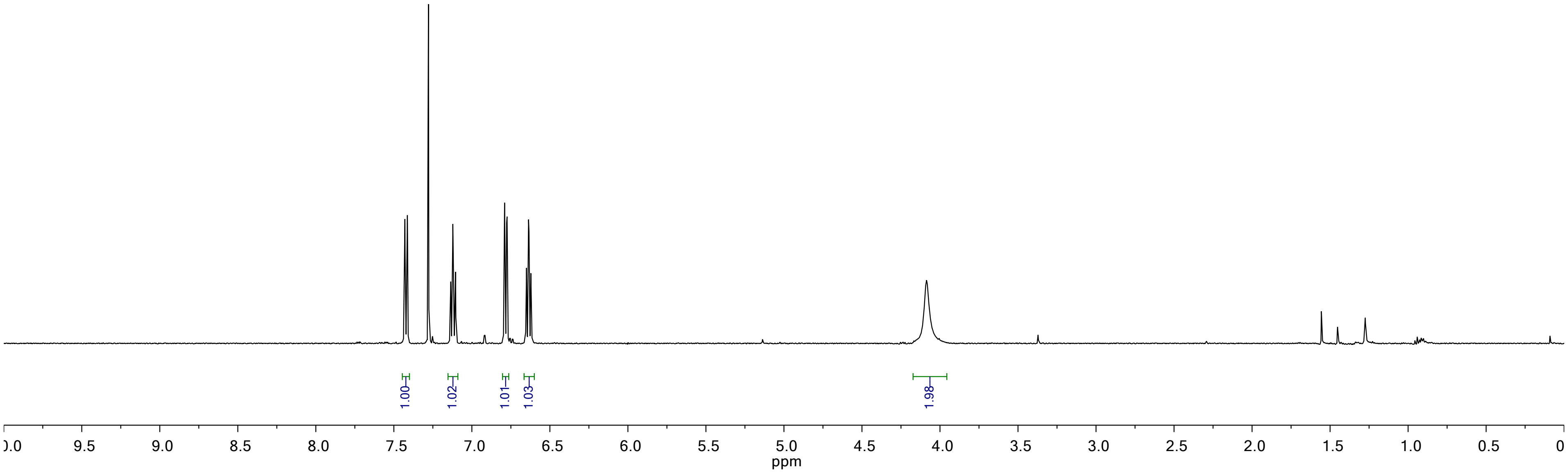

^{13}C NMR of 2-bromoaniline (S4b)

CD_2Cl_2, 23 ºC

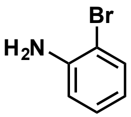

S4b

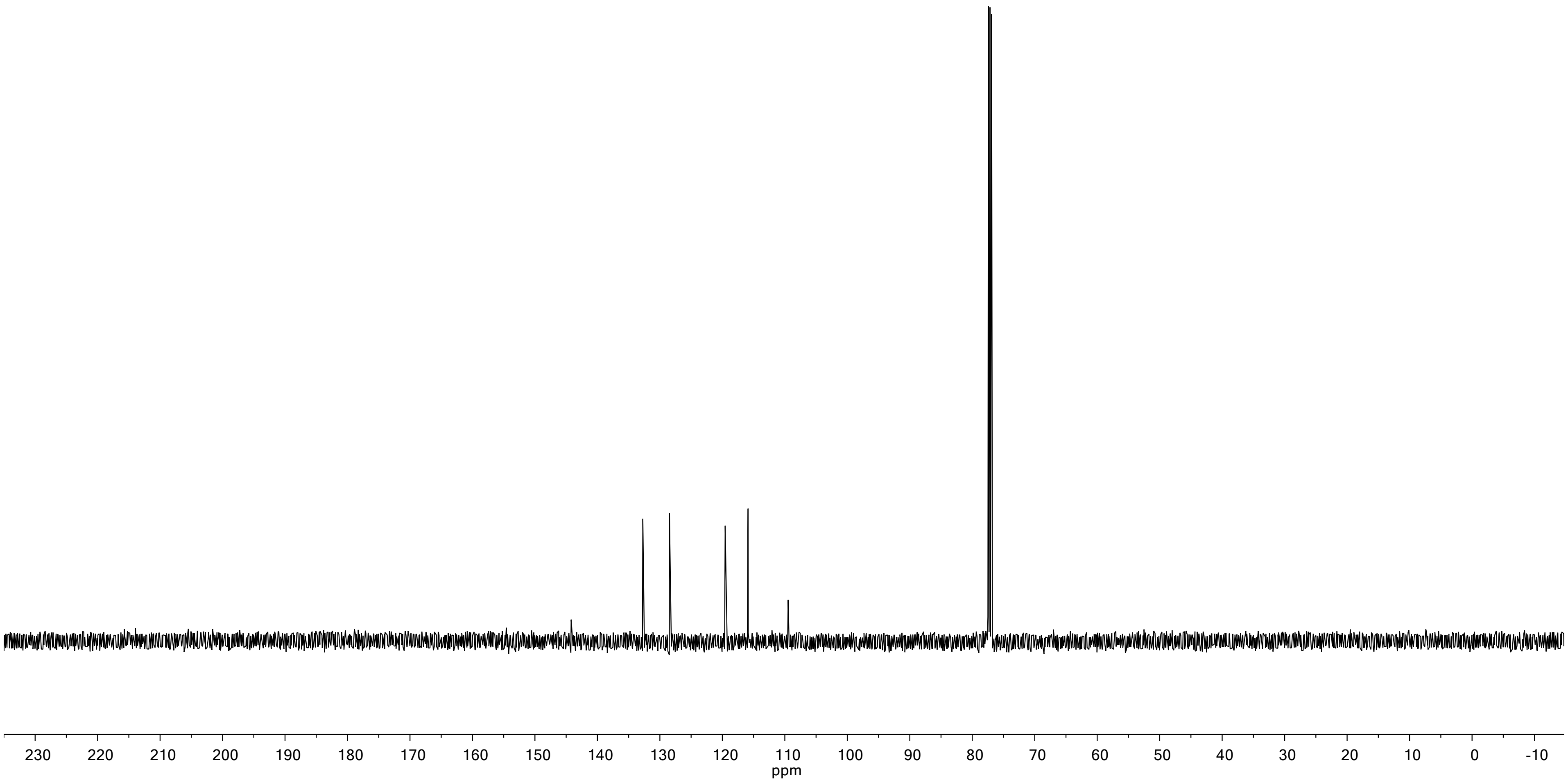

^1H NMR of 3-bromoaniline (S4c)

CD_2Cl_2, 23 ºC

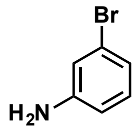

S4c

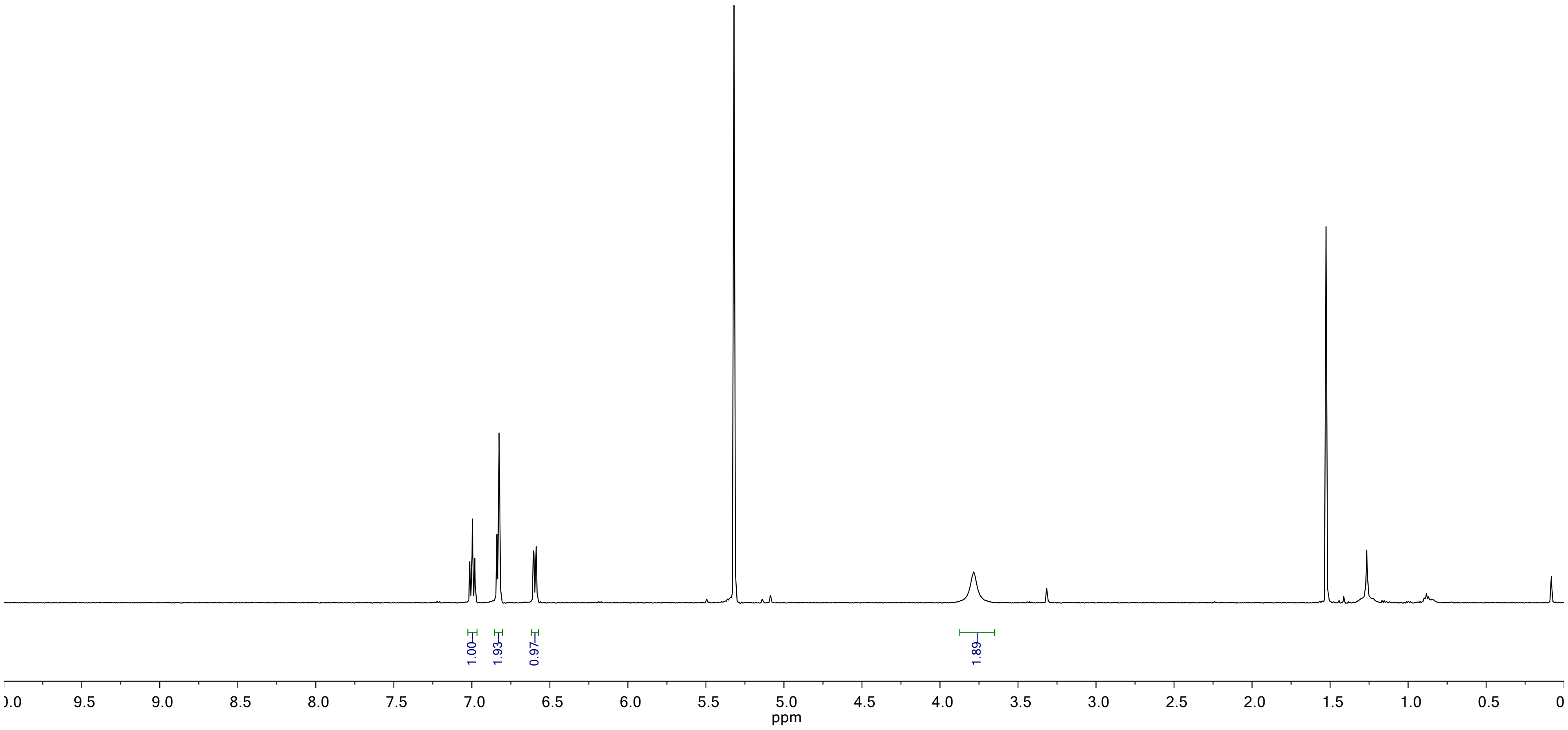

^{13}C NMR of 3-bromoaniline (S4c)

CD_2Cl_2, 23 ºC

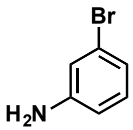

S4c

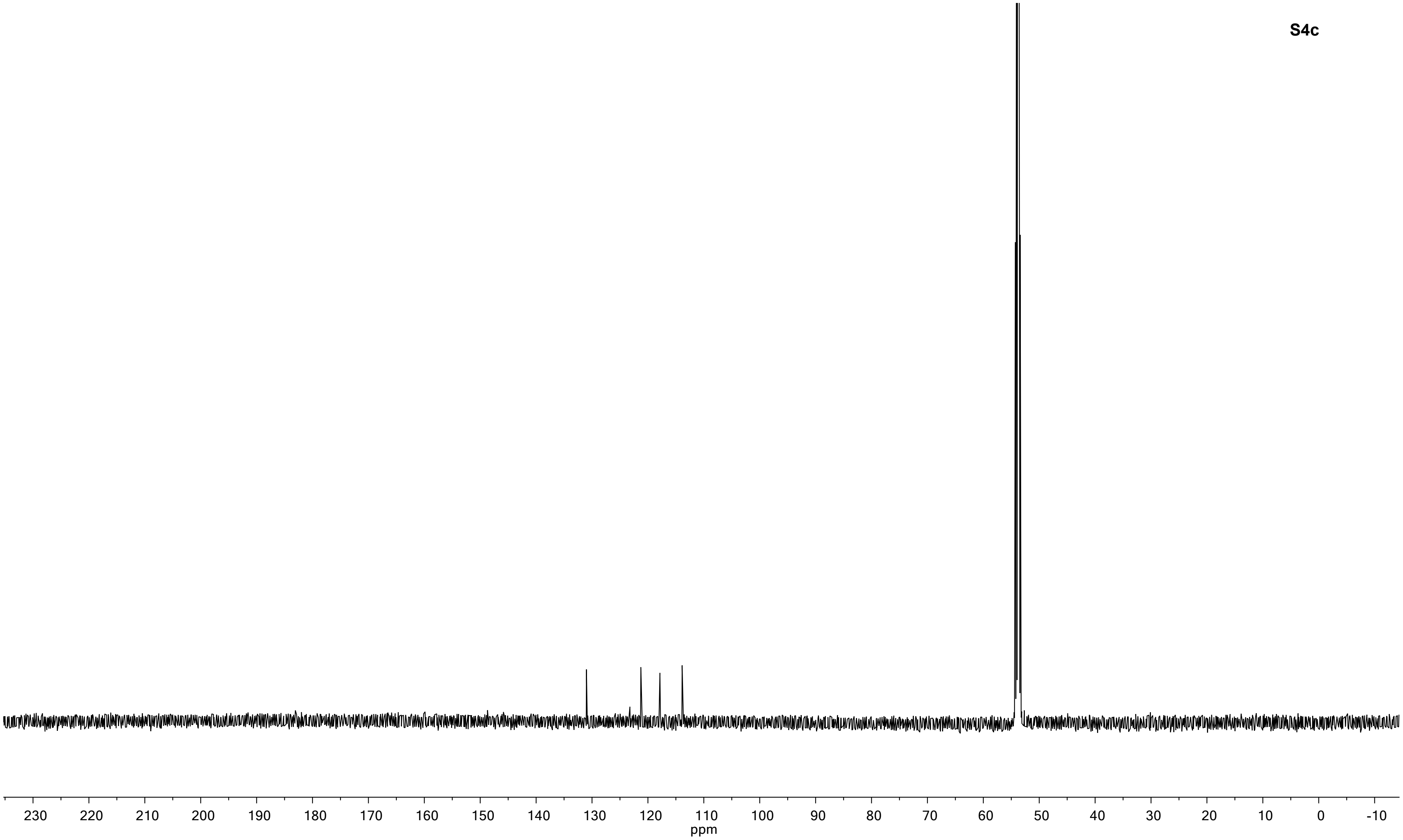

^{1}H NMR of 4-methoxyaniline (S5a)

CD_2Cl_2, 23 ºC

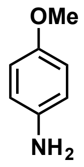

S5a

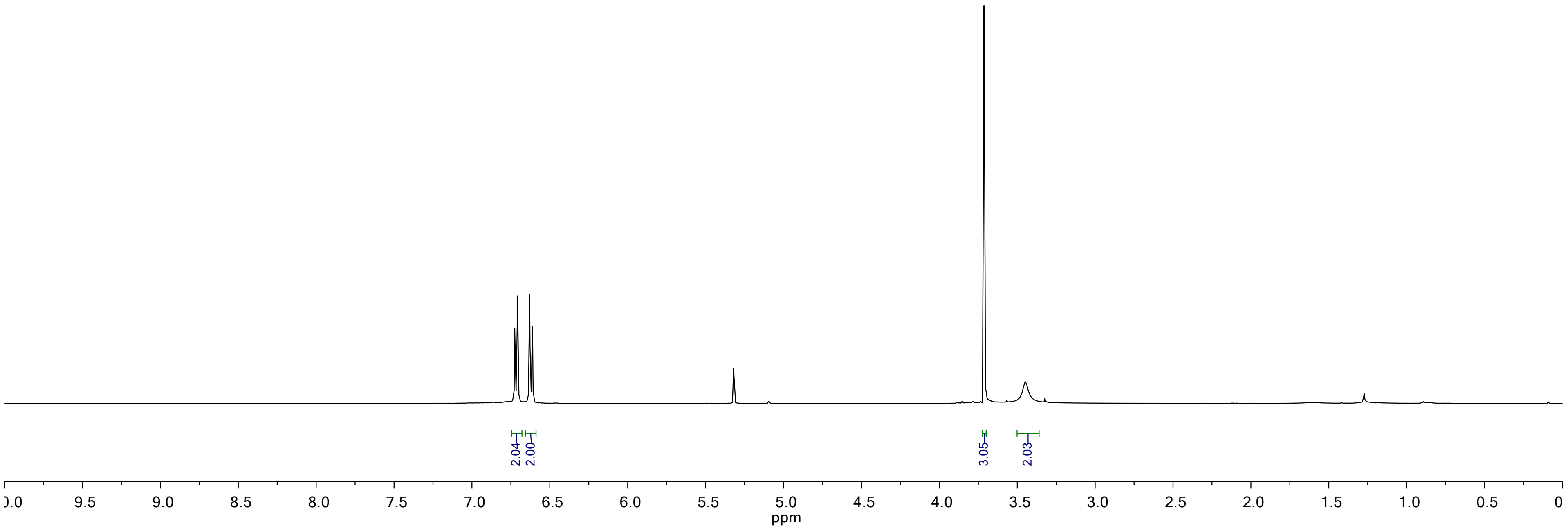

^{13}C NMR of 4-methoxyaniline (S5a)

CD_2Cl_2, 23 ºC

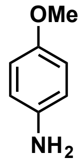

S5a

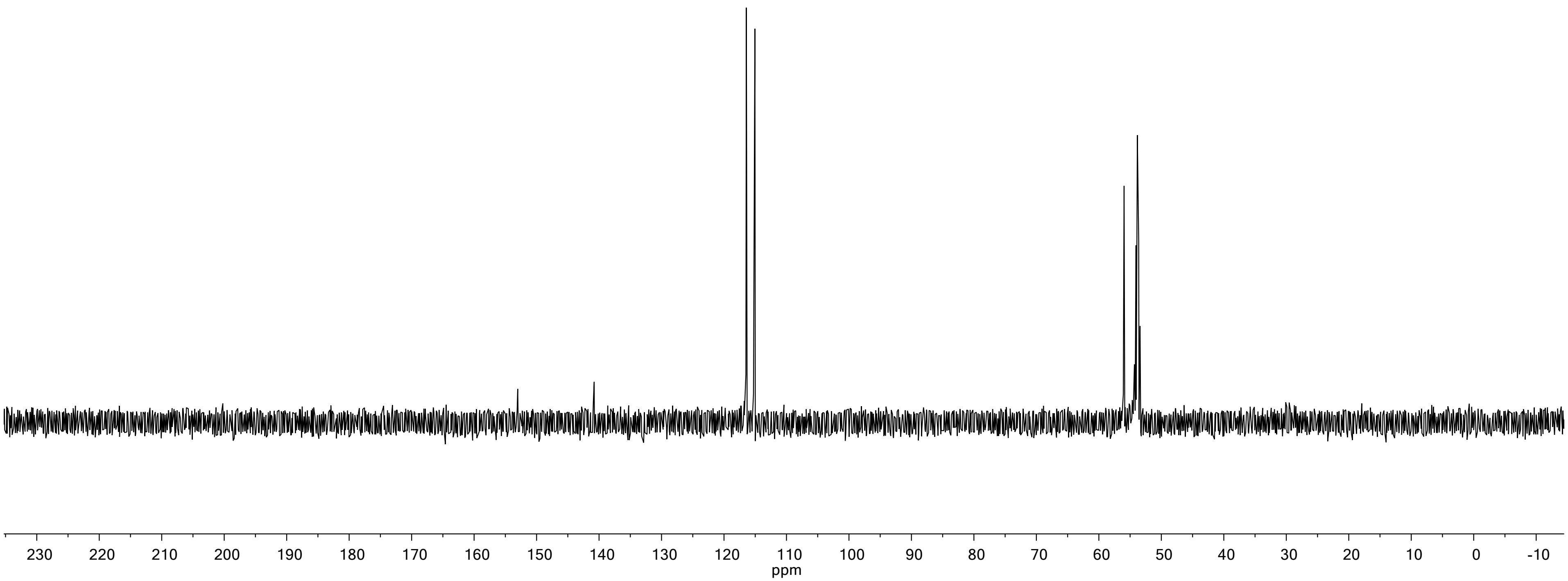

^{1}H NMR of 2-methoxyaniline (S5b)

$CDCl_3$, 23 ºC

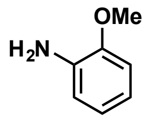

S5b

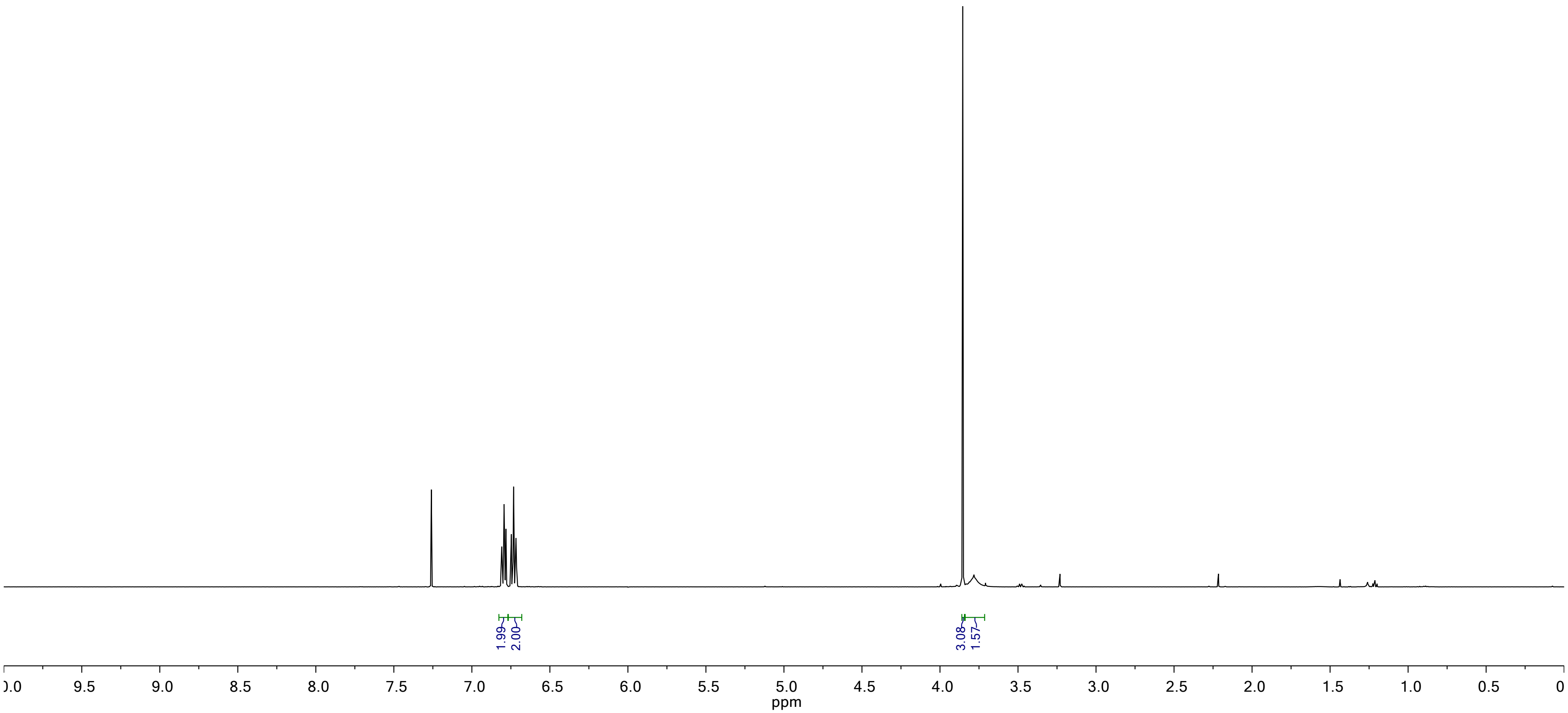

^{13}C NMR of 2-methoxyaniline (S5b)

$CDCl_3$, 23 ºC

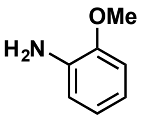

S5b

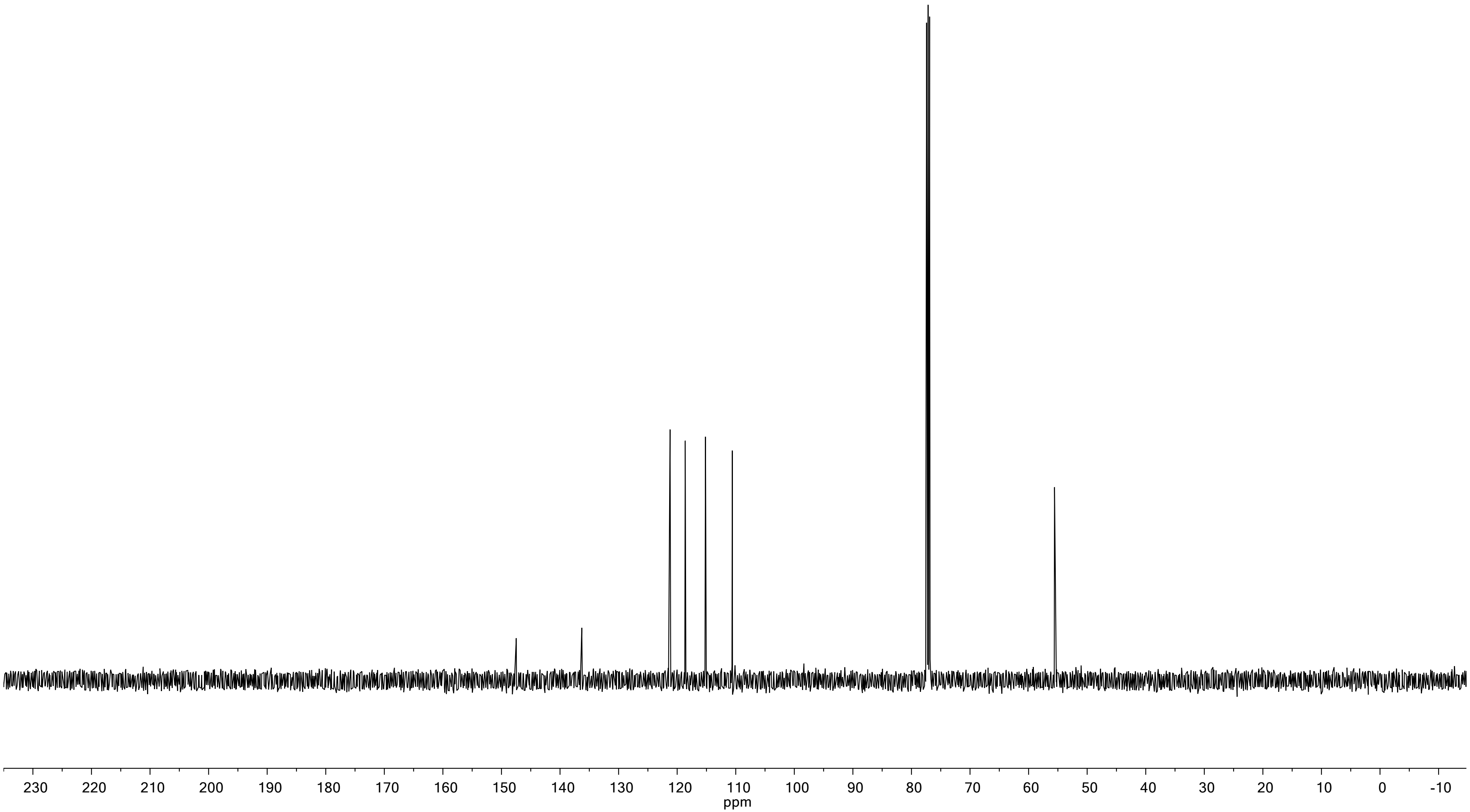

^{1}H NMR of 3-methoxyaniline (S5c)

CD_2Cl_2, 23 ºC

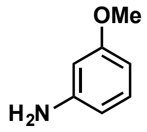

S5c

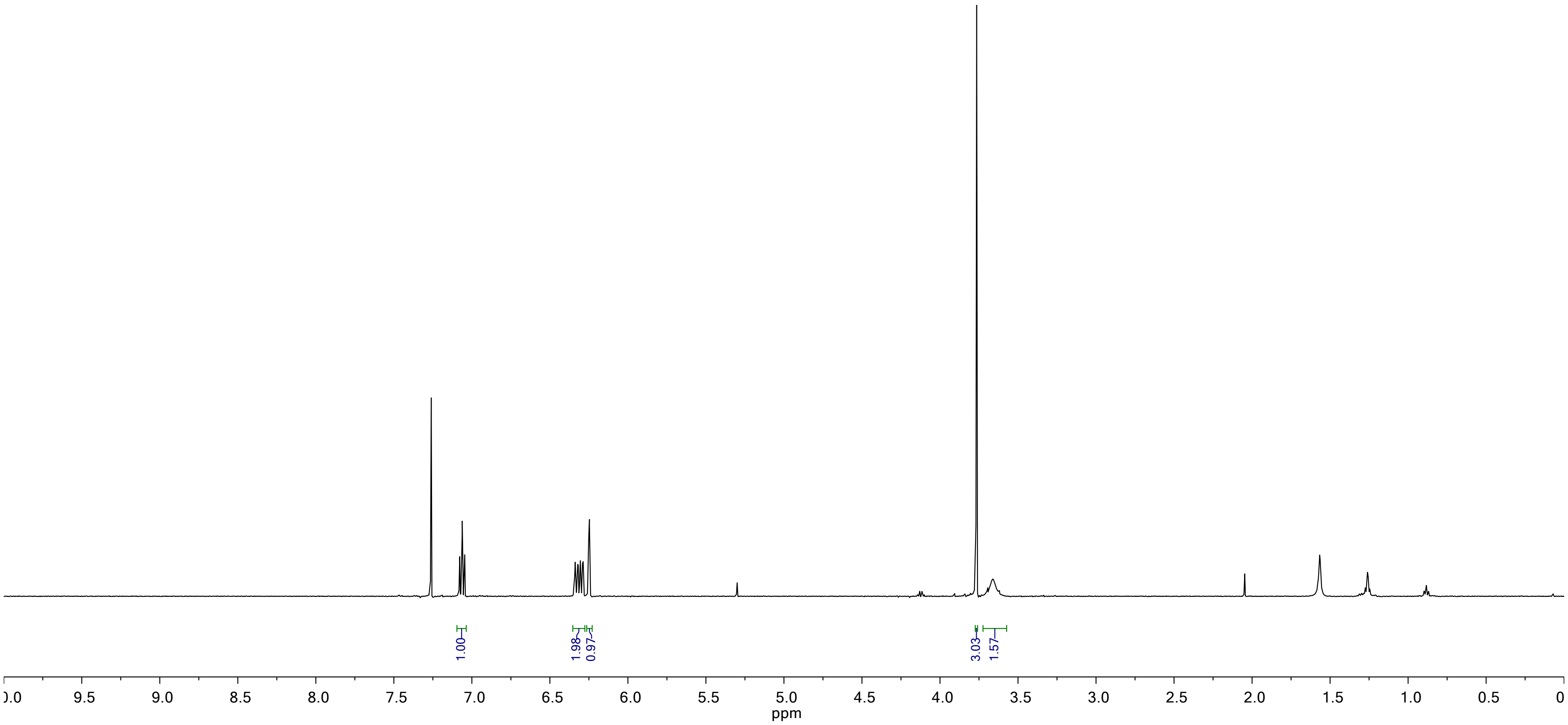

^{13}C NMR of 3-methoxyaniline (S5c)

CD_2Cl_2, 23 ºC

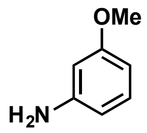

S5c

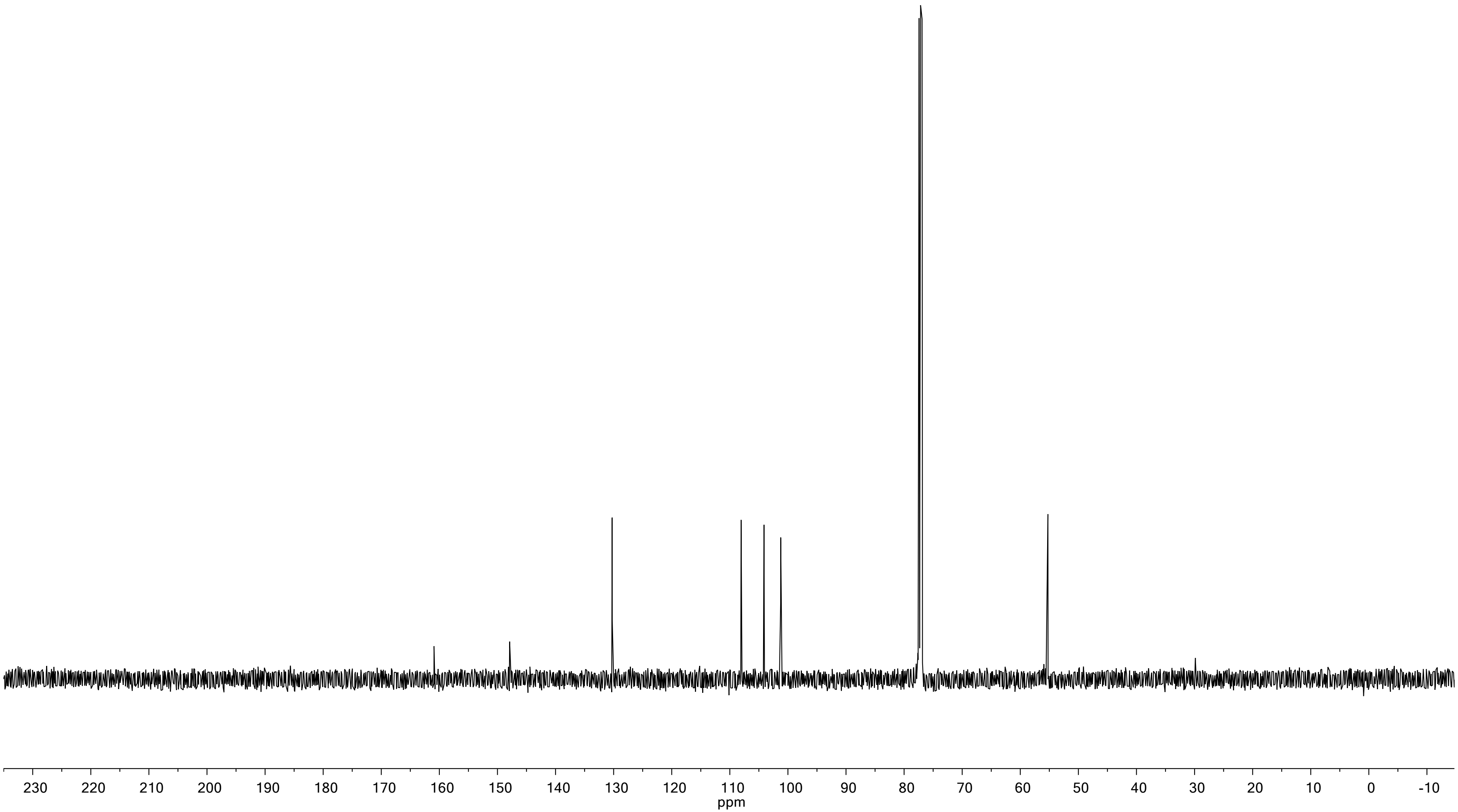

^{1}H NMR of [MsO–NH_3]ONf (S6)

$MsO{-}\overset{+}{N}H_3\ {}^{-}ONf$

S6

$(CD_3)_2CO$, 23 ºC

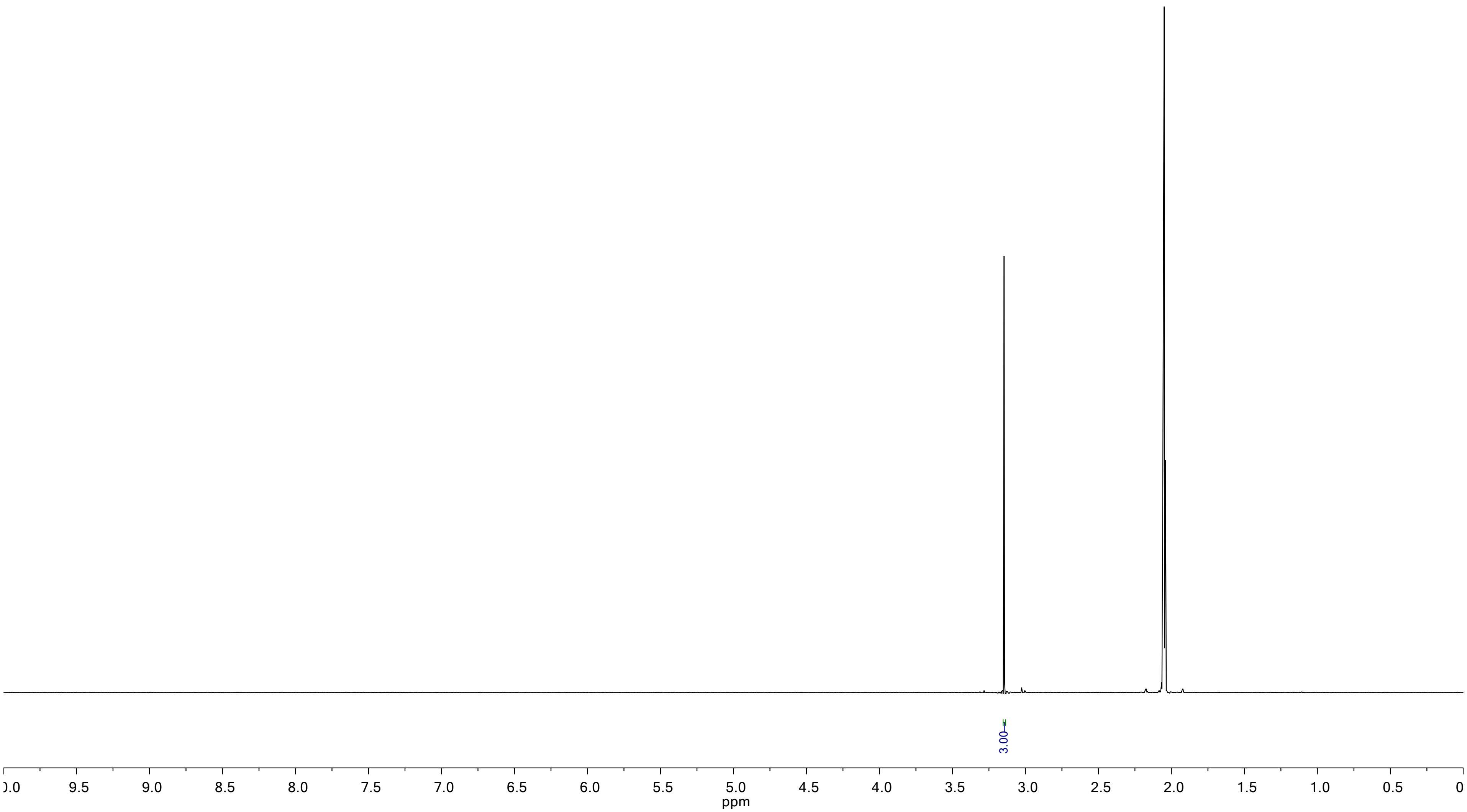

^{13}C NMR of [MsO–NH_3]ONf (S6)

$(CD_3)_2CO$, 23 ºC

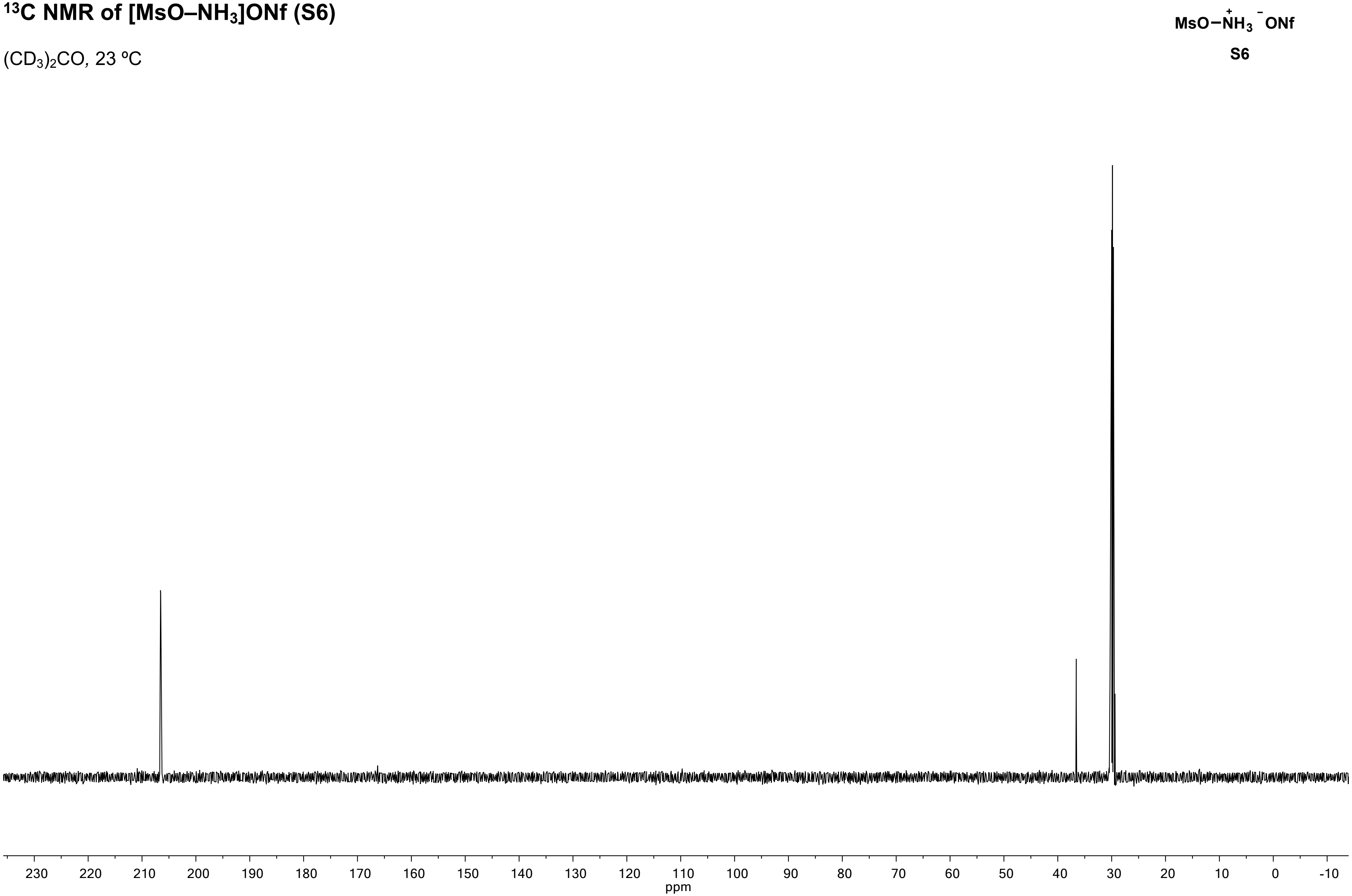

^{19}F NMR of [MsO–NH_3]ONf (S6)

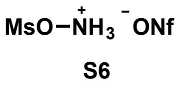

CD$_3$CN, 23 ºC

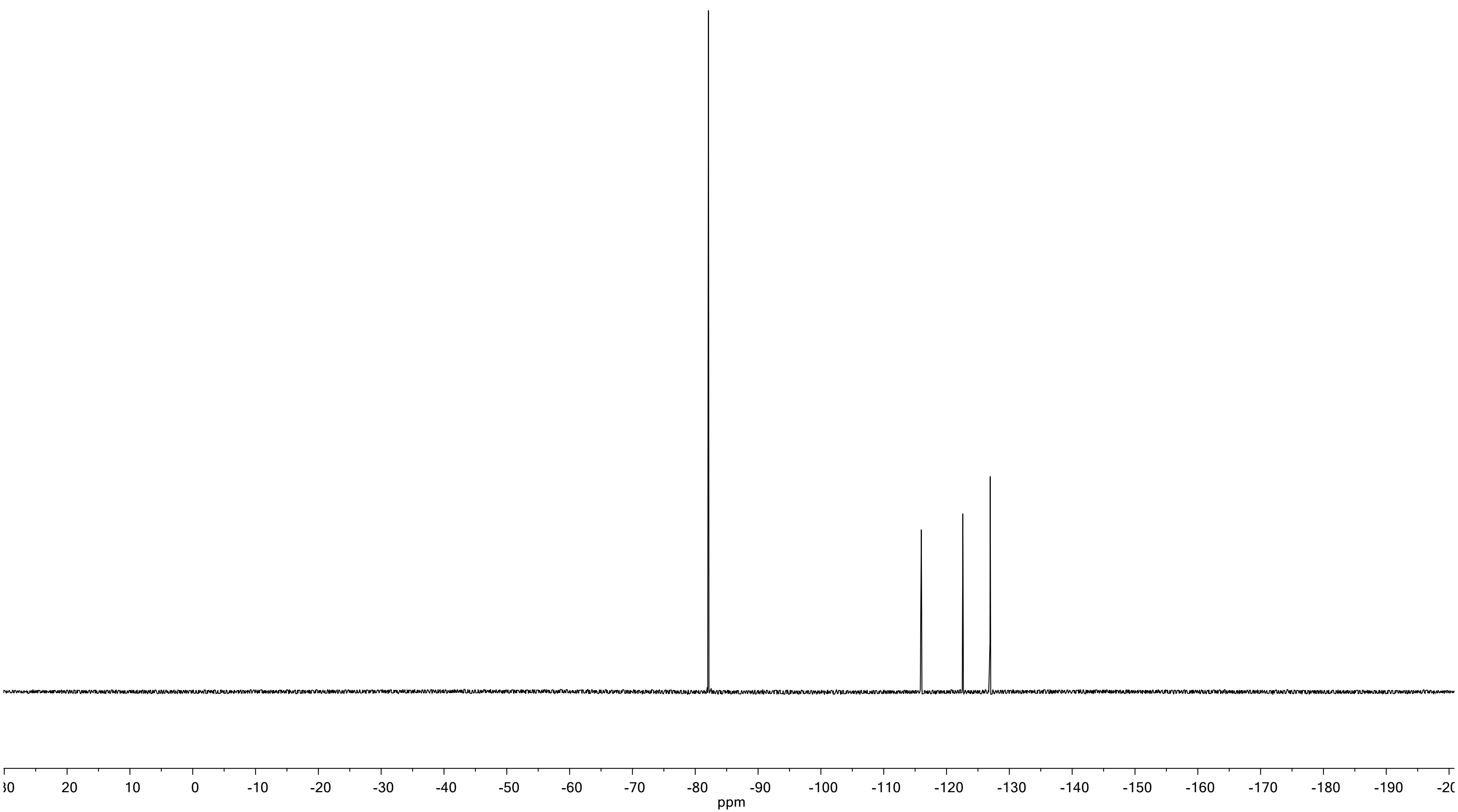

Supplement: Supplementary file 1 [file SC-010-C8SC04966A-s001.pdf]
